# Supplementary material for: Estimating the prevalence of discrepancies between study registrations and publications: a systematic review and meta-analyses
Source: BMJ Open. 2023 Oct 4;13(10):e076264. doi: 10.1136/bmjopen-2023-076264 (PMC10551944; doi:10.1136/bmjopen-2023-076264)
Supplement: Author's manuscript [file bmjopen-2023-076264.draft_revisions.pdf]

BMJ Open is committed to open peer review. As part of this commitment we make the peer review history of every article we publish publicly available.

When an article is published we post the peer reviewers' comments and the authors' responses online. We also post the versions of the paper that were used during peer review. These are the versions that the peer review comments apply to.

The versions of the paper that follow are the versions that were submitted during the peer review process. They are not the versions of record or the final published versions. They should not be cited or distributed as the published version of this manuscript.

BMJ Open is an open access journal and the full, final, typeset and author-corrected version of record of the manuscript is available on our site with no access controls, subscription charges or pay-per-view fees (http://bmjopen.bmj.com).

If you have any questions on BMJ Open's open peer review process please email
info.bmjopen@bmj.com

BMJ Open

Estimating the prevalence of discrepancies between study registrations and publications: A systematic review and meta-analyses

Journal:	*BMJ Open*
Manuscript ID	bmjopen-2023-076264
Article Type:	Original research
Date Submitted by the Author:	02-Jun-2023
Complete List of Authors:	Thibault, Robert; University of Bristol; MRC Integrative Epidemiology Unit at the University of Bristol<br>Clark, Robbie; University of Bristol<br>Pedder, Hugo ; University of Bristol; ; UK , Bristol Medical School<br>van den Akker, Olmo; Tilburg University<br>Westwood, Samuel; King's College London<br>Thompson, Jacqueline; University of Bristol<br>Munafo, Marcus; University of Bristol, Experimental Psychology
Keywords:	STATISTICS & RESEARCH METHODS, Clinical Trial, REGISTRIES

SCHOLARONE™
Manuscripts

I, the Submitting Author has the right to grant and does grant on behalf of all authors of the Work (as defined in the below author licence), an exclusive licence and/or a non-exclusive licence for contributions from authors who are: i) UK Crown employees; ii) where BMJ has agreed a CC-BY licence shall apply, and/or iii) in accordance with the terms applicable for US Federal Government officers or employees acting as part of their official duties; on a worldwide, perpetual, irrevocable, royalty-free basis to BMJ Publishing Group Ltd ("BMJ") its licensees and where the relevant Journal is co-owned by BMJ to the co-owners of the Journal, to publish the Work in this journal and any other BMJ products and to exploit all rights, as set out in our licence.

The Submitting Author accepts and understands that any supply made under these terms is made by BMJ to the Submitting Author unless you are acting as an employee on behalf of your employer or a postgraduate student of an affiliated institution which is paying any applicable article publishing charge ("APC") for Open Access articles. Where the Submitting Author wishes to make the Work available on an Open Access basis (and intends to pay the relevant APC), the terms of reuse of such Open Access shall be governed by a Creative Commons licence – details of these licences and which Creative Commons licence will apply to this Work are set out in our licence referred to above.

Other than as permitted in any relevant BMJ Author's Self Archiving Policies, I confirm this Work has not been accepted for publication elsewhere, is not being considered for publication elsewhere and does not duplicate material already published. I confirm all authors consent to publication of this Work and authorise the granting of this licence.

For peer review only

Title page as per BMJ Open requirements.

Estimating the prevalence of discrepancies between study registrations and publications: A systematic review and meta-analyses

We are submitting this manuscript under the name of a single group/consortium authorship: **"TARG Meta-Research Group & Collaborators"**.

This manuscript includes several **contributors** as stated in the contributorship statement before the references. The contributors include:

Robert T. Thibault*[1,2,7], Robbie Clark[1,2], Hugo Pedder[3], Olmo van den Akker[4], Samuel Westwood[5,6], Jacqueline Thompson[1,2], Marcus Munafò[1,2]

1 School of Psychological Science, University of Bristol, United Kingdom

2 MRC Integrative Epidemiology Unit at the University of Bristol, United Kingdom

3 Department Population Health Sciences, Bristol Medical School, University of Bristol, United Kingdom

4 Department of Methodology and Statistics, Tilburg School of Social and Behavioral Sciences, Tilburg University, Netherlands

5 Department of Child & Adolescent Psychiatry, Institute of Psychiatry, Psychology, Neuroscience, King's College London, United Kingdom

6 Department of Psychology, Institute of Human Sciences, Faculty of Education, Health and Wellbeing, University of Wolverhampton, United Kingdom

7 Meta-Research Innovation Center at Stanford (METRICS), Stanford University, United States

*Corresponding contributor. 12a Priory Road, BRISTOL BS8 1TU, United Kingdom, robert.thibault@stanford.edu, telephone: +44 117 33 17902, fax: NA

Keywords: registration; selective reporting; outcome switching; meta-research; reproducibility

Word count: 3748

For peer review only

Estimating the prevalence of discrepancies between study registrations and publications: A systematic review and meta-analyses

TARG Meta-Research Group & Collaborators

Abstract

Objectives. Prospectively registering study plans in a permanent time-stamped and publicly accessible document is becoming more common across disciplines and aims to reduce risk of bias and make risk of bias transparent. Selective reporting persists, however, when researchers deviate from their registered plans without disclosure. This systematic review aims to estimate the prevalence of undisclosed discrepancies between prospectively registered study plans and their associated publication. We further aim to identify the research disciplines where these discrepancies have been observed, whether interventions to reduce discrepancies have been conducted, and gaps in the literature.

Design. Systematic review and meta-analyses.

Data sources. Scopus and Web of Knowledge, published up to 15 December 2019.

Eligibility criteria. Articles that included quantitative data about discrepancies between registrations or study protocols and their associated publications.

Data extraction and synthesis. Each included article was independently coded by two of four reviewers using a coding form designed for this review (osf.io/728ys). We used random-effects meta-analyses to synthesize the results.

Results. We reviewed k = 89 articles, which included k = 70 that report on primary outcome discrepancies from n = 6314 studies and, k = 22 that report on secondary outcome discrepancies from n = 1436 studies. Meta-analyses indicate that between 29% to 37% (95% confidence interval) of studies contain at least one primary outcome discrepancy and between 50% to 75% (95% confidence interval) contain at least one secondary outcome discrepancy. Almost all articles assessed clinical literature, and there was considerable heterogeneity. We identified only one article that attempted to correct discrepancies.

Conclusions. Many articles did not include information on whether discrepancies were disclosed, which version of a registration they compared publications to, and whether the registration was prospective. Thus, our estimates represent discrepancies broadly, rather than our target of *undisclosed* discrepancies between *prospectively* registered study plans and their associated publications. Discrepancies are common and reduce the trustworthiness of medical research. Interventions to reduce discrepancies could prove valuable.

Registration. osf.io/ktmdg. Protocol amendments are listed in Supplementary Material A.

For peer review only

Strengths and limitations of this study.

- We employ a wide-reaching search strategy and captured 89 articles including over 6,000 registrations and publications.
- Our coding procedure includes fine-grained information that allowed us to run meta-regressions and test whether several parameters impact discrepancies.
- All our data and code are openly available.
- The high heterogeneity in the meta-analyses led to wide-ranging confidence intervals and prediction intervals.
- Many articles did not fully operationalize their definition of what constitutes a discrepancy (e.g., which version of the registration was used).

For peer review only

Introduction

In 2000, ClinicalTrials.gov and the ISRCTN Registry were launched with several aims, including aiding participant recruitment, facilitating knowledge synthesis, and reducing duplication, publication bias and selective reporting [1]. In 2005, the International Committee of Medical Journal Editors (ICMJE) made *prospective* registration a condition of consideration for publication [2]. Thousands of journals now claim to follow this policy [3]. In parallel, the World Health Organization International Clinical Trials Registry Platform established a minimum set of required information for a trial to be considered fully registered, including experimental design elements such as the conditions being studied, intervention, key inclusion and exclusion criteria, sample size, primary outcomes, and key secondary outcomes [4]. While the relatively widespread uptake of clinical trial registration has substantially improved transparency, many trials remain unregistered, are registered after enrollment of participants begins or analyses are complete (i.e., *retrospective* registration), are never published, or publish outcomes discrepant with those in the registration without disclosing the discrepancy [e.g., 5,6]. Nevertheless, the existence of registries allows researchers to identify and quantify these issues.

In research disciplines other than clinical trials, study registration is becoming more common, but remains far from standard practice [e.g., 109-111]. For example, since about 2011 the field of psychology has increasingly taken the "replication crisis" seriously and many researchers and journals now use registration to reduce bias and make risk of bias transparent. Other disciplines have created dedicated registries, such as PROSPERO for systematic reviews and the American Economic Association RCT Trial Registry.

Here we systematically review articles that quantify the prevalence of discrepancies between registrations or study protocols and their associated publications (e.g., in primary outcome measures). Our analysis extends beyond the three systematic reviews already published on this topic in several ways [7–9]. First, registration has expanded beyond clinical trials; we included all research disciplines and used key word searches for registries including the Open Science Framework, the American Economic Association RCT Trial Registry, and PROSPERO. Second, we extracted more fine-grained information about a wide range of discrepancies (e.g., outcomes, analysis, sample size), as well as which version of the registration was surveyed and whether discrepancies were disclosed (we believe disclosed discrepancies present little reason for concern). Third, our review includes over twice as many studies as previous systematic reviews on this topic, provides meta-analytic estimates, and uses meta-regression and additional analyses to attempt to identify predictors of discrepancies.

Methods

Terminology

We present a systematic review of k = 89 articles that assessed a wide range of outcome discrepancies and non-outcome discrepancies across over n = 7,000 studies. To avoid confusion, this report consistently uses the terms *studies* to refer to the over n = 7,000

individual studies that were *assessed*, and the term *article* to refer to the k = 89 articles that assessed these studies, and that we *reviewed*. We restrict our usage of the term *publication* to refer to the publications stemming from the *studies* (not to refer to the *articles*). We use the term *discrepancy* to refer to any incongruity between the content of a publication and its associated registration (e.g., on clinicaltrials.gov) or study protocol (e.g., submitted to an ethics review board or funding agency)—see Box 1 for examples. We use the term *prospective registration* broadly to include terms used in different research disciplines, such as prospective trial registration, preregistration, and pre-analysis plans. All these terms indicate the registration of study details *before* commencing a study, or in some cases, before viewing the data or removing the blind. They are in contrast to *retrospective* registration, which occurs after participant enrollment begins or analyses are complete. We use the term *outcome discrepancy* to indicate a discrepancy in the outcome measure registered versus the outcome measure reported in a publication (not to indicate a discrepancy in the value of a reported outcome between these documents).

Box 1. Examples of discrepancies.

We coded 10 types of outcome discrepancies (listed in Table 2) and 10 types of non-outcome discrepancies (listed in Table 4). The degree to which the information in a registration is discrepant with the information in a publication can range widely[1]. The associated concern about risk of bias can also range widely and often requires domain expertise to assess. We present two examples from [54] in this box. Many more examples are available in the appendix of [54] and at https://www.compare-trials.org/results.

Registered: Primary outcome: Visual Acuity [Time Frame: 6 months].

Published: Primary outcome: 3-year cumulative incidence rate of myopia. Myopia was defined as a spherical equivalent refractive error (sphere +½ cylinder) of at least −0.50 D

Coded as: Timing of outcome measurement changed.

Registered: Primary outcome: Severity of Device and Procedure related complications [Time Frame: At the time of ExAblate Transcranial thalamotomy procedure]. Secondary outcome: Effectiveness of the ExAblate Transcranial MRgFUS treatment determined using the Clinical Rating Scale for Tremor (CRST) [Time Frame: Participants will be followed from the date of treatment until study completion, approximately up to 12 months].

Published: Primary outcome: Change from baseline to 3 months in the tremor score for the hand derived from the CRST, Part A (three items: resting, postural, and action or intention components of hand tremor), and the CRST, Part B (five tasks involving handwriting, drawing, and pouring).

Coded as: Secondary outcome promoted to primary outcome; Timing of outcome measurement changed; Primary outcome omitted.

clinical trial study protocols instead of registrations [112]. Study protocols are generally far more thorough and are more likely to be updated when trialists change their plans (which may occur before starting a study, but after registering). Study protocols contain timestamps, yet they often become public only at the time of publication.

Eligibility criteria

We included articles that reported quantitative data about discrepancies between registrations or study protocols and their associated publication. We excluded conference proceedings and articles written in a language other than English (for full inclusion and exclusion criteria, see our preregistered protocol at osf.io/ktmdg).

Study selection

We searched Scopus and Web of Science on 15 December 2019 using the queries in Appendix A and Appendix B of our preregistered protocol (osf.io/ktmdg). Briefly, our queries included (1) variations of the terms preregistration, pre-analysis plans, and prospective registration in the title or keyword fields; (2) terms indicating discrepancies such as "outcome switching" in the title, keywords or abstract; (3) names of registration or protocol repositories such as "clinicaltrials.gov" in the title or keywords; and excluded overlapping but irrelevant terms (e.g., "nursing preregistration"). To limit the number of irrelevant articles, we did not search for variations of the term preregistration or for repository names in the abstract field.

Our search returned 4,283 articles after duplicates were removed (see Figure 1 for a PRISMA flowchart). Articles were screened independently by two reviewers in two stages. In Stage One, reviewers screened titles and, if necessary, briefly examined abstracts of articles to determine inclusion in the systematic review or in a scoping review on prospective registration[2]. If at least one of the reviewers deemed an article potentially relevant, it was included in Stage Two screening. In Stage Two, the reviewers independently examined the remaining 464 abstracts in greater detail for eligibility. Disagreements were resolved through discussion between the two reviewers and eventual consensus. Inter-rater agreement for the 464 articles was Cohen's k = 0.67 for inclusion in the systematic review (the list of articles and coding is available at osf.io/wa62f). Inter-rater agreement for all 4,283 articles was Cohen's k = 0.72. We then used a snowball method and identified 33 additional articles that met our inclusion criteria, mostly through citations in [7] and [9]. These 33 additional articles are not included in the inter-rater agreement scores. After full-text review, we included 89 articles in our systematic review.

****Insert Figure 1 around here****

Coding items

Each included article was independently coded by two of four reviewers (RTT, RC, OvdA, SW) using a coding form designed for this review. The form consisted of five sections that assessed (1) article characteristics, (2) study registration details, (3) 10 types of outcome discrepancies (listed in Table 2), (4) 10 types of non-outcome discrepancies (listed in Table 4), and (5) any additional descriptive or inferential statistics on discrepancies. The form details the operationalization of each variable we coded, and is available at osf.io/728ys. We

[2] We had planned to report on this scoping review separately. However, the scoping review has been terminated (details provided at https://osf.io/ktmdg).

chose items to code based on a pilot test of our protocol, as well as the categories used in a seminal paper [10] and a recent systematic review on discrepancies [7].

The data we extracted was often presented as summary results in a table and sometimes as text in the results section. To be included in our meta-analyses this data had to include at least two of (1) the number of studies assessed (denominator), (2) the number of studies with a given discrepancy (numerator), and (3) the percentage of studies with a given discrepancy—form which we could calculate an unreported numerator or denominator. We did not access the raw data. If an article did not report data for a certain measure (e.g., secondary outcome discrepancies), then we did not include that article in the meta-analysis for that measure (this is why k varies among the meta-analyses we present —see Tables 2 and 4). For the meta-regressions, we could not find data on (1) the version of the registry that publications were compared to for 35 articles, and (2) the number of studies that disclosed discrepancies for 57 articles. We coded these cases as "not reported" and included "not reported" as a factor in the meta-regressions. All other meta-regression data was complete. Further coding details are available in Supplementary Material B.

The complete dataset, including the coding of each reviewer and the resolved coding, is available at osf.io/ue2c6. A cleaned dataset with only the resolved coding is available at osf.io/6cn9m.

Statistical analyses

We performed two main random effects meta-analyses: one on the proportion of studies with at least one primary outcome discrepancy, and another on the proportion of studies with at least one secondary outcome discrepancy. We used random effects models because they allow for the true effect to vary across the populations the articles sampled from, and the articles we reviewed differ in their methodologies and the research disciplines that they assess. We used a random intercept logistic regression model with the Knapp-Hartung adjustment for the synthesis of proportions [113]. We used the maximum-likelihood method for estimating the between-study heterogeneity (Tau). We also performed meta-regressions to test whether article characteristics are associated with the proportion of studies with at least one primary or secondary outcome discrepancy.

For pooled estimates, we report both confidence intervals and prediction intervals. Whereas researchers are likely more familiar with confidence intervals, interpreting confidence intervals can be unintuitive [11], and their pooled-estimate does not incorporate uncertainty due to the between-article heterogeneity. If we assume that we could resample from our population, 95% of the resampled *meta-analyses* would yield a 95% confidence interval that contains the true value of the parameter being estimated (e.g., prevalence of discrepancies). Alternatively, if we are interested in the results that would come from another *article* assessing discrepancies, we would want a 95% prediction interval. In other words, of 100 articles drawn from the same population, we could expect the results from 95 of them—on average—to fall within the 95% prediction interval. While prediction intervals are not commonly reported, methodologists recommend reporting them for random effects meta-analysis, particularly when few articles are included or, as in our case, included articles are highly heterogeneous [12,13].

For peer review only

Whereas we did not perform a formal risk of bias assessment—because our review differed substantially from the purpose these tools were built for—we shed light on a few potential sources of bias with additional analyses that consider the funding source, statistical significance, and the timing of registration of included *studies*. These additional analyses were not prospectively registered. We made a few amendments to our preregistered study protocol which are listed in Supplementary Material A.

Results

Articles characteristics

We identified and reviewed k = 89 articles that report at least one type of discrepancy. Articles that checked for outcome discrepancies assessed a median of 68 studies (IQR: 33 to 112). Article characteristics are outlined in Table 1. All articles except for two, one preprint in economics [14] and one preprint in psychology [15], focused on clinical trials or systematic reviews. All but k = 10 articles were solely observational. Only one article attempted to correct published discrepancies [16]. The authors assessed all trials published in five journals over a six-week period and sent a letter to the editor for each trial that published a discrepant outcome (for 58 letters in total). In the time since our literature search, at least two interventional studies were published [114, 115]. One reports a trial that attempted to reduce discrepancies at medical journals by sending peer reviewers information about the study registration [114]. They found null results. The other was a feasibility study that assigned a peer reviewer to specifically check for discrepancies in manuscripts submitted for publication [115]. Further details about article characteristics are available in Supplementary Material C.

Table 1. Article characteristics

**Article characteristic**	***k* = 89**	***n* = 6929**
**Discipline**		
medicine	81	6452
dentistry	3	254
psychology	3	68
economics	1	93
physical therapy	1	62
**Source of registration or protocol assessed for discrepancies**		
registry	73	6107
ethics application	7	146
other protocol	5	466
marketing application	2	126
grant application	2	84
**Sources searched to identify studies**		
journals	32	3264
registries	23	1547

For peer review only

Table 1. Article characteristics

Article characteristic	*k* = 89	*n* = 6929
search engines	16	1527
ethics boards	7	146
funders	3	96
registries and search engines	3	27
regulators	2	126
research group	2	44
registries and journals	1	152
**Type of study**		
solely observational	79	6344
observational and study authors were contacted	9	518
observational and interventional	1	67
**Version of registry that publications were compared to**		
original	29	2607
most recent	15	1281
other version/unclear	10	670
not reported	35	2371
**Number of studies within each article that disclosed discrepancies**		
one or more	19	1547
none	9	441
article excluded publications with disclosed discrepancies	4	326
not reported	57	4615

Registration timing

Articles varied in the level of detail they provided about whether and when studies were registered. For example, whereas some articles presented their sample only after selecting for prospectively registered studies, other articles detailed their selection process including how many studies were registered and if so, when they were registered. Using the terminology in the articles we reviewed, articles identified studies that were registered retrospectively (k = 29), registered during participant enrollment (k = 17), registered after participant enrollment was complete (k = 14), and studies that were not registered (k = 36)[3]. Many articles were ambiguous regarding when some studies were registered (k = 47) and whether or not some studies were registered at all (k = 24). While these data do not provide fine-grained detail, they highlight two overarching issues: many studies are not registered,

[3] By definition, non-registered studies cannot be assessed for discrepancies between their registration and publication. Several articles identified non-registered studies in their sampling process, but these studies were not included in the final sample of articles that compared registrations to publications.

For peer review only

and many registered studies are registered retrospectively. These studies fail to meet the Declaration of Helsinki [17] (item 35) requirement that "Every research study involving human subjects must be registered in a publicly accessible database before recruitment of the first subject" and the equivalent International Committee of Medical Journal Editors (ICMJE) policy [18], which thousands of journals claim to follow [3].

Eighty of the k = 89 articles we reviewed report at least one type of outcome discrepancy. Of these, 23 report only on studies that were unambiguously prospectively registered, 51 do not unambiguously distinguish between prospectively and retrospectively registered studies, and 6 report outcome discrepancies separately for each of prospectively and retrospectively registered studies. Separate meta-analyses for unambiguously prospectively registered studies and studies with unclear timing of registration are presented in Supplementary Material D.

Forty-six of the k = 89 articles report at least one non-outcome discrepancy (e.g., in sample size or analyses). Of these, 12 report only on studies that were unambiguously prospectively registered, 33 do not unambiguously distinguish between prospectively and retrospectively registered studies, and one reports non-outcome discrepancies separately for each of prospectively and retrospectively registered studies.

Primary outcome discrepancies

An estimated 29-37% (95% confidence interval) of the population of studies contain at least one primary outcome discrepancy (Figure 2). The 95% prediction interval is 10-68%.

****Insert Figure 2 around here****

This meta-analysis had high heterogeneity (I^2 = 86%), suggesting that the broad range of estimates across the articles stem largely from differences in the methodology of the articles or populations they sample from, rather than from chance. Heterogeneity could not be explained by meta-regression of any of the following article-level characteristics: discipline (p = 0.28)[4], whether the publications were compared to registry entries versus other protocol formats (e.g., ethics applications) (p = 0.46), sources searched to identify studies (p = 0.65), version of the registry analyzed (p = 0.77)[5], whether discrepancies were disclosed (p = 0.97), and year of article publication (p = 0.83). The meta-regression on discipline had low power because 63 articles assessed medical research and 7 assessed studies across dentistry, psychology, physical therapy, and economics. To increase statistical power, we reran this meta-regression after dichotomizing discipline and found that studies in disciplines other than medicine may have a greater proportion of studies with at least one primary outcome discrepancy (p = 0.09; OR 95% CI: 0.91-3.19). We ran another meta-regression after dichotomizing the source which publications were compared to into

---

[4] If readers want to find if a study on discrepancies exists within a particular sub-discipline, we invite them to explore the variable 'disciplineDetail' in our dataset.

registrations versus other protocols and did not find evidence to suggest this moderator is playing a role (p = 0.42). We also conducted a sensitivity analysis that included all six regressors in a single meta-regression and found that publications compared to the most recent version of a registration (k = 15) may have a smaller proportion of studies with at least one primary outcome discrepancy as compared to publications compared to the original version of a registration (k = 29) (p = 0.08; OR 95% CI: 0.32 to 1.07). All meta-regression model summaries are presented in Supplementary Material E.

The high heterogeneity in this meta-analysis may stem from genuine differences among the articles, including the sub-disciplines surveyed, specific sources searched, definition of a discrepancy (e.g., whereas some articles considered a change in the timing of an outcome as a discrepancy, others did not), and other article characteristics that may or may not have been reported. Our dataset contains more fine-grained information about the specific sub-discipline surveyed and specific sources searched. While we do not further explore these potential moderators in the present report, we note that, whereas some sub-disciplines and sources were highly specific (e.g., cystic fibrosis, lung cancer immunotherapy, Global Resource of Eczema Trials database), others were broad (e.g., medicine, clinicaltrials.gov, core clinical MEDLINE journals). We did not collect information on the exact definitions an article used to identify a primary outcome discrepancy. However, we did collect information on the proportion of articles with sub-categories of outcome discrepancies, which are more strictly defined and listed in Table 2 (e.g., promoting a secondary outcome to a primary outcome). We ran meta-analyses on these sub-categories of outcome discrepancies and found they also had high heterogeneity (Table 2). Thus, varying definitions are unlikely to be the main driver of the high heterogeneity in the present analysis on primary outcome discrepancies.

Secondary outcome discrepancies

An estimated 50-75% (95% confidence interval) of the population of studies contain at least one secondary outcome discrepancy (Figure 3). The 95% prediction interval is 13-95% .

****Insert Figure 3 around here****

This meta-analysis also had high heterogeneity (I^2 = 90%) which could not be explained by meta-regression of the version of the registry analyzed (p = 0.8) or the year of article publication (p = 0.72). Meta-regression of the sources searched to identify studies explained some heterogeneity in that searches stemming from journals, compared to registries, had a greater proportion of publications with at least one secondary outcome discrepancy (p = 0.03; OR 95% CI: 1.89-13.45). Meta-regressions on discipline (p = 0.29), whether discrepancies were disclosed (p = 0.68), and whether the publications were compared to registry entries versus other protocol formats (p = 0.08) had very low statistical power because almost all articles had the same characteristic. All meta-regression model summaries are included in Supplementary Material E.

Descriptively, omitting secondary outcomes and adding secondary outcomes appears to occur more frequently than omitting primary outcomes, adding primary outcomes, or

For peer review only

demoting primary outcomes, which in turn appear to occur more frequently than promoting a secondary outcome (see Table 2).

Table 2. Meta-analytic estimates for the proportion of studies that contain various types of outcome discrepancies

	**Discrepant studies (95% CI)**	**Discrepant studies (95% PI)**	**k**	**n**
Any outcome discrepancy	41-75%	7-97%	18	1113
Any primary outcome discrepancy	29-37%	10-68%	70	6314
Any secondary outcome discrepancy	50-75%	13-95%	22	1436
Primary outcome demoted to secondary outcome	6-10%	2-31%	51	4560
Primary outcome omitted	6-12%	1-43%	51	4338
Primary outcome added	7-11%	2-34%	54	4697
Secondary outcome promoted to primary outcome	4-6%	1-19%	46	4135
Secondary outcome omitted	16-35%	4-72%	18	1243
Secondary outcome added	19-43%	3-83%	20	1305
Timing of outcome measurement changed	6-16%	1-62%	30	3472

We only coded 'any outcome discrepancy' for articles that checked for both primary and secondary outcome discrepancies in the studies they assessed.

Parameters potentially related to outcome discrepancies

A subset of articles contained information on parameters potentially related to the proportion of outcome discrepancies. These include the disclosure of discrepancies, presence of a 'statistically significant' result, funding source, and timing of registration (Table 3; see Supplementary Material F for forest plots).

For peer review only

Table 3. Additional analyses regarding discrepancies

**Analysis**	**95% CI**	**95% PI**	**k**	**n**
Percentage of studies with at least one outcome discrepancy that disclose an outcome discrepancy	4-19%	0.3-74%	21	1747
Percentage of outcome discrepancies that favored statistically significant results	49-66%	23-86%	24	2216
Likelihood ratio of a study with versus without an outcome discrepancy to contain statistically significant results	0.56-1.06	0.42-1.43	7	508
*Likelihood ratio of a study with versus without a statistically significant results to contain an outcome discrepancy	0.64-0.99	0.59-1.06	7	508
Likelihood ratio of a study with versus without industry funded to contain an outcome discrepancy	0.61-0.91	0.44-1.27	22	2585
Likelihood ratio of a prospectively registered study versus retrospectively registered study to contain a primary outcome discrepancy	0.46-2.57	0.13-8.85	4	261

* This analysis uses the same data as the likelihood ratio analysis before it. One of these seven articles checked discrepancies in analyses, rather than discrepancies in outcome measures.

Non-outcome discrepancies

The meta-analyses for the non-outcome discrepancies have high heterogeneity and both wide confidence intervals and prediction intervals (Table 4). Articles varied in the criteria they used to identify non-outcome discrepancies and there were fewer articles than for outcome discrepancies. Prediction intervals can be particularly imprecise when few articles are included in a meta-analysis [116]. Whereas our coding procedure divided outcome discrepancies into 10 sub-categories, it did not employ the same level of granularity for non-outcome discrepancies. Supplementary Material G contains additional information on non-outcome discrepancies.

For peer review only

Table 4. Meta-analytic estimates for the proportion of studies that contain various types of non-outcome discrepancies

	Discrepant studies (95% CI)	Discrepant studies (95% PI)	k	n
Eligibility criteria	25-57%	5-90%	15	1153
Sample size	26-44%	8-78%	25	1398
Randomization	2-64%	0.06-98%	5	176
Blinding	5-42%	0.04-99%	3	224
Intervention	3-52%	0.1-97%	7	550
Study duration	3-89%	0.02-99.94%	4	184
Analysis	19-52%	4-86%	12	404
Subgroup analysis	35-93%	2-99.7%	9	545
Funding	7-84%	0.2-99.5%	5	212
Results	7-82%	0.2-99%	6	262

Values less than 0.5% and greater than 99.5% are rounded to one significant digit from 0 or 100.

Gaps in the literature

We identified several gaps in the literature on discrepancies. There exists little research on: (1) the prevalence of discrepancies in fields other than clinical research, (2) the prevalence of discrepancies in a representative sample across clinical disciplines, (3) the level of specificity in registrations, and (4) interventions to reduce undisclosed discrepancies (see Supplementary Material H for additional information about these gaps). We also identified several themes from surveying the conclusions of the articles we reviewed. These include the need for awareness surrounding discrepancies, the need for mandates, enforcement, and/or new initiatives to address discrepancies, and the benefit of registering additional information such as analysis plans (see Supplementary Table H1).

For peer review only

Discussion

We find that outcome measures in registrations and study protocols often differ from published outcome measures, that the prevalence of discrepancies varies substantially across the articles we reviewed, and that this heterogeneity is not easily assigned to specific article characteristics.

Limitations

Given the wide range of discrepancy prevalence across individual articles, point estimates and confidence intervals may provide false precision when extrapolating our findings to the registered literature at large. Moreover, because heterogeneity could not be explained by meta-regression of article characteristics, more precise estimates cannot be derived for subsets of the literature. The prediction intervals can reasonably be used to extrapolate to another article in the registered medical literature at large, although the included studies do not necessarily form a representative sample.

Comparison to previous research

Our main findings are in line with those from previous systematic reviews. These reviews included 27 articles each and found that 31% of studies had a primary outcome discrepancy in the median article they reviewed [9] and 54% of studies had any outcome discrepancy in the median article they reviewed [7]. The latter review did not distinguish between primary and secondary outcomes, and many articles they reviewed only assessed primary outcomes. Our review included all the articles contained in these systematic reviews, except for a few that did not meet our inclusion criteria (e.g., a PhD thesis, an abstract).

Implications

Our review raises broader issues regarding the efficiency of the research ecosystem and the trustworthiness of research outputs. We identified articles that documented discrepancies between publications and all of registrations, protocols, ethics applications, funding applications, and marketing approval applications. The existence of multiple documents outlining the same study raises the likelihood of discrepancies and, in the absence of a clearly demarcated 'master' document, leaves ambiguity regarding which document is 'correct'. Rehashing the same study details for different audiences may also be an inefficient use of researchers' time. Identifying a single publicly accessible document as the version of record (this could be the registration) and having all other documents point to this version of record for key information could reduce ambiguity and improve efficiency.

As for trustworthiness, registration has had a clearly positive influence on medical research [117, 118]. At the same time, some registration policies have poor adherence (e.g., many trials are registered retrospectively, and many trial results are never reported [19]). The existence of research policies that are regularly overlooked, rarely monitored, and come with no consequence for non-compliance, can be damaging in at least two ways. They risk devaluing research policies altogether and they can reduce the trustworthiness of research outputs by creating a false impression that rigorous research practices were employed.

Conceiving research as a complex ecosystem comprised of various agents with diverse incentives (e.g., funders, publishers, institutions, individual researchers) can help to comprehend why some policies have poor adherence and to develop and implement effective research infrastructure.

Conclusion

Registrations provide the evidence to detect selective reporting and outcome switching, which we found to be common. Nearly all articles we reviewed focused on documenting issues. Future efforts regarding discrepancies—and research improvement broadly—could prove more fruitful by shifting focus towards developing and testing solutions to these now well-documented issues.

Supplementary material

osf.io/byqhp

Funding

Robert Thibault is supported by a postdoctoral fellowship from the *Fonds de la recherche en santé du Québec*. Hugo Pedder was funded by the NIHR Biomedical Research Centre at University Hospitals Bristol and Weston NHS Foundation Trust and the University of Bristol. The views expressed are those of the authors and not necessarily those of the NIHR or the Department of Health and Social Care. Olmo van den Akker is supported by a Consolidator Grant (IMPROVE) from the European Research Council (ERC; grant no. 726361). Robbie Clark is supported by a SWDTP ESRC +3 PhD studentship. Jacqueline Thompson was funded by a grant from Jisc during the course of this research. Marcus Munafò, Robert Thibault, Jacqueline Thompson, and Robbie Clark are part of the MRC Integrative Epidemiology Unit (MC_UU_00011/7). The funders had no role in study design, data collection and analysis, decision to publish, or preparation of the manuscript.

Transparency statement

Robert Thibault, the manuscript's guarantor, affirms that the manuscript is an honest, accurate, and transparent account of the study being reported; that no important aspects of the study have been omitted; and that any discrepancies from the study as originally planned have been explained.

Data Availability

All data and analysis code will be openly shared on the University of Bristol Data Repository upon acceptance for publication. At present, these materials are publicly available at osf.io/5gfty

For peer review only

Patient and public involvement

No patient involved.

Competing interests

None declared.

Contributors

Contributions according to Contributor Roles Taxonomy (CRediT) (casrai.org/credit/)

Conceptualization	Robert Thibault(1,2), Jacqueline Thompson(1,2), Marcus Munafò(1,2)
Data curation	Robert Thibault
Formal Analysis	Robert Thibault, Hugo Pedder(3)
Funding acquisition	Robert Thibault, Marcus Munafò
Investigation	Robert Thibault, Robbie Clark(1,2), Olmo van den Akker(4), Samuel Westwood(5,6)
Methodology	Robert Thibault, Robbie Clark, Marcus Munafò
Project administration	Robert Thibault
Resources	not applicable
Software	Robert Thibault, Hugo Pedder
Supervision	Robert Thibault, Marcus Munafò
Validation	Robert Thibault
Visualization	Robert Thibault
Writing – original draft	Robert Thibault
Writing – review & editing	Robert Thibault, Jacqueline Thompson, Hugo Pedder, Robbie Clark, Olmo van den Akker, Marcus Munafò

[1] School of Psychological Science, University of Bristol. robert.thibault@bristol.ac.uk, Reseach Associate; jackie.thompson@bristol.ac.uk, Senior Research Associate; marcus.munafo@bristol.ac.uk, Professor; robbie.clark.2018@bristol.ac.uk, PhD Student
[2] MRC Integrative Epidemiology Unit at the University of Bristol
[3] Department Population Health Sciences, Bristol Medical School, University of Bristol. hugo.pedder@bristol.ac.uk, Senior Research Associate
[4] Department of Methodology and Statistics, Tilburg School of Social and Behavioral Sciences, Tilburg University. o.r.vdnakker@tilburguniversity.edu, PhD Student
[5] Department of Child & Adolescent Psychiatry, Institute of Psychiatry, Psychology, Neuroscience, King's College London. s.westwood4@wlv.ac.uk, Lecturer
[6] Department of Psychology, Institute of Human Sciences, Faculty of Education, Health and Wellbeing, University of Wolverhampton

Figure captions

Figure 1. PRISMA flowchart of article inclusion

Figure 2. Forest plot of articles reporting the proportion of assessed studies with at least one primary outcome discrepancy.

Figure 3. Forest plot of articles reporting the proportion of assessed studies with at least one secondary outcome discrepancy.

References

1 Zarin DA, Tse T, Williams RJ, *et al.* Update on Trial Registration 11 Years after the ICMJE Policy Was Established. *New England Journal of Medicine* 2017;**376**:383–91. doi:10.1056/NEJMsr1601330

2 De Angelis C, Drazen JM, Frizelle FA, *et al.* Clinical Trial Registration: A Statement from the International Committee of Medical Journal Editors. *New England Journal of Medicine* 2004;**351**:1250–1. doi:10.1056/NEJMe048225

3 ICMJE. Journals stating that they follow the ICMJE Recommendations. 2021.

4 Sim I, Chan A-W, Gülmezoglu AM, *et al.* Clinical trial registration: Transparency is the watchword. *The Lancet* 2006;**367**:1631–3. doi:10.1016/S0140-6736(06)68708-4

5 Chan AW, Pello A, Kitchen J, *et al.* Association of Trial Registration With Reporting of Primary Outcomes in Protocols and Publications. *JAMA* 2017;**318**:1709. doi:10.1001/jama.2017.13001

6 Scott A, Rucklidge JJ, Mulder RT. Is mandatory prospective trial registration working to prevent publication of unregistered trials and selective outcome reporting? An observational study of five psychiatry journals that mandate prospective clinical trial registration. *PLoS ONE* 2015;**10**:1–3. doi:10.1371/journal.pone.0133718

7 Li G, Abbade LPF, Nwosu I, *et al.* A systematic review of comparisons between protocols or registrations and full reports in primary biomedical research. *BMC Medical Research Methodology* 2018;**18**:9. doi:10.1186/s12874-017-0465-7

8 Dwan K, Gamble C, Williamson PR, *et al.* Systematic Review of the Empirical Evidence of Study Publication Bias and Outcome Reporting Bias An Updated Review. *PLOS ONE* 2013;**8**:e66844. doi:10.1371/journal.pone.0066844

9 Jones CW, Keil LG, Holland WC, *et al.* Comparison of registered and published outcomes in randomized controlled trials: A systematic review. *BMC MEDICINE* 2015;**13**. doi:10.1186/s12916-015-0520-3

10 Chan AW, Hróbjartsson A, Haahr MT, *et al. Empirical evidence for selective reporting of outcomes in randomized trials: Comparison of protocols to published articles*. 2004. doi:10.1001/jama.291.20.2457

11 Hoekstra R, Morey RD, Rouder JN, *et al.* Robust misinterpretation of confidence intervals. *Psychonomic Bulletin and Review* 2014;**21**:1157–64. doi:10.3758/s13423-013-0572-3

For peer review only

12 Riley RD, Higgins JPT, Deeks JJ. Interpretation of random effects meta-analyses. *BMJ* 2011;**342**:d549. doi:10.1136/bmj.d549

13 Higgins JPT, Thompson SG, Spiegelhalter DJ. A re-evaluation of random-effects meta-analysis. *Journal of the Royal Statistical Society Series A, (Statistics in Society)* 2009;**172**:137–59. doi:10.1111/j.1467-985X.2008.00552.x

14 Ofosu G, Posner DN. Pre-analysis Plans: A Stocktaking. MetaArXiv 2019. doi:10.31222/osf.io/e4pum

15 Claesen A, Gomes SLBT, tuerlinckx francis, *et al.* Preregistration: Comparing Dream to Reality. PsyArXiv 2019. doi:10.31234/osf.io/d8wex

16 Goldacre B, Drysdale H, Dale A, *et al.* COMPare: A prospective cohort study correcting and monitoring 58 misreported trials in real time. *Trials* 2019;**20**:1–6. doi:10.1186/s13063-019-3173-2

17 World Medical Association. Declaration of Helsinki Ethical Principles for Medical Research Involving Human Subjects. 2013.

18 ICMJE. Recommendations for the Conduct, Reporting, Editing, and Publication of Scholarly Work in Medical Journals. 2019.

19 DeVito NJ, Bacon S, Goldacre B. FDAAA TrialsTracker: A live informatics tool to monitor compliance with FDA requirements to report clinical trial results. *bioRxiv* 2019;266452. doi:10.1101/266452

20 Ross A, George D, Wayant C, *et al.* Registration Practices of Randomized Clinical Trials in Rhinosinusitis: A Cross-sectional Review. *JAMA OTOLARYNGOLOGY-HEAD & NECK SURGERY* 2019;**145**:468–74. doi:10.1001/jamaoto.2019.0145

21 Wiebe J, Detten G, Scheckel C, *et al.* The heart of the matter: Outcome reporting bias and registration status in cardio-thoracic surgery. *INTERNATIONAL JOURNAL OF CARDIOLOGY* 2017;**227**:299–304. doi:10.1016/j.ijcard.2016.11.098

22 Shinohara K, Tajika A, Imai H, *et al.* Protocol registration and selective outcome reporting in recent psychiatry trials: New antidepressants and cognitive behavioural therapies. *ACTA PSYCHIATRICA SCANDINAVICA* 2015;**132**:489–98. doi:10.1111/acps.12502

23 Saquib N, Saquib J, Ioannidis JPA. Practices and impact of primary outcome adjustment in randomized controlled trials: Meta-epidemiologic study. *BMJ (Online)* Published Online First: 2013. doi:10.1136/bmj.f4313

24 Chan AW, Krleža-Jerić K, Schmid I, *et al.* Outcome reporting bias in randomized trials funded by the Canadian Institutes of Health Research. *CMAJ* Published Online First: 2004. doi:10.1503/cmaj.1041086

For peer review only

25 Redmond S, Von Elm E, Blümle A, *et al.* Cohort study of trials submitted to ethics committee identified discrepant reporting of outcomes in publications. *Journal of Clinical Epidemiology* Published Online First: 2013. doi:10.1016/j.jclinepi.2013.06.020

26 Wayant C, Scheckel C, Hicks C, *et al.* Evidence of selective reporting bias in hematology journals: A systematic review. *PLOS ONE* 2017;**12**. doi:10.1371/journal.pone.0178379

27 Jones CW, Adams A, Weaver MA, *et al.* Peer reviewed evaluation of registered end-points of randomised trials (the PRE-REPORT study): Protocol for a stepped-wedge, cluster-randomised trial. *BMJ Open* 2019;**9**:1–8. doi:10.1136/bmjopen-2018-028694

28 Pandis N, Fleming PS, Worthington H, *et al.* Discrepancies in Outcome Reporting Exist Between Protocols and Published Oral Health Cochrane Systematic Reviews. *PLOS ONE* 2015;**10**. doi:10.1371/journal.pone.0137667

29 Su C-X, Han M, Ren J, *et al.* Empirical evidence for outcome reporting bias in randomized clinical trials of acupuncture: Comparison of registered records and subsequent publications. *TRIALS* 2015;**16**. doi:10.1186/s13063-014-0545-5

30 Delgado AF, Delgado AF. Inconsistent Reporting Between Meta-analysis Protocol and Publication - A Cross-Sectional Study. *ANTICANCER RESEARCH* 2017;**37**:5101–7. doi:10.21873/anticanres.11928

31 Dwan K, Kirkham JJ, Williamson PR, *et al.* Selective reporting of outcomes in randomised controlled trials in systematic reviews of cystic fibrosis. *BMJ OPEN* 2013;**3**. doi:10.1136/bmjopen-2013-002709

32 IntHout J, Ioannidis JPA, Rovers MM, *et al.* Plea for routinely presenting prediction intervals in meta-analysis. *BMJ Open* 2016;**6**:e010247. doi:10.1136/bmjopen-2015-010247

33 Rosenthal R, Dwan K. Comparison of Randomized Controlled Trial Registry Entries and Content of Reports in Surgery Journals. *ANNALS OF SURGERY* 2013;**257**:1007–15. doi:10.1097/SLA.0b013e318283cf7f

34 Sendyk DI, Rovai ES, Souza NV, *et al.* Selective outcome reporting in randomized clinical trials of dental implants. *Journal of Clinical Periodontology* 2019;**46**:758–65. doi:10.1111/jcpe.13128

35 Soares HP, Daniels S, Kumar A, *et al.* Bad reporting does not mean bad methods for randomised trials: Observational study of randomised controlled trials performed by the Radiation Therapy Oncology Group. *British Medical Journal* Published Online First: 2004. doi:10.1136/bmj.328.7430.22

36 Fleming PS, Koletsi D, Dwan K, *et al.* Outcome Discrepancies and Selective Reporting: Impacting the Leading Journals? *PLOS ONE* 2015;**10**. doi:10.1371/journal.pone.0127495

37 Korevaar DA, Ochodo EA, Bossuyt PMM, *et al.* Publication and Reporting of Test Accuracy Studies Registered in ClinicalTrials.gov. *CLINICAL CHEMISTRY* 2014;**60**:651–9. doi:10.1373/clinchem.2013.218149

For peer review only

38 Huić M, Marušić M, Marušić A. Completeness and changes in registered data and reporting bias of randomized controlled trials in ICMJE journals after trial registration policy. *PLoS ONE* Published Online First: 2011. doi:10.1371/journal.pone.0025258

39 Jurić D, Bolić A, Pranić S, *et al.* Drugdrug interaction trials incompletely described drug interventions in ClinicalTrials.gov and published articles: An observational study. *Journal of Clinical Epidemiology* 2020;**117**:126–37. doi:10.1016/j.jclinepi.2019.10.002

40 Pranić S, Marušić A. Changes to registration elements and results in a cohort of Clinicaltrials.gov trials were not reflected in published articles. *Journal of Clinical Epidemiology* 2016;**70**:26–37. doi:10.1016/j.jclinepi.2015.07.007

41 Rosati P, Porzsolt F, Ricciotti G, *et al.* Major discrepancies between what clinical trial registries record and paediatric randomised controlled trials publish. *TRIALS* 2016;**17**. doi:10.1186/s13063-016-1551-6

42 Shepshelovich D, Yelin D, Gafter-Gvili A, *et al.* Comparison of reporting phase III randomized controlled trials of antibiotic treatment for common bacterial infections in ClinicalTrials.gov and matched publications. *CLINICAL MICROBIOLOGY AND INFECTION* 2018;**24**. doi:10.1016/j.cmi.2018.02.010

43 Chan AW, Hróbjartsson A, Jørgensen KJ, *et al.* Discrepancies in sample size calculations and data analyses reported in randomised trials: Comparison of publications with protocols. *BMJ* Published Online First: 2008. doi:10.1136/bmj.a2299

44 Zarin DA, Tse T, Williams RJ, *et al.* The ClinicalTrials.gov Results Database - Update and Key Issues. *NEW ENGLAND JOURNAL OF MEDICINE* 2011;**364**:852–60. doi:10.1056/NEJMsa1012065

45 Hardwicke TE, Serghiou S, Janiaud P, *et al.* Calibrating the Scientific Ecosystem Through Meta-Research. *Annual Review of Statistics and Its Application* 2020;**7**:11–37. doi:10.1146/annurev-statistics-031219-041104

46 Aggarwal R, Oremus M. Selective outcome reporting is present in randomized controlled trials in lung cancer immunotherapies. *JOURNAL OF CLINICAL EPIDEMIOLOGY* 2019;**106**:145–6. doi:10.1016/j.jclinepi.2018.10.010

47 Al-Marzouki S, Roberts I, Evans S, *et al.* *Selective reporting in clinical trials: Analysis of trial protocols accepted by The Lancet.* 2008. doi:10.1016/S0140-6736(08)61060-0

48 Anand V, Scales DC, Parshuram CS, *et al.* Registration and design alterations of clinical trials in critical care: A cross-sectional observational study. *Intensive Care Medicine* Published Online First: 2014. doi:10.1007/s00134-014-3250-7

49 Azar M, Riehm KE, Saadat N, *et al.* Evaluation of Journal Registration Policies and Prospective Registration of Randomized Clinical Trials of Nonregulated Health Care Interventions. *JAMA INTERNAL MEDICINE* 2019;**179**:624–32. doi:10.1001/jamainternmed.2018.8009

For peer review only

50 Bonnot B, Yavchitz A, Mantz J, *et al.* Selective primary outcome reporting in high-impact journals of anaesthesia and pain. *BRITISH JOURNAL OF ANAESTHESIA* 2016;**117**:542–3. doi:10.1093/bja/aew280

51 Boonacker CWB, Hoes AW, Van Liere-Visser K, *et al.* A comparison of subgroup analyses in grant applications and publications. *American Journal of Epidemiology* Published Online First: 2011. doi:10.1093/aje/kwr075

52 Braakhekke M, Scholten I, Mol F, *et al.* Selective outcome reporting and sponsorship in randomized controlled trials in IVF and ICSI. *HUMAN REPRODUCTION* 2017;**32**:2117–22. doi:10.1093/humrep/dex273

53 Bradley HA, Rucklidge JJ, Mulder RT. A systematic review of trial registration and selective outcome reporting in psychotherapy randomized controlled trials. *ACTA PSYCHIATRICA SCANDINAVICA* 2017;**135**:65–77. doi:10.1111/acps.12647

54 Calméjane L, Dechartres A, Tran VT, *et al.* Making protocols available with the article improved evaluation of selective outcome reporting. *Journal of Clinical Epidemiology* 2018;**104**:95–102. doi:10.1016/j.jclinepi.2018.08.020

55 Chahal J, Tomescu SS, Ravi B, *et al.* Publication of Sports Medicine-Related Randomized Controlled Trials Registered in ClinicalTrials.gov. *AMERICAN JOURNAL OF SPORTS MEDICINE* 2012;**40**:1970–7. doi:10.1177/0363546512448363

56 Chen T, Li C, Qin R, *et al.* Comparison of Clinical Trial Changes in Primary Outcome and Reported Intervention Effect Size Between Trial Registration and Publication. *JAMA NETWORK OPEN* 2019;**2**. doi:10.1001/jamanetworkopen.2019.7242

57 Dekkers OM, Cevallos M, Bührer J, *et al.* Comparison of noninferiority margins reported in protocols and publications showed incomplete and inconsistent reporting. *Journal of Clinical Epidemiology* Published Online First: 2015. doi:10.1016/j.jclinepi.2014.09.015

58 Delgado AF, Delgado AF. Outcome switching in randomized controlled oncology trials reporting on surrogate endpoints: A cross-sectional analysis. *SCIENTIFIC REPORTS* 2017;**7**. doi:10.1038/s41598-017-09553-y

59 Ewart R, Lausen H, Millian N. Undisclosed changes in outcomes in randomized controlled trials: An observational study. *Annals of Family Medicine* Published Online First: 2009. doi:10.1370/afm.1017

60 Gandhi R, Jan M, Smith HN, *et al.* Comparison of published orthopaedic trauma trials following registration in Clinicaltrials.gov. *BMC MUSCULOSKELETAL DISORDERS* 2011;**12**. doi:10.1186/1471-2474-12-278

61 Gopal AD, Desai NR, Tse T, *et al.* Reporting of Noninferiority Trials in ClinicalTrials.gov and Corresponding Publications. *JAMA-JOURNAL OF THE AMERICAN MEDICAL ASSOCIATION* 2015;**313**:1163–5. doi:10.1001/jama.2015.1697

62 Hahn S, Williamson PR, Hutton JL. Investigation of within-study selective reporting in clinical research: Follow-up of applications submitted to a local research ethics committee. *Journal of Evaluation in Clinical Practice* Published Online First: 2002. doi:10.1046/j.1365-2753.2002.00314.x

63 Hannink G, Gooszen HG, Rovers MM. Comparison of Registered and Published Primary Outcomes in Randomized Clinical Trials of Surgical Interventions. *ANNALS OF SURGERY* 2013;**257**:818–23. doi:10.1097/SLA.0b013e3182864fa3

64 Hartung DM, Zarin DA, Guise J-M, *et al.* Reporting Discrepancies Between the ClinicalTrials.gov Results Database and Peer-Reviewed Publications. *ANNALS OF INTERNAL MEDICINE* 2014;**160**:477+. doi:10.7326/M13-0480

65 Hernández AV, Steyerberg EW, Taylor GS, *et al. Subgroup analysis and covariate adjustment in randomized clinical trials of traumatic brain injury: A systematic review*. 2005. doi:10.1227/01.NEU.0000186039.57548.96

66 Hildenbrand A, Conour C, Straus JA, *et al.* Trial Registration and Outcome Reporting in Child and Pediatric Psychology: A Systematic Review. *JOURNAL OF PEDIATRIC PSYCHOLOGY* 2019;**44**:1024–33. doi:10.1093/jpepsy/jsz054

67 Howard B, Scott JT, Blubaugh M, *et al.* Systematic review: Outcome reporting bias is a problem in high impact factor neurology journals. *PLoS ONE* 2017;**12**:1–4. doi:10.1371/journal.pone.0180986

68 Jones CW, Platts-Mills TF. Quality of Registration for Clinical Trials Published in Emergency Medicine Journals. *ANNALS OF EMERGENCY MEDICINE* 2012;**60**:458–64. doi:10.1016/j.annemergmed.2012.02.005

69 Jones PM, Chow JTY, Arango MF, *et al.* Comparison of Registered anti Reported Outcomes in Randomized Clinical Trials Published in Anesthesiology Journals. *ANESTHESIA AND ANALGESIA* 2017;**125**:1292–300. doi:10.1213/ANE.0000000000002272

70 Jones CW, Misemer BS, Platts-Mills TF, *et al.* Primary outcome switching among drug trials with and without principal investigator financial ties to industry: A cross-sectional study. *BMJ Open* 2018;**8**:1–7. doi:10.1136/bmjopen-2017-019831

71 Kasenda B, Schandelmaier S, Sun X, *et al.* Subgroup analyses in randomised controlled trials: Cohort study on trial protocols and journal publications. *BMJ (Online)* Published Online First: 2014. doi:10.1136/bmj.g4539

72 Khaleel S, Cleveland B, Kalapara A, *et al.* The fate of urological systematic reviews registered in PROSPERO. *WORLD JOURNAL OF UROLOGY* doi:10.1007/s00345-019-03032-x

73 Khan NA, Lombeida JI, Singh M, *et al.* Association of industry funding with the outcome and quality of randomized controlled trials of drug therapy for rheumatoid arthritis. *Arthritis and Rheumatism* Published Online First: 2012. doi:10.1002/art.34393

For peer review only

74 Killeen S, Sourallous P, Hunter IA, *et al.* Registration Rates, Adequacy of Registration, and a Comparison of Registered and Published Primary Outcomes in Randomized Controlled Trials Published in Surgery Journals. *ANNALS OF SURGERY* 2014;**259**:193–6. doi:10.1097/SLA.0b013e318299d00b

75 Korevaar DA, Hooft L, Askie LM, *et al.* Facilitating Prospective Registration of Diagnostic Accuracy Studies: A STARD Initiative. *CLINICAL CHEMISTRY* 2017;**63**:1331–41. doi:10.1373/clinchem.2017.272765

76 Koufatzidou M, Koletsi D, Fleming PS, *et al.* Outcome reporting discrepancies between trial entries and published final reports of orthodontic randomized controlled trials. *EUROPEAN JOURNAL OF ORTHODONTICS* 2019;**41**:225–30. doi:10.1093/ejo/cjy046

77 Krsticevic M, Saric D, Saric F, *et al.* Selective reporting bias due to discrepancies between registered and published outcomes in osteoarthritis trials. *Journal of Comparative Effectiveness Research* 2019;**8**:1265–73. doi:10.2217/cer-2019-0068

78 Li X-Q, Yang G-L, Tao K-M, *et al.* Comparison of registered and published primary outcomes in randomized controlled trials of gastroenterology and hepatology. *Scandinavian Journal of Gastroenterology* 2013;**48**:1474–83. doi:10.3109/00365521.2013.845909

79 Ludwig DS, Ebbeling CB, Heymsfield SB. Discrepancies in the Registries of Diet vs Drug Trials. *JAMA network open* 2019;**2**:e1915360. doi:10.1001/jamanetworkopen.2019.15360

80 Mathieu S, Boutron I, Moher D, *et al.* Comparison of Registered and Published Primary Outcomes in Randomized Controlled Trials. *JAMA-JOURNAL OF THE AMERICAN MEDICAL ASSOCIATION* 2009;**302**:977–84. doi:10.1001/jama.2009.1242

81 Mathieu S, Giraudeau B, Soubrier M, *et al.* Misleading abstract conclusions in randomized controlled trials in rheumatology: Comparison of the abstract conclusions and the results section. *Joint Bone Spine* Published Online First: 2012. doi:10.1016/j.jbspin.2011.05.008

82 Maund E, Tendal B, Hróbjartsson A, *et al.* Benefits and harms in clinical trials of duloxetine for treatment of major depressive disorder: Comparison of clinical study reports, trial registries, and publications. *BMJ (Online)* Published Online First: 2014. doi:10.1136/bmj.g3510

83 Mhaskar R, Djulbegovic B, Magazin A, *et al.* Published methodological quality of randomized controlled trials does not reflect the actual quality assessed in protocols. *Journal of Clinical Epidemiology* Published Online First: 2012. doi:10.1016/j.jclinepi.2011.10.016

84 Nankervis H, Baibergenova A, Williams HC, *et al.* Prospective Registration and Outcome-Reporting Bias in Randomized Controlled Trials of Eczema Treatments: A Systematic Review. *JOURNAL OF INVESTIGATIVE DERMATOLOGY* 2012;**132**:2727–34. doi:10.1038/jid.2012.231

For peer review only

85 Norris SL, Holmer HK, Fu R, *et al.* Clinical trial registries are of minimal use for identifying selective outcome and analysis reporting. *RESEARCH SYNTHESIS METHODS* 2014;**5**:273–84. doi:10.1002/jrsm.1113

86 Pinto RZ, Elkins MR, Moseley AM, *et al.* Many Randomized Trials of Physical Therapy Interventions Are Not Adequately Registered: A Survey of 200 Published Trials. *PHYSICAL THERAPY* 2013;**93**:299–309. doi:10.2522/ptj.20120206

87 Raghav KPS, Mahajan S, Yao JC, *et al.* From Protocols to Publications: A Study in Selective Reporting of Outcomes in Randomized Trials in Oncology. *JOURNAL OF CLINICAL ONCOLOGY* 2015;**33**:3583+. doi:10.1200/JCO.2015.62.4148

88 Rankin J, Ross A, Baker J, *et al.* Selective outcome reporting in obesity clinical trials: A cross-sectional review. *CLINICAL OBESITY* 2017;**7**:245–54. doi:10.1111/cob.12199

89 Riehm KE, Azar M, Thombs BD. Transparency of outcome reporting and trial registration of randomized controlled trials in top psychosomatic and behavioral health journals: A 5-year follow-up. *JOURNAL OF PSYCHOSOMATIC RESEARCH* 2015;**79**:1–2. doi:10.1016/j.jpsychores.2015.04.010

90 Rising K, Bacchetti P, Bero L. *Reporting bias in drug trials submitted to the Food and Drug Administration: Review of publication and presentation*. 2008. doi:10.1371/journal.pmed.0050217

91 Rongen JJ, Hannink G. Comparison of Registered and Published Primary Outcomes in Randomized Controlled Trials of Orthopaedic Surgical Interventions. *JOURNAL OF BONE AND JOINT SURGERY-AMERICAN VOLUME* 2016;**98**:403–9. doi:10.2106/JBJS.15.00400

92 Ruegger CM, Dawson JA, Donath SM, *et al.* Nonpublication and discontinuation of randomised controlled trials in newborns. *ACTA PAEDIATRICA* 2017;**106**:1940–4. doi:10.1111/apa.14062

93 Shepshelovich D, Goldvaser H, Wang L, *et al.* Comparison of reporting phase I trial results in ClinicalTrials.gov and matched publications. *INVESTIGATIONAL NEW DRUGS* 2017;**35**:827–33. doi:10.1007/s10637-017-0510-8

94 Smith HN, Bhandari M, Mahomed NN, *et al.* Comparison of Arthroplasty Trial Publications After Registration in ClinicalTrials.gov. *JOURNAL OF ARTHROPLASTY* 2012;**27**:1283–8. doi:10.1016/j.arth.2011.11.005

95 Smith SM, Wang AT, Pereira A, *et al.* Discrepancies between registered and published primary outcome specifications in analgesic trials: ACTTION systematic review and recommendations. *PAIN* 2013;**154**:2769–74. doi:10.1016/j.pain.2013.08.011

96 Stockmann C, Ross JS, Sherwin CMT, *et al.* Rate of asthma trial outcomes reporting on ClinicalTrials.gov and in the published literature. *JOURNAL OF ALLERGY AND CLINICAL IMMUNOLOGY* 2014;**134**:1443–6. doi:10.1016/j.jaci.2014.09.019

For peer review only

97 Thomas ET, Clark J, Glasziou P. Publication and outcome reporting of homeopathy trials registered in clinicaltrials.gov. *Focus on Alternative and Complementary Therapies* 2016;**21**:127–33. doi:10.1111/fct.12278

98 Tricco AC, Cogo E, Page MJ, *et al.* A third of systematic reviews changed or did not specify the primary outcome: A PROSPERO register study. *JOURNAL OF CLINICAL EPIDEMIOLOGY* 2016;**79**:46–54. doi:10.1016/j.jclinepi.2016.03.025

99 Vedula SS, Bero L, Scherer RW, *et al.* Outcome Reporting in Industry-Sponsored Trials of Gabapentin for Off-Label Use. *NEW ENGLAND JOURNAL OF MEDICINE* 2009;**361**:1963–71. doi:10.1056/NEJMsa0906126

100 Vedula SS, Li T, Dickersin K. Differences in Reporting of Analyses in Internal Company Documents Versus Published Trial Reports: Comparisons in Industry-Sponsored Trials in Off-Label Uses of Gabapentin. *PLoS Medicine* Published Online First: 2013. doi:10.1371/journal.pmed.1001378

101 Vera-badillo FE, Shapiro R, Ocana A, *et al.* Bias in reporting of end points of efficacy and toxicity in randomized, clinical trials for women with breast cancer. *Annals of Oncology* Published Online First: 2013. doi:10.1093/annonc/mds636

102 Walker KF, Stevenson G, Thornton JG. Discrepancies between registration and publication of randomised controlled trials: An observational study. *JRSM Short Reports* 2014;**5**:1–4. doi:10.1177/2042533313517688

103 Wandalkar P, Gandhe P, Pai A, *et al.* A study comparing trial registry entries of randomized controlled trials with publications of their results in a high impact factor journal: The Journal of the American Medical Association. *Perspectives in Clinical Research* 2017;**8**:167–71. doi:10.4103/2229-3485.215978

104 White VA, Walker KF, Thornton JG. Trials of antenatal corticosteroids for preterm fetal lung maturity: A review of the potential for selective outcome reporting. *EUROPEAN JOURNAL OF OBSTETRICS & GYNECOLOGY AND REPRODUCTIVE BIOLOGY* 2019;**236**:58–68. doi:10.1016/j.ejogrb.2019.02.031

105 Wildt S, Krag A, Gluud L. Characteristics of randomised trials on diseases in the digestive system registered in ClinicalTrials.gov: A retrospective analysis. *BMJ OPEN* 2011;**1**. doi:10.1136/bmjopen-2011-000309

106 Won J, Kim S, Bae I, *et al.* Trial registration as a safeguard against outcome reporting bias and spin? A case study of randomized controlled trials of acupuncture. *PLoS ONE* 2019;**14**. doi:10.1371/journal.pone.0223305

107 You B, Gan HK, Pond G, *et al. Consistency in the analysis and reporting of primary end points in oncology randomized controlled trials from registration to publication: A systematic review*. 2012. doi:10.1200/JCO.2011.37.0890

108 Zhang S, Liang F, Li W. Comparison between publicly accessible publications, registries, and protocols of phase III trials indicated persistence of selective outcome

For peer review only

reporting. *JOURNAL OF CLINICAL EPIDEMIOLOGY* 2017;**91**:87–94. doi:10.1016/j.jclinepi.2017.07.010

109. Hardwicke TE, Thibault RT, Kosie JE, Wallach JD, Kidwell MC, Ioannidis JP. Estimating the prevalence of transparency and reproducibility-related research practices in psychology (2014–2017). Perspectives on Psychological Science. 2022 Jan;17(1):239-51.

110. Hardwicke TE, Wallach JD, Kidwell MC, Bendixen T, Crüwell S, Ioannidis JP. An empirical assessment of transparency and reproducibility-related research practices in the social sciences (2014–2017). Royal Society Open Science. 2020 Feb 19;7(2):190806.

111. Scoggins B, Robertson MP. Measuring Transparency in the Social Sciences: Political Science and International Relations. I4R Discussion Paper Series; 2023.

112. The Editors. Discrepancies between prespecified and reported outcomes. Annals of Internal Medicine. 2016 Mar 1;164(5):374.

113. Hartung J, Knapp G. An Alternative Test Procedure for Meta-Analysis. In R. Schulze, H. Holling, & D. Böhning (Eds.), Meta-analysis: New Developments and Applications in Medical and Social Sciences (pp. 53–69). Hogrefe & Huber Publishers. 2003

114. Jones CW, Adams A, Misemer BS, Weaver MA, Schroter S, Khan H, Margolis B, Schriger DL, Platts-Mills TF. Peer Reviewed Evaluation of Registered End-Points of Randomised Trials (the PRE-REPORT study): a stepped wedge, cluster-randomised trial. BMJ open. 2022 Sep 1;12(9):e066624.

115. TARG Meta-Research Group and Collaborators*. Discrepancy review: A feasibility study of a novel peer review intervention to reduce undisclosed discrepancies between registrations and publications. Royal Society Open Science. 2022 Jul 27;9(7):220142.

116. IntHout J, Ioannidis JP, Rovers MM, Goeman JJ. Plea for routinely presenting prediction intervals in meta-analysis. BMJ open. 2016 Jul 1;6(7):e010247.

117. Kaplan RM, Irvin VL. Likelihood of null effects of large NHLBI clinical trials has increased over time. PloS one. 2015 Aug 5;10(8):e0132382.

118. De Vito NJ. Trial registries for transparency and accountability in clinical research (Doctoral dissertation, University of Oxford). https://ora.ox.ac.uk/objects/uuid:3c901316-5c24-4b42-a78e-1037d56d35e4

119. Hardwicke TE, Wagenmakers EJ. Reducing bias, increasing transparency and calibrating confidence with preregistration. Nature Human Behaviour. 2023 Jan;7(1):15-26.

For peer review only - http://bmjopen.bmj.com/site/about/guidelines.xhtml

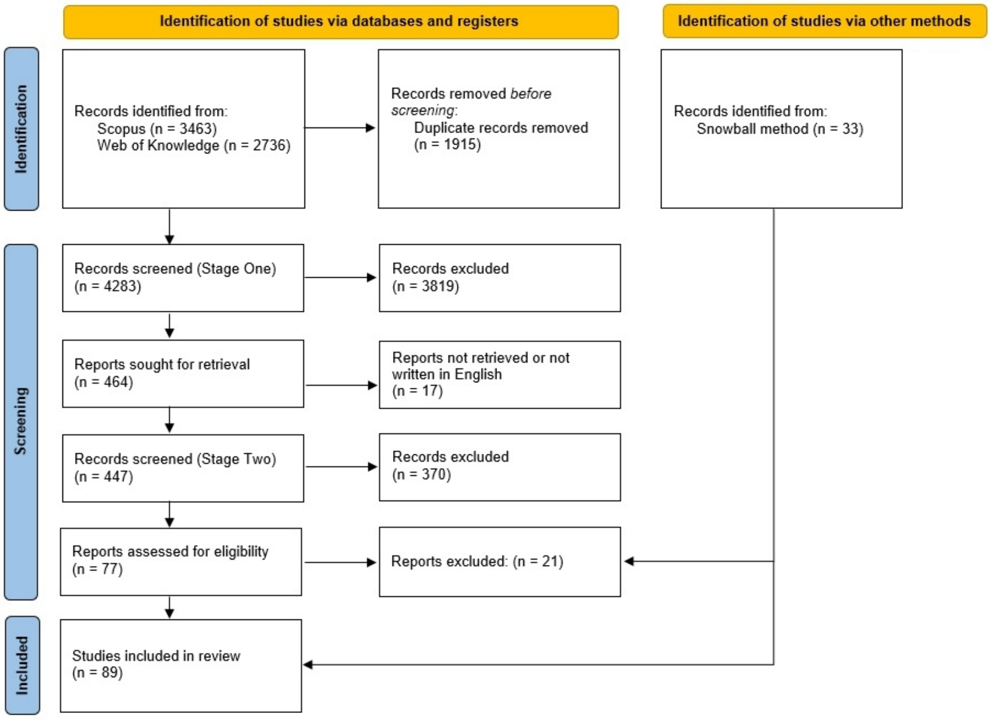

Figure 1. PRISMA flowchart of article inclusion

160x115mm (144 x 144 DPI)

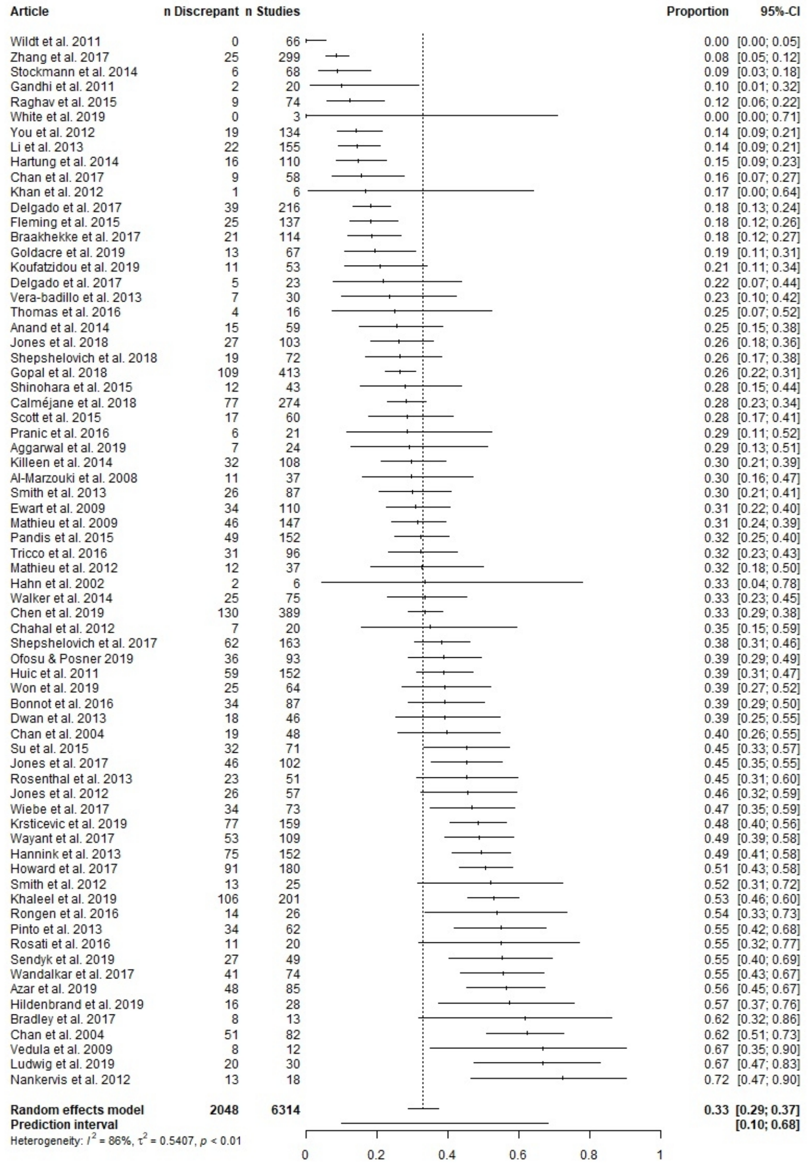

Figure 2. Forest plot of articles reporting the proportion of assessed studies with at least one primary outcome discrepancy.

141x202mm (144 x 144 DPI)

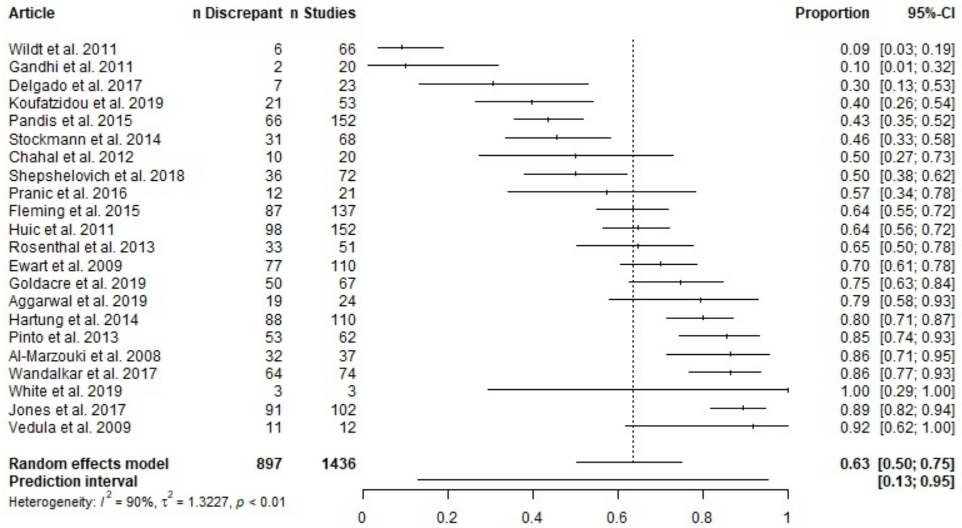

Figure 3. Forest plot of articles reporting the proportion of assessed studies with at least one secondary outcome discrepancy.

141x84mm (144 x 144 DPI)

Supplementary materials

A. Protocol amendments

As our systematic review was largely exploratory, many elements of the protocol are detailed more precisely in the manuscript than they were in our preregistered study protocol. We made the following amendments to this protocol:

- We included additional contributors and some of the projected contributor roles changed.
- Before beginning data extraction, we operationalized Table 2 from the protocol into a Qualtrics survey form and made the items more precise and more detailed.
- The protocol uses the term "*preregistration-publication discrepancies*". However, because many articles were unclear whether the registrations were prospective, we included data on discrepancies between publications and registrations regardless of the timing of registration. We also include discrepancies between publications and protocols, ethics applications, grant applications, and marketing applications. We did not foresee articles being published on discrepancies between these documents, but believe them to be relevant to our systematic review.
- Whereas the protocol states that the review will be descriptive, we used inferential statistics to run meta-analyses and to test for parameters that might influence the prevalence of discrepancies (i.e., the analyses presented in Table 2). We had preregistered that we would search for "*Additional inferential statistics relevant to preregistration–publication discrepancies*", but did not preregister that we would run our own inferential analyses. We used inferential statistics because we found many more articles than we anticipated and we developed our data extraction form to have more precision than we originally intended.
- The scoping review was terminated, as explained in the updated version or the registration associated with this study.
- When screening, for that sake of simplicity, we did not end up using the bin "*(iv) relevant to preregistration but not directly relevant to our study objectives.*"
- A peer reviewer suggested we run a meta-regression which includes all the predictors. We ran this meta-regression after knowing the results of all the meta-regressions run with single predictors. We report on this in Supplementary Material E.

For peer review only - http://bmjopen.bmj.com/site/about/guidelines.xhtml

B. Coding

The coding form is divided into five sections and available at osf.io/728ys. Section One coded the search strategy and design of each article, including how the researchers identified studies (e.g., through registries or journals), first and last year of publication of the assessed studies, whether the article was solely observational, the research discipline assessed (as defined by the Scopus discipline categories outlined in Appendix C of our preregistered protocol), and a verbatim copy of the main conclusion that was relevant to findings on discrepancies.

Section Two coded when the assessed studies were registered (e.g., prospectively, retrospectively, unclear), the version of the registration used when checking for discrepancies (e.g., original version, most recent version), and whether the article checked if authors disclosed reasons for any of the discrepancies.

Section Three coded outcome discrepancies. Whereas some articles made it clear that they only assessed studies that were prospectively registered, others were unclear about when the studies they assessed were registered. We coded discrepancies for unambiguously preregistered studies separate from studies with unclear registration timing or retrospective registration. If an article reported discrepancies separately for prospectively and retrospectively registered studies, we coded both these categories. We divided outcome discrepancies into 10 non-exclusive subcategories: any outcome discrepancy, a primary outcome discrepancy, a secondary outcome discrepancy, demoting a primary outcome to a secondary outcome, omitting a primary outcome, adding a primary outcome, promoting a secondary outcome to a primary outcome, omitting a secondary outcome, adding a secondary outcome, and changing the timing at which an outcome was measured. We only coded *any outcome discrepancy* for articles that checked for both primary and secondary outcome discrepancies in the studies they assessed. If we had also included articles that only checked for primary outcome discrepancies in this sub-category, then we would have biased our estimate downwards because the assessed studies may also have secondary outcome discrepancies, but that information would not be documented. Articles used slightly different methods for identifying and defining primary outcome discrepancies. Rather than use a stringent definition of what constitutes a primary outcome discrepancy across all articles, we coded the number of studies with primary outcome discrepancies based on the data presented in the results section of each article, regardless of the method or definition the article used. A definition used in many articles comes from Chan, Hróbjartsson, et al. (2004) and includes (1) demoting a primary outcome to a secondary outcome, (2) omitting a primary outcome, (3) adding a primary outcome, (4) promoting a secondary outcome to a primary outcome, and (5) changing the timing at which a primary outcome is measured.

Section Four coded non-outcome discrepancies. These included discrepancies in participant eligibility (e.g., inclusions and exclusion criteria), sample size, randomization (e.g., number of experimental arms), method of blinding (e.g., single versus double blind), interventions (e.g., a change in the drug administered), study duration, analyses, subgroup analyses, funding, and reported results. There was heterogeneity between articles in their method for identifying and defining each type of discrepancy. As we did for the outcome discrepancies, we coded the number of studies with each type of discrepancies based on the data presented in the results section of each article, regardless of the method or definition the article used.

Section Five noted whether articles provided any additional descriptive or inferential statistics on discrepancies. In particular, it noted whether discrepancies were related to funding source, statistical significance, or the timing of registration.

For each type of discrepancy, we coded the numbers of publications containing at least one discrepancy of that type, rather than coding the total number of discrepancies of each type per publication (e.g., a single publication may have five discrepancies in secondary outcomes). Of the k = 89 articles we reviewed, 17 reported a measure of the total number of discrepancies. Sixteen of these 17 articles also reported the number of publications with at least one discrepancy, and as such we were able to include them in our analysis. We excluded the one remaining article that did not report the number of publications with at least one discrepancy. We do not analyze the total number of discrepancies in the present report.

After completing the data extraction and analyses described above, we extracted additional information from articles that we had identified as providing information on whether discrepancies were disclosed, as well as

For peer review only - http://bmjopen.bmj.com/site/about/guidelines.xhtml

whether discrepancies were related to funding source, statistical significance, or the timing of registration. We extracted this information directly into a spreadsheet, we did not create an additional coding form. We divided studies into industry funded or non-industry funded (which included public, private, and no funding). We excluded studies from this analysis if they did not report their funding or were funded by both industry and non-industry sources. We also coded the proportion of discrepancies that favored statistical significance. Favoring statistical significance included when a novel significant outcome was introduced, or a non-significant primary outcome was downgraded to a secondary outcome. It is impossible to know whether omitted outcomes were statistically significant or not. If we were to assume that the majority of omitted outcomes were non-significant, then this analysis would provide an underestimation of the proportion of studies that favor statistical significance.

For peer review only

For peer review only - http://bmjopen.bmj.com/site/about/guidelines.xhtml

C. Article characteristics

We identified and reviewed k = 89 articles that report at least one type of discrepancy. The vast majority of these articles assessed medical research (k = 81). The remaining articles focused on dentistry (k = 3), psychology (k = 3), physical therapy (k = 1), and economics (k = 1).

Articles compared publications to registrations deposited in conventional registries such as clinicaltrials.gov (k = 73), or to protocols submitted for research ethics review (k = 7), funding applications (k = 2), marketing approval (k = 2), or other purposes (k = 5). The 89 articles we reviewed identified studies through different sources, including journals (k = 33), registries (k = 27), search engines (k = 19), research ethics boards (k = 7), funders (k = 3), regulatory agencies (k = 2), and research groups (k = 2). Four articles used multiple sources. Seventy-one articles reported the year the earliest study they included was published (median = 2009, IQR: 2004-2012), and 80 reported the last year (median = 2013, IQR: 2010-2015).

All but 10 articles were solely observational. Three of these 10 articles emailed authors for registration numbers (Ross et al., 2019; Shinohara et al., 2015; Wiebe et al., 2017) and one emailed authors for full protocols (Saquib et al., 2013). Four articles sent a survey to researchers to ask about the discrepancies (Chan, Hróbjartsson, et al., 2004; Chan, Krleža-Jerić, et al., 2004; Redmond et al., 2013; Wayant et al., 2017). One article sent a survey to better understand user experience with pre-analysis plans (Ofosu & Posner, 2019). Only one article attempted to correct published discrepancies. They sent letters to the editor within weeks of a study being published with outcome discrepancies (Goldacre et al., 2019). One currently ongoing study (not included in our systematic review) encourages peer reviewers to check for selective outcome reporting of primary outcomes (C. W. Jones et al., 2019). We do not further discuss the non-observational aspects of these studies in this review.

Whereas k = 29 articles clearly state that they compared the publications to the original registry entry or protocol, 15 used the most recent version of the registry entry, 35 do not mention which registry version they used, and 10 used a different version of the registration or were unclear about the version they used.

For peer review only

For peer review only - http://bmjopen.bmj.com/site/about/guidelines.xhtml

D. Meta-analyses of each outcome and non-outcome discrepancy separated by timing of registration

Here, we performed two additional sets of random effects meta-analyses: (1) for articles that reported on unambiguously prospectively registered studies (i.e., studies that were clearly prospectively registered), (2) for articles that reported on studies where the timing of registration was unclear.

While some of the outcome discrepancies appear to occur more frequently in studies that were unambiguously identified as prospectively registered, as compared to those with unclear timing of registration, the confidence intervals are largely overlapping (Supplementary Table D1). And yet, we cannot ascertain that prospectively and retrospectively registered studies have a similar frequency of discrepancies because the unclear category consists of both retrospectively and prospectively registered studies in an unknown proportion. As for non-outcome discrepancies, we deemed it unreasonable to compare unambiguously prospectively registered studies to studies with unclear timing of registration. There were too few studies and their methods were highly heterogeneous.

Supplementary Table D1. Meta-analytic estimates for the proportion of studies that contain various types of outcome discrepancies and non-outcome discrepancies. Articles are separated into those which unambiguously assessed only prospectively registered studies versus articles where the registration timing of the assessed studies was retrospective, unclear, or mixed.

	Prospective registration				Unclear registration timing			
	95% CI	95% PI	k	n	95% CI	95% PI	k	n
Any outcome discrepancy	38-91%	5-99%	6	279	32-70%	6-95%	13	850
Any primary outcome discrepancy	25-38%	11-64%	24	1767	28-39%	9-72%	51	4857
Any secondary outcome discrepancy	46-87%	12-98%	7	506	44-74%	11-95%	16	1052
Primary outcome demoted to secondary outcome	7-18%	2-44%	15	1287	5-9%	2-27%	36	3273
Primary outcome omitted	6-16%	1-56%	20	1600	6-11%	1-36%	32	2860
Primary outcome added	7-14%	3-30%	18	1403	6-12%	1-37%	36	3294
Secondary outcome promoted to primary outcome	3-9%	1-25%	14	1250	3-6%	1-18%	32	2885
Secondary outcome omitted	14-62%	2-93%	7	566	14-31%	5-58%	12	799
Secondary outcome added	8-80%	0.3-99%	6	413	18-41%	5-73%	14	892
Timing of outcome measurement changed	3-18%	1-55%	9	864	6-19%	1-68%	21	2608
Eligibility criteria	1-99%	23-67%	2	50	23-59%	4-92%	13	1103
Sample size	40-59%	39-60%	6	174	22-43%	6-78%	20	1244
Randomization	46-70%	46-70%	1	58	1-56%	0.02-98%	4	118
Blinding	NA	NA	NA	NA	5-42%	0.04-99%	3	224
Intervention	1-22%	1-22%	1	27	3-63%	0.1-98%	6	523
Study duration	NA	NA	NA	NA	3-89%	0.02-99.94%	4	184
Analysis	24-71%	6-92%	6	232	7-46%	2-80%	6	172
Subgroup analysis	7-99%	0.01-99.998%	4	215	20-97%	1-99.93%	5	330
Funding	7-67%	7-67%	1	7	4-93%	0.01-99.98%	4	205
Results	4-20%	4-20%	1	54	7-91%	0.1-99.8%	5	208

Values less than 0.5% and greater than 99.5% are rounded to one significant digit from 0 or 100. Prediction intervals cannot be calculated for meta-analyses with fewer than three studies.

For peer review only - http://bmjopen.bmj.com/site/about/guidelines.xhtml

The 38 forest plots that follow align with the meta-analyses presented in Supplementary Table D1.

For peer review only

For peer review only - http://bmjopen.bmj.com/site/about/guidelines.xhtml

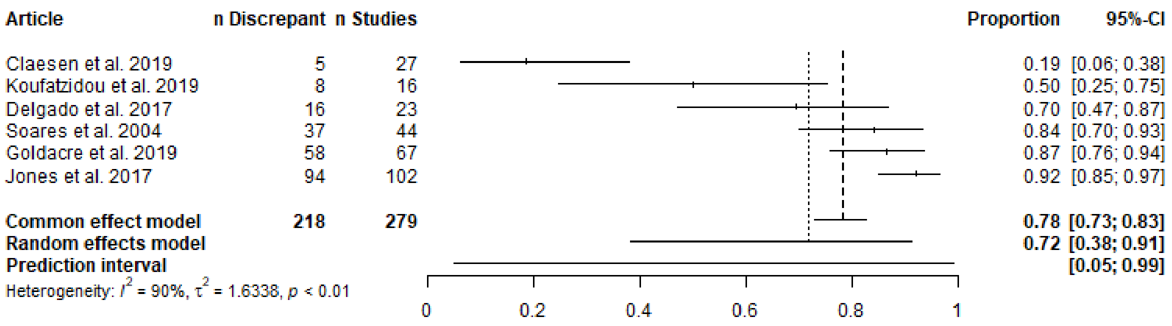

Supplementary Figure D1. Any outcome discrepancy, prospective.

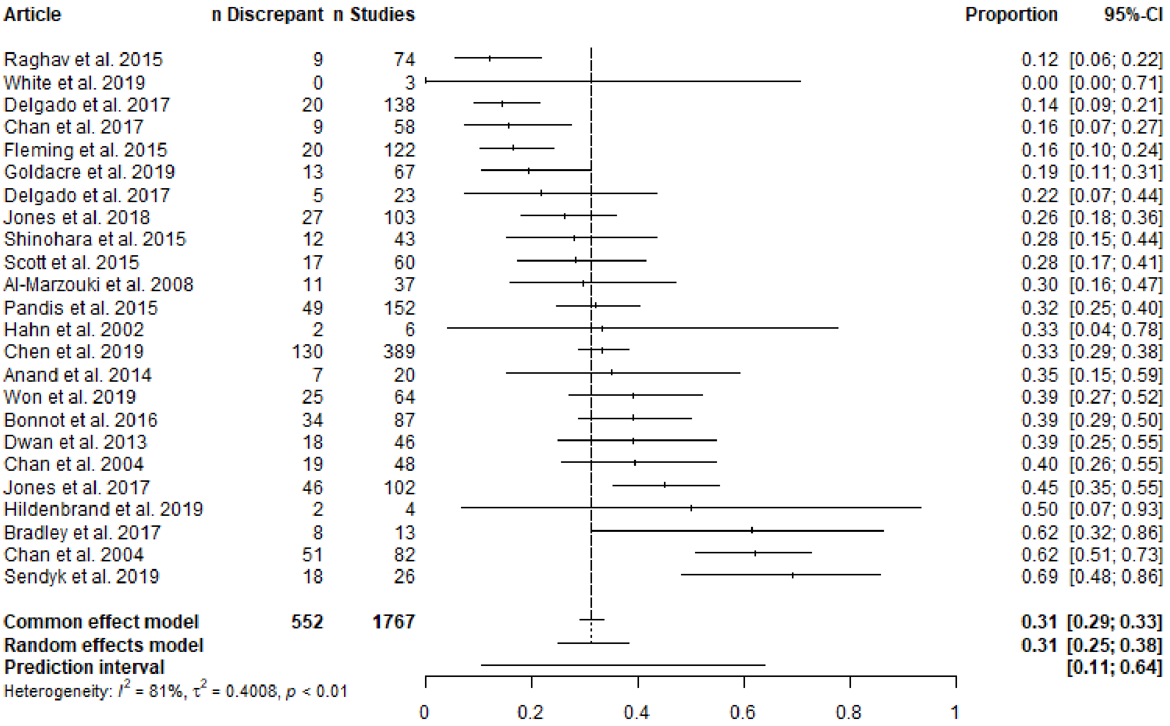

Supplementary Figure D2. Any primary outcome discrepancy, prospective.

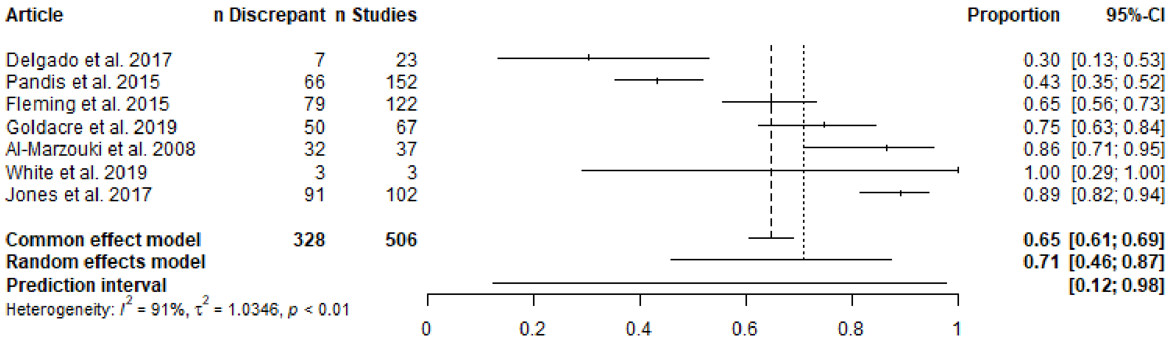

Supplementary Figure D3. Any secondary outcome discrepancy, prospective.

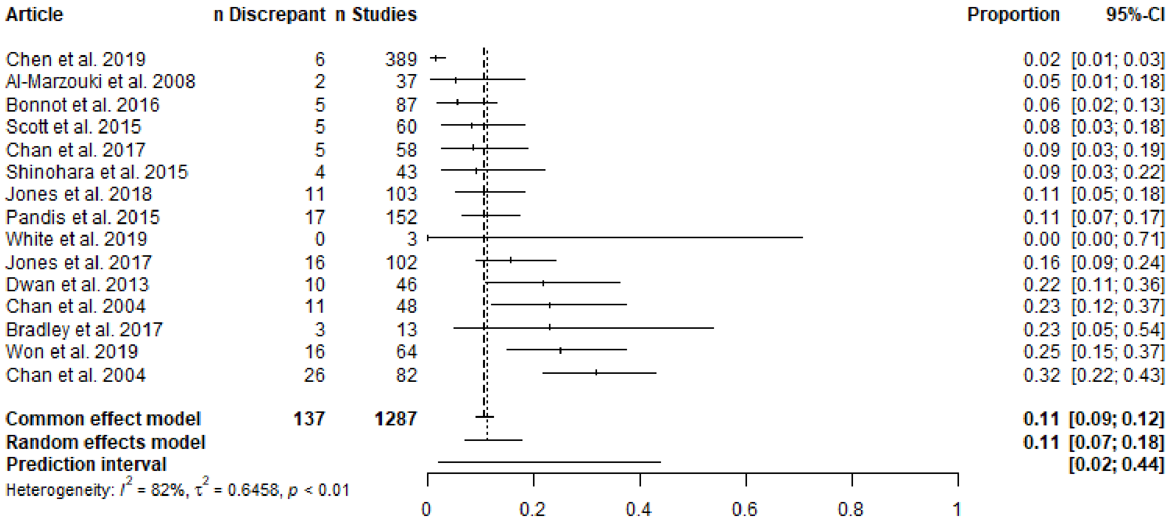

Supplementary Figure D4. Primary outcome demoted to secondary outcome, prospective.

Article	n Discrepant	n Studies	Proportion	95%-CI
Raghav et al. 2015	1	74	0.01	[0.00; 0.07]
Chan et al. 2017	1	58	0.02	[0.00; 0.09]
Bonnot et al. 2016	3	87	0.03	[0.01; 0.10]
Dwan et al. 2013	2	46	0.04	[0.01; 0.15]
Jones et al. 2018	5	103	0.05	[0.02; 0.11]
Fleming et al. 2015	8	122	0.07	[0.03; 0.13]
Goldacre et al. 2019	5	67	0.07	[0.02; 0.17]
Shinohara et al. 2015	4	43	0.09	[0.03; 0.22]
Won et al. 2019	6	64	0.09	[0.04; 0.19]
Pandis et al. 2015	15	152	0.10	[0.06; 0.16]
Jones et al. 2017	11	102	0.11	[0.06; 0.18]
Chan et al. 2004	6	48	0.12	[0.05; 0.25]
White et al. 2019	0	3	0.00	[0.00; 0.71]
Al-Marzouki et al. 2008	5	37	0.14	[0.05; 0.29]
Scott et al. 2015	9	60	0.15	[0.07; 0.27]
Bradley et al. 2017	2	13	0.15	[0.02; 0.45]
Hahn et al. 2002	1	6	0.17	[0.00; 0.64]
Chen et al. 2019	66	389	0.17	[0.13; 0.21]
Chan et al. 2004	20	82	0.24	[0.16; 0.35]
Soares et al. 2004	37	44	0.84	[0.70; 0.93]
**Common effect model**	**207**	**1600**	**0.13**	**[0.11; 0.15]**
**Random effects model**			**0.10**	**[0.06; 0.16]**
**Prediction interval**				**[0.01; 0.56]**

Heterogeneity: $I^2 = 84\%$, $\tau^2 = 1.2863$, $p < 0.01$

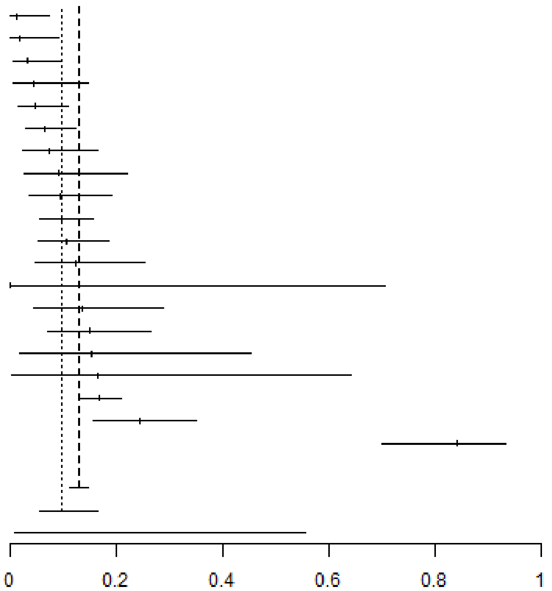

Supplementary Figure D5. Primary outcome omitted, prospective.

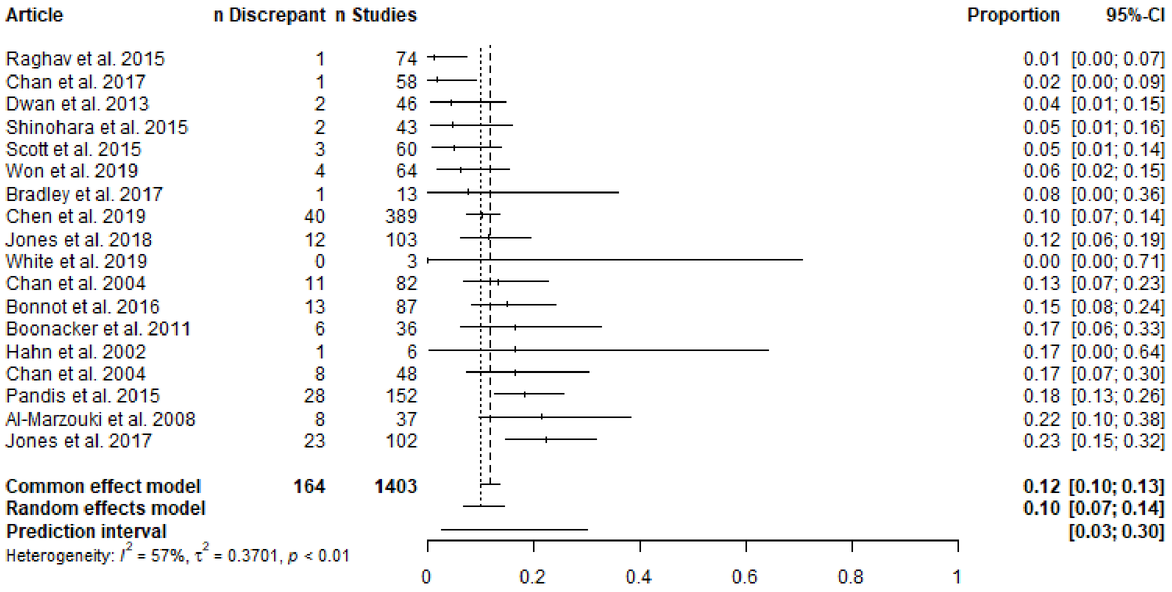

Supplementary Figure D6. Primary outcome added, prospective.

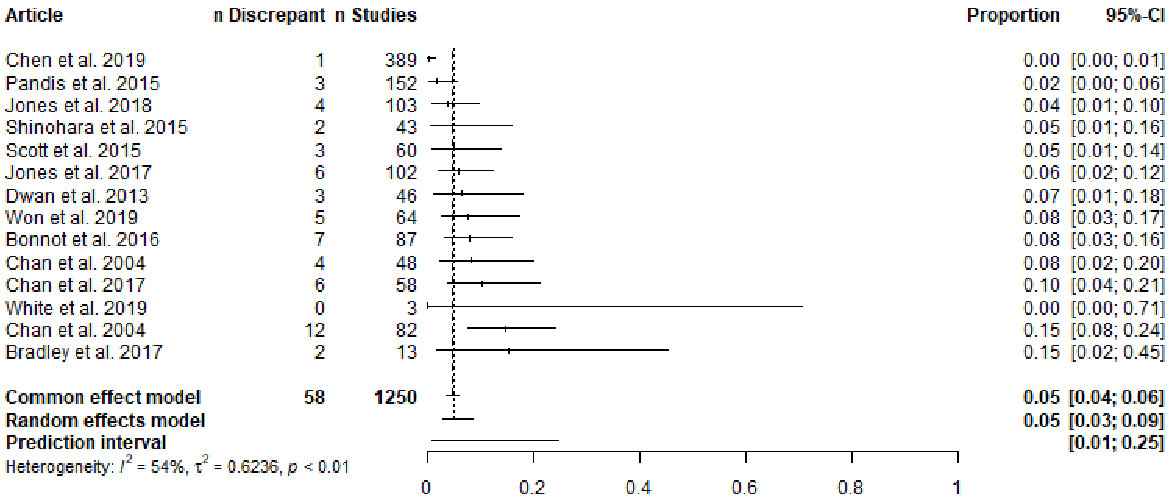

Supplementary Figure D7. Secondary outcome promoted to primary outcome, prospective.

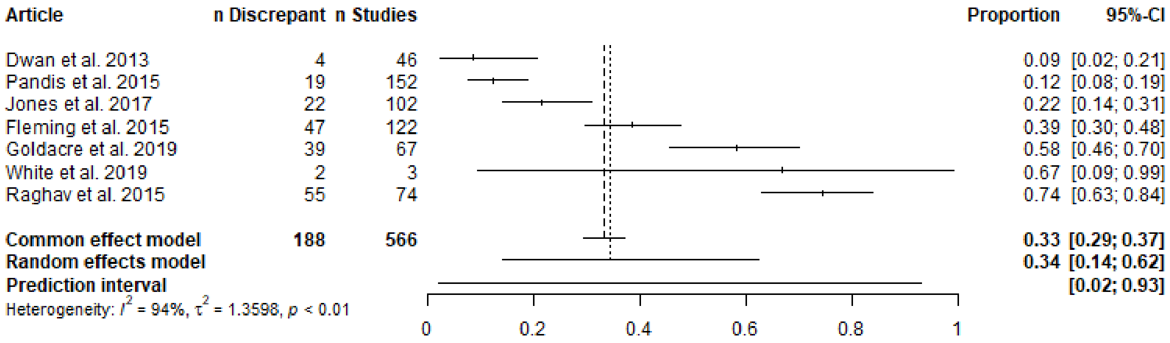

Supplementary Figure D8. Secondary outcome omitted, prospective.

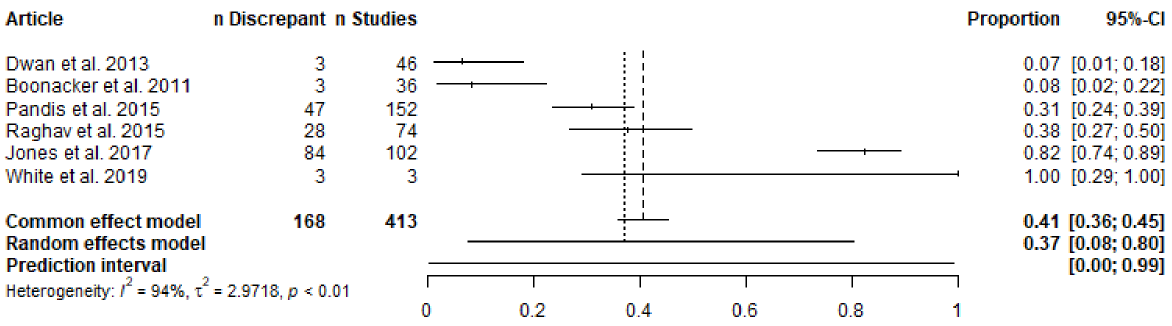

Supplementary Figure D9. Secondary outcome added, prospective.

For peer review only - http://bmjopen.bmj.com/site/about/guidelines.xhtml

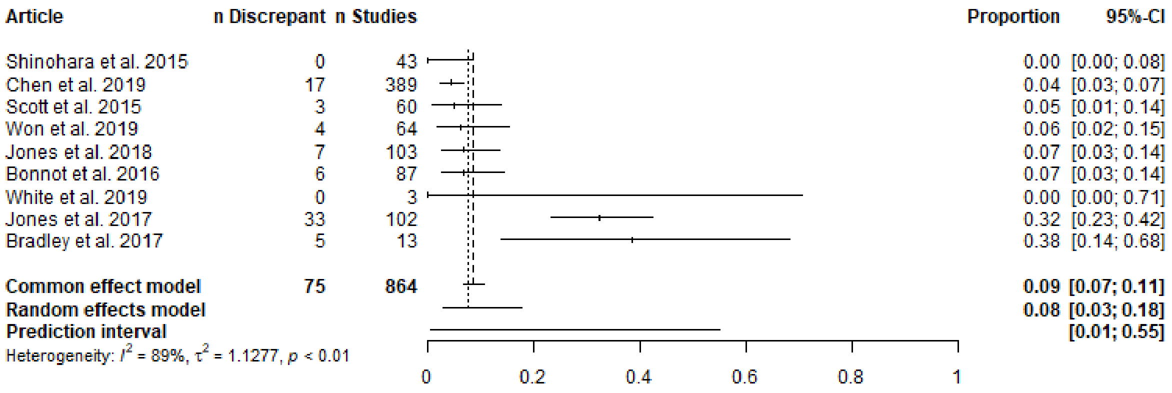

Supplementary Figure D10. Timing of outcome measurement changed, prospective.

For peer review only - http://bmjopen.bmj.com/site/about/guidelines.xhtml

Article	n Discrepant	n Studies	Proportion	95%-CI
Rankin et al. 2017	22	142	0.15	[0.10; 0.23]
Braakhekke et al. 2017	21	114	0.18	[0.12; 0.27]
Gandhi et al. 2011	4	20	0.20	[0.06; 0.44]
Korevaar et al. 2014	32	131	0.24	[0.17; 0.33]
Ross et al. 2019	22	70	0.31	[0.21; 0.44]
Riehm et al. 2015	1	3	0.33	[0.01; 0.91]
Koufatzidou et al. 2019	25	53	0.47	[0.33; 0.61]
Shepshelovich et al. 2017	118	163	0.72	[0.65; 0.79]
Norris et al. 2014	27	36	0.75	[0.58; 0.88]
Aggarwal et al. 2019	19	24	0.79	[0.58; 0.93]
Sendyk et al. 2019	39	49	0.80	[0.66; 0.90]
Chahal et al. 2012	16	20	0.80	[0.56; 0.94]
Smith et al. 2012	22	25	0.88	[0.69; 0.97]
**Common effect model**	**368**	**850**	**0.43**	**[0.40; 0.47]**
**Random effects model**			**0.52**	**[0.32; 0.70]**
**Prediction interval**				**[0.06; 0.95]**

Heterogeneity: $I^2 = 94\%$, $\tau^2 = 1.5464$, $p < 0.01$

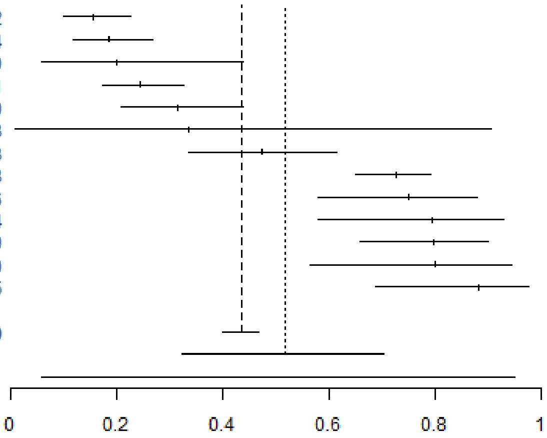

Supplementary Figure D11. Any outcome discrepancy, unclear registration timing.

For peer review only - http://bmjopen.bmj.com/site/about/guidelines.xhtml

Article	n Discrepant	n Studies	Proportion	95%-CI
Wildt et al. 2011	0	66	0.00	[0.00; 0.05]
Zhang et al. 2017	25	299	0.08	[0.05; 0.12]
Stockmann et al. 2014	6	68	0.09	[0.03; 0.18]
Gandhi et al. 2011	2	20	0.10	[0.01; 0.32]
You et al. 2012	19	134	0.14	[0.09; 0.21]
Li et al. 2013	22	155	0.14	[0.09; 0.21]
Hartung et al. 2014	16	110	0.15	[0.09; 0.23]
Khan et al. 2012	1	6	0.17	[0.00; 0.64]
Delgado et al. 2017	39	216	0.18	[0.13; 0.24]
Fleming et al. 2015	25	137	0.18	[0.12; 0.26]
Braakhekke et al. 2017	21	114	0.18	[0.12; 0.27]
Koufatzidou et al. 2019	11	53	0.21	[0.11; 0.34]
Vera-badillo et al. 2013	7	30	0.23	[0.10; 0.42]
Thomas et al. 2016	4	16	0.25	[0.07; 0.52]
Anand et al. 2014	15	59	0.25	[0.15; 0.38]
Shepshelovich et al. 2018	19	72	0.26	[0.17; 0.38]
Gopal et al. 2018	109	413	0.26	[0.22; 0.31]
Calméjane et al. 2018	77	274	0.28	[0.23; 0.34]
Pranić et al. 2016	6	21	0.29	[0.11; 0.52]
Aggarwal et al. 2019	7	24	0.29	[0.13; 0.51]
Killeen et al. 2014	32	108	0.30	[0.21; 0.39]
Smith et al. 2013	26	87	0.30	[0.21; 0.41]
Ewart et al. 2009	34	110	0.31	[0.22; 0.40]
Mathieu et al. 2009	46	147	0.31	[0.24; 0.39]
Tricco et al. 2016	31	96	0.32	[0.23; 0.43]
Mathieu et al. 2012	12	37	0.32	[0.18; 0.50]
Walker et al. 2014	25	75	0.33	[0.23; 0.45]
Chahal et al. 2012	7	20	0.35	[0.15; 0.59]
Shepshelovich et al. 2017	62	163	0.38	[0.31; 0.46]
Ofosu & Posner 2019	36	93	0.39	[0.29; 0.49]
Huić et al. 2011	59	152	0.39	[0.31; 0.47]
Su et al. 2015	32	71	0.45	[0.33; 0.57]
Rosenthal et al. 2013	23	51	0.45	[0.31; 0.60]
Jones et al. 2012	26	57	0.46	[0.32; 0.59]
Wiebe et al. 2017	34	73	0.47	[0.35; 0.59]
Krsticevic et al. 2019	77	159	0.48	[0.40; 0.56]
Wayant et al. 2017	53	109	0.49	[0.39; 0.58]
Hannink et al. 2013	75	152	0.49	[0.41; 0.58]
Howard et al. 2017	91	180	0.51	[0.43; 0.58]
Smith et al. 2012	13	25	0.52	[0.31; 0.72]
Khaleel et al. 2019	106	201	0.53	[0.46; 0.60]
Rongen et al. 2016	14	26	0.54	[0.33; 0.73]
Pinto et al. 2013	34	62	0.55	[0.42; 0.68]
Rosati et al. 2016	11	20	0.55	[0.32; 0.77]
Sendyk et al. 2019	27	49	0.55	[0.40; 0.69]
Wandalkar et al. 2017	41	74	0.55	[0.43; 0.67]
Azar et al. 2019	48	85	0.56	[0.45; 0.67]
Hildenbrand et al. 2019	16	28	0.57	[0.37; 0.76]
Vedula et al. 2009	8	12	0.67	[0.35; 0.90]
Ludwig et al. 2019	20	30	0.67	[0.47; 0.83]
Nankervis et al. 2012	13	18	0.72	[0.47; 0.90]
**Common effect model**	**1563**	**4857**	**0.32**	**[0.31; 0.34]**
**Random effects model**			**0.33**	**[0.28; 0.39]**
**Prediction interval**				**[0.09; 0.72]**

Heterogeneity: $I^2 = 88\%$, $\tau^2 = 0.6399$, $p < 0.01$

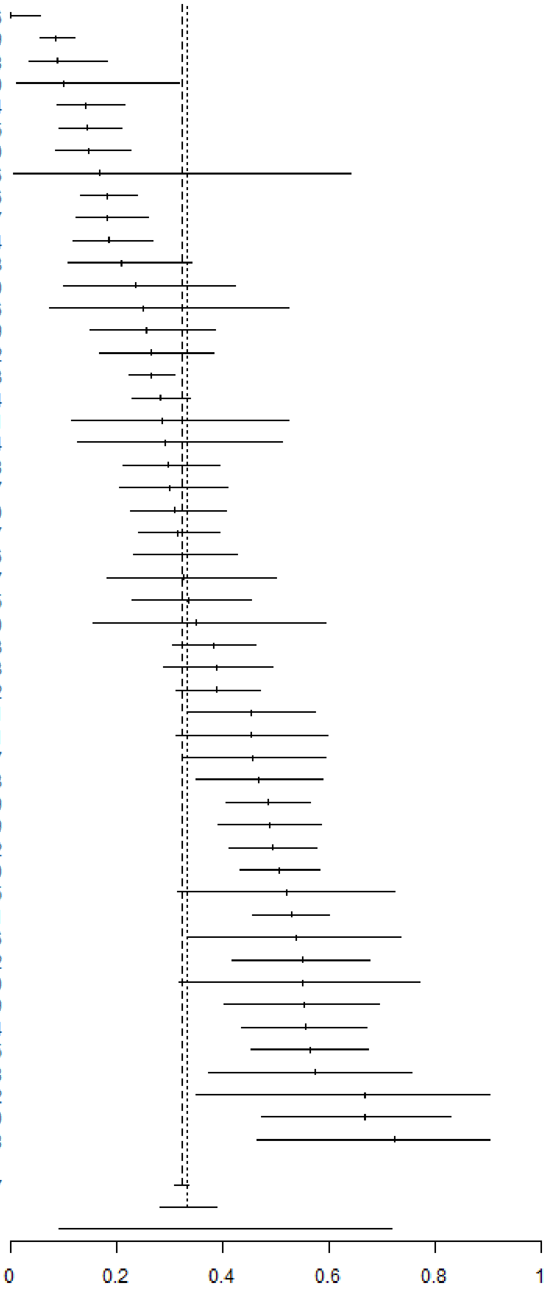

Supplementary Figure D12. Any primary outcome discrepancy, unclear registration timing.

For peer review only - http://bmjopen.bmj.com/site/about/guidelines.xhtml

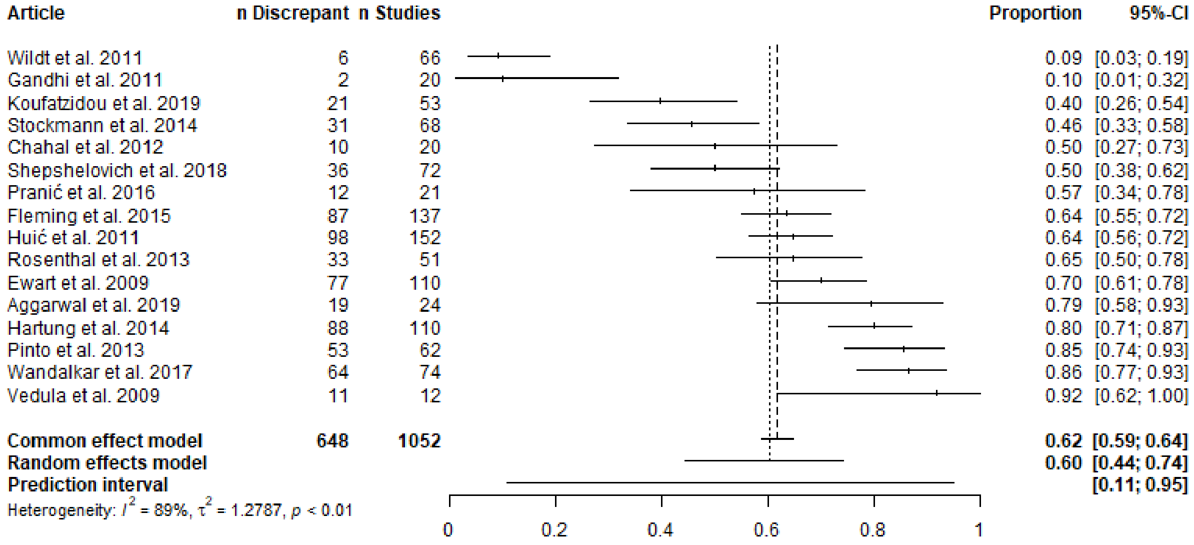

Supplementary Figure D13. Any secondary outcome discrepancy, unclear registration timing.

For peer review only - http://bmjopen.bmj.com/site/about/guidelines.xhtml

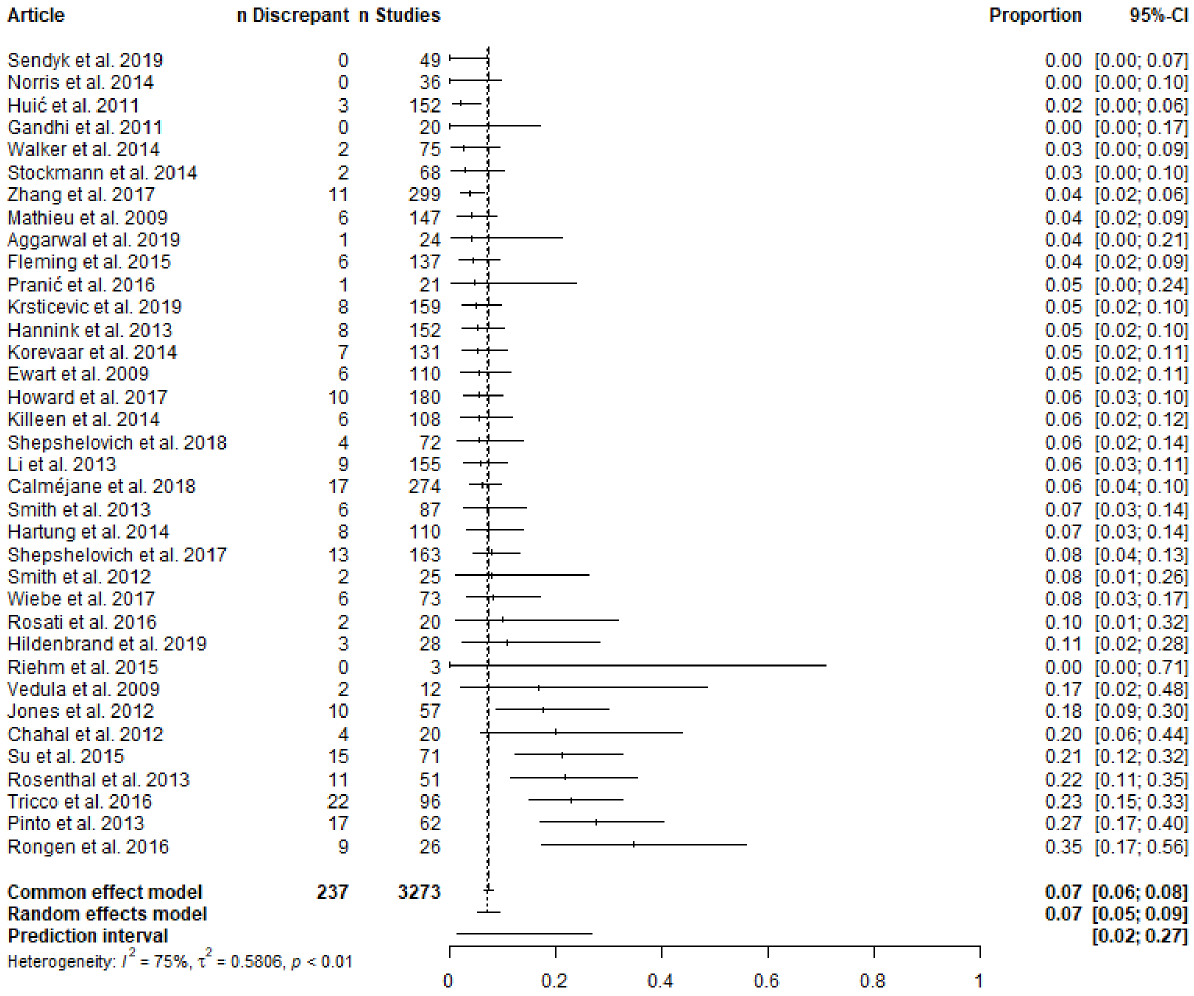

Supplementary Figure D14. Primary outcome demoted to secondary outcome, unclear registration timing.

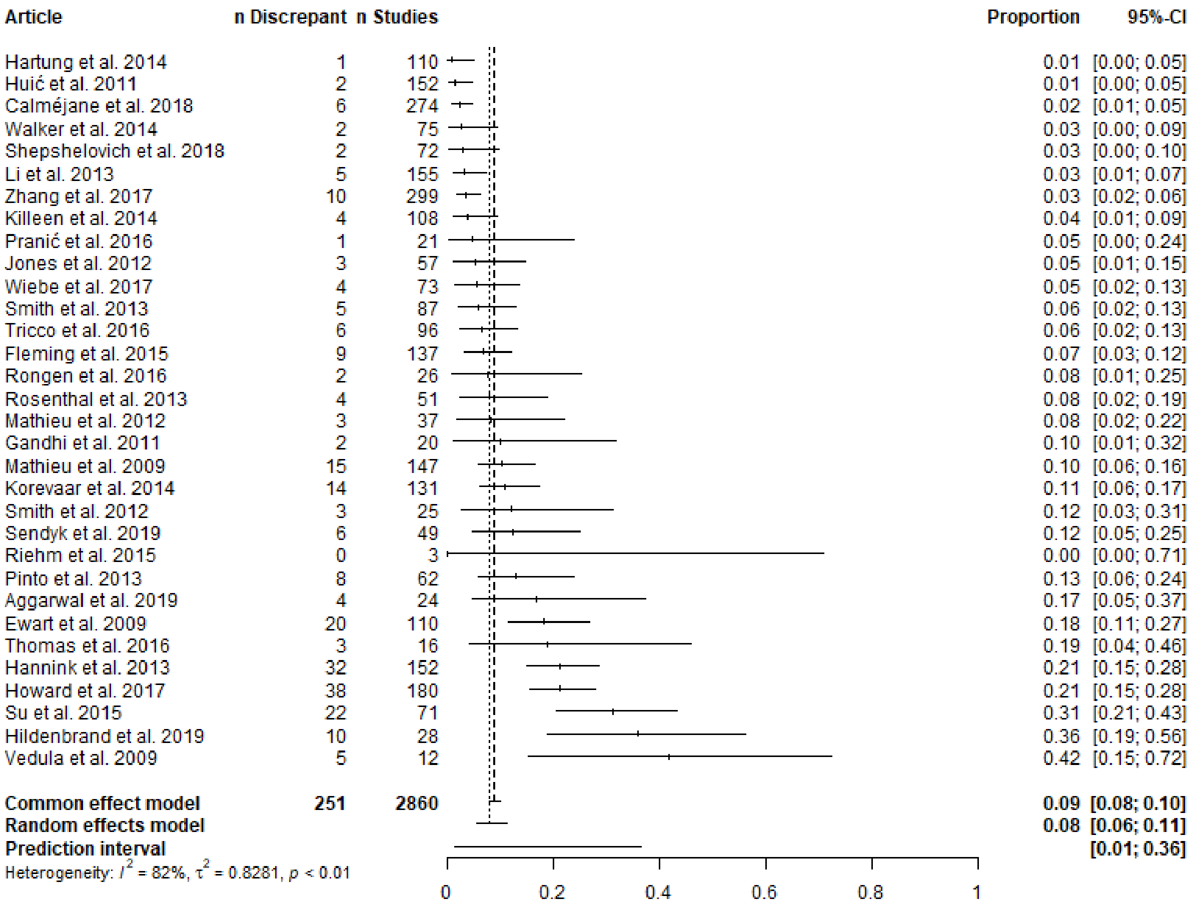

Supplementary Figure D15. Primary outcome omitted, unclear registration timing.

Article	n Discrepant	n Studies	Proportion	95%-CI
Norris et al. 2014	0	36	0.00	[0.00; 0.10]
Calméjane et al. 2018	5	274	0.02	[0.01; 0.04]
Huić et al. 2011	3	152	0.02	[0.00; 0.06]
Gandhi et al. 2011	0	20	0.00	[0.00; 0.17]
Zhang et al. 2017	8	299	0.03	[0.01; 0.05]
Killeen et al. 2014	4	108	0.04	[0.01; 0.09]
Wiebe et al. 2017	3	73	0.04	[0.01; 0.12]
Shepshelovich et al. 2018	3	72	0.04	[0.01; 0.12]
Fleming et al. 2015	6	137	0.04	[0.02; 0.09]
Stockmann et al. 2014	3	68	0.04	[0.01; 0.12]
Li et al. 2013	7	155	0.05	[0.02; 0.09]
Smith et al. 2013	4	87	0.05	[0.01; 0.11]
Pranić et al. 2016	1	21	0.05	[0.00; 0.24]
Korevaar et al. 2014	7	131	0.05	[0.02; 0.11]
Tricco et al. 2016	6	96	0.06	[0.02; 0.13]
Thomas et al. 2016	1	16	0.06	[0.00; 0.30]
Khaleel et al. 2019	13	201	0.06	[0.03; 0.11]
Aggarwal et al. 2019	2	24	0.08	[0.01; 0.27]
Ewart et al. 2009	10	110	0.09	[0.04; 0.16]
Rosenthal et al. 2013	5	51	0.10	[0.03; 0.21]
Riehm et al. 2015	0	3	0.00	[0.00; 0.71]
Su et al. 2015	9	71	0.13	[0.06; 0.23]
Walker et al. 2014	10	75	0.13	[0.07; 0.23]
Shepshelovich et al. 2017	22	163	0.13	[0.09; 0.20]
Mathieu et al. 2009	22	147	0.15	[0.10; 0.22]
Chahal et al. 2012	3	20	0.15	[0.03; 0.38]
Jones et al. 2012	9	57	0.16	[0.07; 0.28]
Hannink et al. 2013	24	152	0.16	[0.10; 0.23]
Sendyk et al. 2019	8	49	0.16	[0.07; 0.30]
Ofosu & Posner 2019	17	93	0.18	[0.11; 0.28]
Rongen et al. 2016	5	26	0.19	[0.07; 0.39]
Pinto et al. 2013	12	62	0.19	[0.10; 0.31]
Hildenbrand et al. 2019	8	28	0.29	[0.13; 0.49]
Smith et al. 2012	8	25	0.32	[0.15; 0.54]
Howard et al. 2017	61	180	0.34	[0.27; 0.41]
Vedula et al. 2009	6	12	0.50	[0.21; 0.79]
**Common effect model**	**315**	**3294**	**0.10**	**[0.09; 0.11]**
**Random effects model**			**0.09**	**[0.06; 0.12]**
**Prediction interval**				**[0.01; 0.37]**

Heterogeneity: $I^2 = 83\%$, $\tau^2 = 0.7961$, $p < 0.01$

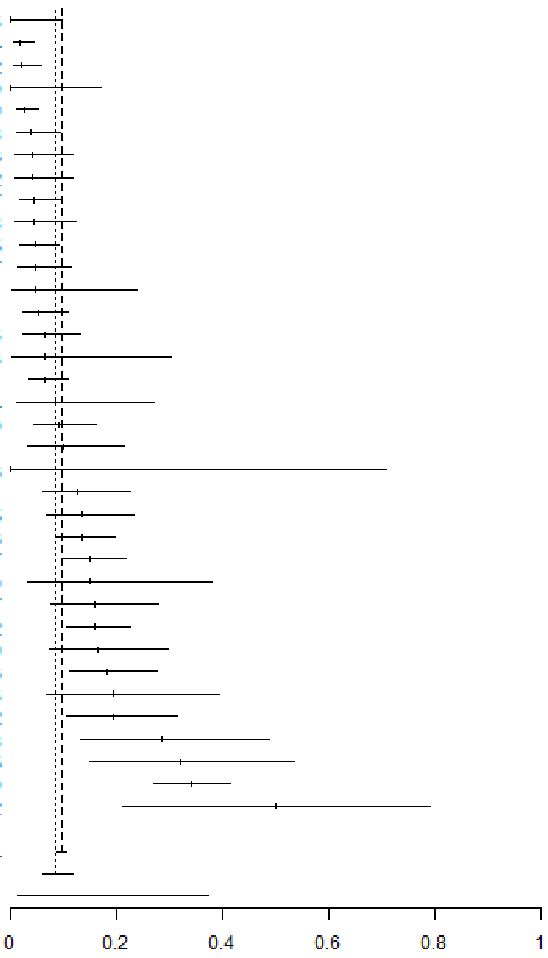

Supplementary Figure D16. Primary outcome added, unclear registration timing.

For peer review only - http://bmjopen.bmj.com/site/about/guidelines.xhtml

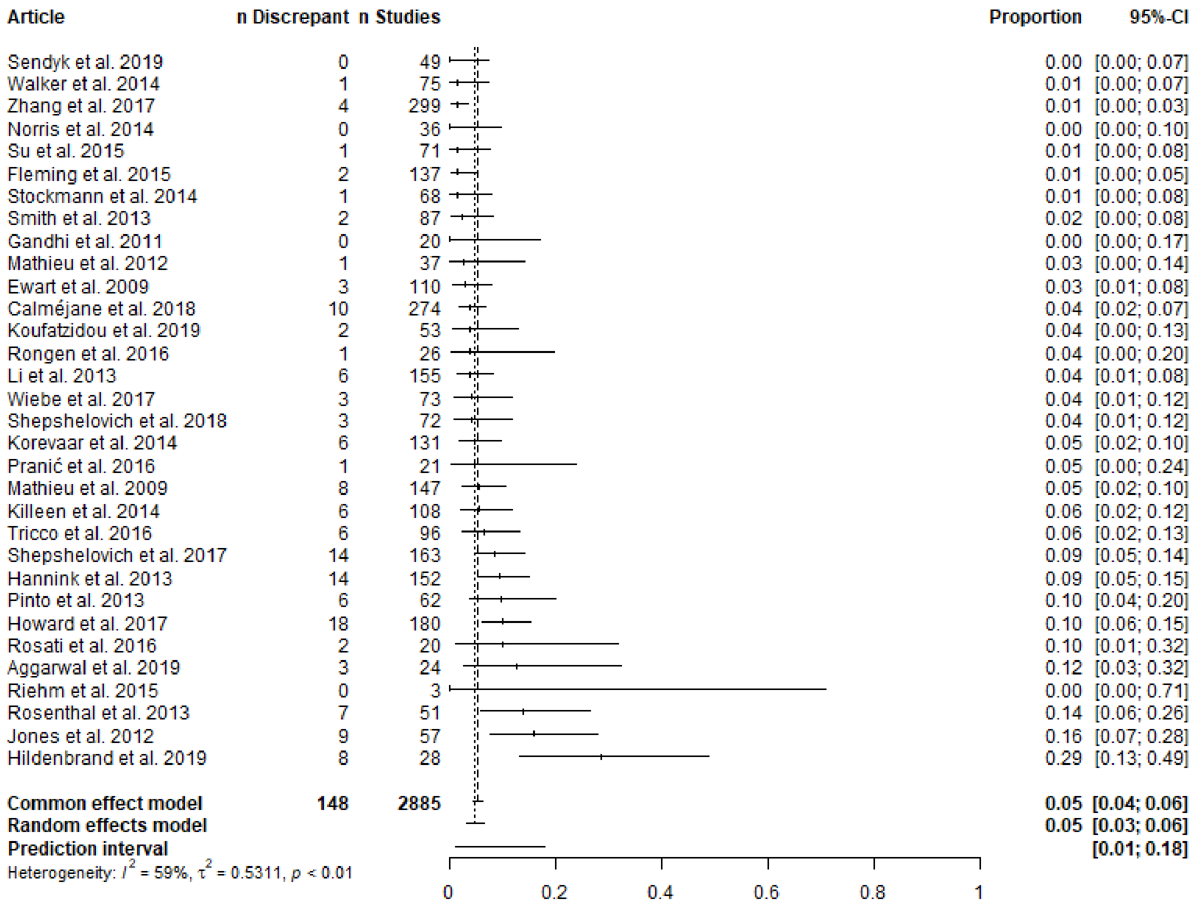

Supplementary Figure D17. Secondary outcome promoted to primary outcome, unclear registration timing.

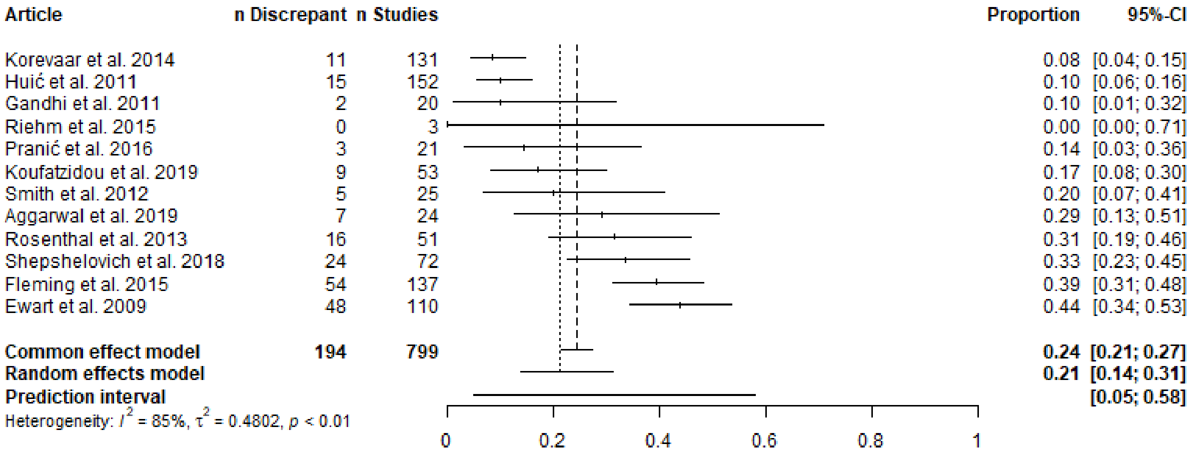

Supplementary Figure D17. Secondary outcome omitted, unclear registration timing.

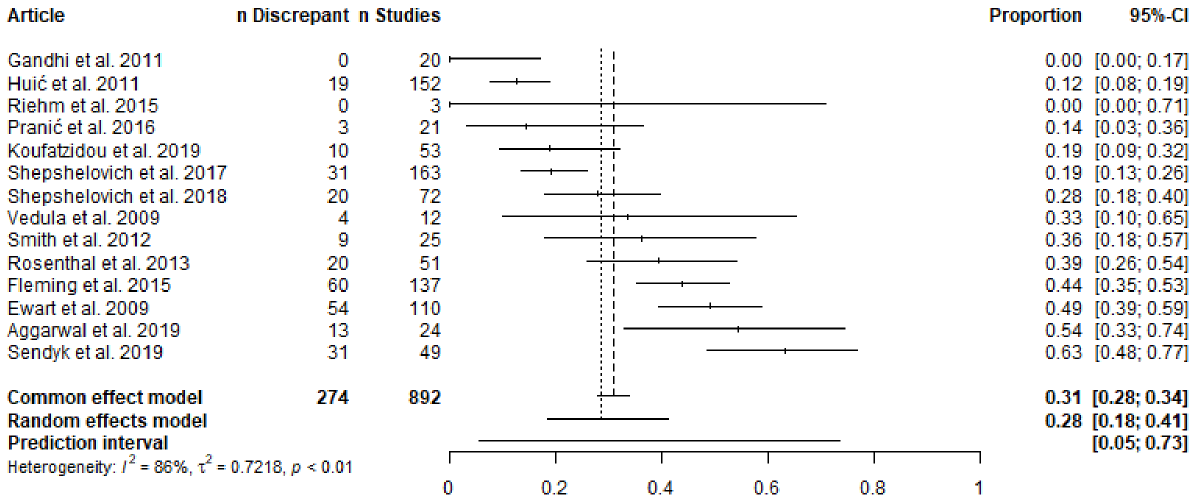

Supplementary Figure D19. Secondary outcome added, unclear registration timing.

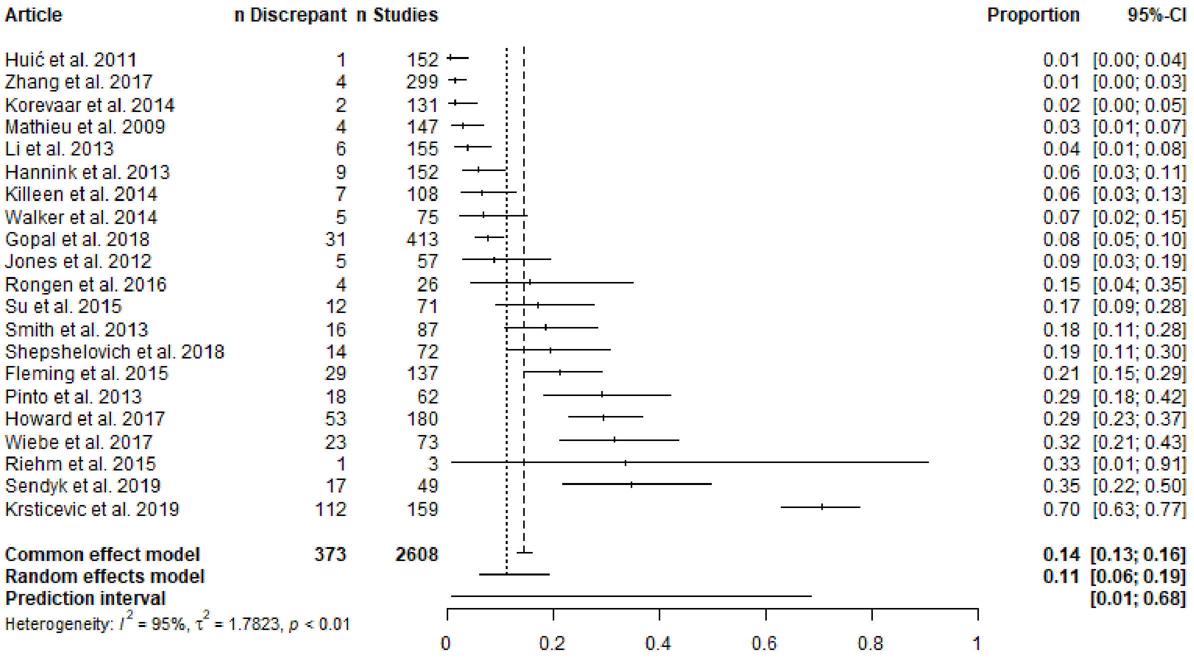

Supplementary Figure D20. Timing of outcome measurement changed, unclear registration timing.

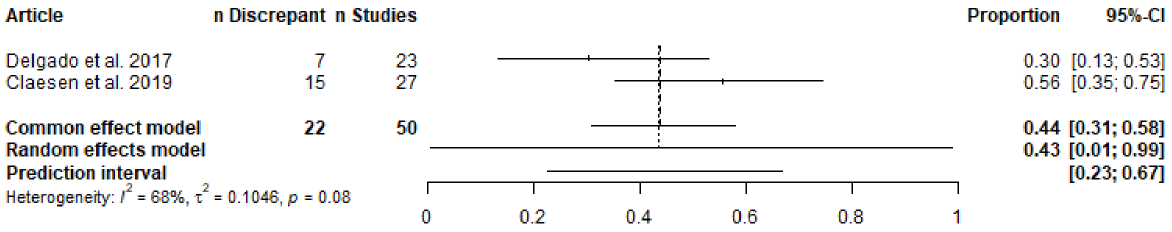

Supplementary Figure D21. Eligibility criteria, prospective.

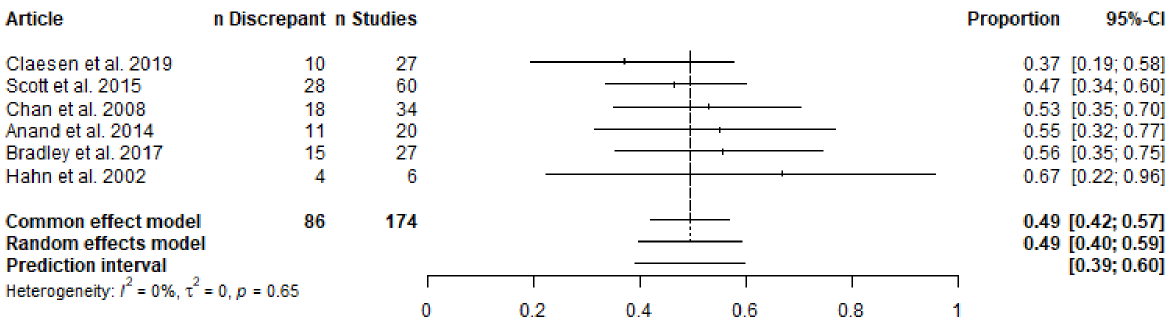

Supplementary Figure D22. Sample size, prospective.

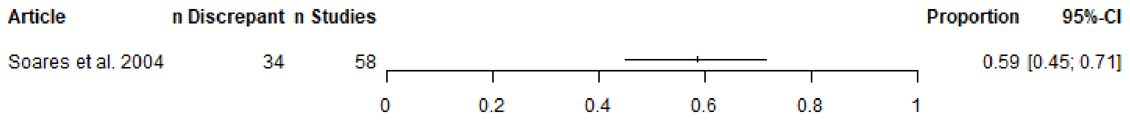

Supplementary Figure D23. Randomization, prospective.

For peer review only - http://bmjopen.bmj.com/site/about/guidelines.xhtml

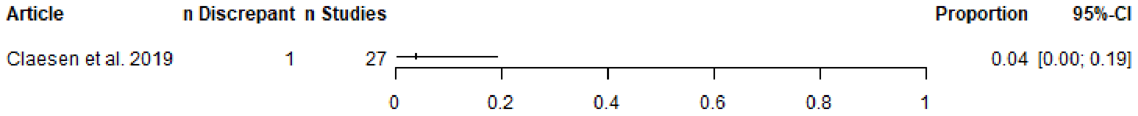

Supplementary Figure D24. Intervention, prospective.

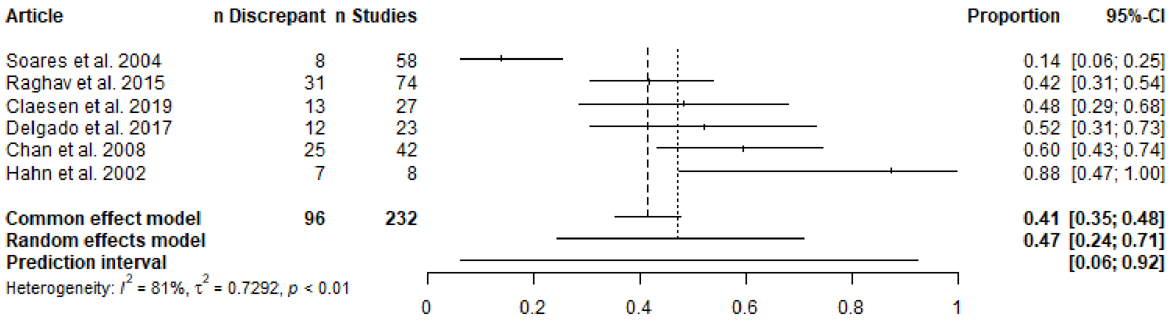

Supplementary Figure D25. Analyses, prospective.

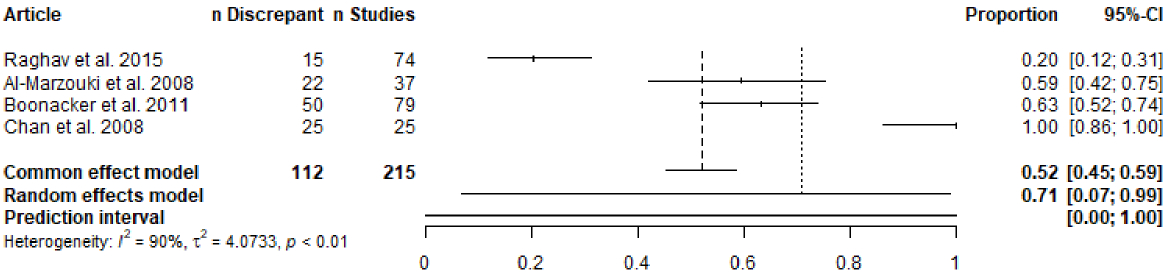

Supplementary Figure D26. Subgroup analyses, prospective.

For peer review only - http://bmjopen.bmj.com/site/about/guidelines.xhtml

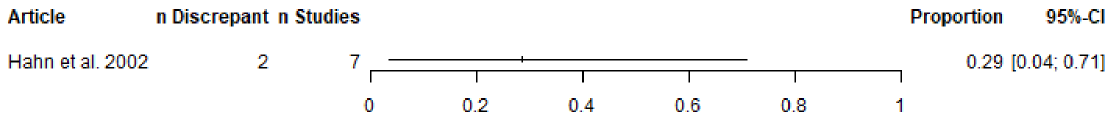

Supplementary Figure D27. Funding, prospective.

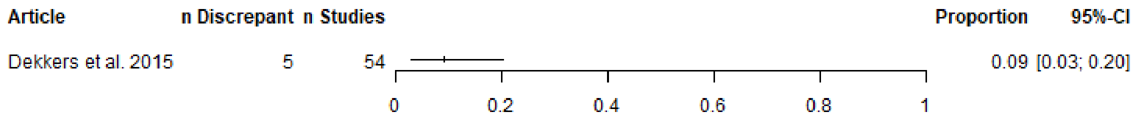

Supplementary Figure D28. Results, prospective.

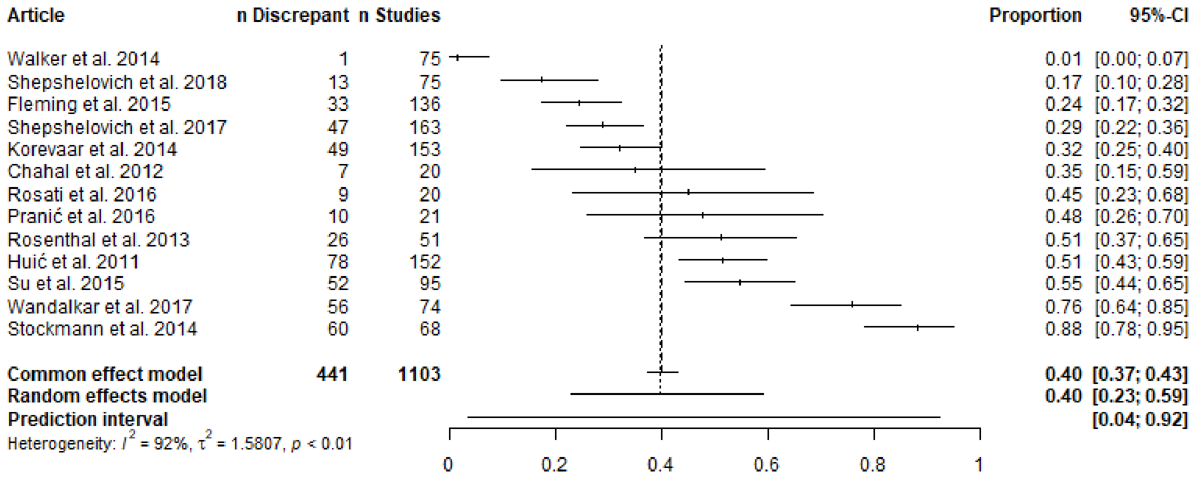

Supplementary Figure D29. Eligibility criteria, unclear registration timing.

For peer review only - http://bmjopen.bmj.com/site/about/guidelines.xhtml

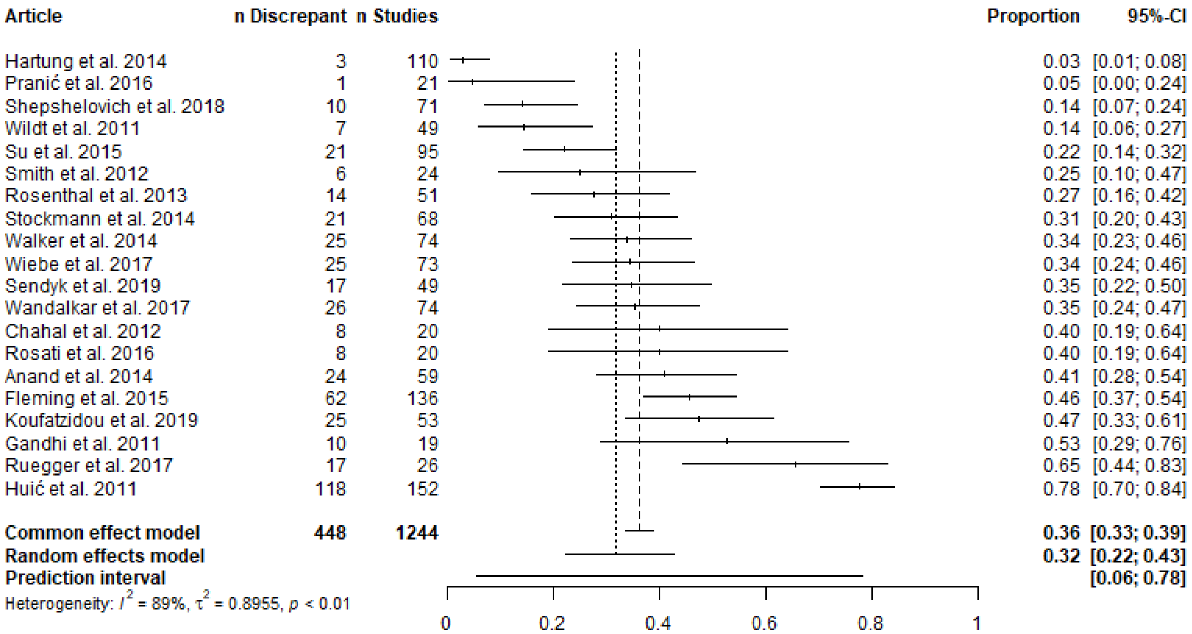

Supplementary Figure D30. Sample size, unclear registration timing.

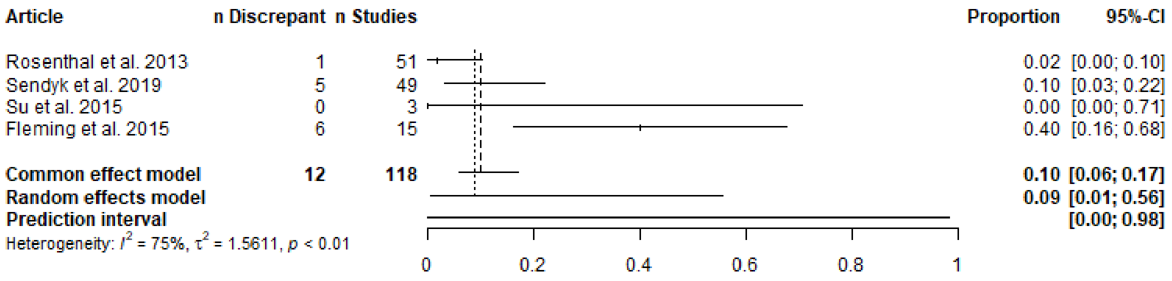

Supplementary Figure D31. Randomization, unclear registration timing.

For peer review only - http://bmjopen.bmj.com/site/about/guidelines.xhtml

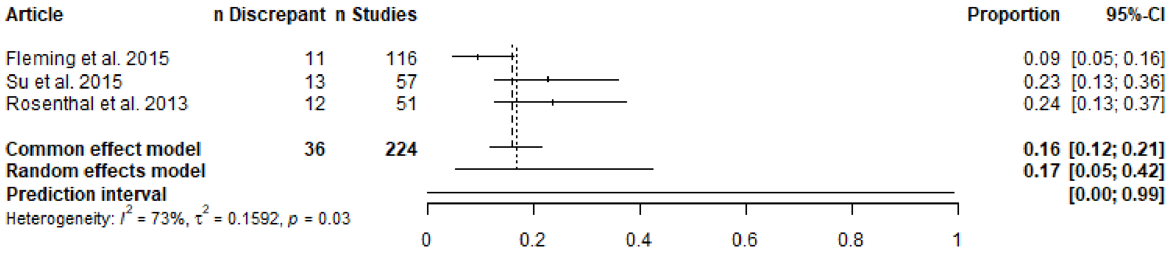

Supplementary Figure D32. Blinding, unclear registration timing.

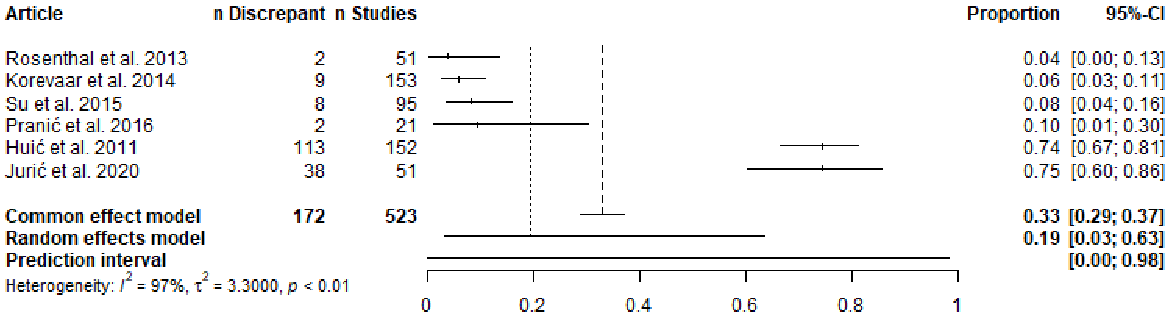

Supplementary Figure D33. Intervention, unclear registration timing.

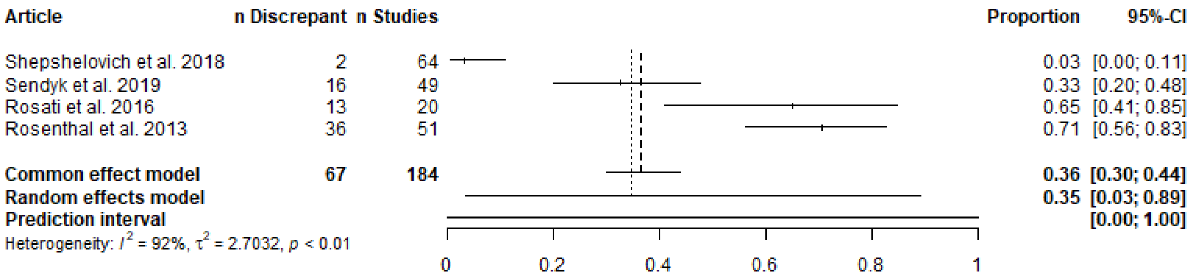

Supplementary Figure D34. Study duration, unclear registration timing.

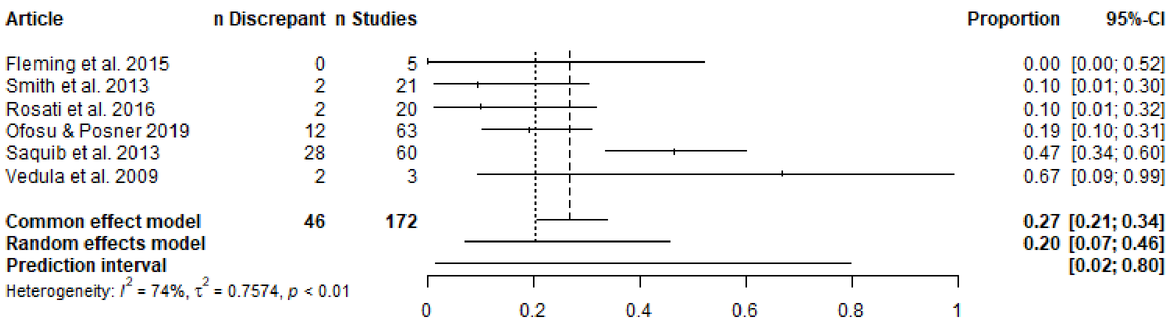

Supplementary Figure D35. Analyses, unclear registration timing.

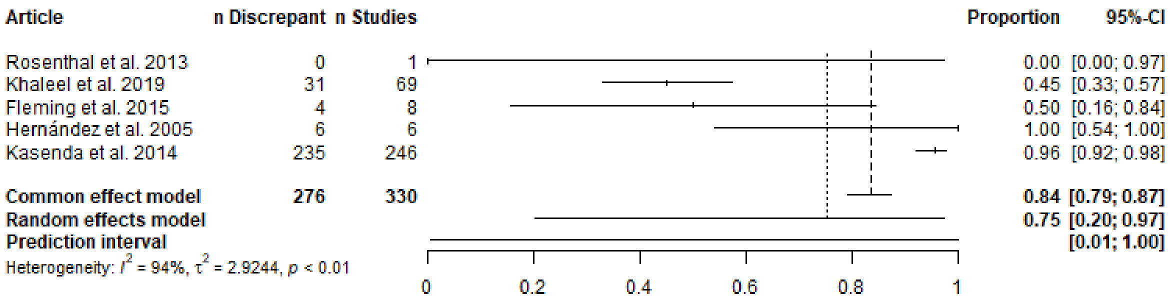

Supplementary Figure D36. Subgroup analyses, unclear registration timing.

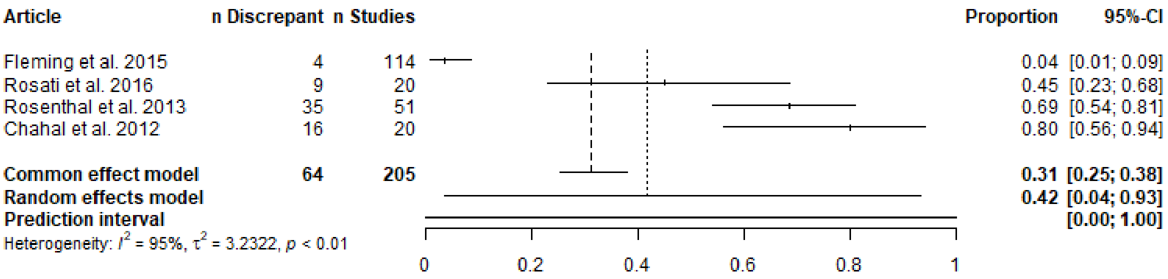

Supplementary Figure D37. Funding, unclear registration timing.

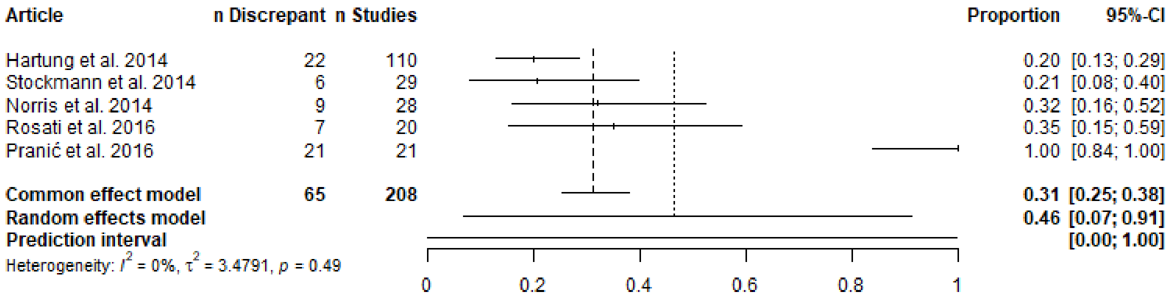

Supplementary Figure D38. Results, unclear registration timing.

E. Meta-regression model summaries

Meta-regression sensitivity analysis

We ran the meta-regressions presented in the manuscript with one predictor at a time. In line with a suggestion from a reviewer, we now also run a meta-regression with all predictors at the same time. We have no a priori thoughts on how the predictors may interact and thus do not include the interaction of predictors in our meta-regressions.

This meta-regression revealed 6 predictors with $p < 0.10$. The full output is appended below. Five of the predictors include between 1 to 4 articles in one category and thus may stem from idiosyncrasies of one or more individual studies, rather than from the predictor itself. As Cochrane advises against meta-regression when $k < 10$ (https://training.cochrane.org/handbook/current/chapter- 13#section-13-3-5-6), we do not explore these findings further.

This meta-regression revealed one predictor of potential interest. Articles that compared publications to the most recent version of a registration ($k = 15$) may be less likely to have at least one primary outcome discrepancy than articles that compared publications to the original version of a registration ($k = 29$) ($p = 0.09$; OR 95% CI: 0.32-1.07).

```
##
## Mixed-Effects Model (k = 70; tau^2 estimator: ML)
##
## tau^2 (estimated amount of residual heterogeneity):     0.3664
## tau (square root of estimated tau^2 value):             0.6053
## I^2 (residual heterogeneity / unaccounted variability): 86.00%
## H^2 (unaccounted variability / sampling variability):   7.14
##
## Tests for Residual Heterogeneity:
## Wld(df = 50) = 342.2743, p-val < .0001
## LRT(df = 50) = 458.3887, p-val < .0001
##
## Test of Moderators (coefficients 2:20):
## F(df1 = 19, df2 = 50) = 1.3420, p-val = 0.2007
##
## Model Results:
##
##                                                                estimate
## intrcpt                                                        -42.8460
## df1$pubYear                                                      0.0210
## df1$disciplineDentistry (DENT)                                   0.1016
## df1$disciplineEconomics, Econometrics and Finance (ECON)         0.4086
## df1$disciplineHealth Professions (HEAL)                          1.4988
## df1$disciplinePsychology (PSYC)                                  1.2335
## df1$sourceOther: ethics committee                                0.4129
## df1$sourceOther: funder                                          2.4149
## df1$sourcereg                                                   -0.3632
## df1$sourcereg,journal                                            0.6233
## df1$sourcereg,searchEngine                                      -0.8627
## df1$sourcesearchEngine                                          -0.2628
## df1$versionMost recent version                                  -0.5331
## df1$versionNo                                                   -0.1020
## df1$versionOther                                                -0.2049
## df1$disclosedexcludedDisclosedDiscrepancies                      0.3049
## df1$disclosedNo                                                  0.0273
```


For peer review only - http://bmjopen.bmj.com/site/about/guidelines.xhtml

```
## df1$disclosedYes, and NONE of the discrepancies were disclosed    0.2042
## df1$comparatorgrant                                              -2.3269
## df1$comparatorprotocol                                           -0.9981
##                                                                      se
## intrcpt                                                         62.0366
## df1$pubYear                                                      0.0308
## df1$disciplineDentistry (DENT)                                   0.4287
## df1$disciplineEconomics, Econometrics and Finance (ECON)         0.7221
## df1$disciplineHealth Professions (HEAL)                          0.7510
## df1$disciplinePsychology (PSYC)                                  0.6494
## df1$sourceOther: ethics committee                                0.5356
## df1$sourceOther: funder                                          1.0151
## df1$sourcereg                                                    0.2454
## df1$sourcereg,journal                                            0.7028
## df1$sourcereg,searchEngine                                       0.7230
## df1$sourcesearchEngine                                           0.2496
## df1$versionMost recent version                                   0.3005
## df1$versionNo                                                    0.2152
## df1$versionOther                                                 0.3291
## df1$disclosedexcludedDisclosedDiscrepancies                      0.4616
## df1$disclosedNo                                                  0.2301
## df1$disclosedYes, and NONE of the discrepancies were disclosed   0.3577
## df1$comparatorgrant                                              1.2765
## df1$comparatorprotocol                                           0.4604
##                                                                    tval  df
## intrcpt                                                         -0.6907  50
## df1$pubYear                                                      0.6828  50
## df1$disciplineDentistry (DENT)                                   0.2370  50
## df1$disciplineEconomics, Econometrics and Finance (ECON)         0.5659  50
## df1$disciplineHealth Professions (HEAL)                          1.9957  50
## df1$disciplinePsychology (PSYC)                                  1.8993  50
## df1$sourceOther: ethics committee                                0.7709  50
## df1$sourceOther: funder                                          2.3789  50
## df1$sourcereg                                                   -1.4799  50
## df1$sourcereg,journal                                            0.8869  50
## df1$sourcereg,searchEngine                                      -1.1933  50
## df1$sourcesearchEngine                                          -1.0527  50
## df1$versionMost recent version                                  -1.7741  50
## df1$versionNo                                                   -0.4740  50
## df1$versionOther                                                -0.6224  50
## df1$disclosedexcludedDisclosedDiscrepancies                      0.6604  50
## df1$disclosedNo                                                  0.1188  50
## df1$disclosedYes, and NONE of the discrepancies were disclosed   0.5709  50
## df1$comparatorgrant                                             -1.8229  50
## df1$comparatorprotocol                                          -2.1680  50
##                                                                   pval
## intrcpt                                                         0.4930
## df1$pubYear                                                     0.4979
## df1$disciplineDentistry (DENT)                                  0.8136
## df1$disciplineEconomics, Econometrics and Finance (ECON)        0.5740
## df1$disciplineHealth Professions (HEAL)                         0.0514
## df1$disciplinePsychology (PSYC)                                 0.0633
## df1$sourceOther: ethics committee                               0.4444
## df1$sourceOther: funder                                         0.0212
```

For peer review only

```
## df1$sourcereg                                                 0.1452
## df1$sourcereg,journal                                         0.3794
## df1$sourcereg,searchEngine                                    0.2384
## df1$sourcesearchEngine                                        0.2975
## df1$versionMost recent version                                0.0821
## df1$versionNo                                                 0.6376
## df1$versionOther                                              0.5365
## df1$disclosedexcludedDisclosedDiscrepancies                   0.5120
## df1$disclosedNo                                               0.9059
## df1$disclosedYes, and NONE of the discrepancies were disclosed  0.5706
## df1$comparatorgrant                                           0.0743
## df1$comparatorprotocol                                        0.0349
##                                                                  ci.lb
## intrcpt                                                      -167.4502
## df1$pubYear                                                    -0.0408
## df1$disciplineDentistry (DENT)                                 -0.7595
## df1$disciplineEconomics, Econometrics and Finance (ECON)       -1.0417
## df1$disciplineHealth Professions (HEAL)                        -0.0096
## df1$disciplinePsychology (PSYC)                                -0.0710
## df1$sourceOther: ethics committee                              -0.6629
## df1$sourceOther: funder                                         0.3760
## df1$sourcereg                                                  -0.8561
## df1$sourcereg,journal                                          -0.7882
## df1$sourcereg,searchEngine                                     -2.3149
## df1$sourcesearchEngine                                         -0.7641
## df1$versionMost recent version                                 -1.1366
## df1$versionNo                                                  -0.5343
## df1$versionOther                                               -0.8659
## df1$disclosedexcludedDisclosedDiscrepancies                    -0.6224
## df1$disclosedNo                                                -0.4348
## df1$disclosedYes, and NONE of the discrepancies were disclosed   -0.5142
## df1$comparatorgrant                                            -4.8908
## df1$comparatorprotocol                                         -1.9227
##                                                                 ci.ub
## intrcpt                                                       81.7581
## df1$pubYear                                                    0.0829
## df1$disciplineDentistry (DENT)                                 0.9626
## df1$disciplineEconomics, Econometrics and Finance (ECON)       1.8589
## df1$disciplineHealth Professions (HEAL)                        3.0072  .
## df1$disciplinePsychology (PSYC)                                2.5379  .
## df1$sourceOther: ethics committee                              1.4888
## df1$sourceOther: funder                                        4.4538  *
## df1$sourcereg                                                  0.1297
## df1$sourcereg,journal                                          2.0348
## df1$sourcereg,searchEngine                                     0.5895
## df1$sourcesearchEngine                                         0.2386
## df1$versionMost recent version                                 0.0704  .
## df1$versionNo                                                  0.3303
## df1$versionOther                                               0.4562
## df1$disclosedexcludedDisclosedDiscrepancies                    1.2321
## df1$disclosedNo                                                0.4895
## df1$disclosedYes, and NONE of the discrepancies were disclosed   0.9227
## df1$comparatorgrant                                            0.2370  .
## df1$comparatorprotocol                                        -0.0734  *
```


For peer review only - http://bmjopen.bmj.com/site/about/guidelines.xhtml

```
##
## ---
## Signif. codes:  0 '***' 0.001 '**' 0.01 '*' 0.05 '.' 0.1 ' ' 1
```

Discipline, primary outcomes

```
##
## Mixed-Effects Model (k = 70; tau^2 estimator: ML)
##
## tau^2 (estimated amount of residual heterogeneity):     0.4973
## tau (square root of estimated tau^2 value):             0.7052
## I^2 (residual heterogeneity / unaccounted variability): 89.80%
## H^2 (unaccounted variability / sampling variability):   9.80
##
## Tests for Residual Heterogeneity:
## Wld(df = 65) = 484.2655, p-val < .0001
## LRT(df = 65) = 619.5987, p-val < .0001
##
## Test of Moderators (coefficients 2:5):
## F(df1 = 4, df2 = 65) = 1.3093, p-val = 0.2759
##
## Model Results:
##
##                                                           estimate      se
## intrcpt                                                    -0.7695  0.0982
## df1$disciplineDentistry (DENT)                              0.1419  0.4466
## df1$disciplineEconomics, Econometrics and Finance (ECON)    0.3052  0.7434
## df1$disciplineHealth Professions (HEAL)                     0.9665  0.7569
## df1$disciplinePsychology (PSYC)                             1.1512  0.6119
##                                                              tval  df    pval
## intrcpt                                                   -7.8374  65  <.0001
## df1$disciplineDentistry (DENT)                             0.3178  65  0.7517
## df1$disciplineEconomics, Econometrics and Finance (ECON)   0.4106  65  0.6827
## df1$disciplineHealth Professions (HEAL)                    1.2768  65  0.2062
## df1$disciplinePsychology (PSYC)                            1.8813  65  0.0644
##                                                             ci.lb    ci.ub
## intrcpt                                                   -0.9656  -0.5734  ***
## df1$disciplineDentistry (DENT)                            -0.7501   1.0339
## df1$disciplineEconomics, Econometrics and Finance (ECON)  -1.1795   1.7900
## df1$disciplineHealth Professions (HEAL)                   -0.5452   2.4782
## df1$disciplinePsychology (PSYC)                           -0.0709   2.3733    .
##
## ---
## Signif. codes:  0 '***' 0.001 '**' 0.01 '*' 0.05 '.' 0.1 ' ' 1
```


For peer review only - http://bmjopen.bmj.com/site/about/guidelines.xhtml

Comparator, primary outcomes

```
##
## Mixed-Effects Model (k = 70; tau^2 estimator: ML)
##
## tau^2 (estimated amount of residual heterogeneity):     0.5031
## tau (square root of estimated tau^2 value):             0.7093
## I^2 (residual heterogeneity / unaccounted variability): 89.96%
## H^2 (unaccounted variability / sampling variability):   9.96
##
## Tests for Residual Heterogeneity:
## Wld(df = 66) = 439.1139, p-val < .0001
## LRT(df = 66) = 546.7582, p-val < .0001
##
## Test of Moderators (coefficients 2:4):
## F(df1 = 3, df2 = 66) = 0.8637, p-val = 0.4644
##
## Model Results:
##
##                                       estimate      se     tval  df    pval
## intrcpt                                -0.6892  0.0989  -6.9690  66  <.0001
## droplevels(df1$comparator)ethics        0.1085  0.4982   0.2177  66  0.8283
## droplevels(df1$comparator)grant         0.2585  0.7757   0.3333  66  0.7400
## droplevels(df1$comparator)protocol     -0.6395  0.4155  -1.5389  66  0.1286
##                                         ci.lb    ci.ub
## intrcpt                               -0.8866  -0.4917  ***
## droplevels(df1$comparator)ethics      -0.8862   1.1031
## droplevels(df1$comparator)grant       -1.2902   1.8072
## droplevels(df1$comparator)protocol    -1.4691   0.1902
##
## ---
## Signif. codes:  0 '***' 0.001 '**' 0.01 '*' 0.05 '.' 0.1 ' ' 1
```


For peer review only - http://bmjopen.bmj.com/site/about/guidelines.xhtml

Source, primary outcomes

```
##
## Mixed-Effects Model (k = 70; tau^2 estimator: ML)
##
## tau^2 (estimated amount of residual heterogeneity):     0.5197
## tau (square root of estimated tau^2 value):             0.7209
## I^2 (residual heterogeneity / unaccounted variability): 89.95%
## H^2 (unaccounted variability / sampling variability):   9.95
##
## Tests for Residual Heterogeneity:
## Wld(df = 63) = 491.6691, p-val < .0001
## LRT(df = 63) = 629.6278, p-val < .0001
##
## Test of Moderators (coefficients 2:7):
## F(df1 = 6, df2 = 63) = 0.6989, p-val = 0.6515
##
## Model Results:
##
##                                                  estimate      se     tval  df
## intrcpt                                           -0.6406  0.1456  -4.3983  63
## droplevels(df1$source)Other: ethics committee      0.0578  0.5158   0.1121  63
## droplevels(df1$source)Other: funder                0.6779  0.6178   1.0973  63
## droplevels(df1$source)reg                         -0.3005  0.2346  -1.2808  63
## droplevels(df1$source)reg,journal                  0.1826  0.7542   0.2421  63
## droplevels(df1$source)reg,searchEngine            -0.6840  0.7832  -0.8734  63
## droplevels(df1$source)searchEngine                -0.0312  0.2515  -0.1239  63
##                                                    pval    ci.lb    ci.ub
## intrcpt                                          <.0001  -0.9316  -0.3495  ***
## droplevels(df1$source)Other: ethics committee    0.9111  -0.9729   1.0886
## droplevels(df1$source)Other: funder              0.2767  -0.5566   1.9124
## droplevels(df1$source)reg                        0.2050  -0.7693   0.1684
## droplevels(df1$source)reg,journal                0.8095  -1.3245   1.6897
## droplevels(df1$source)reg,searchEngine           0.3858  -2.2491   0.8810
## droplevels(df1$source)searchEngine               0.9018  -0.5338   0.4715
##
## ---
## Signif. codes:  0 '***' 0.001 '**' 0.01 '*' 0.05 '.' 0.1 ' ' 1
```

For peer review only

Version, primary outcomes

```
##
## Mixed-Effects Model (k = 70; tau^2 estimator: ML)
##
## tau^2 (estimated amount of residual heterogeneity):     0.5251
## tau (square root of estimated tau^2 value):             0.7247
## I^2 (residual heterogeneity / unaccounted variability): 90.05%
## H^2 (unaccounted variability / sampling variability):   10.05
##
## Tests for Residual Heterogeneity:
## Wld(df = 66) = 485.3920, p-val < .0001
## LRT(df = 66) = 612.4631, p-val < .0001
##
## Test of Moderators (coefficients 2:4):
## F(df1 = 3, df2 = 66) = 0.3719, p-val = 0.7735
##
## Model Results:
##
##                                  estimate      se     tval  df    pval    ci.lb
## intrcpt                           -0.6048  0.1599  -3.7832  66  0.0003  -0.9239
## df1$versionMost recent version   -0.2477  0.2642  -0.9376  66  0.3519  -0.7752
## df1$versionNo                     -0.1046  0.2299  -0.4551  66  0.6505  -0.5636
## df1$versionOther                  -0.2433  0.3301  -0.7372  66  0.4636  -0.9023
##                                    ci.ub
## intrcpt                          -0.2856  ***
## df1$versionMost recent version   0.2798
## df1$versionNo                     0.3543
## df1$versionOther                  0.4157
##
## ---
## Signif. codes:  0 '***' 0.001 '**' 0.01 '*' 0.05 '.' 0.1 ' ' 1
```

For peer review only

Disclosed, primary outcomes

```
##
## Mixed-Effects Model (k = 70; tau^2 estimator: ML)
##
## tau^2 (estimated amount of residual heterogeneity):     0.5403
## tau (square root of estimated tau^2 value):             0.7350
## I^2 (residual heterogeneity / unaccounted variability): 90.43%
## H^2 (unaccounted variability / sampling variability):   10.45
##
## Tests for Residual Heterogeneity:
## Wld(df = 66) = 494.1488, p-val < .0001
## LRT(df = 66) = 644.7748, p-val < .0001
##
## Test of Moderators (coefficients 2:4):
## F(df1 = 3, df2 = 66) = 0.0739, p-val = 0.9738
##
## Model Results:
##
##                                                           estimate
## intrcpt                                                    -0.7709
## df1$disclosedexcludedDisclosedDiscrepancies                 0.2332
## df1$disclosedNo                                             0.0600
## df1$disclosedYes, and NONE of the discrepancies were disclosed    0.0611
##                                                               se     tval
## intrcpt                                                   0.2012  -3.8314
## df1$disclosedexcludedDisclosedDiscrepancies               0.5094   0.4578
## df1$disclosedNo                                           0.2362   0.2541
## df1$disclosedYes, and NONE of the discrepancies were disclosed  0.3419   0.1788
##                                                           df    pval
## intrcpt                                                   66  0.0003
## df1$disclosedexcludedDisclosedDiscrepancies               66  0.6486
## df1$disclosedNo                                           66  0.8002
## df1$disclosedYes, and NONE of the discrepancies were disclosed  66  0.8587
##                                                             ci.lb
## intrcpt                                                   -1.1725
## df1$disclosedexcludedDisclosedDiscrepancies               -0.7838
## df1$disclosedNo                                           -0.4115
## df1$disclosedYes, and NONE of the discrepancies were disclosed  -0.6215
##                                                             ci.ub
## intrcpt                                                   -0.3692  ***
## df1$disclosedexcludedDisclosedDiscrepancies                1.2502
## df1$disclosedNo                                            0.5315
## df1$disclosedYes, and NONE of the discrepancies were disclosed   0.7437
##
## ---
## Signif. codes:  0 '***' 0.001 '**' 0.01 '*' 0.05 '.' 0.1 ' ' 1
```


For peer review only - http://bmjopen.bmj.com/site/about/guidelines.xhtml

Publication year, primary outcomes

```
##
## Mixed-Effects Model (k = 70; tau^2 estimator: ML)
##
## tau^2 (estimated amount of residual heterogeneity):     0.5405
## tau (square root of estimated tau^2 value):             0.7352
## I^2 (residual heterogeneity / unaccounted variability): 90.44%
## H^2 (unaccounted variability / sampling variability):   10.46
##
## Tests for Residual Heterogeneity:
## Wld(df = 68) = 505.5885, p-val < .0001
## LRT(df = 68) = 647.2738, p-val < .0001
##
## Test of Moderators (coefficient 2):
## F(df1 = 1, df2 = 68) = 0.0438, p-val = 0.8348
##
## Model Results:
##
##                estimate       se     tval  df    pval      ci.lb    ci.ub
## intrcpt        -11.8783  53.3266  -0.2227  68  0.8244  -118.2899  94.5332
## df1$pubYear      0.0055   0.0265   0.2093  68  0.8348    -0.0473   0.0584
##
## ---
## Signif. codes:  0 '***' 0.001 '**' 0.01 '*' 0.05 '.' 0.1 ' ' 1
```

For peer review only

Discpline binary, primary outcomes

```
##
## Mixed-Effects Model (k = 70; tau^2 estimator: ML)
##
## tau^2 (estimated amount of residual heterogeneity):     0.5175
## tau (square root of estimated tau^2 value):             0.7194
## I^2 (residual heterogeneity / unaccounted variability): 90.11%
## H^2 (unaccounted variability / sampling variability):   10.11
##
## Tests for Residual Heterogeneity:
## Wld(df = 68) = 497.9543, p-val < .0001
## LRT(df = 68) = 634.4749, p-val < .0001
##
## Test of Moderators (coefficient 2):
## F(df1 = 1, df2 = 68) = 2.8890, p-val = 0.0938
##
## Model Results:
##
##                                  estimate      se     tval  df    pval    ci.lb
## intrcpt                           -0.7703  0.0999  -7.7124  68  <.0001  -0.9696
## dfDiscipline1$disciplineother      0.5342  0.3143   1.6997  68  0.0938  -0.0929
##                                     ci.ub
## intrcpt                           -0.5710  ***
## dfDiscipline1$disciplineother      1.1613    .
##
## ---
## Signif. codes:  0 '***' 0.001 '**' 0.01 '*' 0.05 '.' 0.1 ' ' 1
```


For peer review only - http://bmjopen.bmj.com/site/about/guidelines.xhtml

Comparator binary, primary outcomes

```
##
## Mixed-Effects Model (k = 70; tau^2 estimator: ML)
##
## tau^2 (estimated amount of residual heterogeneity):     0.5305
## tau (square root of estimated tau^2 value):             0.7284
## I^2 (residual heterogeneity / unaccounted variability): 90.37%
## H^2 (unaccounted variability / sampling variability):   10.38
##
## Tests for Residual Heterogeneity:
## Wld(df = 68) = 491.3839, p-val < .0001
## LRT(df = 68) = 609.5590, p-val < .0001
##
## Test of Moderators (coefficient 2):
## F(df1 = 1, df2 = 68) = 0.6521, p-val = 0.4222
##
## Model Results:
##
##                               estimate      se     tval  df    pval    ci.lb
## intrcpt                        -0.9418  0.2945  -3.1978  68  0.0021  -1.5296
## df1Comparator$comparatorreg     0.2516  0.3115   0.8076  68  0.4222  -0.3701
##                                  ci.ub
## intrcpt                        -0.3541  **
## df1Comparator$comparatorreg     0.8733
##
## ---
## Signif. codes:  0 '***' 0.001 '**' 0.01 '*' 0.05 '.' 0.1 ' ' 1
```


For peer review only - http://bmjopen.bmj.com/site/about/guidelines.xhtml

For peer review only

Discipline, secondary outcomes

```
##
## Mixed-Effects Model (k = 22; tau^2 estimator: ML)
##
## tau^2 (estimated amount of residual heterogeneity):     1.1358
## tau (square root of estimated tau^2 value):             1.0658
## I^2 (residual heterogeneity / unaccounted variability): 92.81%
## H^2 (unaccounted variability / sampling variability):   13.91
##
## Tests for Residual Heterogeneity:
## Wld(df = 19) = 152.4015, p-val < .0001
## LRT(df = 19) = 232.6890, p-val < .0001
##
## Test of Moderators (coefficients 2:3):
## F(df1 = 2, df2 = 19) = 1.3121, p-val = 0.2926
##
## Model Results:
##
##                                          estimate      se     tval  df    pval
## intrcpt                                    0.5830  0.2642   2.2067  19  0.0398
## df2$disciplineDentistry (DENT)            -0.9288  0.8150  -1.1396  19  0.2686
## df2$disciplineHealth Professions (HEAL)    1.2320  1.1580   1.0639  19  0.3007
##                                            ci.lb   ci.ub
## intrcpt                                   0.0300  1.1359  *
## df2$disciplineDentistry (DENT)           -2.6346  0.7771
## df2$disciplineHealth Professions (HEAL)  -1.1917  3.6557
##
## ---
## Signif. codes:  0 '***' 0.001 '**' 0.01 '*' 0.05 '.' 0.1 ' ' 1
```

For peer review only

Comparator, secondary outcomes

```
##
## Mixed-Effects Model (k = 22; tau^2 estimator: ML)
##
## tau^2 (estimated amount of residual heterogeneity):     1.1341
## tau (square root of estimated tau^2 value):             1.0650
## I^2 (residual heterogeneity / unaccounted variability): 93.39%
## H^2 (unaccounted variability / sampling variability):   15.12
##
## Tests for Residual Heterogeneity:
## Wld(df = 20) = 188.4912, p-val < .0001
## LRT(df = 20) = 268.8685, p-val < .0001
##
## Test of Moderators (coefficient 2):
## F(df1 = 1, df2 = 20) = 3.4451, p-val = 0.0782
##
## Model Results:
##
##                                    estimate      se    tval  df    pval
## intrcpt                              0.4164  0.2535  1.6425  20  0.1161
## droplevels(df2$comparator)protocol   1.7751  0.9564  1.8561  20  0.0782
##                                      ci.lb   ci.ub
## intrcpt                            -0.1124  0.9452
## droplevels(df2$comparator)protocol -0.2198  3.7700  .
##
## ---
## Signif. codes:  0 '***' 0.001 '**' 0.01 '*' 0.05 '.' 0.1 ' ' 1
```


For peer review only - http://bmjopen.bmj.com/site/about/guidelines.xhtml

Source, secondary outcomes

```
##
## Mixed-Effects Model (k = 22; tau^2 estimator: ML)
##
## tau^2 (estimated amount of residual heterogeneity):     0.6838
## tau (square root of estimated tau^2 value):             0.8269
## I^2 (residual heterogeneity / unaccounted variability): 88.00%
## H^2 (unaccounted variability / sampling variability):   8.33
##
## Tests for Residual Heterogeneity:
## Wld(df = 16) = 135.1594, p-val < .0001
## LRT(df = 16) = 187.0805, p-val < .0001
##
## Test of Moderators (coefficients 2:6):
## F(df1 = 5, df2 = 16) = 3.3793, p-val = 0.0284
##
## Model Results:
##
##                              estimate      se     tval  df    pval    ci.lb
## intrcpt                       -0.3517  0.3203  -1.0981  16  0.2884  -1.0306
## df2$sourcejournal              1.6186  0.4626   3.4989  16  0.0030   0.6379
## df2$sourceOther: funder        2.9203  1.4041   2.0798  16  0.0540  -0.0563
## df2$sourcereg,journal          0.9517  0.9029   1.0540  16  0.3075  -0.9624
## df2$sourcereg,searchEngine     2.1280  0.9400   2.2639  16  0.0378   0.1353
## df2$sourcesearchEngine         0.5912  0.6049   0.9774  16  0.3429  -0.6911
##                               ci.ub
## intrcpt                      0.3272
## df2$sourcejournal            2.5993  **
## df2$sourceOther: funder      5.8968   .
## df2$sourcereg,journal        2.8658
## df2$sourcereg,searchEngine   4.1206   *
## df2$sourcesearchEngine       1.8735
##
## ---
## Signif. codes:  0 '***' 0.001 '**' 0.01 '*' 0.05 '.' 0.1 ' ' 1
```

For peer review only

Version, secondary outcomes

```
##
## Mixed-Effects Model (k = 22; tau^2 estimator: ML)
##
## tau^2 (estimated amount of residual heterogeneity):     1.2281
## tau (square root of estimated tau^2 value):             1.1082
## I^2 (residual heterogeneity / unaccounted variability): 93.15%
## H^2 (unaccounted variability / sampling variability):   14.60
##
## Tests for Residual Heterogeneity:
## Wld(df = 18) = 164.5429, p-val < .0001
## LRT(df = 18) = 247.8955, p-val < .0001
##
## Test of Moderators (coefficients 2:4):
## F(df1 = 3, df2 = 18) = 0.3299, p-val = 0.8038
##
## Model Results:
##
##                                    estimate      se     tval  df    pval    ci.lb
## intrcpt                              0.3831  0.4475   0.8560  18  0.4033  -0.5571
## df2$versionMost recent version       0.5515  0.6480   0.8512  18  0.4058  -0.8098
## df2$versionNo                       -0.0300  0.6500  -0.0462  18  0.9637  -1.3956
## df2$versionOther                     0.1605  0.9210   0.1743  18  0.8636  -1.7744
##                                     ci.ub
## intrcpt                            1.3233
## df2$versionMost recent version     1.9128
## df2$versionNo                      1.3356
## df2$versionOther                   2.0954
##
## ---
## Signif. codes:  0 '***' 0.001 '**' 0.01 '*' 0.05 '.' 0.1 ' ' 1
```

For peer review only

Disclosed, secondary outcomes

```
##
## Mixed-Effects Model (k = 22; tau^2 estimator: ML)
##
## tau^2 (estimated amount of residual heterogeneity):     1.2148
## tau (square root of estimated tau^2 value):             1.1022
## I^2 (residual heterogeneity / unaccounted variability): 93.11%
## H^2 (unaccounted variability / sampling variability):   14.52
##
## Tests for Residual Heterogeneity:
## Wld(df = 18) = 173.0004, p-val < .0001
## LRT(df = 18) = 241.2204, p-val < .0001
##
## Test of Moderators (coefficients 2:4):
## F(df1 = 3, df2 = 18) = 0.5040, p-val = 0.6843
##
## Model Results:
##
##                                                                 estimate
## intrcpt                                                           0.2683
## df2$disclosedexcludedDisclosedDiscrepancies                       0.7064
## df2$disclosedNo                                                   0.3885
## df2$disclosedYes, and NONE of the discrepancies were disclosed   -0.5224
##                                                                     se     tval
## intrcpt                                                         0.6643   0.4039
## df2$disclosedexcludedDisclosedDiscrepancies                     1.0390   0.6799
## df2$disclosedNo                                                 0.7324   0.5304
## df2$disclosedYes, and NONE of the discrepancies were disclosed  1.0664  -0.4899
##                                                                 df    pval
## intrcpt                                                         18  0.6910
## df2$disclosedexcludedDisclosedDiscrepancies                     18  0.5052
## df2$disclosedNo                                                 18  0.6023
## df2$disclosedYes, and NONE of the discrepancies were disclosed  18  0.6301
##                                                                   ci.lb   ci.ub
## intrcpt                                                         -1.1273  1.6639
## df2$disclosedexcludedDisclosedDiscrepancies                     -1.4765  2.8894
## df2$disclosedNo                                                 -1.1503  1.9272
## df2$disclosedYes, and NONE of the discrepancies were disclosed  -2.7627  1.7179
##
## intrcpt
## df2$disclosedexcludedDisclosedDiscrepancies
## df2$disclosedNo
## df2$disclosedYes, and NONE of the discrepancies were disclosed
##
## ---
## Signif. codes:  0 '***' 0.001 '**' 0.01 '*' 0.05 '.' 0.1 ' ' 1
```


For peer review only - http://bmjopen.bmj.com/site/about/guidelines.xhtml

Publication year, secondary outcomes

```
##
## Mixed-Effects Model (k = 22; tau^2 estimator: ML)
##
## tau^2 (estimated amount of residual heterogeneity):     1.3181
## tau (square root of estimated tau^2 value):             1.1481
## I^2 (residual heterogeneity / unaccounted variability): 93.94%
## H^2 (unaccounted variability / sampling variability):   16.51
##
## Tests for Residual Heterogeneity:
## Wld(df = 20) = 199.2193, p-val < .0001
## LRT(df = 20) = 284.0615, p-val < .0001
##
## Test of Moderators (coefficient 2):
## F(df1 = 1, df2 = 20) = 0.1305, p-val = 0.7217
##
## Model Results:
##
##                estimate        se     tval  df    pval      ci.lb     ci.ub
## intrcpt        -55.6213  155.4756  -0.3577  20  0.7243  -379.9378  268.6951
## df2$pubYear      0.0279    0.0772   0.3613  20  0.7217    -0.1331    0.1889
##
## ---
## Signif. codes:  0 '***' 0.001 '**' 0.01 '*' 0.05 '.' 0.1 ' ' 1
```

For peer review only

F. Parameters potentially related to discrepancies

Disclosure of discrepancies

Some publications disclose the discrepancies between their registration or study protocol and publication. Of the articles we reviewed, k = 8 state that none of the publications disclose their primary outcome discrepancies, k = 13 state that one or more publication disclosed one or more primary outcome discrepancy, k = 68 do not report how many publications disclosed their primary outcome discrepancies, and k = 4 included only publications with undisclosed discrepancies in their sample. A random effects meta-analysis of the k = 21 articles that report the number of disclosed primary outcome discrepancies estimates that 4-19% (95% CI) (95% prediction interval: 0.3-74%) of publications with a discrepancy at least mention that a discrepancy is present (see Supplementary Figure F1 for a forest plot). Notably, a mention does not imply that the publication justified that discrepancy (e.g., Pandis et al., 2015). A few articles identified a much higher proportion of publications that disclosed discrepancies compared to the other articles. These articles focused on acupuncture (Su et al., 2015), meta-analyses (Delgado & Delgado, 2017b), and systematic reviews (Dwan, Kirkham, et al., 2013).

In our initial coding form, we identified 19 articles reporting that the studies they assessed disclosed one or more discrepancy and 9 articles reporting that the studies they assessed disclosed no discrepancies (28 articles total). Only 21 of these 28 articles reported the number of studies assessed and the number of discrepancies disclosed (or percentages from which we could calculate these values) This forest plot only includes data from those 21 articles.

Statistical significance

k = 24 articles checked whether discrepancies favored statistical significance. Of the studies for which favoring statistical significance could be determined, 49-66% (95% CI) (95% prediction interval: 23-86%) of discrepancies favored statistical significance (see Supplementary Figure F2 for a forest plot).

k = 7 articles provided information on the prevalence of discrepancies in relation to statistical significance. Studies with discrepancies may be *less* likely than studies without discrepancies to report a statistically significant result—likelihood ratio: 0.56-1.06 (95% CI) (95% prediction interval: 0.42-1.43) (see Supplementary Figure F3 for a forest plot). Likewise, rearranging the analysis on this same data shows that studies that report a statistically significant finding are *less* likely to contain a discrepancy—likelihood ratio: 0.64-0.99 (95% CI) (95% prediction interval: 0.59-1.06) (see Supplementary Figure F4 for a forest plot). Whereas these findings may appear counterintuitive, a sampling bias—in the sense that the incentive to switch outcomes is less apparent when the registered outcome reaches statistical significance—may influence these results.

Industry funding

k = 22 articles provided information on the prevalence of discrepancies in relation to the type of funding a study received. A meta-analysis of these articles shows that industry funded studies are perhaps *less* likely to contain discrepancies compared to non-industry funded studies—likelihood ratio: 0.61-0.91 (95% CI) (95% prediction interval: 0.44-1.27) (see Supplementary Figure F5 for a forest plot).

Timing of registration

k = 4 articles compared whether prospectively registered studies were more likely to have primary outcome discrepancies compared to retrospectively registered studies. A meta-analysis of these studies did not find a difference—likelihood ratio: 0.46-2.57 (95% CI) (95% prediction interval: 0.13-8.85) (see Supplementary Figure F6 for a forest plot).

For peer review only - http://bmjopen.bmj.com/site/about/guidelines.xhtml

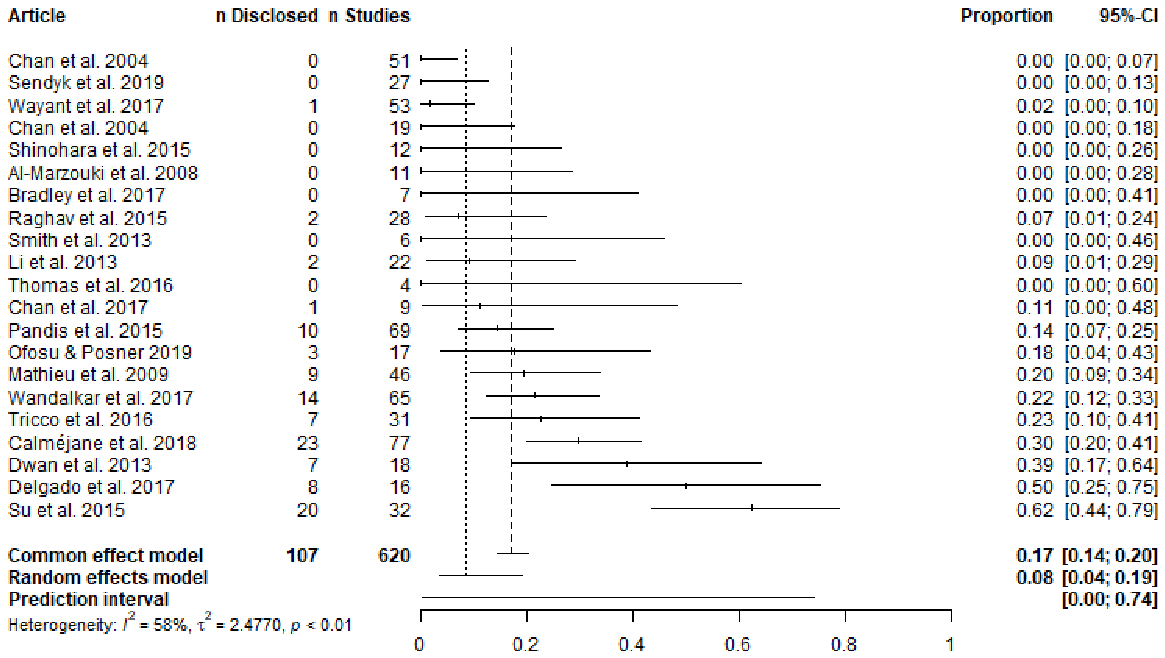

Supplementary Figure F1. Proportion of studies with at least one outcome discrepancy that also disclose an outcome discrepancy.

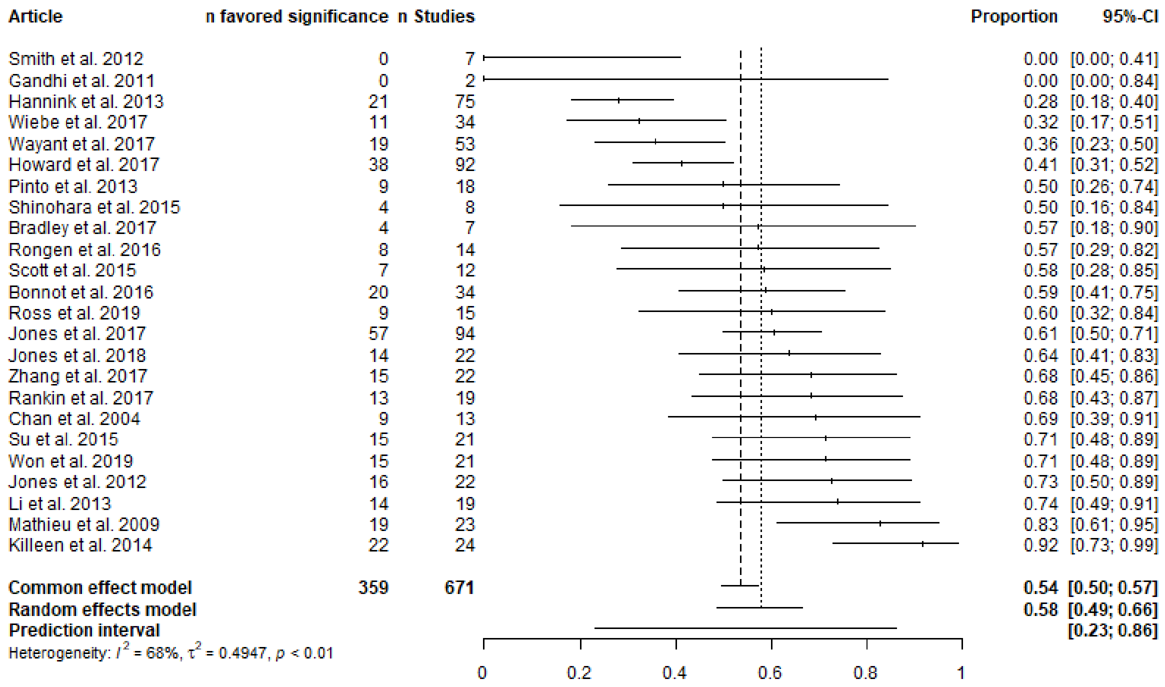

Supplementary Figure F2. Proportion of outcome discrepancies that favor statistically significant results.

For peer review only - http://bmjopen.bmj.com/site/about/guidelines.xhtml

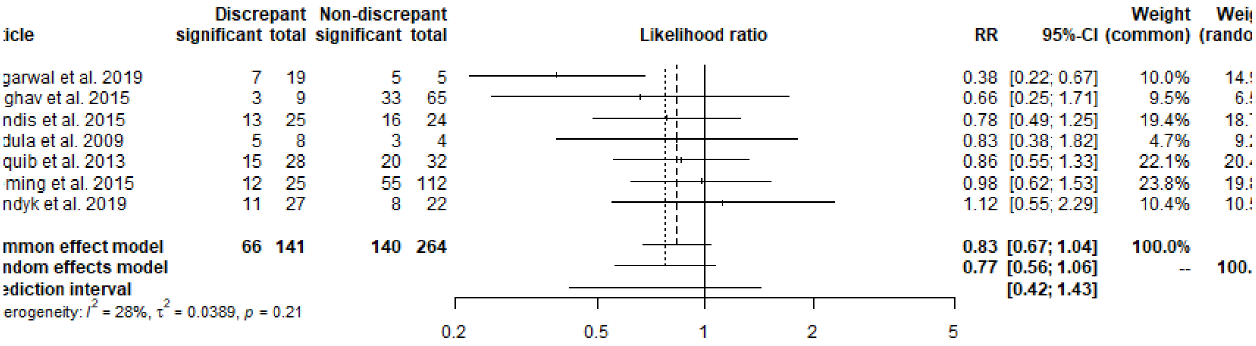

Supplementary Figure F3. Likelihood that a publication with at least one outcome discrepancy, compared to a publication with no outcome discrepancy, reports statistically significant results.

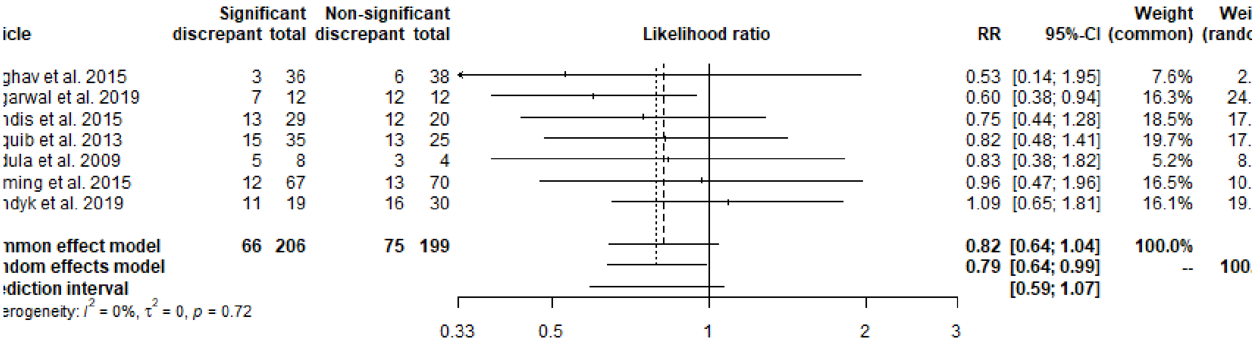

Supplementary Figure F4. Likelihood that a publication reporting a statistically significant result, compared to a publication not reporting a statistically significant results, contains at least one outcome discrepancy.

For peer review only - http://bmjopen.bmj.com/site/about/guidelines.xhtml

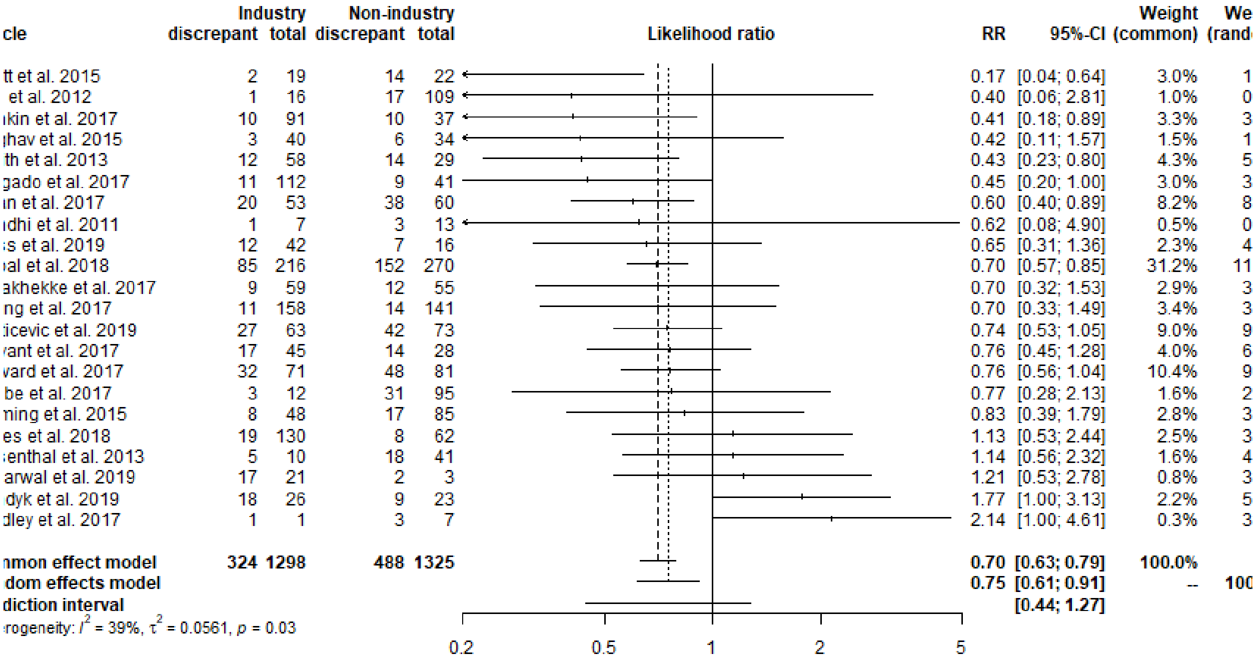

Supplementary Figure F5. Likelihood that a study funded by industry, compared to a study not funded by industry, includes at least one outcome discrepancy.

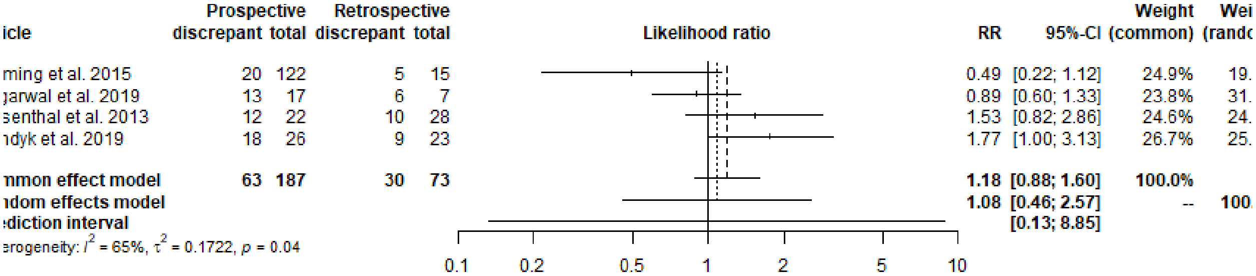

Supplementary Figure F6. Likelihood that a prospectively registered study, compared to a retrospectively registered study, includes at least one primary outcome discrepancy.

For peer review only - http://bmjopen.bmj.com/site/about/guidelines.xhtml

G. Non-outcome discrepancies

Eligibility

Discrepancies in eligibility were sometimes reported separately for inclusion and exclusion criteria. In this case, we took the larger of the two numbers because at least that many studies had a discrepancy in eligibility. Other definitions such as a "change in study population" were also used. While our meta-analysis (k = 1153) estimates that 25-57% (95% CI) (95% prediction interval: 5-90%) of publications have at least one discrepancy in eligibility criteria, a broader definition of eligibility would yield an estimate in the higher end of this interval.

Sample size

Of the k = 1398 articles that report on discrepancies in sample size in our meta-analysis, two required a sample size difference of at least 20%, 7 required a sample size difference of at least 10%, one required a sample size difference of at least 6%, and 15 had no mention of a minimum magnitude of change in sample size. A few articles reported both any discrepancy in sample size and discrepancies surpassing a specified magnitude. In these cases, we counted the discrepancies that reached the largest magnitude cut-off identified in the article. An estimated 26-44% (95% CI) (95% prediction interval: 8-78%) of studies had discrepancies in their sample size.

Randomization and blinding

Articles varied greatly in the way they counted discrepancies in randomization. One article surveyed for "a general statement about randomization" (Rosenthal & Dwan, 2013); one counted discrepancies in randomization if there was a change in the number of arms of a study (Sendyk et al., 2019); and one assessed only three studies with generated allocation sequences (Su et al., 2015). These articles found few discrepancies. Other articles counted studies that did not report randomization (Soares et al., 2004) or checked for discrepancies in the randomization procedure (Fleming et al., 2015). These articles found many discrepancies. Few studies include details about randomization in both their registry and publication (Fleming et al., 2015).

Only three of the k = 89 articles we reviewed reported discrepancies in blinding by checking who was blinded (Fleming et al., 2015; Rosenthal & Dwan, 2013; Su et al., 2015). Methods and results were less heterogeneous as compared to discrepancies in randomization.

Intervention

Five hundred and fifty articles reported on discrepancies in interventions. Three of these articles only counted high-level discrepancies (e.g., the name of the intervention) and found few discrepancies (Korevaar et al., 2014; Rosenthal & Dwan, 2013; Su et al., 2015). Three other articles counted more fine-grained discrepancies in interventions (e.g., dosage, duration of administration) and found that discrepancies were common (Huić et al., 2011; Jurić et al., 2020; Pranić & Marušić, 2016).

Study duration

One hundred and eighty-four articles reported on discrepancies in study duration. Although the definition of a discrepancy was similar among three of the studies, their results varied substantially—between 3% to 65% discrepant (Rosati et al., 2016; Rosenthal & Dwan, 2013; Shepshelovich et al., 2018).

For peer review only - http://bmjopen.bmj.com/site/about/guidelines.xhtml

Analyses and subgroup analyses

Twelve articles checked for discrepancies in analysis plans in general, and k = 9 checked specifically for discrepancies in subgroup analyses. Very few studies included a general analysis plan, or a subgroup analysis plan, in both their registration and publication (e.g., Fleming et al., 2015; Rosenthal & Dwan, 2013). Some articles we reviewed only evaluated discrepancies for studies that included an analysis in both their registration and publication. Three of the articles with low estimates for analysis discrepancies only checked whether plans to conduct intention-to-treat analyses were followed (Fleming et al., 2015; Rosati et al., 2016; Soares et al., 2004). Other articles checked for discrepancies in the statistical test for the primary outcome (Chan et al., 2008) or in model adjustments (Saquib et al., 2013). If articles evaluated a broader spectrum of discrepancies in the analyses they checked, and if they considered the addition of an analyses as a discrepancy, the resulting point estimate would be in the higher end of this interval: 19-52% (95% CI) (95% prediction interval: 4-86%). Discrepancies in subgroup analyses were more common: 35-93% (95% CI) (95% prediction interval: 2-99.7%).

Funding and results

Discrepancies in funding source and reported results are not directly relevant to the main theme of this paper because they do not imply a deviation from a prospectively planned study protocol. We coded for these discrepancies nonetheless, but the intervals are too wide to draw conclusions about their prevalence (Table S2).

For peer review only

For peer review only - http://bmjopen.bmj.com/site/about/guidelines.xhtml

H. Gaps in the literature

In addition to the quantitative data presented thus far, our reviewers took qualitative notes including a conclusion from each article, details regarding the source used to identify studies and the sub-disciplines they surveyed, and other ad hoc observations. One reviewer (RTT) extracted themes related to discrepancies and identified gaps in the literature using narrative synthesis. This process revealed several issues parallel to discrepancies: many studies are never registered, registrations are often imprecise, they rarely include information on analysis plans, and additional initiatives or enforcement may be necessary to improve registration in practice (see Table S3 for a list of themes and article quotes).

We further identified at least four gaps in the literature: (1) discrepancy prevalence in fields other than clinical research, (2) discrepancy prevalence in a representative sample across clinical disciplines, (3) the level of detail in registrations, and (4) interventions that attempt to reduce undisclosed discrepancies.

Only one preprint surveyed each of psychology and economics, and no other article assessed preclinical or animal research, or sourced registrations from the Open Science Framework, AsPredicted.org, the American Economic Association (AEA) and Evidence in Governance and Politics (EGAP) registration platform, or the Registry for International Development Impact Evaluations (RIDIE). Notably, registration standards differ across disciplines and this necessitates different methods for identifying discrepancies. For example, pre-analysis plans on EGAP often contain over 10 hypotheses (Ofosu & Posner, 2019) and clinicaltrials.gov does not contain a question regarding an analysis plan.

Many articles we reviewed restricted their search to high-impact factor journals or a specific clinical discipline. Randomly sampling registrations and publications from the broader clinical literature could better estimate the prevalence of discrepancies at the level where some interventions could be applied (e.g., an ICMJE policy). With a sufficient sample size, random sampling could allow comparisons among clinical disciplines and the potential to identify best practices.

Most articles did not identify the level of specificity with which an outcome was registered. For example, outcomes can be specified at the level of domain (e.g., anxiety), specific measurement (e.g., Hamilton Anxiety Rating Scale), specific metric (e.g. change from baseline), and method of aggregation (e.g., proportion of participants with decrease >50%) (example taken from Deborah A. Zarin et al. (2011)). Outcomes registered with low specificity allow for a greater range of researcher degrees of freedom. Future research efforts can also benefit from identifying whether the registrations were prospective, which version of the registration was being assessed, and if discrepancies were disclosed. This information would help reveal how problematic the discrepancies are.

Finally, nearly all the articles focused on documenting discrepancies. Future efforts may be more fruitful if they attempt to understand the causes of discrepancies and seek to develop and evaluate solutions to the issue (e.g., following the framework for meta-research presented by Hardwicke et al. (2020)).

For peer review only - http://bmjopen.bmj.com/site/about/guidelines.xhtml

Supplementary Table H1. Additional themes related to registration and discrepancies

Theme	Quotes
Many studies are never registered	"The more concerning issue, however, is the lack of registration or provision of registration number for randomized controlled trials within these journals." (Wiebe et al., 2017)<br>"Only 16 per cent of the published orthodontic RCTs had been registered." (Koufatzidou et al., 2019)
Registrations are imprecise	"Our results suggest that [selective outcome reporting] might well be substantial; however, the bias can only be broadly identified as protocols are not sufficiently precise." (Hahn et al., 2002)<br>"Only 5 out of the 109 trials (5%) provided enough information for us to be confident that the outcomes reported in the published trial were consistent with the original registration" (Nankervis et al., 2012)
Consumers of research should be aware of outcome switching	"If clinicians base their decisions on evidence distorted by primary outcome switching, patient care could be negatively affected" (C. W. Jones et al., 2017)<br>"Readers of medical literature should not assume that selective outcome reporting is not present, even if the report has been published in a high IF journal." (Shinohara et al., 2015)
Registration should be mandated or enforced	"there is insufficient quality control of data by data providers, ClinicalTrials.gov administrators, and journal editors, emphasizing much-needed enforcement of complete descriptions of drug interventions." (Jurić et al., 2020)<br>"Journal editors, regulators, research ethics committees, funders, and sponsors should implement policies mandating prospective registration for all clinical trials" (Chan et al., 2017)<br>"Reporting completeness and consistency were significantly better after July 2005. The ICMJE requirement for mandatory registration was associated with significant improvement in reporting quality in infectious diseases trials." (Shepshelovich et al., 2017)
Additional initiatives are needed to reduce reporting discrepancies	"Efforts to prevent nonregistration of protocols and selective reporting are needed." (Krsticevic et al., 2019)<br>"To foster credible evidence-based medicine, additional initiatives are needed to minimize selective reporting." (Raghav et al., 2015)<br>"Novel approaches are required to address this problem." (Fleming et al., 2015)
Analysis plans should be registered	"More immediate and specific remedies for the deficiencies of diet trial registries include (1) posting a detailed final statistical analysis plan before unmasking random group assignments or beginning data analyses as a minimum quality criterion..." (Ludwig et al., 2019)<br>"Definitive judgments regarding credibility of claimed subgroup effects are not possible without access to protocols and analysis plans of randomised controlled trials." (Kasenda et al., 2014)

For peer review only - http://bmjopen.bmj.com/site/about/guidelines.xhtml

PRISMA 2020 for Abstracts Checklist

Section and Topic	Item #	Checklist item	Reported (Yes/No)
**TITLE**			
Title	1	Identify the report as a systematic review.	Yes
**BACKGROUND**			
Objectives	2	Provide an explicit statement of the main objective(s) or question(s) the review addresses.	Yes
**METHODS**			
Eligibility criteria	3	Specify the inclusion and exclusion criteria for the review.	Yes
Information sources	4	Specify the information sources (e.g. databases, registers) used to identify studies and the date when each was last searched.	Yes
Risk of bias	5	Specify the methods used to assess risk of bias in the included studies.	No. We did not perform a formal risk of bias assessment because our review differed substantially from the purpose these tools were built for. We address this point in the main text of the manuscript.
Synthesis of results	6	Specify the methods used to present and synthesise results.	Yes
**RESULTS**			
Included studies	7	Give the total number of included studies and participants and summarise relevant characteristics of studies.	Yes
Synthesis of results	8	Present results for main outcomes, preferably indicating the number of included studies and participants for each. If meta-analysis was done, report the summary estimate and confidence/credible interval. If comparing groups, indicate the direction of the effect (i.e. which group is favoured).	Yes
**DISCUSSION**			
Limitations of evidence	9	Provide a brief summary of the limitations of the evidence included in the review (e.g. study risk of bias, inconsistency and imprecision).	Yes
Interpretation	10	Provide a general interpretation of the results and important implications.	Yes
**OTHER**			
Funding	11	Specify the primary source of funding for the review.	No. There was no funding provided specifically for this review. This information is included after the main text.
Registration	12	Provide the register name and registration number.	Yes

For peer review only

PRISMA 2020 for Abstracts Checklist

From: Page MJ, McKenzie JE, Bossuyt PM, Boutron I, Hoffmann TC, Mulrow CD, et al. The PRISMA 2020 statement: an updated guideline for reporting systematic reviews. BMJ 2021;372:n71. doi: 10.1136/bmj.n71

For more information, visit: http://www.prisma-statement.org/

For peer review only

PRISMA 2020 Checklist

Section and Topic	Item #	Checklist item	Location where item is reported
**TITLE**			
Title	1	Identify the report as a systematic review.	Title
**ABSTRACT**			
Abstract	2	See the PRISMA 2020 for Abstracts checklist.	Abstract
**INTRODUCTION**			
Rationale	3	Describe the rationale for the review in the context of existing knowledge.	"In 2000, ClinicalTrials.gov and the ISRCTN Registry were launched with several aims, including aiding participant recruitment, facilitating knowledge synthesis, and reducing duplication, publication bias and selective reporting (Zarin et al., 2017). In 2005, the International Committee of Medical Journal Editors (ICMJE) made prospective registration a condition of consideration for publication (De Angelis et al., 2004). Thousands of journals now claim to follow this policy (ICMJE, 2021). In parallel, the World Health Organization International Clinical Trials Registry Platform established a minimum set of required information for a trial to be considered fully registered, including experimental design elements such as the conditions being studied, intervention, key inclusion and exclusion criteria, sample size, primary outcomes, and key secondary outcomes (Sim et al., 2006). While the relatively widespread uptake of clinical trial registration has substantially improved transparency, many trials remain unregistered, are registered after enrollment of participants begins or analyses are complete (i.e., retrospective registration), are never published, or publish outcomes discrepant with those in the registration without disclosing the discrepancy (e.g., Chan et al., 2017; Scott et al., 2015). Nevertheless, the existence of registries allows researchers to identify and quantify these issues."
Objectives	4	Provide an explicit statement of the objective(s) or question(s) the review addresses.	"Here we systematically review articles that quantify the prevalence of discrepancies between registrations or study protocols and their associated publications (e.g., in primary outcome measures)."
**METHODS**			
Eligibility criteria	5	Specify the inclusion and exclusion criteria for the review and how studies were grouped for the syntheses.	"We excluded conference proceedings and articles written in a language other than English (for full inclusion and exclusion criteria, see our protocol osf.io/ktmdg)."
Information sources	6	Specify all databases, registers, websites, organisations, reference lists and other sources searched or consulted to identify studies. Specify the date when each source was last searched or consulted.	"We searched Scopus and Web of Science on 15th December 2019 using the queries in Appendix A and Appendix B of our preregistered protocol osf.io/ktmdg."
Search strategy	7	Present the full search strategies for all databases, registers and websites, including any filters and limits used.	"We searched Scopus and Web of Science on 15th December 2019 using the queries in Appendix A and Appendix B of our preregistered protocol osf.io/ktmdg."
Selection process	8	Specify the methods used to decide whether a study met the inclusion criteria of the review, including how many reviewers screened each record and each report retrieved, whether they worked independently, and if applicable, details of automation tools used in the process.	See manuscript sections *Eligibility criteria* and *Study selection*, as well as the included PRISMA flowchart.
Data collection	9	Specify the methods used to collect data from reports, including how many reviewers	See manuscript section *Study Selection*

For peer review only - http://bmjopen.bmj.com/site/about/guidelines.xhtml

PRISMA 2020 Checklist

Section and Topic	Item #	Checklist item	Location where item is reported
process		collected data from each report, whether they worked independently, any processes for obtaining or confirming data from study investigators, and if applicable, details of automation tools used in the process.	
Data items	10a	List and define all outcomes for which data were sought. Specify whether all results that were compatible with each outcome domain in each study were sought (e.g. for all measures, time points, analyses), and if not, the methods used to decide which results to collect.	"Each included article was independently coded by two of four reviewers (RTT, RC, OvdA, SW) using a coding form designed for this review. The form consisted of five sections that assessed (1) article characteristics, (2) study registration details, (3) 10 types of outcome discrepancies (listed in Table 2), (4) 10 types of non-outcome discrepancies (listed in Table 4), and (5) any additional descriptive or inferential statistics on discrepancies. The form details the operationalization of each variable we coded, and is available at osf.io/728ys. We chose items to code based on a pilot test of our protocol, as well as the categories used in a seminal paper [10] and a recent systematic review on discrepancies [7]."
	10b	List and define all other variables for which data were sought (e.g. participant and intervention characteristics, funding sources). Describe any assumptions made about any missing or unclear information.	"To be included in our meta-analyses this data had to include at least two of (1) the number of studies assessed (denominator), (2) the number of studies with a given discrepancy (numerator), and (3) the percentage of studies with a given discrepancy—form which we could calculate an unreported numerator or denominator. We did not access the raw data. If an article did not report data for a certain measure (e.g., secondary outcome discrepancies), then we did not include that article in the meta-analysis for that measure (this is why k varies among the meta-analyses we present —see Tables 2 and 4)."
Study risk of bias assessment	11	Specify the methods used to assess risk of bias in the included studies, including details of the tool(s) used, how many reviewers assessed each study and whether they worked independently, and if applicable, details of automation tools used in the process.	"Whereas we did not perform a formal risk of bias assessment—because our review differed substantially from the purpose these tools were built for—we shed light on a few potential sources of bias with additional analyses that consider the funding source, statistical significance, and the timing of registration of included studies. " … "Each included article was independently coded by two of four reviewers (RTT, RC, OvdA, SW) using a coding form designed for this review. The form is available at (osf.io/sc8p6/) and details the operationalization of each variable we coded."
Effect measures	12	Specify for each outcome the effect measure(s) (e.g. risk ratio, mean difference) used in the synthesis or presentation of results.	Included in all figures and tables.
Synthesis methods	13a	Describe the processes used to decide which studies were eligible for each synthesis (e.g. tabulating the study intervention characteristics and comparing against the planned groups for each synthesis (item #5)).	"We included articles that reported quantitative data about discrepancies between registrations or study protocols and their associated publication."
	13b	Describe any methods required to prepare the data for presentation or synthesis, such as handling of missing summary statistics, or data conversions.	"To be included in our meta-analyses this data had to include at least two of (1) the number of studies assessed (denominator), (2) the number of studies with a given discrepancy (numerator), and (3) the percentage of studies with a given discrepancy—form which we could calculate an unreported numerator or denominator. We did not access the raw data. If an article did not report data for a certain measure (e.g., secondary outcome discrepancies), then we did not include that article in the meta-analysis for that measure (this is why k varies among the meta-analyses we present —see Tables 2 and 4)."
	13c	Describe any methods used to tabulate or visually display results of individual studies and syntheses.	Data on individual studies is available in our dataset which is available as open data. We do not tabulate data from individual studies within the manuscript.

For peer review only

PRISMA 2020 Checklist

Section and Topic	Item #	Checklist item	Location where item is reported
	13d	Describe any methods used to synthesize results and provide a rationale for the choice(s). If meta-analysis was performed, describe the model(s), method(s) to identify the presence and extent of statistical heterogeneity, and software package(s) used.	See section *Statistical analyses*.
	13e	Describe any methods used to explore possible causes of heterogeneity among study results (e.g. subgroup analysis, meta-regression).	See section *Statistical analyses*.
	13f	Describe any sensitivity analyses conducted to assess robustness of the synthesized results.	See section *Statistical analyses*.
Reporting bias assessment	14	Describe any methods used to assess risk of bias due to missing results in a synthesis (arising from reporting biases).	"Whereas we did not perform a formal risk of bias assessment—because our review differed substantially from the purpose these tools were built for—we shed light on a few potential sources of bias with additional analyses that consider the funding source, statistical significance, and the timing of registration of included studies."
Certainty assessment	15	Describe any methods used to assess certainty (or confidence) in the body of evidence for an outcome.	We present prediction intervals, confidence intervals, and heterogeneity.
**RESULTS**			
Study selection	16a	Describe the results of the search and selection process, from the number of records identified in the search to the number of studies included in the review, ideally using a flow diagram.	See included PRISMA flowchart.
	16b	Cite studies that might appear to meet the inclusion criteria, but which were excluded, and explain why they were excluded.	We did not identify studies that meet this criteria. Also: "(the list of articles and coding is available at osf.io/wa62f)."
Study characteristics	17	Cite each included study and present its characteristics.	See section *Article characteristics*. All included articles are in the reference list and listed in the open data.
Risk of bias in studies	18	Present assessments of risk of bias for each included study.	"Whereas we did not perform a formal risk of bias assessment—because our review differed substantially from the purpose these tools were built for—we shed light on a few potential sources of bias with additional analyses that consider the funding source, statistical significance, and the timing of registration of included studies."
Results of individual studies	19	For all outcomes, present, for each study: (a) summary statistics for each group (where appropriate) and (b) an effect estimate and its precision (e.g. confidence/credible interval), ideally using structured tables or plots.	See forest plots which include confidence intervals and a prediction interval.
Results of	20a	For each synthesis, briefly summarise the characteristics and risk of bias among	See sections *Primary outcome discrepancies* and *Secondary outcome discrepancies.*

For peer review only - http://bmjopen.bmj.com/site/about/guidelines.xhtml

PRISMA 2020 Checklist

Section and Topic	Item #	Checklist item	Location where item is reported
syntheses		contributing studies.	
	20b	Present results of all statistical syntheses conducted. If meta-analysis was done, present for each the summary estimate and its precision (e.g. confidence/credible interval) and measures of statistical heterogeneity. If comparing groups, describe the direction of the effect.	See forest plots in main manuscript as well as Supplementary Materials.
	20c	Present results of all investigations of possible causes of heterogeneity among study results.	Meta-regression results are included in the sections *Primary outcome discrepancies* and *Secondary outcome discrepancies.*
	20d	Present results of all sensitivity analyses conducted to assess the robustness of the synthesized results.	"We also conducted a sensitivity analysis that included all six regressors in a single meta-regression and found that publications compared to the most recent version of a registration (k = 15) may have a smaller proportion of studies with at least one primary outcome discrepancy as compared to publications compared to the original version of a registration (k = 29) (p = 0.08; OR 95% CI: 0.32 to 1.07). All meta-regression model summaries are presented in Supplementary Material E."
Reporting biases	21	Present assessments of risk of bias due to missing results (arising from reporting biases) for each synthesis assessed.	We feel this item is not relevant to the subject matter of our systematic review.
Certainty of evidence	22	Present assessments of certainty (or confidence) in the body of evidence for each outcome assessed.	We present prediction intervals and confidence intervals throughout.
**DISCUSSION**			
Discussion	23a	Provide a general interpretation of the results in the context of other evidence.	"Our main findings are in line with those from previous systematic reviews. These reviews included 27 articles each and found that 31% of studies had a primary outcome discrepancy in the median article they reviewed (C. W. Jones et al., 2015) and 54% of studies had any outcome discrepancy in the median article they reviewed (G. Li et al., 2018). The latter review did not distinguish between primary and secondary outcomes, and many articles they reviewed only assessed primary outcomes. Our review included all the articles contained in these systematic reviews, except for a few that did not meet our inclusion criteria (e.g., a PhD thesis, an abstract)."
	23b	Discuss any limitations of the evidence included in the review.	"We find that outcome measures in registrations and study protocols often differ from published outcome measures, that the prevalence of discrepancies varies substantially across the articles we reviewed, and that this heterogeneity is not easily assigned to specific article characteristics. Given the wide range of discrepancy prevalence across individual articles, point estimates and confidence intervals may provide false precision when extrapolating our findings to the registered literature at large. Moreover, because heterogeneity could not be explained by meta-regression of article characteristics, more precise estimates cannot be derived for subsets of the literature. The prediction intervals can reasonably be used to extrapolate to the registered medical literature at large, although the included studies do not necessarily form a representative sample."<br>Also see *Limitations* section of the Discussion.
	23c	Discuss any limitations of the review processes used.	See response to previous box.
	23d	Discuss implications of the results for practice, policy, and future research.	See *Implications* section of the Discussion.

For peer review only - http://bmjopen.bmj.com/site/about/guidelines.xhtml

PRISMA 2020 Checklist

Section and Topic	Item #	Checklist item	Location where item is reported
**OTHER INFORMATION**			
Registration and protocol	24a	Provide registration information for the review, including register name and registration number, or state that the review was not registered.	"Registration. osf.io/ktmdg. Protocol amendments are listed in Supplementary Material A."
	24b	Indicate where the review protocol can be accessed, or state that a protocol was not prepared.	"Registration. osf.io/ktmdg. Protocol amendments are listed in Supplementary Material A."…"Each included article was independently coded by two of four reviewers (RTT, RC, OvdA, SW) using a coding form designed for this review. The form details the operationalization of each variable we coded, and is available at osf.io/728ys. The complete dataset, including the coding of each reviewer and the resolved coding, is available at osf.io/ue2c6. A cleaned dataset with only the resolved coding is available at osf.io/6cn9m."
	24c	Describe and explain any amendments to information provided at registration or in the protocol.	**"Registration**. osf.io/ktmdg. Protocol amendments are listed in Supplementary Material A."
Support	25	Describe sources of financial or non-financial support for the review, and the role of the funders or sponsors in the review.	See *Funding* section.
Competing interests	26	Declare any competing interests of review authors.	"None declared"
Availability of data, code and other materials	27	Report which of the following are publicly available and where they can be found: template data collection forms; data extracted from included studies; data used for all analyses; analytic code; any other materials used in the review.	All data and materials are openly shared on the Open Science Framework at www.osf.io/5gfty

From: Page MJ, McKenzie JE, Bossuyt PM, Boutron I, Hoffmann TC, Mulrow CD, et al. The PRISMA 2020 statement: an updated guideline for reporting systematic reviews. BMJ 2021;372:n71. doi: 10.1136/bmj.n71

For more information, visit: http://www.prisma-statement.org/

For peer review only

MOOSE (Meta-analyses Of Observational Studies in Epidemiology) Checklist

A reporting checklist for Authors, Editors, and Reviewers of Meta-analyses of Observational Studies. You must report the page number in your manuscript where you consider each of the items listed in this checklist. If you have not included this information, either revise your manuscript accordingly before submitting or note N/A.

Reporting Criteria	Reported (Yes/No)	Reported on Page No.
**Reporting of Background**		
Problem definition		
Hypothesis statement		
Description of Study Outcome(s)		
Type of exposure or intervention used		
Type of study design used		
Study population		
**Reporting of Search Strategy**		
Qualifications of searchers (eg, librarians and investigators)		
Search strategy, including time period included in the synthesis and keywords		
Effort to include all available studies, including contact with authors		
Databases and registries searched		
Search software used, name and version, including special features used (eg, explosion)		
Use of hand searching (eg, reference lists of obtained articles)		
List of citations located and those excluded, including justification		
Method for addressing articles published in languages other than English		
Method of handling abstracts and unpublished studies		
Description of any contact with authors		
**Reporting of Methods**		
Description of relevance or appropriateness of studies assembled for assessing the hypothesis to be tested		
Rationale for the selection and coding of data (eg, sound clinical principles or convenience)		
Documentation of how data were classified and coded (eg, multiple raters, blinding, and interrater reliability)		
Assessment of confounding (eg, comparability of cases and controls in studies where appropriate		

For peer review only

Reporting Criteria	Reported (Yes/No)	Reported on Page No.
Assessment of study quality, including blinding of quality assessors; stratification or regression on possible predictors of study results		
Assessment of heterogeneity		
Description of statistical methods (eg, complete description of fixed or random effects models, justification of whether the chosen models account for predictors of study results, dose-response models, or cumulative meta-analysis) in sufficient detail to be replicated		
Provision of appropriate tables and graphics		
**Reporting of Results**		
Table giving descriptive information for each study included		
Results of sensitivity testing (eg, subgroup analysis)		
Indication of statistical uncertainty of findings		
**Reporting of Discussion**		
Quantitative assessment of bias (eg, publication bias)		
Justification for exclusion (eg, exclusion of non–English-language citations)		
Assessment of quality of included studies		
**Reporting of Conclusions**		
Consideration of alternative explanations for observed results		
Generalization of the conclusions (ie, appropriate for the data presented and within the domain of the literature review)		
Guidelines for future research		
Disclosure of funding source		

Once you have completed this checklist, please save a copy and upload it as part of your submission. DO NOT include this checklist as part of the main manuscript document. It must be uploaded as a separate file.

For peer review only

Estimating the prevalence of discrepancies between study registrations and publications: A systematic review and meta-analyses

Journal:	*BMJ Open*
Manuscript ID	bmjopen-2023-076264.R1
Article Type:	Original research
Date Submitted by the Author:	27-Jun-2023

Complete List of Authors:	Thibault, Robert; Stanford University, Meta-Research Innovation Center at Stanford (METRICS); University of Bristol, School of Psychological Science<br>Clark, Robbie; University of Bristol, School of Psychological Science; MRC Integrative Epidemiology Unit at the University of Bristol<br>Pedder, Hugo ; University of Bristol, Population Health Sciences; University of Bristol, Bristol Medical School<br>van den Akker, Olmo; Tilburg University, Department of Methodology & Statistics; Berlin Institute of Health at Charité, QUEST Center for Responsible Research<br>Westwood, Samuel; King's College London, Department of Psychology, Institute of Psychiatry, Psychology, Neuroscience<br>Thompson, Jacqueline; University of Bristol; University of Oxford, Bodleian Libraries<br>Munafo, Marcus; University of Bristol, School of Psychological Science; MRC Integrative Epidemiology Unit at the University of Bristol

**Primary Subject Heading**:	Research methods
Secondary Subject Heading:	Medical publishing and peer review
Keywords:	STATISTICS & RESEARCH METHODS, Clinical Trial, REGISTRIES

SCHOLARONE™ Manuscripts

I, the Submitting Author has the right to grant and does grant on behalf of all authors of the Work (as defined in the below author licence), an exclusive licence and/or a non-exclusive licence for contributions from authors who are: i) UK Crown employees; ii) where BMJ has agreed a CC-BY licence shall apply, and/or iii) in accordance with the terms applicable for US Federal Government officers or employees acting as part of their official duties; on a worldwide, perpetual, irrevocable, royalty-free basis to BMJ Publishing Group Ltd ("BMJ") its licensees and where the relevant Journal is co-owned by BMJ to the co-owners of the Journal, to publish the Work in this journal and any other BMJ products and to exploit all rights, as set out in our licence.

The Submitting Author accepts and understands that any supply made under these terms is made by BMJ to the Submitting Author unless you are acting as an employee on behalf of your employer or a postgraduate student of an affiliated institution which is paying any applicable article publishing charge ("APC") for Open Access articles. Where the Submitting Author wishes to make the Work available on an Open Access basis (and intends to pay the relevant APC), the terms of reuse of such Open Access shall be governed by a Creative Commons licence – details of these licences and which Creative Commons licence will apply to this Work are set out in our licence referred to above.

Other than as permitted in any relevant BMJ Author's Self Archiving Policies, I confirm this Work has not been accepted for publication elsewhere, is not being considered for publication elsewhere and does not duplicate material already published. I confirm all authors consent to publication of this Work and authorise the granting of this licence.

For peer review only

Title page as per BMJ Open requirements.

Estimating the prevalence of discrepancies between study registrations and publications: A systematic review and meta-analyses

We are submitting this manuscript under the name of a single group/consortium authorship: "TARG Meta-Research Group & Collaborators".

This manuscript includes several contributors as stated in the contributorship statement before the references. The contributors include:

Robert T. Thibault[1,2†], Robbie Clark[2,3], Hugo Pedder[4,5], Olmo van den Akker[6,7], Samuel Westwood[8], Jacqueline M. Thompson[2,9], Marcus R. Munafò[2,3]

[1]Meta-Research Innovation Center at Stanford (METRICS), Stanford University

[2]School of Psychological Science, University of Bristol

[3]MRC Integrative Epidemiology Unit at the University of Bristol

[4]Population Health Sciences, University of Bristol

[5]Bristol Medical School, University of Bristol

[6]Department of Methodology & Statistics, Tilburg University

[7]QUEST Center for Responsible Research, Berlin Institute of Health

[8]Department of Psychology, Institute of Psychiatry, Psychology, Neuroscience, King's College London

[9]Bodleian Libraries, University of Oxford

*Corresponding contributor. 12a Priory Road, BRISTOL BS8 1TU, United Kingdom, robert.thibault@stanford.edu, telephone: +44 117 33 17902, fax: NA

Keywords: registration; selective reporting; outcome switching; meta-research; reproducibility

Word count: 3748

Estimating the prevalence of discrepancies between study registrations and publications: A systematic review and meta-analyses

TARG Meta-Research Group & Collaborators

Abstract

Objectives. Prospectively registering study plans in a permanent time-stamped and publicly accessible document is becoming more common across disciplines and aims to reduce risk of bias and make risk of bias transparent. Selective reporting persists, however, when researchers deviate from their registered plans without disclosure. This systematic review aimed to estimate the prevalence of undisclosed discrepancies between prospectively registered study plans and their associated publication. We further aimed to identify the research disciplines where these discrepancies have been observed, whether interventions to reduce discrepancies have been conducted, and gaps in the literature.

Design. Systematic review and meta-analyses.

Data sources. Scopus and Web of Knowledge, published up to 15 December 2019.

Eligibility criteria. Articles that included quantitative data about discrepancies between registrations or study protocols and their associated publications.

Data extraction and synthesis. Each included article was independently coded by two reviewers using a coding form designed for this review (osf.io/728ys). We used random-effects meta-analyses to synthesize the results.

Results. We reviewed k = 89 articles, which included k = 70 that reported on primary outcome discrepancies from n = 6314 studies and, k = 22 that reported on secondary outcome discrepancies from n = 1436 studies. Meta-analyses indicated that between 29% to 37% (95% confidence interval) of studies contained at least one primary outcome discrepancy and between 50% to 75% (95% confidence interval) contained at least one secondary outcome discrepancy. Almost all articles assessed clinical literature, and there was considerable heterogeneity. We identified only one article that attempted to correct discrepancies.

Conclusions. Many articles did not include information on whether discrepancies were disclosed, which version of a registration they compared publications to, and whether the registration was prospective. Thus, our estimates represent discrepancies broadly, rather than our target of *undisclosed* discrepancies between *prospectively* registered study plans and their associated publications. Discrepancies are common and reduce the trustworthiness of medical research. Interventions to reduce discrepancies could prove valuable.

Registration. osf.io/ktmdg. Protocol amendments are listed in Supplementary Material A.

For peer review only

Strengths and limitations of this study.

- We employ a wide-reaching search strategy and captured 89 articles including over 6,000 registrations and publications.
- Our coding procedure includes fine-grained information that allowed us to run meta-regressions and test whether several parameters impact discrepancies.
- All our data and code are openly available.
- The high heterogeneity in the meta-analyses led to wide-ranging confidence intervals and prediction intervals.
- Many articles did not fully operationalize their definition of what constitutes a discrepancy (e.g., which version of the registration was used).

For peer review only

Introduction

In 2000, ClinicalTrials.gov and the ISRCTN Registry were launched with several aims, including aiding participant recruitment, facilitating knowledge synthesis, and reducing duplication, publication bias and selective reporting [1]. In 2005, the International Committee of Medical Journal Editors (ICMJE) made *prospective* registration a condition of consideration for publication [2]. Thousands of journals now claim to follow this policy [3]. In parallel, the World Health Organization International Clinical Trials Registry Platform established a minimum set of required information for a trial to be considered fully registered, including experimental design elements such as the conditions being studied, intervention, key inclusion and exclusion criteria, sample size, primary outcomes, and key secondary outcomes [4]. While the relatively widespread uptake of clinical trial registration has substantially improved transparency, many trials remain unregistered, are registered after enrollment of participants begins or analyses are complete (i.e., *retrospective* registration), are never published, or publish outcomes discrepant with those in the registration without disclosing the discrepancy [e.g., 5,6]. Nevertheless, the existence of registries allows researchers to identify and quantify these issues.

In research disciplines other than clinical trials, study registration is becoming more common, but remains far from standard practice [e.g., 7-10]. For example, starting around 2011 the field of psychology has increasingly taken the "replication crisis" seriously and many researchers and journals now use registration to reduce bias and make risk of bias transparent. Other disciplines have created dedicated registries, such as PROSPERO for systematic reviews and the American Economic Association RCT Trial Registry.

In this manuscript, we systematically reviewed articles that quantify the prevalence of discrepancies between registrations or study protocols and their associated publications (e.g., in primary outcome measures). Our analysis extended beyond the three systematic reviews already published on this topic in several ways [11-13]. First, registration has expanded beyond clinical trials; we included all research disciplines and used key word searches for registries including the Open Science Framework, the American Economic Association RCT Trial Registry, and PROSPERO. Second, we extracted more fine-grained information about a wide range of discrepancies (e.g., outcomes, analysis, sample size), as well as which version of the registration was surveyed and whether discrepancies were disclosed (we believe disclosed discrepancies present little reason for concern). Third, our review included over twice as many studies as previous systematic reviews on this topic, provides meta-analytic estimates, and used meta-regression and additional analyses to attempt to identify predictors of discrepancies.

Methods

Terminology

We present a systematic review of k = 89 articles that assessed a wide range of outcome discrepancies and non-outcome discrepancies across over n = 7,000 studies. To avoid confusion, this report consistently uses the terms *studies* to refer to the over n = 7,000

For peer review only

individual studies that were *assessed*, and the term *article* to refer to the k = 89 articles that assessed these studies, and that we *reviewed*. We restrict our usage of the term *publication* to refer to the publications stemming from the *studies* (not to refer to the *articles*).

We use the term *discrepancy* to refer to any incongruity between the content of a publication and its associated registration (e.g., on clinicaltrials.gov) or study protocol (e.g., submitted to an ethics review board or funding agency)—see Box 1 for examples. We use the term *prospective registration* broadly to include terms used in different research disciplines, such as prospective trial registration, preregistration, and pre-analysis plans. All these terms indicate the registration of study details *before* commencing a study, or in some cases, before viewing the data or removing the blind. They are in contrast to *retrospective* registration, which occurs after participant enrollment begins or analyses are complete. We use the term *outcome discrepancy* to indicate a discrepancy in the outcome measure registered versus the outcome measure reported in a publication (not to indicate a discrepancy in the value of a reported outcome between these documents).

Box 1. Examples of discrepancies.

We coded 10 types of outcome discrepancies (listed in Table 2) and 10 types of non-outcome discrepancies (listed in Table 4). The degree to which the information in a registration is discrepant with the information in a publication can range widely[1]. The associated concern about risk of bias can also range widely and often requires domain expertise to assess. We present two examples from [14] in this box. Many more examples are available in the appendix of [14,15] and at https://www.compare-trials.org/results.

Registered: Primary outcome: Visual Acuity [Time Frame: 6 months].

Published: Primary outcome: 3-year cumulative incidence rate of myopia. Myopia was defined as a spherical equivalent refractive error (sphere +½ cylinder) of at least −0.50 D

Coded as: Timing of outcome measurement changed.

Registered: Primary outcome: Severity of Device and Procedure related complications [Time Frame: At the time of ExAblate Transcranial thalamotomy procedure]. Secondary outcome: Effectiveness of the ExAblate Transcranial MRgFUS treatment determined using the Clinical Rating Scale for Tremor (CRST) [Time Frame: Participants will be followed from the date of treatment until study completion, approximately up to 12 months].

Published: Primary outcome: Change from baseline to 3 months in the tremor score for the hand derived from the CRST, Part A (three items: resting, postural, and action or intention components of hand tremor), and the CRST, Part B (five tasks involving handwriting, drawing, and pouring).

Coded as: Secondary outcome promoted to primary outcome; Timing of outcome measurement changed; Primary outcome omitted.

before starting a study, but after registering). Study protocols contain timestamps, yet they often become public only at the time of publication.

For peer review only - http://bmjopen.bmj.com/site/about/guidelines.xhtml

Eligibility criteria

We included articles that reported quantitative data about discrepancies between registrations or study protocols and their associated publication. We excluded conference proceedings and articles written in a language other than English (for full inclusion and exclusion criteria, see our preregistered protocol at osf.io/ktmdg).

Study selection

We searched Scopus and Web of Science on 15 December 2019 using the queries in Appendix A and Appendix B of our preregistered protocol (osf.io/ktmdg). Briefly, our queries included (1) variations of the terms preregistration, pre-analysis plans, and prospective registration in the title or keyword fields; (2) terms indicating discrepancies such as "outcome switching" in the title, keywords or abstract; (3) names of registration or protocol repositories such as "clinicaltrials.gov" in the title or keywords; and excluded overlapping but irrelevant terms (e.g., "nursing preregistration"). To limit the number of irrelevant articles, we did not search for variations of the term preregistration or for repository names in the abstract field.

Our search returned 4,283 articles after duplicates were removed (see Figure 1 for a PRISMA flowchart). Articles were screened independently by two reviewers in two stages. In Stage One, reviewers screened titles and, if necessary, briefly examined abstracts of articles to determine inclusion in the systematic review or in a scoping review on prospective registration[2]. If at least one of the reviewers deemed an article potentially relevant, it was included in Stage Two screening. In Stage Two, the reviewers independently examined the remaining 464 abstracts in greater detail for eligibility. Disagreements were resolved through discussion between the two reviewers and eventual consensus. Inter-rater agreement for the 464 articles was Cohen's k = 0.67 for inclusion in the systematic review (the list of articles and coding is available at osf.io/wa62f). Inter-rater agreement for all 4,283 articles was Cohen's k = 0.72. We then used a snowball method and identified 33 additional articles that met our inclusion criteria, mostly through citations in [11] and [13]. These 33 additional articles are not included in the inter-rater agreement scores. After full-text review, we included 89 articles in our systematic review.

****Insert Figure 1 around here****

Coding items

Each included article was independently coded by two of four reviewers (RTT, RC, OvdA, SW) using a coding form designed for this review. The form consisted of five sections that assessed (1) article characteristics, (2) study registration details, (3) 10 types of outcome discrepancies (listed in Table 2), (4) 10 types of non-outcome discrepancies (listed in Table 4), and (5) any additional descriptive or inferential statistics on discrepancies. The form

[2] We had planned to report on this scoping review separately. However, the scoping review has been terminated (details provided at https://osf.io/ktmdg).

For peer review only - http://bmjopen.bmj.com/site/about/guidelines.xhtml

details the operationalization of each variable we coded, and is available at osf.io/728ys. We chose items to code based on a pilot test of our protocol, as well as the categories used in a seminal paper [16] and a systematic review on discrepancies [11].

The data we extracted was often presented as summary results in a table and sometimes as text in the results section. To be included in our meta-analyses this data had to include at least two of (1) the number of studies assessed (denominator), (2) the number of studies with a given discrepancy (numerator), and (3) the percentage of studies with a given discrepancy—from which we could calculate an unreported numerator or denominator. We did not access the raw data. If an article did not report data for a certain measure (e.g., secondary outcome discrepancies), then we did not include that article in the meta-analysis for that measure (this is why k varies among the meta-analyses we present—see Tables 2 and 4). For the meta-regressions we performed, we could not find data on (1) the version of the registry that publications were compared to for 35 articles, and (2) the number of studies that disclosed discrepancies for 57 articles. We coded these cases as "not reported" and included "not reported" as a factor in the meta-regressions. All other meta-regression data was complete. Further coding details are available in Supplementary Material B.

The complete dataset, including the coding of each reviewer and the resolved coding, is available at osf.io/ue2c6. A cleaned dataset with only the resolved coding is available at osf.io/6cn9m.

Statistical analyses

We performed two main random effects meta-analyses: one on the proportion of studies with at least one primary outcome discrepancy, and another on the proportion of studies with at least one secondary outcome discrepancy. We used random effects models because they allow for the true effect to vary across the populations the articles sampled from, and the articles we reviewed differ in their methodologies and the research disciplines that they assess. We used a random intercept logistic regression model with the Knapp-Hartung adjustment for the synthesis of proportions [17]. We used the maximum-likelihood method for estimating the between-study heterogeneity (Tau). We also performed meta-regressions to test whether article characteristics are associated with the proportion of studies with at least one primary or secondary outcome discrepancy.

For pooled estimates, we report both confidence intervals and prediction intervals. Whereas researchers are likely more familiar with confidence intervals, interpreting confidence intervals can be unintuitive [18], and their pooled-estimate does not incorporate uncertainty due to the between-article heterogeneity. If we assume that we could resample from our population, 95% of the resampled *meta-analyses* would yield a 95% confidence interval that contains the true value of the parameter being estimated (e.g., proportion of articles with at least one primary outcome discrepancy). Alternatively, if we are interested in the results that would come from another *article* assessing discrepancies, we would want a 95% prediction interval. In other words, of 100 articles drawn from the same population, we could expect the results from 95 of them—on average—to fall within the 95% prediction interval. While prediction intervals are not commonly reported, methodologists recommend reporting

For peer review only

them for random effects meta-analysis, particularly when few articles are included or, as in our case, included articles are highly heterogeneous [19,20].

Whereas we did not perform a formal risk of bias assessment—because our review differed substantially from the purpose these tools were built for—we shed light on a few potential sources of bias with additional analyses that consider the funding source, statistical significance, and the timing of registration of included *studies*. These additional analyses were not prospectively registered. We made a few amendments to our preregistered study protocol which are listed in Supplementary Material A.

Patient and Public Involvement

Patients and members of the public were not involved in the design, conduct or reporting of this systematic review.

Results

Articles characteristics

We identified and reviewed k = 89 articles that report at least one type of discrepancy. Articles that checked for outcome discrepancies assessed a median of 68 studies (IQR: 33 to 112). Article characteristics are outlined in Table 1. All articles except for two, one preprint in economics [21] and one preprint in psychology [22], focused on clinical trials or systematic reviews. All but k = 10 articles were solely observational. Only one article attempted to correct published discrepancies [23]. The authors assessed all trials published in five journals over a six-week period and sent a letter to the editor for each trial that published a discrepant outcome (for 58 letters in total). In the time since our literature search, at least two more interventional studies were published. One reports a trial that attempted to reduce discrepancies at medical journals by sending peer reviewers information about the study registration [24]. They found null results. The other was a feasibility study that assigned a peer reviewer to specifically check for discrepancies in manuscripts submitted for publication [25]. Further details about article characteristics are available in Supplementary Material C.

Table 1. Article characteristics

**Article characteristic**	***k* = 89**	***n* = 6929**
**Discipline**		
medicine	81	6452
dentistry	3	254
psychology	3	68
economics	1	93
physical therapy	1	62
**Source of registration or protocol assessed for discrepancies**		
registry	73	6107

For peer review only

Table 1. Article characteristics

**Article characteristic**	***k* = 89**	***n* = 6929**
ethics application	7	146
other protocol	5	466
marketing application	2	126
grant application	2	84
**Sources searched to identify studies**		
journals	32	3264
registries	23	1547
search engines	16	1527
ethics boards	7	146
funders	3	96
registries and search engines	3	27
regulators	2	126
research group	2	44
registries and journals	1	152
**Type of study**		
solely observational	79	6344
observational and study authors were contacted	9	518
observational and interventional	1	67
**Version of registry that publications were compared to**		
original	29	2607
most recent	15	1281
other version/unclear	10	670
not reported	35	2371
**Number of studies within each article that disclosed discrepancies**		
one or more	19	1547
none	9	441
article excluded publications with disclosed discrepancies	4	326
not reported	57	4615

Registration timing

Articles varied in the level of detail they provided about whether and when studies were registered. For example, whereas some articles presented their sample only after selecting for prospectively registered studies, other articles detailed their selection process including how many studies were registered and if so, when they were registered. Using the terminology in the articles we reviewed, articles identified studies that were registered retrospectively (k = 29), registered during participant enrollment (k = 17), registered after

For peer review only

participant enrollment was complete (k = 14), and studies that were not registered (k = 36)[3]. Many articles were ambiguous regarding when some studies were registered (k = 47) and whether or not some studies were registered at all (k = 24). While these data do not provide fine-grained detail, they highlight two overarching issues: many studies are not registered, and many registered studies are registered retrospectively. These studies fail to meet the Declaration of Helsinki [26] (item 35) requirement that "Every research study involving human subjects must be registered in a publicly accessible database before recruitment of the first subject" and the equivalent International Committee of Medical Journal Editors (ICMJE) policy [27], which thousands of journals claim to follow [3].

Eighty of the k = 89 articles we reviewed report at least one type of outcome discrepancy. Of these, 23 report only on studies that were unambiguously prospectively registered, 51 do not unambiguously distinguish between prospectively and retrospectively registered studies, and 6 report outcome discrepancies separately for each of prospectively and retrospectively registered studies. Separate meta-analyses for unambiguously prospectively registered studies and studies with unclear timing of registration are presented in Supplementary Material D.

Forty-six of the k = 89 articles report at least one non-outcome discrepancy (e.g., in sample size or analyses). Of these, 12 report only on studies that were unambiguously prospectively registered, 33 do not unambiguously distinguish between prospectively and retrospectively registered studies, and one reports non-outcome discrepancies separately for each of prospectively and retrospectively registered studies.

Primary outcome discrepancies

An estimated 29-37% (95% confidence interval) of the population of studies contained at least one primary outcome discrepancy (Figure 2). The 95% prediction interval is 10-68%.

****Insert Figure 2 around here****

This meta-analysis had high heterogeneity (I^2 = 86%), suggesting that the broad range of estimates across the articles stem largely from differences in the methodology of the articles or populations they sample from, rather than from chance. Heterogeneity could not be explained by meta-regression of any of the following article-level characteristics: discipline (p = 0.28)[4], whether the publications were compared to registry entries versus other protocol formats (e.g., ethics applications) (p = 0.46), sources searched to identify studies (p = 0.65), version of the registry analyzed (p = 0.77), whether discrepancies were disclosed (p = 0.97), and year of article publication (p = 0.83). The meta-regression on discipline had low

[3] By definition, non-registered studies cannot be assessed for discrepancies between their registration and publication. Several articles identified non-registered studies in their sampling process, but did not include these studies in their final sample.

[4] If readers want to know if a study on discrepancies exists within a particular sub-discipline, we invite them to explore the variable 'disciplineDetail' in our dataset.

For peer review only - http://bmjopen.bmj.com/site/about/guidelines.xhtml

power because 63 articles assessed medical research and 7 assessed studies across dentistry, psychology, physical therapy, and economics. To increase statistical power, we reran this meta-regression after dichotomizing discipline and found that non-medical disciplines may have a greater proportion of studies with at least one primary outcome discrepancy (p = 0.09; OR 95% CI: 0.91-3.19). We ran another meta-regression after dichotomizing the source which publications were compared to—into registrations versus other protocols—and did not find evidence to suggest this moderator played a role (p = 0.42). We also conducted a sensitivity analysis that included all six article-level characteristics in a single meta-regression. We found that publications compared to the most recent version of a registration may have a smaller proportion of studies with at least one primary outcome discrepancy, relative to publications that were compared to the original version of a registration (p = 0.08; OR 95% CI: 0.32 to 1.07). All meta-regression model summaries are presented in Supplementary Material E.

The high heterogeneity in this meta-analysis may stem from genuine differences among the articles, including the sub-disciplines surveyed, specific sources searched, definition of a discrepancy (e.g., whereas some articles considered a change in the timing of an outcome as a discrepancy, others did not), and other article characteristics that may or may not have been reported. Our dataset contains more fine-grained information about the specific sub-discipline surveyed and specific sources searched. While we do not further explore these potential moderators in the present report, we note that, whereas some sub-disciplines and sources were highly specific (e.g., cystic fibrosis, lung cancer immunotherapy, Global Resource of Eczema Trials database), others were broad (e.g., medicine, clinicaltrials.gov, core clinical MEDLINE journals). We did not collect information on the exact definitions an article used to identify a primary outcome discrepancy. However, we did collect information on the proportion of articles with sub-categories of outcome discrepancies, which are more strictly defined and listed in Table 2 (e.g., promoting a secondary outcome to a primary outcome). We ran meta-analyses on these sub-categories of outcome discrepancies and found they also had high heterogeneity (Table 2). Thus, varying definitions are unlikely to be the main driver of the high heterogeneity in the present analysis on primary outcome discrepancies.

Secondary outcome discrepancies

An estimated 50-75% (95% confidence interval) of the population of studies contained at least one secondary outcome discrepancy (Figure 3). The 95% prediction interval is 13-95% .

****Insert Figure 3 around here****

This meta-analysis also had high heterogeneity (I^2 = 90%) which could not be explained by meta-regression of the version of the registry analyzed (p = 0.8) or the year of article publication (p = 0.72). Meta-regression of the sources searched to identify studies explained some heterogeneity, in that searches stemming from journals, compared to registries, had a greater proportion of publications with at least one secondary outcome discrepancy (p = 0.03; OR 95% CI: 1.89-13.45). Meta-regressions on discipline (p = 0.29), whether discrepancies were disclosed (p = 0.68), and whether the publications were compared to

For peer review only - http://bmjopen.bmj.com/site/about/guidelines.xhtml

registry entries versus other protocol formats (p = 0.08) had very low statistical power because almost all articles had the same characteristic. All meta-regression model summaries are included in Supplementary Material E.

Descriptively, omitting secondary outcomes and adding secondary outcomes appears to occur more frequently than omitting primary outcomes, adding primary outcomes, or demoting primary outcomes, which in turn appear to occur more frequently than promoting a secondary outcome (see Table 2).

Table 2. Meta-analytic estimates for the proportion of studies that contain various types of outcome discrepancies

	**Discrepant studies (95% CI)**	**Discrepant studies (95% PI)**	**k**	**n**
Any outcome discrepancy	41-75%	7-97%	18	1113
Any primary outcome discrepancy	29-37%	10-68%	70	6314
Any secondary outcome discrepancy	50-75%	13-95%	22	1436
Primary outcome demoted to secondary outcome	6-10%	2-31%	51	4560
Primary outcome omitted	6-12%	1-43%	51	4338
Primary outcome added	7-11%	2-34%	54	4697
Secondary outcome promoted to primary outcome	4-6%	1-19%	46	4135
Secondary outcome omitted	16-35%	4-72%	18	1243
Secondary outcome added	19-43%	3-83%	20	1305
Timing of outcome measurement changed	6-16%	1-62%	30	3472

We only coded 'any outcome discrepancy' for articles that checked for both primary and secondary outcome discrepancies in the studies they assessed. Forest plots for all meta-analyses in this table are in Supplementary Material F.

For peer review only

Parameters potentially related to outcome discrepancies

A subset of articles contained information on parameters potentially related to the proportion of outcome discrepancies. These include the disclosure of discrepancies, presence of a 'statistically significant' result, funding source, and timing of registration (Table 3).

Table 3. Additional analyses regarding discrepancies

Analysis	95% CI	95% PI	k	n
Percentage of studies with at least one outcome discrepancy that disclose an outcome discrepancy	4-19%	0.3-74%	21	620
Percentage of outcome discrepancies that favored statistically significant results	49-66%	23-86%	24	671
Likelihood ratio of a study with versus without an outcome discrepancy to contain statistically significant results	0.56-1.06	0.42-1.43	7	405
*Likelihood ratio of a study with versus without a statistically significant results to contain an outcome discrepancy	0.64-0.99	0.59-1.06	7	405
Likelihood ratio of a study with versus without industry funded to contain an outcome discrepancy	0.61-0.91	0.44-1.27	22	2623
Likelihood ratio of a prospectively registered study versus retrospectively registered study to contain a primary outcome discrepancy	0.46-2.57	0.13-8.85	4	260

* This analysis uses the same data as the likelihood ratio analysis before it. One of these seven articles checked discrepancies in analyses, rather than discrepancies in outcome measures. Forest plots for all meta-analyses in this table are in Supplementary Material G.

Non-outcome discrepancies

The meta-analyses for the non-outcome discrepancies had high heterogeneity, and wide confidence intervals and prediction intervals (Table 4). Articles varied in the criteria they used to identify non-outcome discrepancies and there were fewer articles than for outcome discrepancies. Prediction intervals can be particularly imprecise when few articles are included in a meta-analysis [28]. Whereas our coding procedure divided outcome discrepancies into 10 sub-categories, it did not employ the same level of granularity for non-outcome discrepancies. Supplementary Material H contains additional information on non-outcome discrepancies.

For peer review only - http://bmjopen.bmj.com/site/about/guidelines.xhtml

Table 4. Meta-analytic estimates for the proportion of studies that contain various types of non-outcome discrepancies

	Discrepant studies (95% CI)	Discrepant studies (95% PI)	k	n
Eligibility criteria	25-57%	5-90%	15	1153
Sample size	26-44%	8-78%	25	1398
Randomization	2-64%	0.06-98%	5	176
Blinding	5-42%	0.04-99%	3	224
Intervention	3-52%	0.1-97%	7	550
Study duration	3-89%	0.02-99.94%	4	184
Analysis	19-52%	4-86%	12	404
Subgroup analysis	35-93%	2-99.7%	9	545
Funding	7-84%	0.2-99.5%	5	212
Results	7-82%	0.2-99%	6	262

Values less than 0.5% and greater than 99.5% are rounded to one significant digit from 0 or 100. Forest plots for all meta-analyses in this table are in Supplementary Material I. These results are best interpreted alongside the information provided in Supplementary Material H.

Gaps in the literature

We identified several gaps in the literature on discrepancies. There exists little research on: (1) the prevalence of discrepancies in fields other than clinical research, (2) the prevalence of discrepancies in a representative sample across clinical disciplines, (3) the level of specificity in registrations, and (4) interventions to reduce undisclosed discrepancies (see Supplementary Material J for additional information about these gaps). We also identified several themes from surveying the conclusions of the articles we reviewed. These include the need for awareness surrounding discrepancies, the need for mandates, enforcement, and/or new initiatives to address discrepancies, and the benefit of registering additional information such as analysis plans (Supplementary Table J1 contains additional details).

For peer review only

Discussion

We find that outcome measures in registrations and study protocols often differ from published outcome measures, that these discrepancies are rarely disclosed, that the prevalence of discrepancies varies substantially across the articles we reviewed, and that this heterogeneity is not easily assigned to specific article characteristics.

Limitations

Given the wide range of discrepancy prevalence across individual articles, point estimates and confidence intervals may provide false precision when extrapolating our findings to the registered literature at large. Moreover, because heterogeneity could not be explained by meta-regression of article characteristics, more precise estimates cannot be derived for subsets of the literature. The prediction intervals can reasonably be used to extrapolate to another article in the registered medical literature at large, although the included studies do not necessarily form a representative sample.

Comparison to previous research

Our main findings are in line with those from previous systematic reviews. These reviews included 27 articles each and found that 31% of studies had a primary outcome discrepancy in the median article they reviewed [13] and 54% of studies had any outcome discrepancy in the median article they reviewed [11]. The latter review did not distinguish between primary and secondary outcomes, and many articles they reviewed only assessed primary outcomes. Our review included all the articles contained in these systematic reviews, except for a few that did not meet our inclusion criteria (e.g., a PhD thesis, an abstract).

Implications

Our review raises broader issues regarding the efficiency of the research ecosystem and the trustworthiness of research outputs. We identified articles that documented discrepancies between publications and all of registrations, protocols, ethics applications, funding applications, and marketing approval applications. The existence of multiple documents outlining the same study raises the likelihood of discrepancies and, in the absence of a clearly demarcated 'master' document, leaves ambiguity regarding which document is 'correct'. Rehashing the same study details for different audiences may also be an inefficient use of researchers' time. Identifying a single publicly accessible document as the version of record (this could be the registration) and having all other documents point to this version of record for key information could reduce ambiguity and improve efficiency.

As for trustworthiness, registration has had a clearly positive influence on medical research [29, 30]. At the same time, some registration policies have poor adherence (e.g., many trials are registered retrospectively, and many trial results are never reported [31]). The existence of research policies that are regularly overlooked, rarely monitored, and come with no consequence for non-compliance, can be damaging in at least two ways. They risk devaluing research policies altogether and they can reduce the trustworthiness of research outputs by creating a false impression that rigorous research practices were employed. Conceiving

For peer review only - http://bmjopen.bmj.com/site/about/guidelines.xhtml

research as a complex ecosystem comprised of various agents with diverse incentives (e.g., funders, publishers, institutions, individual researchers) can help to comprehend why some policies have poor adherence and to develop and implement effective research infrastructure.

Conclusion

Registrations provide the evidence to detect selective reporting and outcome switching, which we found to be common. Nearly all articles we reviewed focused on documenting issues. Future efforts regarding discrepancies—and research improvement broadly—could prove more fruitful by shifting focus towards developing and testing solutions to these now well-documented issues.

Supplementary material

osf.io/byqhp

Funding

Robert Thibault was supported by a general support grant awarded to METRICS from Arnold Ventures and postdoctoral fellowships from the Canadian Institutes of Health Research (CIHR) and the Fonds de recherche du Québec - Santé (FRQS). Hugo Pedder was supported by funding from the National Institute of Health and Care Excellence's Guidelines Technical Support Unit and Bristol's National Institute for Health and Care Research Technology Assessment Group. Olmo van den Akker was supported by Consolidator Grant (IMPROVE) from the European Research Council (ERC; grant no. 726361). Marcus Munafò, Robert Thibault, Jacqueline Thompson, and Robbie Clark were part of the MRC Integrative Epidemiology Unit (MC_UU_00011/7). The funders had no role in study design, data collection and analysis, decision to publish, or preparation of the manuscript.

Transparency statement

Robert Thibault, the manuscript's guarantor, affirms that the manuscript is an honest, accurate, and transparent account of the study being reported; that no important aspects of the study have been omitted; and that any discrepancies from the study as originally planned have been explained.

Data Availability

Data, data dictionaries, analysis script and materials related to this study are publicly available on the Open Science Framework at https://osf.io/5gfty/. The study protocol and materials were registered on 17 January 2020 at https://osf.io/ktmdg. Discrepancies between this manuscript and the registered protocol are outlined in the Supplementary

For peer review only

Material A. To facilitate reproducibility, this manuscript was written by interleaving regular prose and analysis code using R Markdown. The relevant files are available in a Code Ocean container (https://doi.org/10.24433/CO.4753181.v1) which recreates the software environment in which the original analyses were performed. This container allows this manuscript to be reproduced from the data and code with a single button press.

Patient and public involvement

No patient involved.

Competing interests

None declared.

Contributors

Conceptualization: Robert T. Thibault, Jacqueline M. Thompson and Marcus R. Munafò.
Data curation: Robert T. Thibault.
Formal analysis: Robert T. Thibault and Hugo Pedder.
Funding acquisition: Robert T. Thibault.
Investigation: Robert T. Thibault, Robbie Clark, Olmo van den Akker, and Samuel Westwood.
Methodology: Robert T. Thibault, Robbie Clark, and Marcus R. Munafò.
Project administration: Robert T. Thibault.
Software: Robert T. Thibault and Hugo Pedder.
Supervision: Robert T. Thibault and Marcus R. Munafò.
Validation: Robert T. Thibault.
Visualization: Robert T. Thibault.
Writing - original draft: Robert T. Thibault.
Writing - review & editing: Robert T. Thibault, Robbie Clark, Hugo Pedder, Olmo van den Akker, Samuel Westwood, Jacqueline M. Thompson, and Marcus R. Munafò.

OR

Robert T. Thibault: Conceptualization, Data curation, Formal analysis, Funding acquisition, Investigation, Methodology, Project administration, Software, Supervision, Validation, Visualization, Writing - original draft, and Writing - review & editing. Robbie Clark: Investigation, Methodology, and Writing - review & editing. Hugo Pedder: Formal analysis, Software, and Writing - review & editing. Olmo van den Akker: Investigation and Writing - review & editing. Samuel Westwood: Investigation and Writing - review & editing. Jacqueline M. Thompson: Conceptualization and Writing - review & editing. Marcus R. Munafò: Conceptualization, Methodology, Supervision, and Writing - review & editing.

For peer review only

Figure captions

Figure 1. PRISMA flowchart of article inclusion

Figure 2. Forest plot of articles reporting the proportion of assessed studies with at least one primary outcome discrepancy.

Figure 3. Forest plot of articles reporting the proportion of assessed studies with at least one secondary outcome discrepancy.

References

1

Zarin DA, Tse T, Williams RJ, *et al.* Update on Trial Registration 11 Years after the ICMJE Policy Was Established. *New England Journal of Medicine* 2017;**376**:383–91. doi:10.1056/NEJMsr1601330

2

De Angelis C, Drazen JM, Frizelle FA, *et al.* Clinical Trial Registration: A Statement from the International Committee of Medical Journal Editors. *New England Journal of Medicine* 2004;**351**:1250–1. doi:10.1056/NEJMe048225

3

ICMJE. Journals stating that they follow the ICMJE Recommendations. 2021.

4

Sim I, Chan A-W, Gülmezoglu AM, *et al.* Clinical trial registration: Transparency is the watchword. *The Lancet* 2006;**367**:1631–3. doi:10.1016/S0140-6736(06)68708-4

For peer review only

5

Chan AW, Pello A, Kitchen J, *et al.* Association of Trial Registration With Reporting of Primary Outcomes in Protocols and Publications. *JAMA* 2017;**318**:1709. doi:10.1001/jama.2017.13001

6

Scott A, Rucklidge JJ, Mulder RT. Is mandatory prospective trial registration working to prevent publication of unregistered trials and selective outcome reporting? An observational study of five psychiatry journals that mandate prospective clinical trial registration. *PLoS ONE* 2015;**10**:1–13. doi:10.1371/journal.pone.0133718

7

Hardwicke TE, Wallach JD, Kidwell MC, *et al.* An empirical assessment of transparency and reproducibility-related research practices in the social sciences (2014). *Royal Society Open Science* 2020;**7**:190806. doi:10.1098/rsos.190806

8

Hardwicke TE, Thibault RT, Kosie JE, *et al.* Estimating the Prevalence of Transparency and Reproducibility-Related Research Practices in Psychology (2014). *Perspectives on Psychological Science* 2021;1745691620979806. doi:10.1177/1745691620979806

9

Scoggins B, Robertson MP. Measuring Transparency in the Social Sciences: Political Science and International Relations. *ECONSTOR* 2023.

10

Thibault RT, Pennington CR, Munaf'o MR. Reflections on Preregistration: Core Criteria, Badges, Complementary Workflows. *Journal of Trial & Error* Published Online First: May 2023. doi:10.36850/mr6

11

Li G, Abbade LPF, Nwosu I, *et al.* A systematic review of comparisons between protocols or registrations and full reports in primary biomedical research. *BMC Medical Research Methodology* 2018;**18**:9. doi:10.1186/s12874-017-0465-7

12

Dwan K, Gamble C, Williamson PR, *et al.* Systematic Review of the Empirical Evidence of Study Publication Bias and Outcome Reporting Bias An Updated Review. *PLOS ONE* 2013;**8**:e66844. doi:10.1371/journal.pone.0066844

13

Jones CW, Keil LG, Holland WC, *et al.* Comparison of registered and published outcomes in randomized controlled trials: A systematic review. *BMC MEDICINE* 2015;**13**. doi:10.1186/s12916-015-0520-3

14

Calmejane L, Dechartres A, Tran VT, *et al.* Making protocols available with the article improved evaluation of selective outcome reporting. *Journal of Clinical Epidemiology* 2018;**104**:95–102. doi:10.1016/j.jclinepi.2018.08.020

15

Annals of Internal Medicine Editors. Discrepancies Between Prespecified and Reported Outcomes. *Annals of Internal Medicine* 2016;**164**:374–5. doi:10.7326/L15-0615

16

Chan AW, Hr'objartsson A, Haahr MT, *et al. Empirical evidence for selective reporting of outcomes in randomized trials: Comparison of protocols to published articles*. 2004. doi:10.1001/jama.291.20.2457

17

Hartung J, Knapp G. An Alternative Test Procedure for Meta-Analysis. In: *Meta-analysis: New Developments and Applications in Medical and Social Sciences*. Ashland, OH, US: : Hogrefe & Huber Publishers 2003. 53–69.

18

Hoekstra R, Morey RD, Rouder JN, *et al.* Robust misinterpretation of confidence intervals. *Psychonomic Bulletin and Review* 2014;**21**:1157–64. doi:10.3758/s13423-013-0572-3

19

Riley RD, Higgins JPT, Deeks JJ. Interpretation of random effects meta-analyses. *BMJ* 2011;**342**:d549. doi:10.1136/bmj.d549

20

Higgins JPT, Thompson SG, Spiegelhalter DJ. A re-evaluation of random-effects meta-analysis. *Journal of the Royal Statistical Society Series A, (Statistics in Society)* 2009;**172**:137–59. doi:10.1111/j.1467-985X.2008.00552.x

21

Ofosu G, Posner DN. Pre-analysis Plans: A Stocktaking. MetaArXiv 2019. doi:10.31222/osf.io/e4pum

22

Claesen A, Gomes SLBT, tuerlinckx francis, *et al.* Preregistration: Comparing Dream to Reality. PsyArXiv 2019. doi:10.31234/osf.io/d8wex

23

Goldacre B, Drysdale H, Dale A, *et al.* COMPare: A prospective cohort study correcting and monitoring 58 misreported trials in real time. *Trials* 2019;**20**:1–16. doi:10.1186/s13063-019-3173-2

24

Jones CW, Adams A, Misemer BS, *et al.* Peer Reviewed Evaluation of Registered End-Points of Randomised Trials (the PRE-REPORT study): A stepped wedge, cluster-randomised trial. *BMJ Open* 2022;**12**:e066624. doi:10.1136/bmjopen-2022-066624

25

TARG Meta-Research Group & Collaborators. Discrepancy review: A feasibility study of a novel peer review intervention to reduce undisclosed discrepancies between registrations and publications | Royal Society Open Science. *Royal Society Open Science* Published Online First: 2022. doi:10.1098/rsos.220142

26

World Medical Association. Declaration of Helsinki Ethical Principles for Medical Research Involving Human Subjects. World Medical Association 2013.

27

ICMJE. Recommendations for the Conduct, Reporting, Editing, and Publication of Scholarly Work in Medical Journals. ICMJE 2019.

28

IntHout J, Ioannidis JPA, Rovers MM, *et al.* Plea for routinely presenting prediction intervals in meta-analysis. *BMJ Open* 2016;**6**:e010247. doi:10.1136/bmjopen-2015-010247

29

DeVito NJ. *Trial registries for transparency and accountability in clinical research*. 2022.

30

Kaplan RM, Irvin VL. Likelihood of Null Effects of Large NHLBI Clinical Trials Has Increased over Time. *PLOS ONE* 2015;**10**:e0132382. doi:10.1371/journal.pone.0132382

31

DeVito NJ, Bacon S, Goldacre B. FDAAA TrialsTracker: A live informatics tool to monitor compliance with FDA requirements to report clinical trial results. *bioRxiv* 2019;266452. doi:10.1101/266452

For peer review only

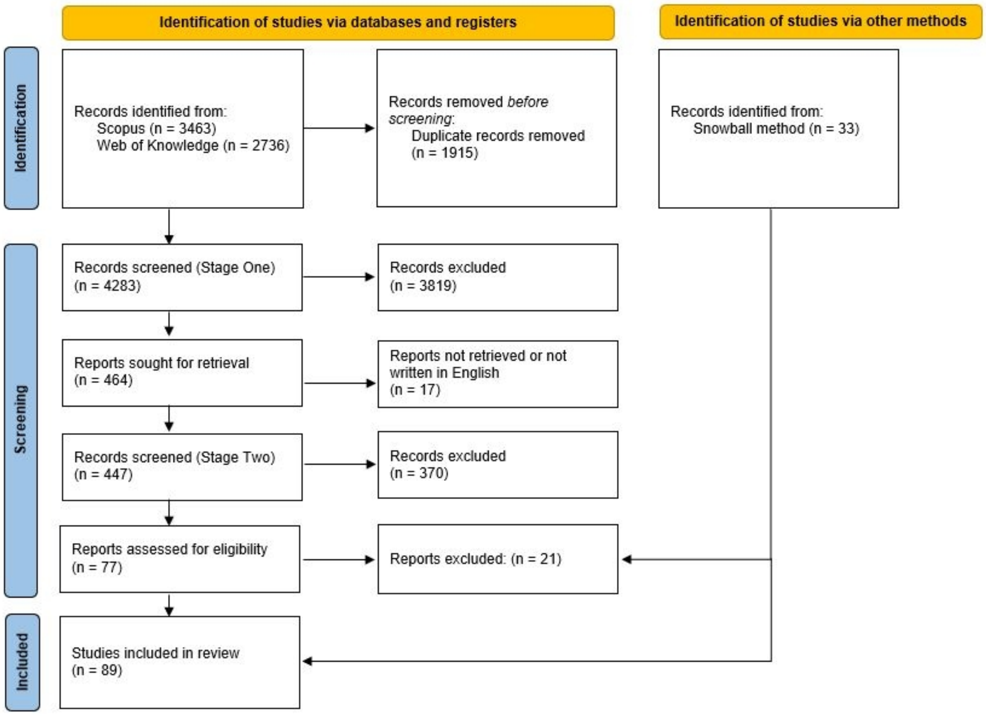

Figure 1. PRISMA flowchart of article inclusion

218x157mm (96 x 96 DPI)

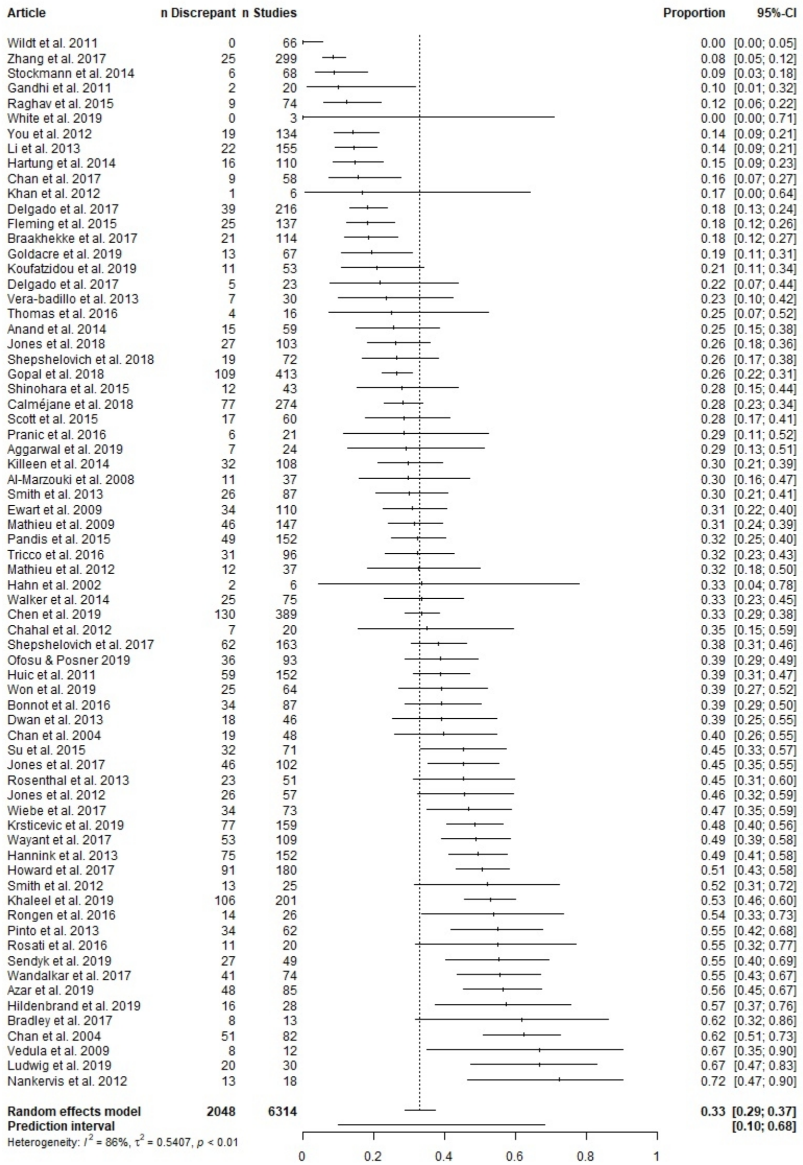

Figure 2. Forest plot of articles reporting the proportion of assessed studies with at least one primary outcome discrepancy.

141x202mm (144 x 144 DPI)

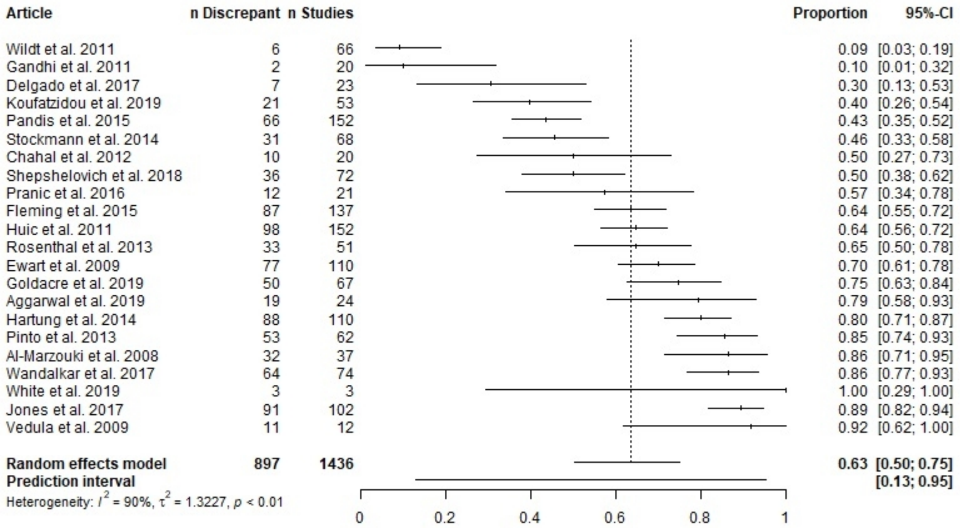

Figure 3. Forest plot of articles reporting the proportion of assessed studies with at least one secondary outcome discrepancy.

141x84mm (144 x 144 DPI)

Supplementary materials

A. Protocol amendments

As our systematic review was largely exploratory, many elements of the protocol are detailed more precisely in the manuscript than they were in our preregistered study protocol. We made the following amendments to this protocol:

- We included additional contributors and some of the projected contributor roles changed.
- Before beginning data extraction, we operationalized Table 2 from the protocol into a Qualtrics survey form and made the items more precise and more detailed.
- The protocol uses the term "*preregistration-publication discrepancies*". However, because many articles were unclear whether the registrations were prospective, we included data on discrepancies between publications and registrations regardless of the timing of registration. We also include discrepancies between publications and protocols, ethics applications, grant applications, and marketing applications. We did not foresee articles being published on discrepancies between these documents, but believe them to be relevant to our systematic review.
- Whereas the protocol states that the review will be descriptive, we used inferential statistics to run meta-analyses and to test for parameters that might influence the prevalence of discrepancies (i.e., the analyses presented in Table 2). We had preregistered that we would search for "*Additional inferential statistics relevant to preregistration–publication discrepancies*", but did not preregister that we would run our own inferential analyses. We used inferential statistics because we found many more articles than we anticipated and we developed our data extraction form to have more precision than we originally intended.
- The scoping review was terminated, as explained in the updated version of the registration associated with this study.
- When screening, for that sake of simplicity, we did not end up using the bin "*(iv) relevant to preregistration but not directly relevant to our study objectives.*"
- A peer reviewer suggested we run a meta-regression which includes all the article-level characteristics. We ran this meta-regression after knowing the results of all the meta-regressions we ran for single article-level characteristics. We report on this analysis in Supplementary Material E.
- We originally planned to share our data and code on the University of Bristol Data Repository. Instead, we have shared it on osf.io (because more people are familiar with this platform) and on www.codeocean.com (because this platform supports reproducible containers).

For peer review only

For peer review only - http://bmjopen.bmj.com/site/about/guidelines.xhtml

B. Coding

The coding form is divided into five sections and available at osf.io/728ys. Section One coded the search strategy and design of each article, including how the researchers identified studies (e.g., through registries or journals), first and last year of publication of the assessed studies, whether the article was solely observational, the research discipline assessed (as defined by the Scopus discipline categories outlined in Appendix C of our preregistered protocol), and a verbatim copy of the main conclusion that was relevant to findings on discrepancies.

Section Two coded when the assessed studies were registered (e.g., prospectively, retrospectively, unclear), the version of the registration used when checking for discrepancies (e.g., original version, most recent version), and whether the article checked if authors disclosed reasons for any of the discrepancies.

Section Three coded outcome discrepancies. Whereas some articles made it clear that they only assessed studies that were prospectively registered, others were unclear about when the studies they assessed were registered. We coded discrepancies for unambiguously preregistered studies separate from studies with unclear registration timing or retrospective registration. If an article reported discrepancies separately for prospectively and retrospectively registered studies, we coded both these categories. We divided outcome discrepancies into 10 non-exclusive subcategories: any outcome discrepancy, a primary outcome discrepancy, a secondary outcome discrepancy, demoting a primary outcome to a secondary outcome, omitting a primary outcome, adding a primary outcome, promoting a secondary outcome to a primary outcome, omitting a secondary outcome, adding a secondary outcome, and changing the timing at which an outcome was measured. We only coded *any outcome discrepancy* for articles that checked for both primary and secondary outcome discrepancies in the studies they assessed. If we had also included articles that only checked for primary outcome discrepancies in this sub-category, then we would have biased our estimate downwards because the assessed studies may also have secondary outcome discrepancies, but that information would not be documented. Articles used slightly different methods for identifying and defining primary outcome discrepancies. Rather than use a stringent definition of what constitutes a primary outcome discrepancy across all articles, we coded the number of studies with primary outcome discrepancies based on the data presented in the results section of each article, regardless of the method or definition the article used. A definition used in many articles comes from Chan, Hr'objartsson, et al. (2004) and includes (1) demoting a primary outcome to a secondary outcome, (2) omitting a primary outcome, (3) adding a primary outcome, (4) promoting a secondary outcome to a primary outcome, and (5) changing the timing at which a primary outcome is measured.

Section Four coded non-outcome discrepancies. These included discrepancies in participant eligibility (e.g., inclusions and exclusion criteria), sample size, randomization (e.g., number of experimental arms), method of blinding (e.g., single versus double blind), interventions (e.g., a change in the drug administered), study duration, analyses, subgroup analyses, funding, and reported results. There was heterogeneity between articles in their method for identifying and defining each type of discrepancy. As we did for the outcome discrepancies, we coded the number of studies with each type of discrepancies based on the data presented in the results section of each article, regardless of the method or definition the article used.

Section Five noted whether articles provided any additional descriptive or inferential statistics on discrepancies. In particular, it noted whether discrepancies were related to funding source, statistical significance, or the timing of registration.

For each type of discrepancy, we coded the numbers of publications containing at least one discrepancy of that type, rather than coding the total number of discrepancies of each type per publication (e.g., a single publication may have five discrepancies in secondary outcomes). Of the $k = 89$ articles we reviewed, 17 reported a measure of the total number of discrepancies. Sixteen of these 17 articles also reported the number of publications with at least one discrepancy, and as such we were able to include them in our analysis. We excluded the one remaining article that did not report the number of publications with at least one discrepancy. We do not analyze the total number of discrepancies in the present report.

After completing the data extraction and analyses described above, we extracted additional information from articles that we had identified as providing information on whether discrepancies were disclosed, as well as

For peer review only - http://bmjopen.bmj.com/site/about/guidelines.xhtml

whether discrepancies were related to funding source, statistical significance, or the timing of registration. We extracted this information directly into a spreadsheet, we did not create an additional coding form. We divided studies into industry funded or non-industry funded (which included public, private, and no funding). We excluded studies from this analysis if they did not report their funding or were funded by both industry and non-industry sources. We also coded the proportion of discrepancies that favored statistical significance. Favoring statistical significance included when a novel significant outcome was introduced, or a non-significant primary outcome was downgraded to a secondary outcome. It is impossible to know whether omitted outcomes were statistically significant or not. If we were to assume that the majority of omitted outcomes were non-significant, then this analysis would provide an underestimation of the proportion of studies that favor statistical significance.

For peer review only

For peer review only - http://bmjopen.bmj.com/site/about/guidelines.xhtml

C. Article characteristics

We identified and reviewed k = 89 articles that report at least one type of discrepancy. The vast majority of these articles assessed medical research (k = 81). The remaining articles focused on dentistry (k = 3), psychology (k = 3), physical therapy (k = 1), and economics (k = 1).

Articles compared publications to registrations deposited in conventional registries such as clinicaltrials.gov (k = 73), or to protocols submitted for research ethics review (k = 7), funding applications (k = 2), marketing approval (k = 2), or other purposes (k = 5). The 89 articles we reviewed identified studies through different sources, including journals (k = 33), registries (k = 27), search engines (k = 19), research ethics boards (k = 7), funders (k = 3), regulatory agencies (k = 2), and research groups (k = 2). Four articles used multiple sources. Seventy-one articles reported the year the earliest study they included was published (median = 2009, IQR: 2004-2012), and 80 reported the last year (median = 2013, IQR: 2010-2015).

All but 10 articles were solely observational. Three of these 10 articles emailed authors for registration numbers (Ross et al., 2019; Shinohara et al., 2015; Wiebe et al., 2017) and one emailed authors for full protocols (Saquib et al., 2013). Four articles sent a survey to researchers to ask about the discrepancies (Chan, Hr'objartsson, et al., 2004; Chan, Krleža-Jeri'c, et al., 2004; Redmond et al., 2013; Wayant et al., 2017). One article sent a survey to better understand user experience with pre-analysis plans (Ofosu & Posner, 2019). Only one article attempted to correct published discrepancies. They sent letters to the editor within weeks of a study being published with outcome discrepancies (Goldacre et al., 2019). We do not further discuss the non-observational aspects of these studies in this review.

Whereas k = 29 articles clearly state that they compared the publications to the original registry entry or protocol, 15 used the most recent version of the registry entry, 35 do not mention which registry version they used, and 10 used a different version of the registration or were unclear about the version they used.

For peer review only

D. Meta-analyses of each outcome and non-outcome discrepancy separated by timing of registration

Here, we performed two additional sets of random effects meta-analyses: (1) for articles that reported on unambiguously prospectively registered studies (i.e., studies that were clearly prospectively registered), (2) for articles that reported on studies where the timing of registration was unclear.

While some of the outcome discrepancies may appear to occur more frequently in studies that were unambiguously identified as prospectively registered, as compared to those with unclear timing of registration, the confidence intervals are largely overlapping. And yet, we cannot ascertain that prospectively and retrospectively registered studies have a similar frequency of discrepancies because the unclear category consists of both retrospectively and prospectively registered studies in an unknown proportion. We deemed it unreasonable to compare unambiguously prospectively registered studies to studies with unclear timing of registration because there were too few studies and their methods were highly heterogeneous.

Supplementary Table D1. Meta-analytic estimates for the proportion of studies that contain various types of outcome discrepancies and non-outcome discrepancies. Articles are separated into those which unambiguously assessed only prospectively registered studies versus articles where the registration timing of the assessed studies was retrospective, unclear, or mixed. This table is essentially a combination of Table 2 and Table 4 from the manuscript, but grouped by timing of registration.

	Prospective registration				Unclear registration timing			
	95% CI	95% PI	k	n	95% CI	95% PI	k	n
Any outcome discrepancy	38-91%	5-99%	6	279	32-70%	6-95%	13	850
Any primary outcome discrepancy	25-38%	11-64%	24	1767	28-39%	9-72%	51	4857
Any secondary outcome discrepancy	46-87%	12-98%	7	506	44-74%	11-95%	16	1052
Primary outcome demoted to secondary outcome	7-18%	2-44%	15	1287	5-9%	2-27%	36	3273
Primary outcome omitted	6-16%	1-56%	20	1600	6-11%	1-36%	32	2860
Primary outcome added	7-14%	3-30%	18	1403	6-12%	1-37%	36	3294
Secondary outcome promoted to primary outcome	3-9%	1-25%	14	1250	3-6%	1-18%	32	2885
Secondary outcome omitted	14-62%	2-93%	7	566	14-31%	5-58%	12	799
Secondary outcome added	8-80%	0.3-99%	6	413	18-41%	5-73%	14	892
Timing of outcome measurement changed	3-18%	1-55%	9	864	6-19%	1-68%	21	2608
Eligibility criteria	1-99%	23-67%	2	50	23-59%	4-92%	13	1103
Sample size	40-59%	39-60%	6	174	22-43%	6-78%	20	1244
Randomization	46-70%	46-70%	1	58	1-56%	0.02-98%	4	118
Blinding	NA	NA	NA	NA	5-42%	0.04-99%	3	224
Intervention	1-22%	1-22%	1	27	3-63%	0.1-98%	6	523
Study duration	NA	NA	NA	NA	3-89%	0.02-99.94%	4	184
Analysis	24-71%	6-92%	6	232	7-46%	2-80%	6	172
Subgroup analysis	7-99%	0.01-99.998%	4	215	20-97%	1-99.93%	5	330
Funding	7-67%	7-67%	1	7	4-93%	0.01-99.98%	4	205
Results	4-20%	4-20%	1	54	7-91%	0.1-99.8%	5	208

Values less than 0.5% and greater than 99.5% are rounded to one significant digit from 0 or 100. Prediction intervals cannot be calculated for meta-analyses with fewer than three studies.

For peer review only - http://bmjopen.bmj.com/site/about/guidelines.xhtml

The 38 forest plots that follow align with the meta-analyses presented in Supplementary Table D1.

For peer review only

For peer review only - http://bmjopen.bmj.com/site/about/guidelines.xhtml

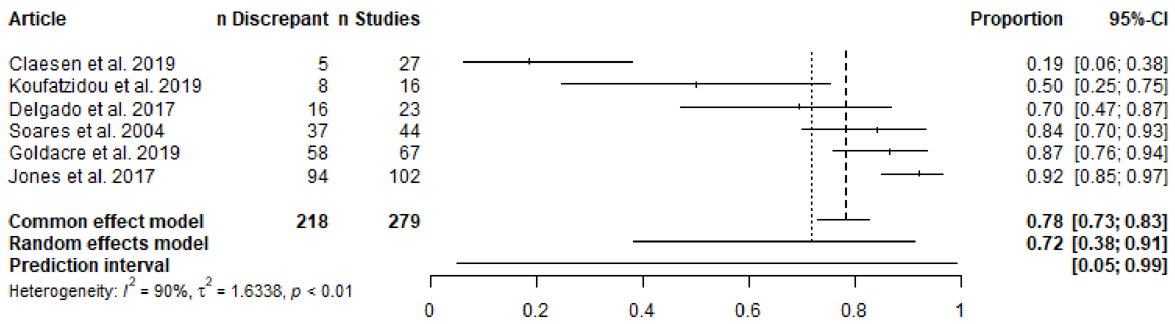

Supplementary Figure D1. Any outcome discrepancy, prospective.

For peer review only - http://bmjopen.bmj.com/site/about/guidelines.xhtml

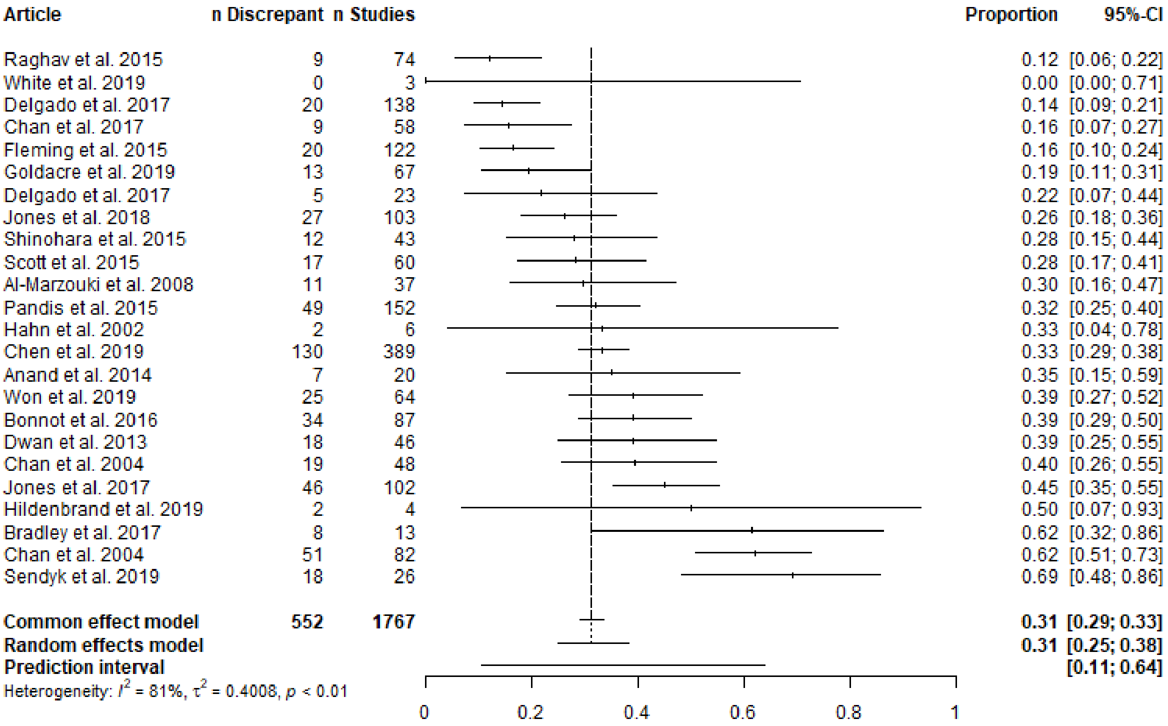

Supplementary Figure D2. Any primary outcome discrepancy, prospective.

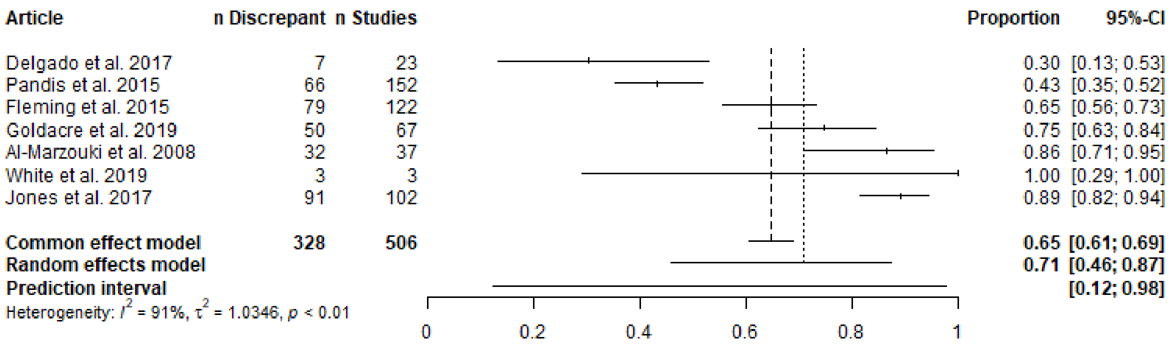

Supplementary Figure D3. Any secondary outcome discrepancy, prospective.

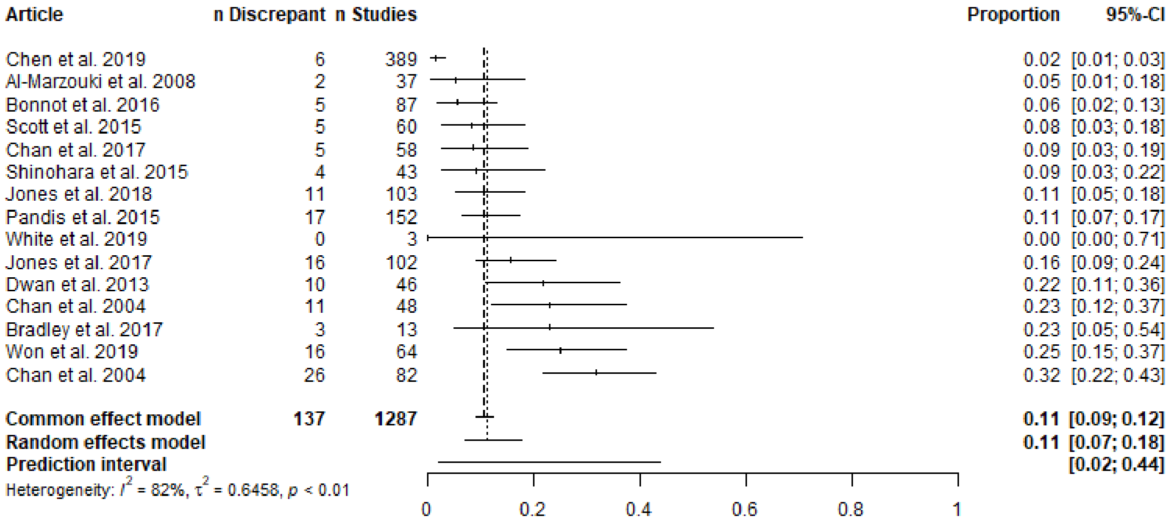

Supplementary Figure D4. Primary outcome demoted to secondary outcome, prospective.

For peer review only - http://bmjopen.bmj.com/site/about/guidelines.xhtml

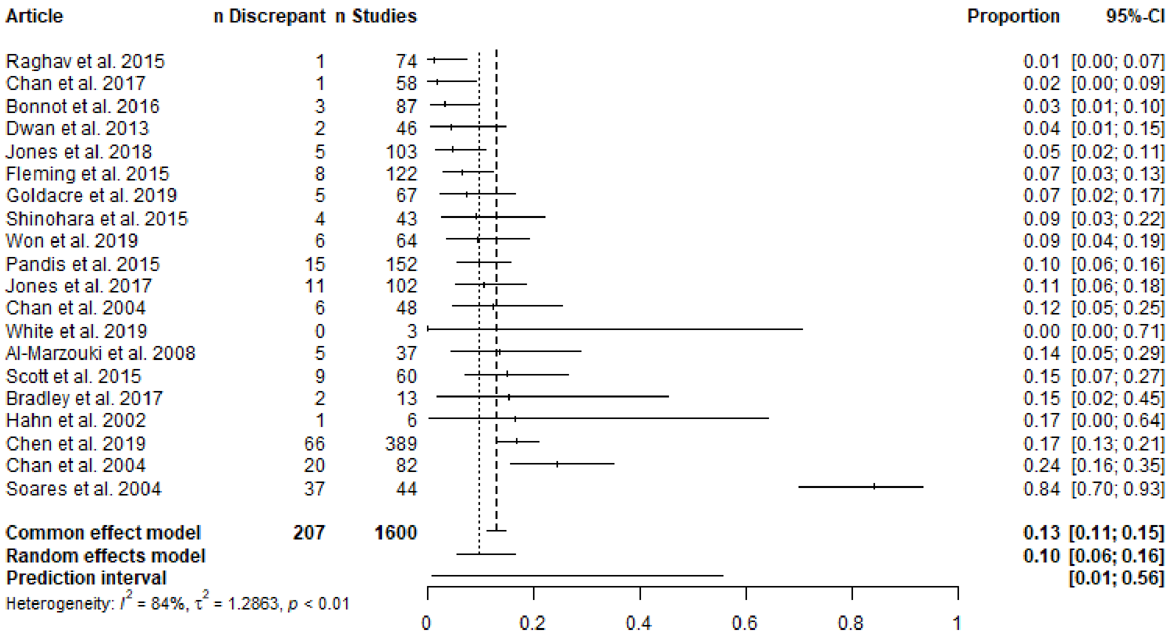

Supplementary Figure D5. Primary outcome omitted, prospective.

For peer review only - http://bmjopen.bmj.com/site/about/guidelines.xhtml

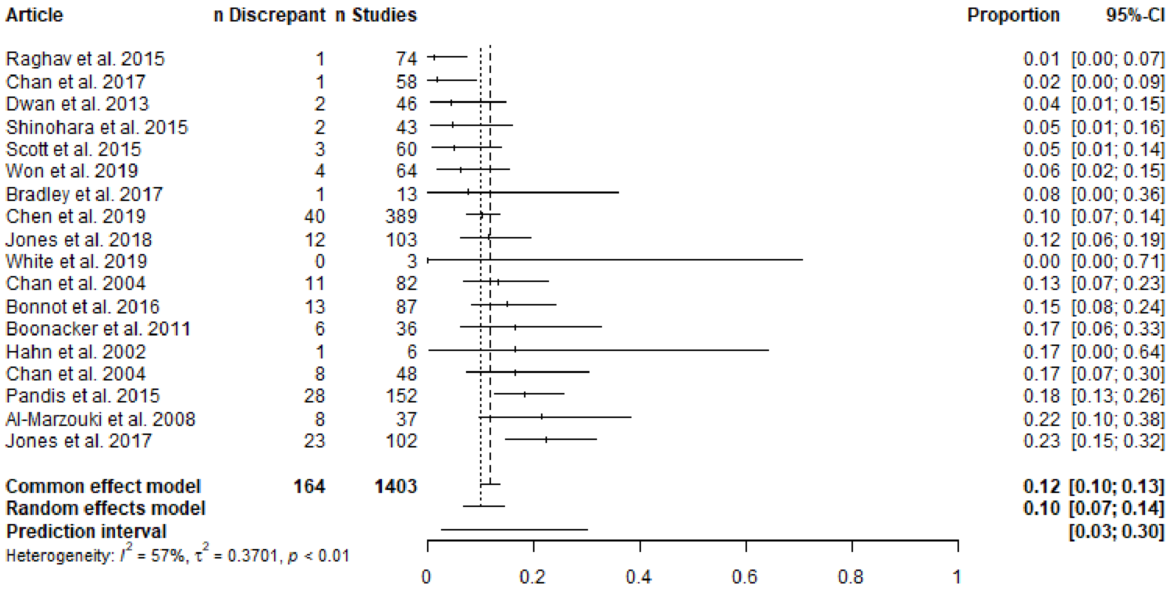

Supplementary Figure D6. Primary outcome added, prospective.

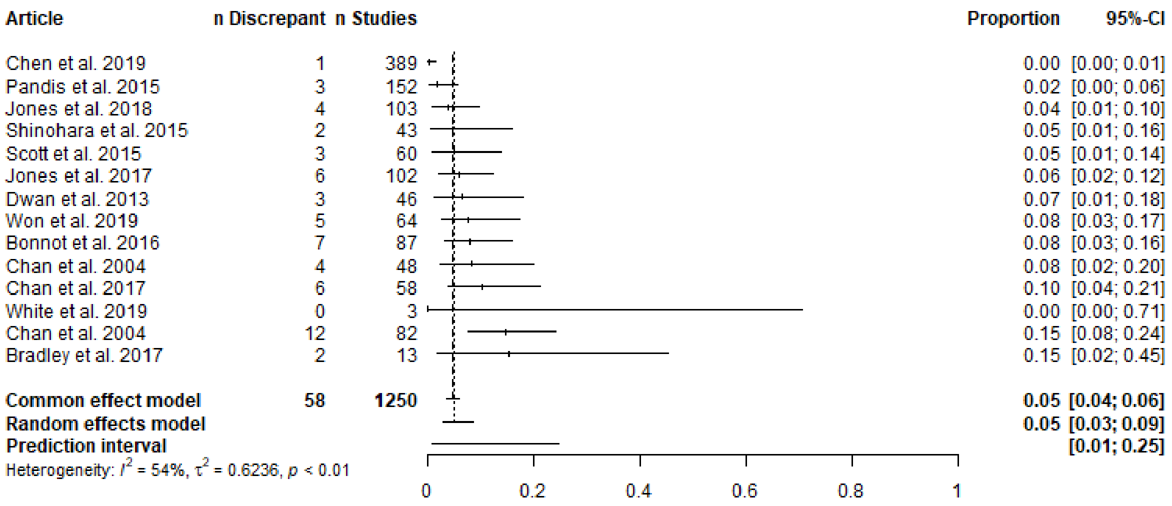

Supplementary Figure D7. Secondary outcome promoted to primary outcome, prospective.

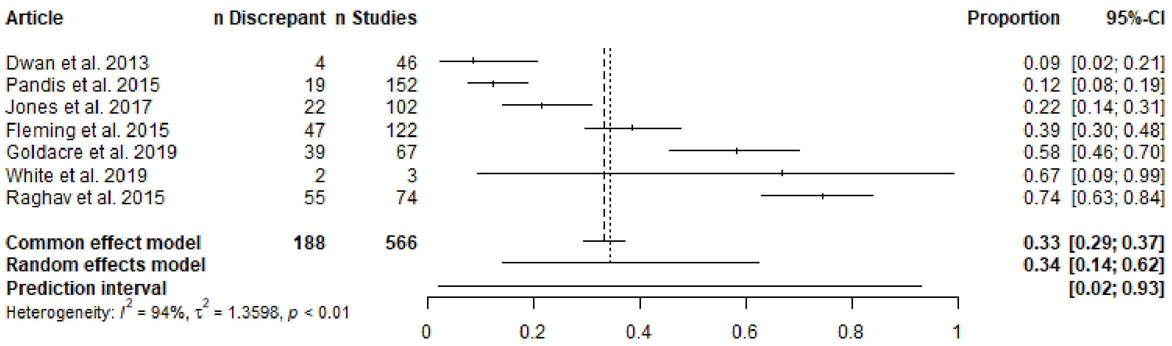

Supplementary Figure D8. Secondary outcome omitted, prospective.

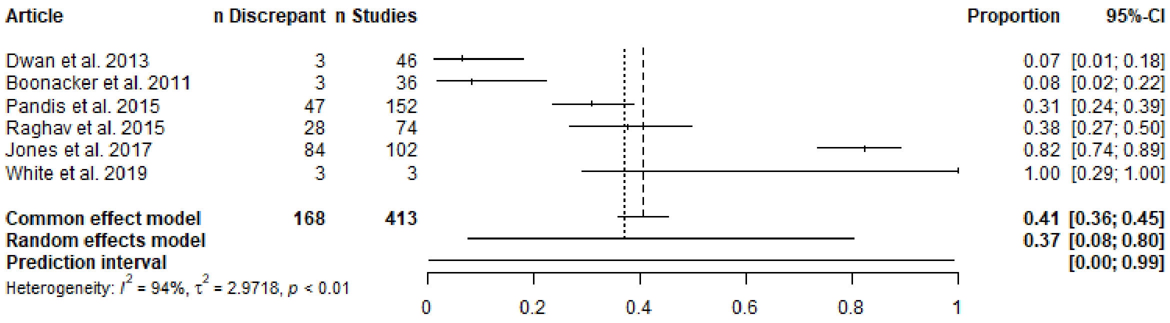

Supplementary Figure D9. Secondary outcome added, prospective.

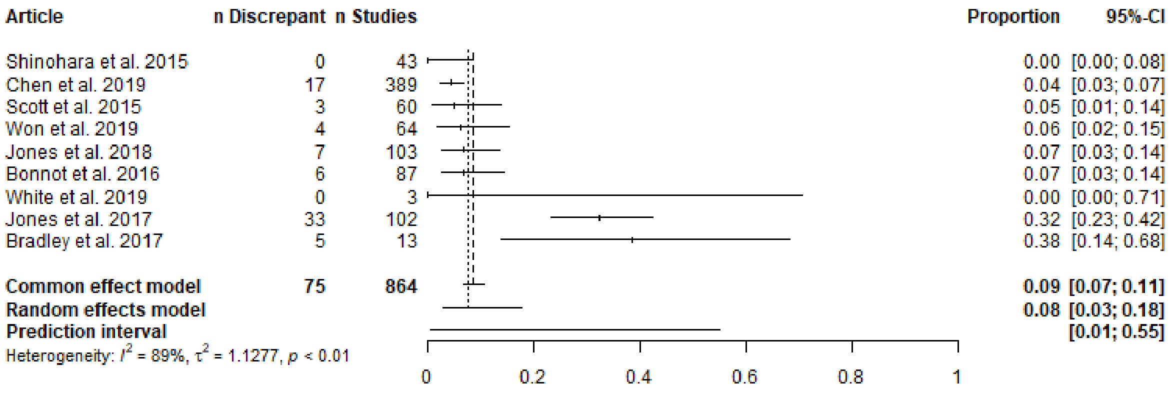

Supplementary Figure D10. Timing of outcome measurement changed, prospective.

For peer review only - http://bmjopen.bmj.com/site/about/guidelines.xhtml

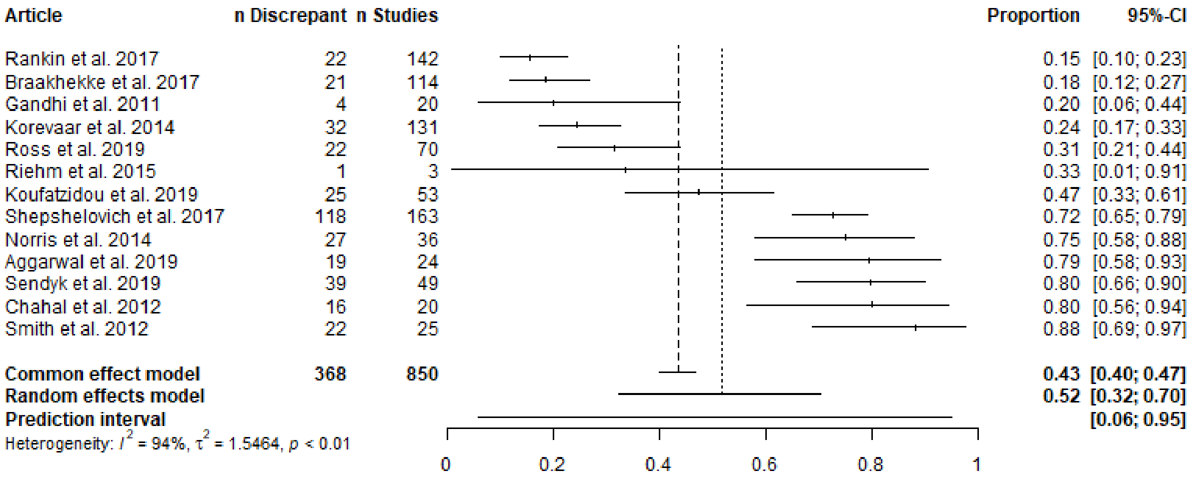

Supplementary Figure D11. Any outcome discrepancy, unclear registration timing.

Article	n Discrepant	n Studies	Proportion	95%-CI
Wildt et al. 2011	0	66	0.00	[0.00; 0.05]
Zhang et al. 2017	25	299	0.08	[0.05; 0.12]
Stockmann et al. 2014	6	68	0.09	[0.03; 0.18]
Gandhi et al. 2011	2	20	0.10	[0.01; 0.32]
You et al. 2012	19	134	0.14	[0.09; 0.21]
Li et al. 2013	22	155	0.14	[0.09; 0.21]
Hartung et al. 2014	16	110	0.15	[0.09; 0.23]
Khan et al. 2012	1	6	0.17	[0.00; 0.64]
Delgado et al. 2017	39	216	0.18	[0.13; 0.24]
Fleming et al. 2015	25	137	0.18	[0.12; 0.26]
Braakhekke et al. 2017	21	114	0.18	[0.12; 0.27]
Koufatzidou et al. 2019	11	53	0.21	[0.11; 0.34]
Vera-badillo et al. 2013	7	30	0.23	[0.10; 0.42]
Thomas et al. 2016	4	16	0.25	[0.07; 0.52]
Anand et al. 2014	15	59	0.25	[0.15; 0.38]
Shepshelovich et al. 2018	19	72	0.26	[0.17; 0.38]
Gopal et al. 2018	109	413	0.26	[0.22; 0.31]
Calméjane et al. 2018	77	274	0.28	[0.23; 0.34]
Pranić et al. 2016	6	21	0.29	[0.11; 0.52]
Aggarwal et al. 2019	7	24	0.29	[0.13; 0.51]
Killeen et al. 2014	32	108	0.30	[0.21; 0.39]
Smith et al. 2013	26	87	0.30	[0.21; 0.41]
Ewart et al. 2009	34	110	0.31	[0.22; 0.40]
Mathieu et al. 2009	46	147	0.31	[0.24; 0.39]
Tricco et al. 2016	31	96	0.32	[0.23; 0.43]
Mathieu et al. 2012	12	37	0.32	[0.18; 0.50]
Walker et al. 2014	25	75	0.33	[0.23; 0.45]
Chahal et al. 2012	7	20	0.35	[0.15; 0.59]
Shepshelovich et al. 2017	62	163	0.38	[0.31; 0.46]
Ofosu & Posner 2019	36	93	0.39	[0.29; 0.49]
Huić et al. 2011	59	152	0.39	[0.31; 0.47]
Su et al. 2015	32	71	0.45	[0.33; 0.57]
Rosenthal et al. 2013	23	51	0.45	[0.31; 0.60]
Jones et al. 2012	26	57	0.46	[0.32; 0.59]
Wiebe et al. 2017	34	73	0.47	[0.35; 0.59]
Krsticevic et al. 2019	77	159	0.48	[0.40; 0.56]
Wayant et al. 2017	53	109	0.49	[0.39; 0.58]
Hannink et al. 2013	75	152	0.49	[0.41; 0.58]
Howard et al. 2017	91	180	0.51	[0.43; 0.58]
Smith et al. 2012	13	25	0.52	[0.31; 0.72]
Khaleel et al. 2019	106	201	0.53	[0.46; 0.60]
Rongen et al. 2016	14	26	0.54	[0.33; 0.73]
Pinto et al. 2013	34	62	0.55	[0.42; 0.68]
Rosati et al. 2016	11	20	0.55	[0.32; 0.77]
Sendyk et al. 2019	27	49	0.55	[0.40; 0.69]
Wandalkar et al. 2017	41	74	0.55	[0.43; 0.67]
Azar et al. 2019	48	85	0.56	[0.45; 0.67]
Hildenbrand et al. 2019	16	28	0.57	[0.37; 0.76]
Vedula et al. 2009	8	12	0.67	[0.35; 0.90]
Ludwig et al. 2019	20	30	0.67	[0.47; 0.83]
Nankervis et al. 2012	13	18	0.72	[0.47; 0.90]
**Common effect model**	**1563**	**4857**	**0.32**	**[0.31; 0.34]**
**Random effects model**			**0.33**	**[0.28; 0.39]**
**Prediction interval**				**[0.09; 0.72]**

Heterogeneity: $I^2 = 88\%$, $\tau^2 = 0.6399$, $p < 0.01$

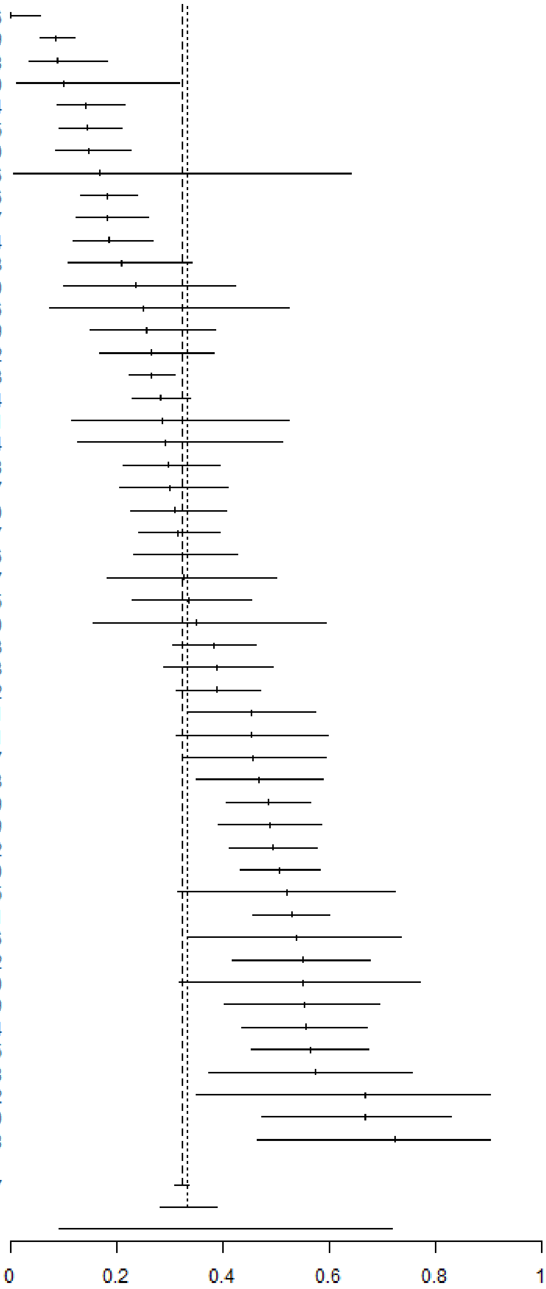

Supplementary Figure D12. Any primary outcome discrepancy, unclear registration timing.

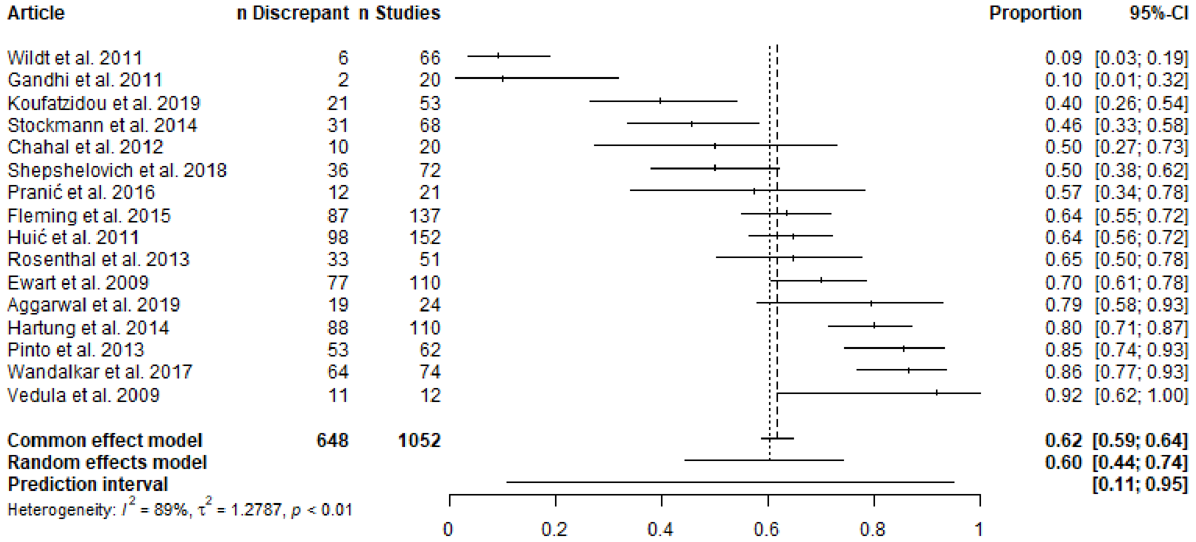

Supplementary Figure D13. Any secondary outcome discrepancy, unclear registration timing.

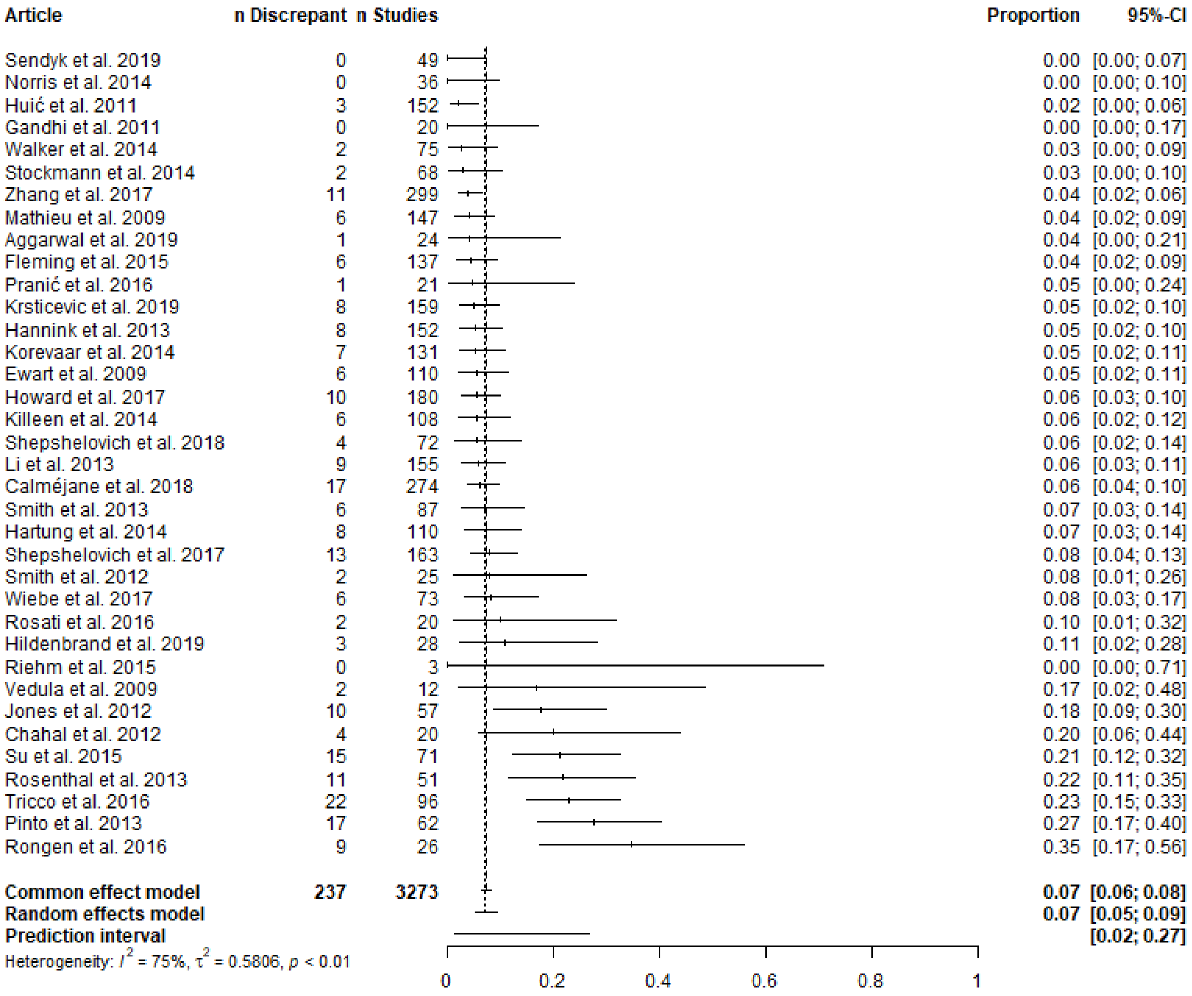

Supplementary Figure D14. Primary outcome demoted to secondary outcome, unclear registration timing.

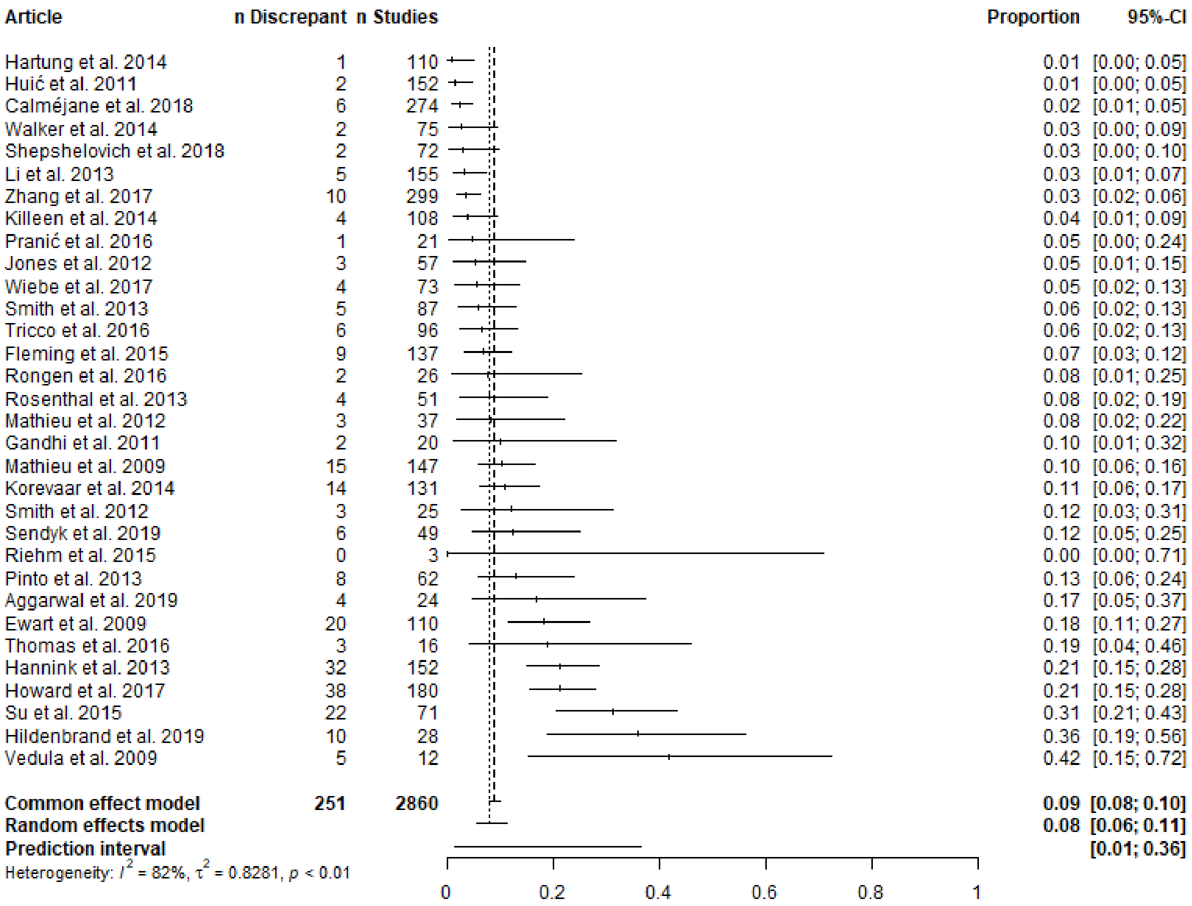

Supplementary Figure D15. Primary outcome omitted, unclear registration timing.

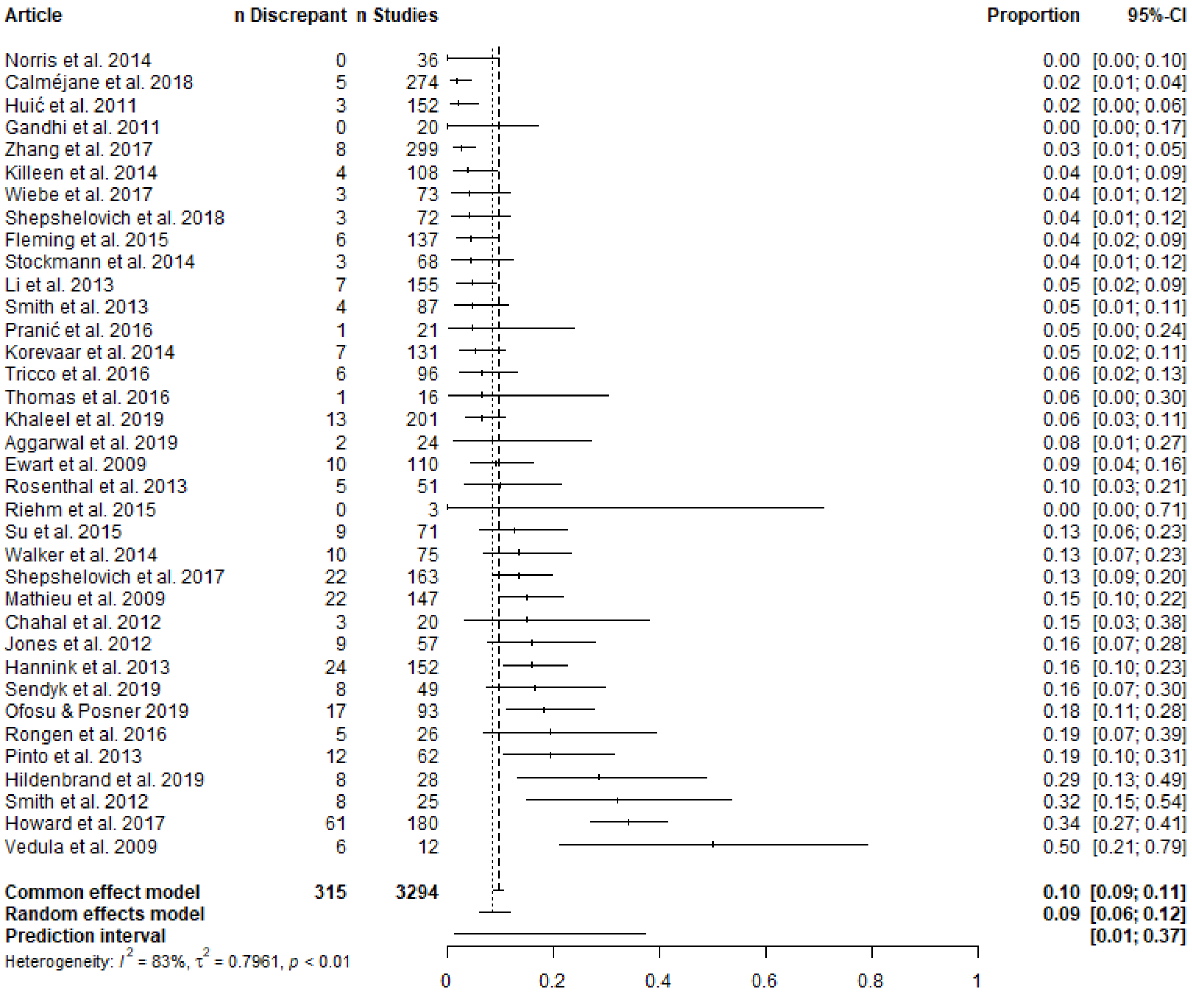

Supplementary Figure D16. Primary outcome added, unclear registration timing.

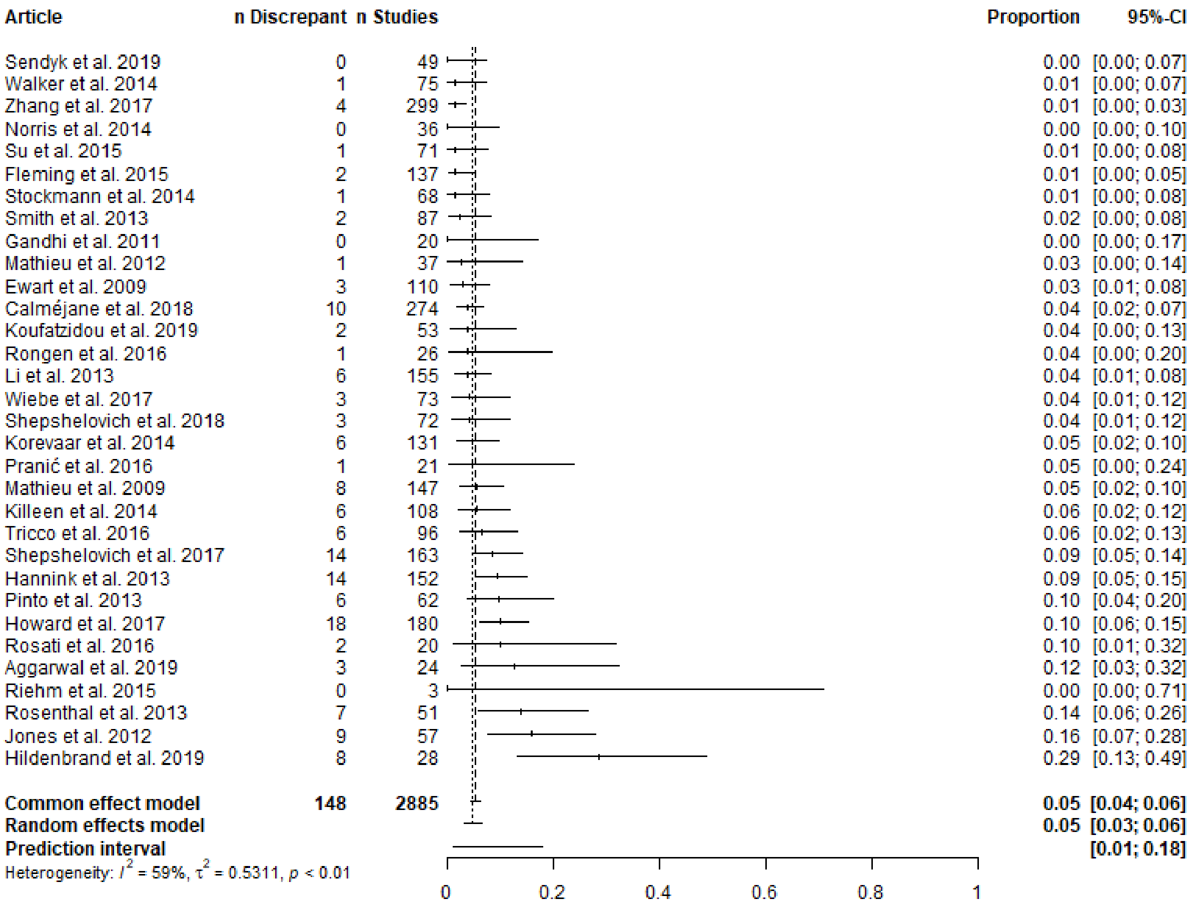

Supplementary Figure D17. Secondary outcome promoted to primary outcome, unclear registration timing.

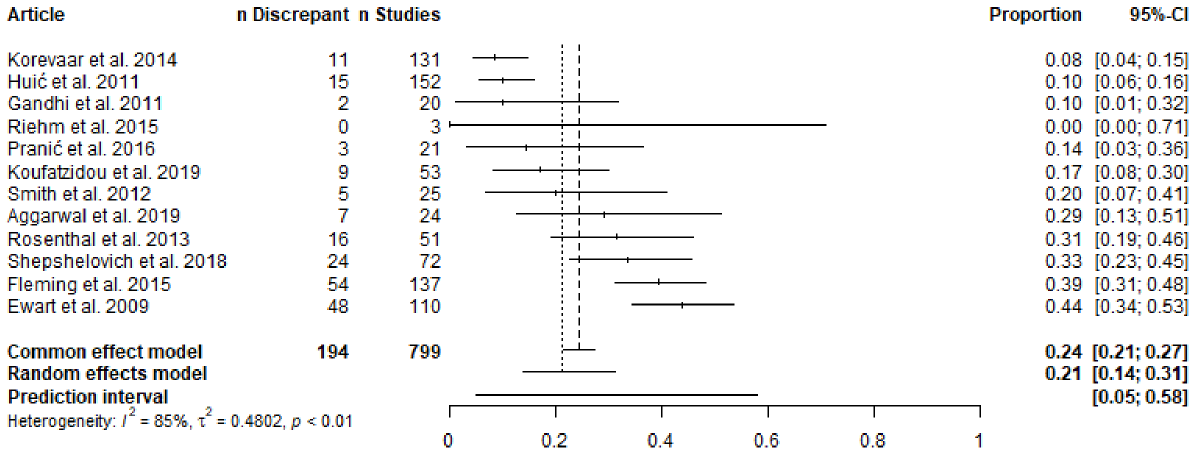

Supplementary Figure D17. Secondary outcome omitted, unclear registration timing.

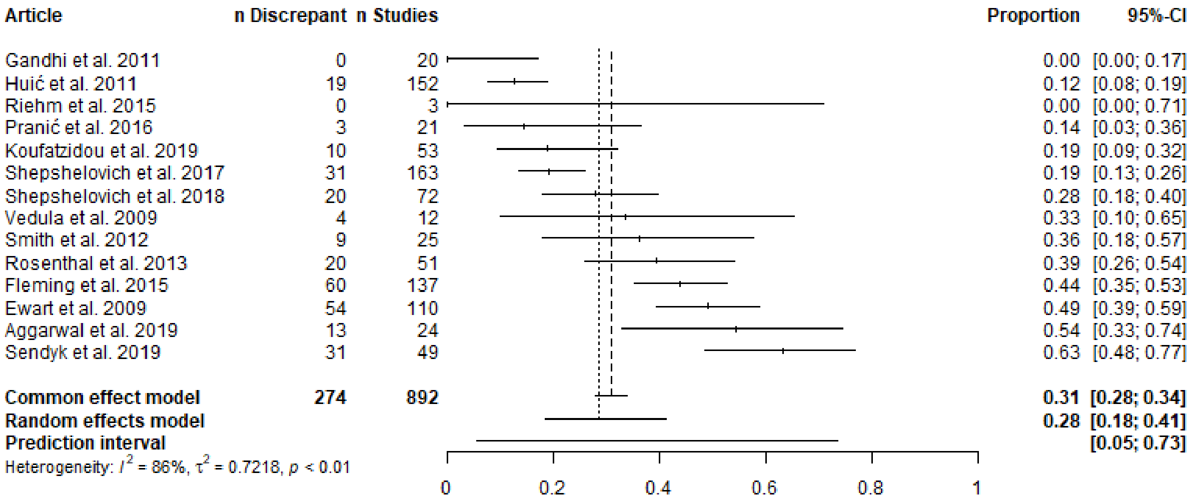

Supplementary Figure D19. Secondary outcome added, unclear registration timing.

For peer review only - http://bmjopen.bmj.com/site/about/guidelines.xhtml

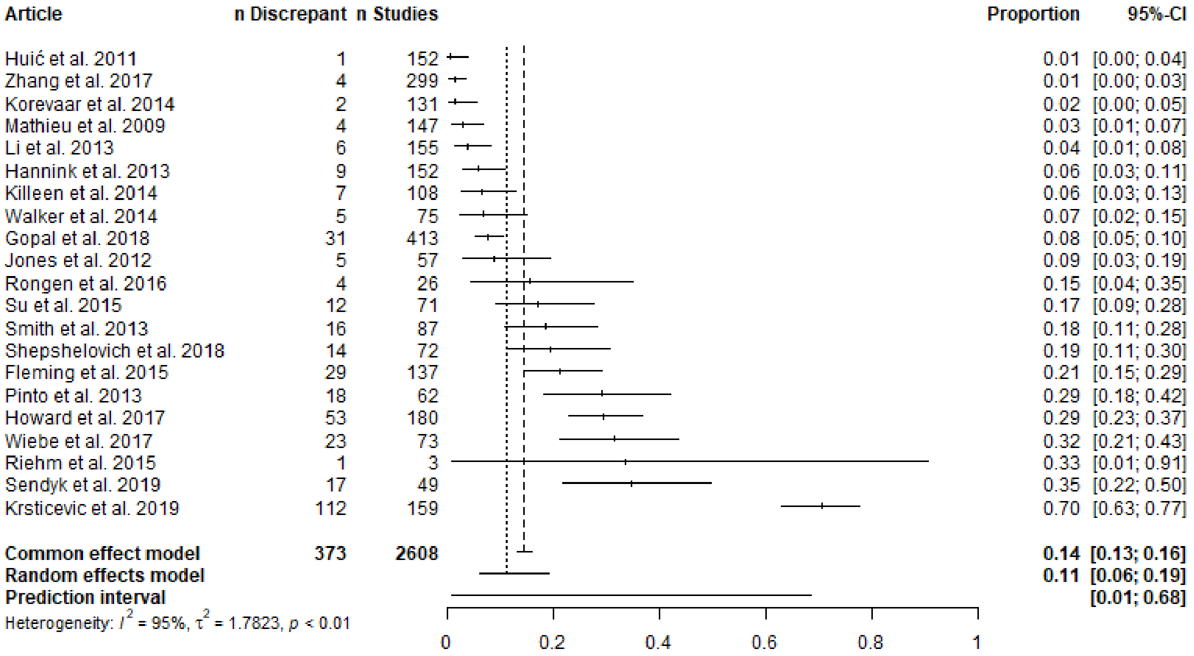

Supplementary Figure D20. Timing of outcome measurement changed, unclear registration timing.

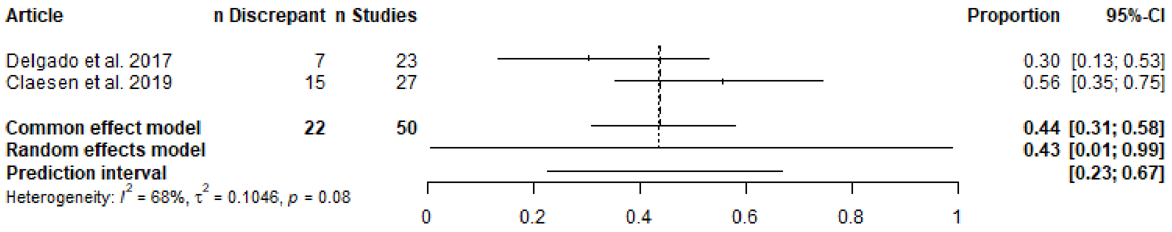

Supplementary Figure D21. Eligibility criteria, prospective.

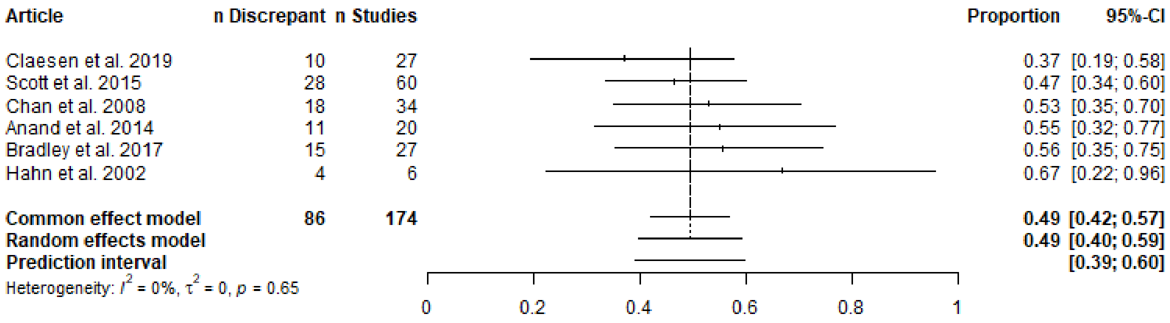

Supplementary Figure D22. Sample size, prospective.

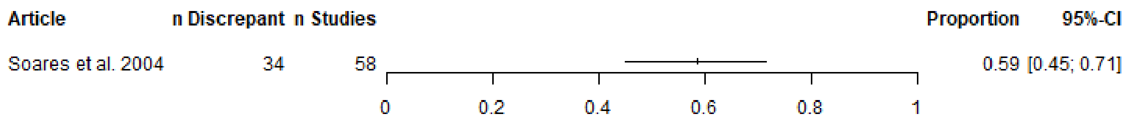

Supplementary Figure D23. Randomization, prospective.

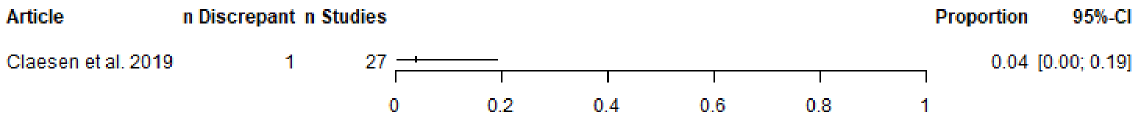

Supplementary Figure D24. Intervention, prospective.

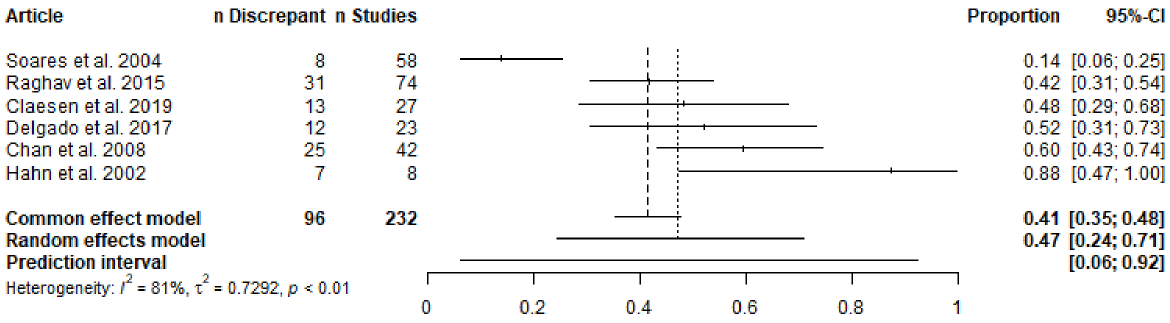

Supplementary Figure D25. Analyses, prospective.

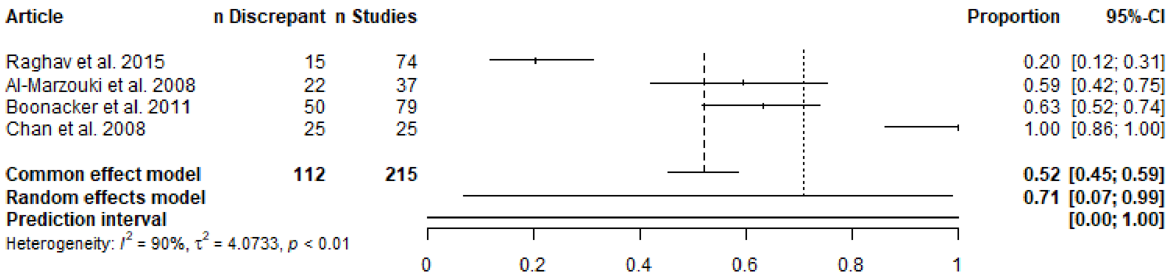

Supplementary Figure D26. Subgroup analyses, prospective.

For peer review only - http://bmjopen.bmj.com/site/about/guidelines.xhtml

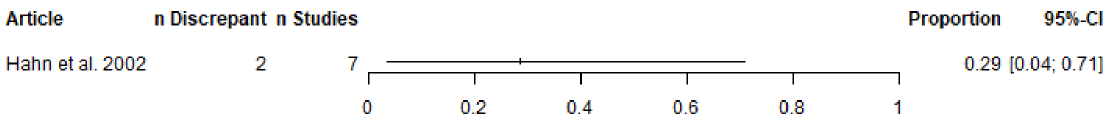

Supplementary Figure D27. Funding, prospective.

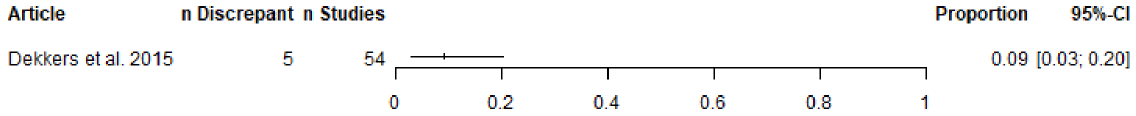

Supplementary Figure D28. Results, prospective.

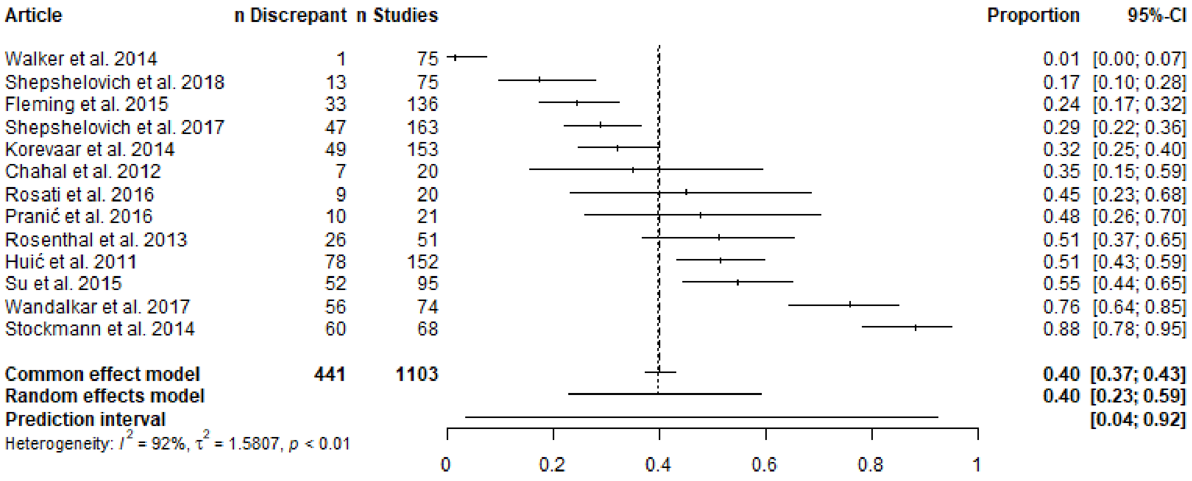

Supplementary Figure D29. Eligibility criteria, unclear registration timing.

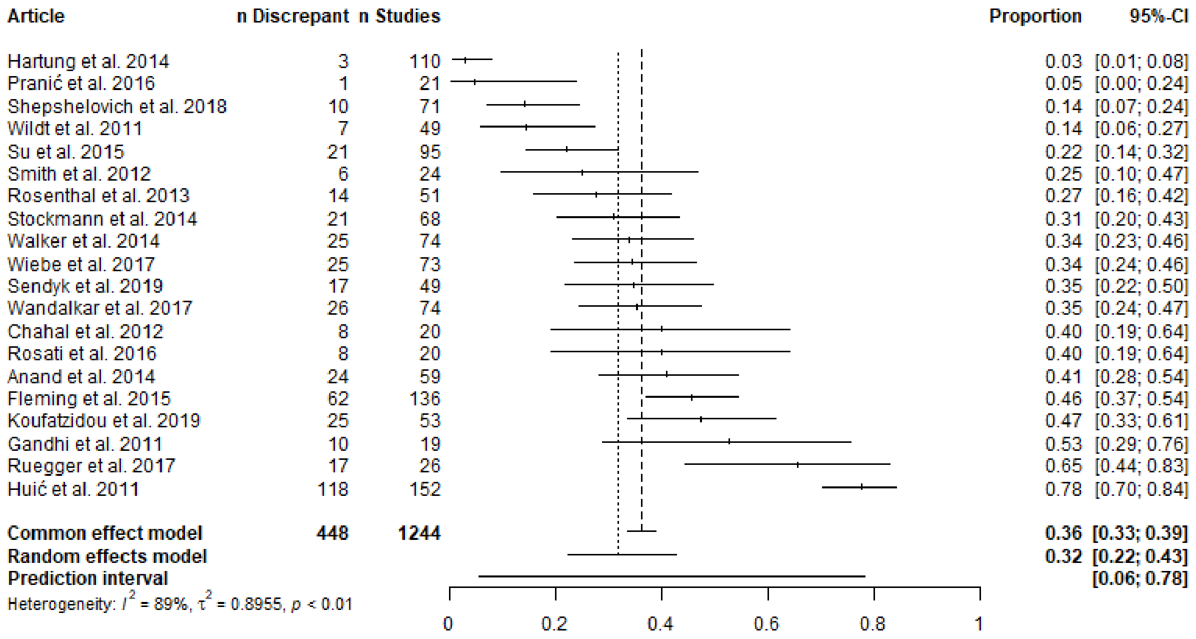

Supplementary Figure D30. Sample size, unclear registration timing.

For peer review only - http://bmjopen.bmj.com/site/about/guidelines.xhtml

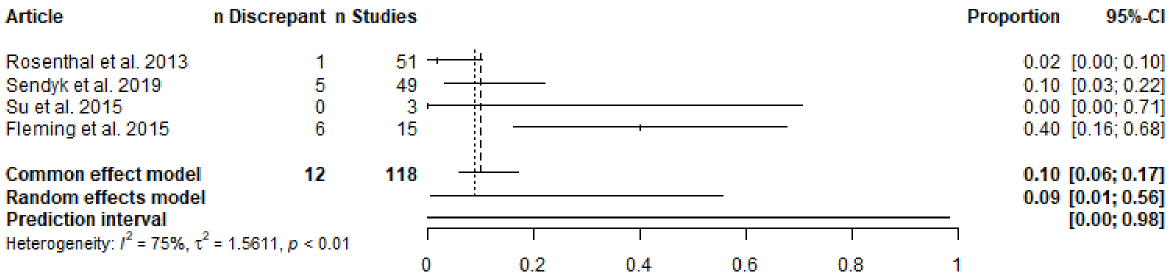

Supplementary Figure D31. Randomization, unclear registration timing.

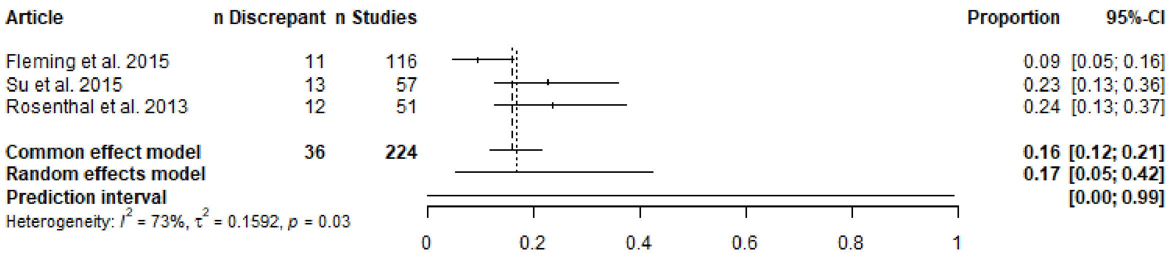

Supplementary Figure D32. Blinding, unclear registration timing.

For peer review only - http://bmjopen.bmj.com/site/about/guidelines.xhtml

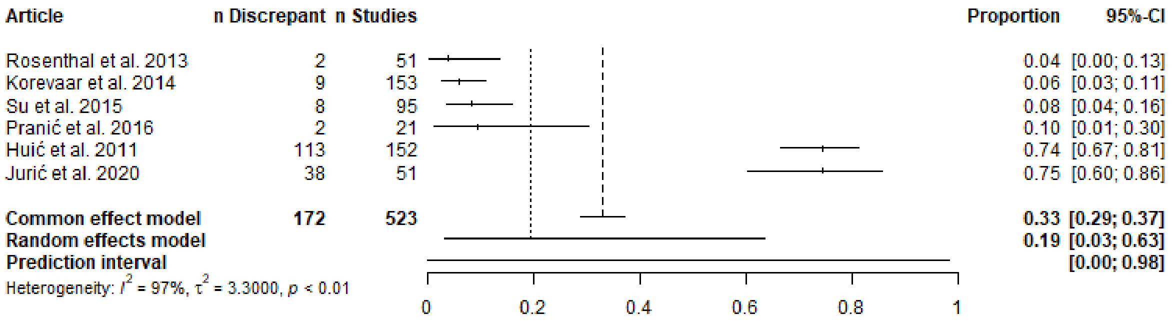

Supplementary Figure D33. Intervention, unclear registration timing.

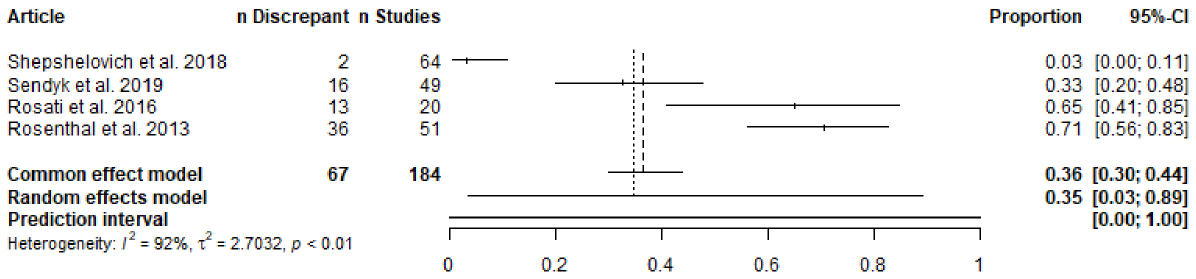

Supplementary Figure D34. Study duration, unclear registration timing.

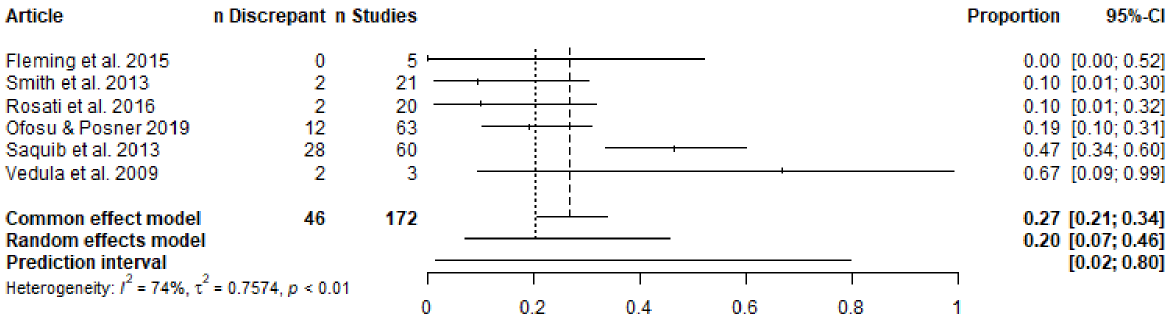

Supplementary Figure D35. Analyses, unclear registration timing.

For peer review only - http://bmjopen.bmj.com/site/about/guidelines.xhtml

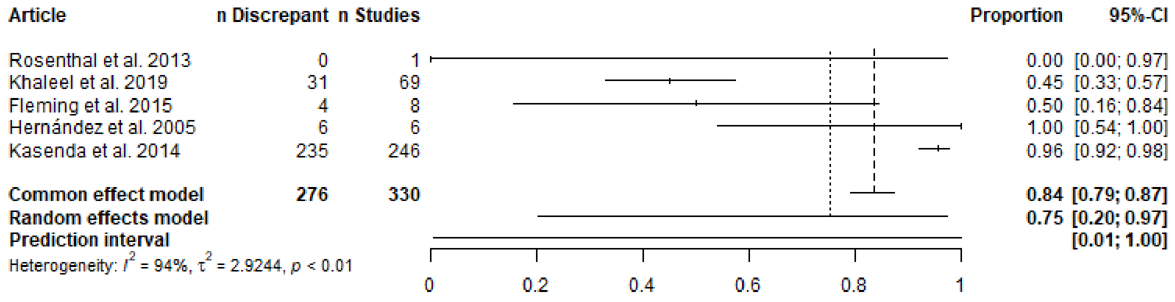

Supplementary Figure D36. Subgroup analyses, unclear registration timing.

For peer review only - http://bmjopen.bmj.com/site/about/guidelines.xhtml

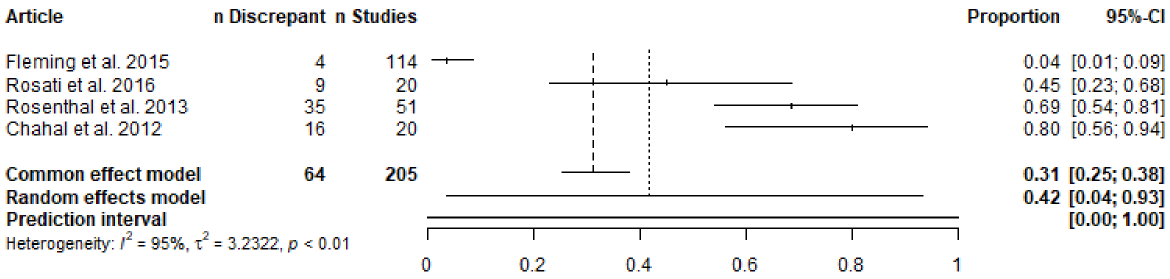

Supplementary Figure D37. Funding, unclear registration timing.

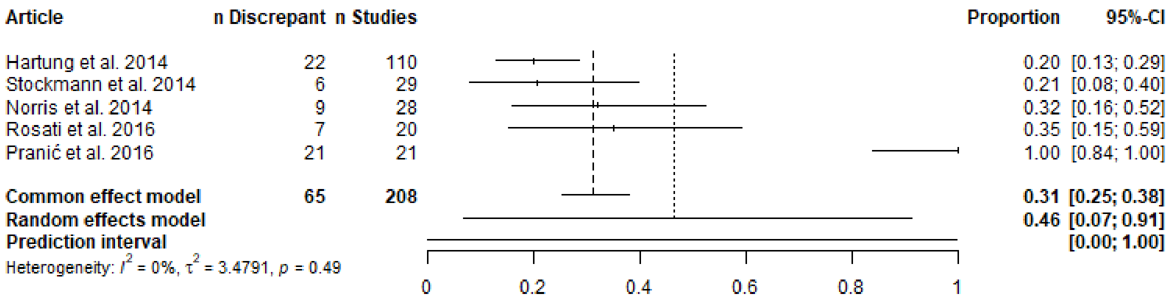

Supplementary Figure D38. Results, unclear registration timing.

E. Meta-regression model summaries

Meta-regression sensitivity analysis

We ran the meta-regressions presented in the manuscript with one predictor at a time. In line with a suggestion from a reviewer, we also ran a meta-regression with all predictors at the same time. We had no a priori thoughts on how the predictors may interact and thus did include the interaction of predictors in our meta-regressions.

This meta-regression revealed 6 predictors with $p < 0.10$. The full output is appended below. Five of the predictors include between 1 to 4 articles in one category and thus may stem from idiosyncrasies of one or more individual studies, rather than from the predictor itself. As Cochrane advises against meta-regression when $k < 10$ (https://training.cochrane.org/handbook/current/chapter-13#section-13-3-5-6), we do not explore these findings further.

This meta-regression revealed one predictor of potential interest. Articles that compared publications to the most recent version of a registration ($k = 15$) may be less likely to have at least one primary outcome discrepancy than articles that compared publications to the original version of a registration ($k = 29$) ($p = 0.09$; OR 95% CI: 0.32-1.07).

```
##
## Mixed-Effects Model (k = 70; tau^2 estimator: ML)
##
## tau^2 (estimated amount of residual heterogeneity):     0.3664
## tau (square root of estimated tau^2 value):             0.6053
## I^2 (residual heterogeneity / unaccounted variability): 86.00%
## H^2 (unaccounted variability / sampling variability):   7.14
##
## Tests for Residual Heterogeneity:
## Wld(df = 50) = 342.2743, p-val < .0001
## LRT(df = 50) = 458.3887, p-val < .0001
##
## Test of Moderators (coefficients 2:20):
## F(df1 = 19, df2 = 50) = 1.3420, p-val = 0.2007
##
## Model Results:
##
##                                                             estimate
## intrcpt                                                     -42.8460
## df1$pubYear                                                   0.0210
## df1$disciplineDentistry (DENT)                                0.1016
## df1$disciplineEconomics, Econometrics and Finance (ECON)      0.4086
## df1$disciplineHealth Professions (HEAL)                       1.4988
## df1$disciplinePsychology (PSYC)                               1.2335
## df1$sourceOther: ethics committee                             0.4129
## df1$sourceOther: funder                                       2.4149
## df1$sourcereg                                                -0.3632
## df1$sourcereg,journal                                         0.6233
## df1$sourcereg,searchEngine                                   -0.8627
## df1$sourcesearchEngine                                       -0.2628
## df1$versionMost recent version                               -0.5331
## df1$versionNo                                                -0.1020
## df1$versionOther                                             -0.2049
## df1$disclosedexcludedDisclosedDiscrepancies                   0.3049
## df1$disclosedNo                                               0.0273
```


For peer review only - http://bmjopen.bmj.com/site/about/guidelines.xhtml

```
## df1$disclosedYes, and NONE of the discrepancies were disclosed    0.2042
## df1$comparatorgrant                                               -2.3269
## df1$comparatorprotocol                                            -0.9981
##                                                                       se
## intrcpt                                                          62.0366
## df1$pubYear                                                       0.0308
## df1$disciplineDentistry (DENT)                                    0.4287
## df1$disciplineEconomics, Econometrics and Finance (ECON)          0.7221
## df1$disciplineHealth Professions (HEAL)                           0.7510
## df1$disciplinePsychology (PSYC)                                   0.6494
## df1$sourceOther: ethics committee                                 0.5356
## df1$sourceOther: funder                                           1.0151
## df1$sourcereg                                                     0.2454
## df1$sourcereg,journal                                             0.7028
## df1$sourcereg,searchEngine                                        0.7230
## df1$sourcesearchEngine                                            0.2496
## df1$versionMost recent version                                    0.3005
## df1$versionNo                                                     0.2152
## df1$versionOther                                                  0.3291
## df1$disclosedexcludedDisclosedDiscrepancies                       0.4616
## df1$disclosedNo                                                   0.2301
## df1$disclosedYes, and NONE of the discrepancies were disclosed    0.3577
## df1$comparatorgrant                                               1.2765
## df1$comparatorprotocol                                            0.4604
##                                                                     tval  df
## intrcpt                                                          -0.6907  50
## df1$pubYear                                                       0.6828  50
## df1$disciplineDentistry (DENT)                                    0.2370  50
## df1$disciplineEconomics, Econometrics and Finance (ECON)          0.5659  50
## df1$disciplineHealth Professions (HEAL)                           1.9957  50
## df1$disciplinePsychology (PSYC)                                   1.8993  50
## df1$sourceOther: ethics committee                                 0.7709  50
## df1$sourceOther: funder                                           2.3789  50
## df1$sourcereg                                                    -1.4799  50
## df1$sourcereg,journal                                             0.8869  50
## df1$sourcereg,searchEngine                                       -1.1933  50
## df1$sourcesearchEngine                                           -1.0527  50
## df1$versionMost recent version                                   -1.7741  50
## df1$versionNo                                                    -0.4740  50
## df1$versionOther                                                 -0.6224  50
## df1$disclosedexcludedDisclosedDiscrepancies                       0.6604  50
## df1$disclosedNo                                                   0.1188  50
## df1$disclosedYes, and NONE of the discrepancies were disclosed    0.5709  50
## df1$comparatorgrant                                              -1.8229  50
## df1$comparatorprotocol                                           -2.1680  50
##                                                                    pval
## intrcpt                                                          0.4930
## df1$pubYear                                                      0.4979
## df1$disciplineDentistry (DENT)                                   0.8136
## df1$disciplineEconomics, Econometrics and Finance (ECON)         0.5740
## df1$disciplineHealth Professions (HEAL)                          0.0514
## df1$disciplinePsychology (PSYC)                                  0.0633
## df1$sourceOther: ethics committee                                0.4444
## df1$sourceOther: funder                                          0.0212
```


For peer review only - http://bmjopen.bmj.com/site/about/guidelines.xhtml

```
## df1$sourcereg                                              0.1452
## df1$sourcereg,journal                                      0.3794
## df1$sourcereg,searchEngine                                 0.2384
## df1$sourcesearchEngine                                     0.2975
## df1$versionMost recent version                             0.0821
## df1$versionNo                                              0.6376
## df1$versionOther                                           0.5365
## df1$disclosedexcludedDisclosedDiscrepancies                0.5120
## df1$disclosedNo                                            0.9059
## df1$disclosedYes, and NONE of the discrepancies were disclosed  0.5706
## df1$comparatorgrant                                        0.0743
## df1$comparatorprotocol                                     0.0349
##                                                               ci.lb
## intrcpt                                                   -167.4502
## df1$pubYear                                                 -0.0408
## df1$disciplineDentistry (DENT)                              -0.7595
## df1$disciplineEconomics, Econometrics and Finance (ECON)    -1.0417
## df1$disciplineHealth Professions (HEAL)                     -0.0096
## df1$disciplinePsychology (PSYC)                             -0.0710
## df1$sourceOther: ethics committee                           -0.6629
## df1$sourceOther: funder                                      0.3760
## df1$sourcereg                                               -0.8561
## df1$sourcereg,journal                                       -0.7882
## df1$sourcereg,searchEngine                                  -2.3149
## df1$sourcesearchEngine                                      -0.7641
## df1$versionMost recent version                              -1.1366
## df1$versionNo                                               -0.5343
## df1$versionOther                                            -0.8659
## df1$disclosedexcludedDisclosedDiscrepancies                 -0.6224
## df1$disclosedNo                                             -0.4348
## df1$disclosedYes, and NONE of the discrepancies were disclosed  -0.5142
## df1$comparatorgrant                                         -4.8908
## df1$comparatorprotocol                                      -1.9227
##                                                            ci.ub
## intrcpt                                                  81.7581
## df1$pubYear                                               0.0829
## df1$disciplineDentistry (DENT)                            0.9626
## df1$disciplineEconomics, Econometrics and Finance (ECON)  1.8589
## df1$disciplineHealth Professions (HEAL)                   3.0072  .
## df1$disciplinePsychology (PSYC)                           2.5379  .
## df1$sourceOther: ethics committee                         1.4888
## df1$sourceOther: funder                                   4.4538  *
## df1$sourcereg                                             0.1297
## df1$sourcereg,journal                                     2.0348
## df1$sourcereg,searchEngine                                0.5895
## df1$sourcesearchEngine                                    0.2386
## df1$versionMost recent version                            0.0704  .
## df1$versionNo                                             0.3303
## df1$versionOther                                          0.4562
## df1$disclosedexcludedDisclosedDiscrepancies               1.2321
## df1$disclosedNo                                           0.4895
## df1$disclosedYes, and NONE of the discrepancies were disclosed  0.9227
## df1$comparatorgrant                                       0.2370  .
## df1$comparatorprotocol                                   -0.0734  *
```


For peer review only - http://bmjopen.bmj.com/site/about/guidelines.xhtml

```
##
## ---
## Signif. codes:  0 '***' 0.001 '**' 0.01 '*' 0.05 '.' 0.1 ' ' 1
```

Discipline, primary outcomes

```
##
## Mixed-Effects Model (k = 70; tau^2 estimator: ML)
##
## tau^2 (estimated amount of residual heterogeneity):     0.4973
## tau (square root of estimated tau^2 value):             0.7052
## I^2 (residual heterogeneity / unaccounted variability): 89.80%
## H^2 (unaccounted variability / sampling variability):   9.80
##
## Tests for Residual Heterogeneity:
## Wld(df = 65) = 484.2655, p-val < .0001
## LRT(df = 65) = 619.5987, p-val < .0001
##
## Test of Moderators (coefficients 2:5):
## F(df1 = 4, df2 = 65) = 1.3093, p-val = 0.2759
##
## Model Results:
##
##                                                           estimate      se
## intrcpt                                                    -0.7695  0.0982
## df1$disciplineDentistry (DENT)                              0.1419  0.4466
## df1$disciplineEconomics, Econometrics and Finance (ECON)    0.3052  0.7434
## df1$disciplineHealth Professions (HEAL)                     0.9665  0.7569
## df1$disciplinePsychology (PSYC)                             1.1512  0.6119
##                                                              tval  df    pval
## intrcpt                                                   -7.8374  65  <.0001
## df1$disciplineDentistry (DENT)                             0.3178  65  0.7517
## df1$disciplineEconomics, Econometrics and Finance (ECON)   0.4106  65  0.6827
## df1$disciplineHealth Professions (HEAL)                    1.2768  65  0.2062
## df1$disciplinePsychology (PSYC)                            1.8813  65  0.0644
##                                                             ci.lb    ci.ub
## intrcpt                                                   -0.9656  -0.5734  ***
## df1$disciplineDentistry (DENT)                            -0.7501   1.0339
## df1$disciplineEconomics, Econometrics and Finance (ECON)  -1.1795   1.7900
## df1$disciplineHealth Professions (HEAL)                   -0.5452   2.4782
## df1$disciplinePsychology (PSYC)                           -0.0709   2.3733    .
##
## ---
## Signif. codes:  0 '***' 0.001 '**' 0.01 '*' 0.05 '.' 0.1 ' ' 1
```


For peer review only - http://bmjopen.bmj.com/site/about/guidelines.xhtml

Comparator, primary outcomes

```
##
## Mixed-Effects Model (k = 70; tau^2 estimator: ML)
##
## tau^2 (estimated amount of residual heterogeneity):     0.5031
## tau (square root of estimated tau^2 value):             0.7093
## I^2 (residual heterogeneity / unaccounted variability): 89.96%
## H^2 (unaccounted variability / sampling variability):   9.96
##
## Tests for Residual Heterogeneity:
## Wld(df = 66) = 439.1139, p-val < .0001
## LRT(df = 66) = 546.7582, p-val < .0001
##
## Test of Moderators (coefficients 2:4):
## F(df1 = 3, df2 = 66) = 0.8637, p-val = 0.4644
##
## Model Results:
##
##                                       estimate      se     tval  df    pval
## intrcpt                                -0.6892  0.0989  -6.9690  66  <.0001
## droplevels(df1$comparator)ethics        0.1085  0.4982   0.2177  66  0.8283
## droplevels(df1$comparator)grant         0.2585  0.7757   0.3333  66  0.7400
## droplevels(df1$comparator)protocol     -0.6395  0.4155  -1.5389  66  0.1286
##                                         ci.lb    ci.ub
## intrcpt                               -0.8866  -0.4917  ***
## droplevels(df1$comparator)ethics      -0.8862   1.1031
## droplevels(df1$comparator)grant       -1.2902   1.8072
## droplevels(df1$comparator)protocol    -1.4691   0.1902
##
## ---
## Signif. codes:  0 '***' 0.001 '**' 0.01 '*' 0.05 '.' 0.1 ' ' 1
```


For peer review only - http://bmjopen.bmj.com/site/about/guidelines.xhtml

Source, primary outcomes

```
##
## Mixed-Effects Model (k = 70; tau^2 estimator: ML)
##
## tau^2 (estimated amount of residual heterogeneity):     0.5197
## tau (square root of estimated tau^2 value):             0.7209
## I^2 (residual heterogeneity / unaccounted variability): 89.95%
## H^2 (unaccounted variability / sampling variability):   9.95
##
## Tests for Residual Heterogeneity:
## Wld(df = 63) = 491.6691, p-val < .0001
## LRT(df = 63) = 629.6278, p-val < .0001
##
## Test of Moderators (coefficients 2:7):
## F(df1 = 6, df2 = 63) = 0.6989, p-val = 0.6515
##
## Model Results:
##
##                                                 estimate      se     tval  df
## intrcpt                                          -0.6406  0.1456  -4.3983  63
## droplevels(df1$source)Other: ethics committee    0.0578  0.5158   0.1121  63
## droplevels(df1$source)Other: funder              0.6779  0.6178   1.0973  63
## droplevels(df1$source)reg                       -0.3005  0.2346  -1.2808  63
## droplevels(df1$source)reg,journal                0.1826  0.7542   0.2421  63
## droplevels(df1$source)reg,searchEngine          -0.6840  0.7832  -0.8734  63
## droplevels(df1$source)searchEngine              -0.0312  0.2515  -0.1239  63
##                                                   pval    ci.lb    ci.ub
## intrcpt                                          <.0001  -0.9316  -0.3495  ***
## droplevels(df1$source)Other: ethics committee   0.9111  -0.9729   1.0886
## droplevels(df1$source)Other: funder             0.2767  -0.5566   1.9124
## droplevels(df1$source)reg                       0.2050  -0.7693   0.1684
## droplevels(df1$source)reg,journal               0.8095  -1.3245   1.6897
## droplevels(df1$source)reg,searchEngine          0.3858  -2.2491   0.8810
## droplevels(df1$source)searchEngine              0.9018  -0.5338   0.4715
##
## ---
## Signif. codes:  0 '***' 0.001 '**' 0.01 '*' 0.05 '.' 0.1 ' ' 1
```

For peer review only

For peer review only - http://bmjopen.bmj.com/site/about/guidelines.xhtml

Version, primary outcomes

```
##
## Mixed-Effects Model (k = 70; tau^2 estimator: ML)
##
## tau^2 (estimated amount of residual heterogeneity):     0.5251
## tau (square root of estimated tau^2 value):             0.7247
## I^2 (residual heterogeneity / unaccounted variability): 90.05%
## H^2 (unaccounted variability / sampling variability):   10.05
##
## Tests for Residual Heterogeneity:
## Wld(df = 66) = 485.3920, p-val < .0001
## LRT(df = 66) = 612.4631, p-val < .0001
##
## Test of Moderators (coefficients 2:4):
## F(df1 = 3, df2 = 66) = 0.3719, p-val = 0.7735
##
## Model Results:
##
##                                  estimate      se     tval  df    pval    ci.lb
## intrcpt                           -0.6048  0.1599  -3.7832  66  0.0003  -0.9239
## df1$versionMost recent version    -0.2477  0.2642  -0.9376  66  0.3519  -0.7752
## df1$versionNo                     -0.1046  0.2299  -0.4551  66  0.6505  -0.5636
## df1$versionOther                  -0.2433  0.3301  -0.7372  66  0.4636  -0.9023
##                                    ci.ub
## intrcpt                          -0.2856  ***
## df1$versionMost recent version    0.2798
## df1$versionNo                     0.3543
## df1$versionOther                  0.4157
##
## ---
## Signif. codes:  0 '***' 0.001 '**' 0.01 '*' 0.05 '.' 0.1 ' ' 1
```


For peer review only - http://bmjopen.bmj.com/site/about/guidelines.xhtml

Disclosed, primary outcomes

```
##
## Mixed-Effects Model (k = 70; tau^2 estimator: ML)
##
## tau^2 (estimated amount of residual heterogeneity):     0.5403
## tau (square root of estimated tau^2 value):             0.7350
## I^2 (residual heterogeneity / unaccounted variability): 90.43%
## H^2 (unaccounted variability / sampling variability):   10.45
##
## Tests for Residual Heterogeneity:
## Wld(df = 66) = 494.1488, p-val < .0001
## LRT(df = 66) = 644.7748, p-val < .0001
##
## Test of Moderators (coefficients 2:4):
## F(df1 = 3, df2 = 66) = 0.0739, p-val = 0.9738
##
## Model Results:
##
##                                                                 estimate
## intrcpt                                                          -0.7709
## df1$disclosedexcludedDisclosedDiscrepancies                       0.2332
## df1$disclosedNo                                                   0.0600
## df1$disclosedYes, and NONE of the discrepancies were disclosed    0.0611
##                                                                     se     tval
## intrcpt                                                         0.2012  -3.8314
## df1$disclosedexcludedDisclosedDiscrepancies                     0.5094   0.4578
## df1$disclosedNo                                                 0.2362   0.2541
## df1$disclosedYes, and NONE of the discrepancies were disclosed  0.3419   0.1788
##                                                                 df    pval
## intrcpt                                                         66  0.0003
## df1$disclosedexcludedDisclosedDiscrepancies                     66  0.6486
## df1$disclosedNo                                                 66  0.8002
## df1$disclosedYes, and NONE of the discrepancies were disclosed  66  0.8587
##                                                                   ci.lb
## intrcpt                                                         -1.1725
## df1$disclosedexcludedDisclosedDiscrepancies                     -0.7838
## df1$disclosedNo                                                 -0.4115
## df1$disclosedYes, and NONE of the discrepancies were disclosed  -0.6215
##                                                                   ci.ub
## intrcpt                                                         -0.3692  ***
## df1$disclosedexcludedDisclosedDiscrepancies                      1.2502
## df1$disclosedNo                                                  0.5315
## df1$disclosedYes, and NONE of the discrepancies were disclosed   0.7437
##
## ---
## Signif. codes:  0 '***' 0.001 '**' 0.01 '*' 0.05 '.' 0.1 ' ' 1
```


For peer review only - http://bmjopen.bmj.com/site/about/guidelines.xhtml

Publication year, primary outcomes

```
##
## Mixed-Effects Model (k = 70; tau^2 estimator: ML)
##
## tau^2 (estimated amount of residual heterogeneity):     0.5405
## tau (square root of estimated tau^2 value):             0.7352
## I^2 (residual heterogeneity / unaccounted variability): 90.44%
## H^2 (unaccounted variability / sampling variability):   10.46
##
## Tests for Residual Heterogeneity:
## Wld(df = 68) = 505.5885, p-val < .0001
## LRT(df = 68) = 647.2738, p-val < .0001
##
## Test of Moderators (coefficient 2):
## F(df1 = 1, df2 = 68) = 0.0438, p-val = 0.8348
##
## Model Results:
##
##                estimate       se     tval  df    pval      ci.lb    ci.ub
## intrcpt        -11.8783  53.3266  -0.2227  68  0.8244  -118.2899  94.5332
## df1$pubYear      0.0055   0.0265   0.2093  68  0.8348    -0.0473   0.0584
##
## ---
## Signif. codes:  0 '***' 0.001 '**' 0.01 '*' 0.05 '.' 0.1 ' ' 1
```

For peer review only

Discpline binary, primary outcomes

```
##
## Mixed-Effects Model (k = 70; tau^2 estimator: ML)
##
## tau^2 (estimated amount of residual heterogeneity):     0.5175
## tau (square root of estimated tau^2 value):             0.7194
## I^2 (residual heterogeneity / unaccounted variability): 90.11%
## H^2 (unaccounted variability / sampling variability):   10.11
##
## Tests for Residual Heterogeneity:
## Wld(df = 68) = 497.9543, p-val < .0001
## LRT(df = 68) = 634.4749, p-val < .0001
##
## Test of Moderators (coefficient 2):
## F(df1 = 1, df2 = 68) = 2.8890, p-val = 0.0938
##
## Model Results:
##
##                                  estimate      se     tval  df    pval    ci.lb
## intrcpt                           -0.7703  0.0999  -7.7124  68  <.0001  -0.9696
## dfDiscipline1$disciplineother      0.5342  0.3143   1.6997  68  0.0938  -0.0929
##                                    ci.ub
## intrcpt                          -0.5710  ***
## dfDiscipline1$disciplineother     1.1613    .
##
## ---
## Signif. codes:  0 '***' 0.001 '**' 0.01 '*' 0.05 '.' 0.1 ' ' 1
```


For peer review only - http://bmjopen.bmj.com/site/about/guidelines.xhtml

Comparator binary, primary outcomes

```
##
## Mixed-Effects Model (k = 70; tau^2 estimator: ML)
##
## tau^2 (estimated amount of residual heterogeneity):     0.5305
## tau (square root of estimated tau^2 value):             0.7284
## I^2 (residual heterogeneity / unaccounted variability): 90.37%
## H^2 (unaccounted variability / sampling variability):   10.38
##
## Tests for Residual Heterogeneity:
## Wld(df = 68) = 491.3839, p-val < .0001
## LRT(df = 68) = 609.5590, p-val < .0001
##
## Test of Moderators (coefficient 2):
## F(df1 = 1, df2 = 68) = 0.6521, p-val = 0.4222
##
## Model Results:
##
##                               estimate      se     tval  df    pval    ci.lb
## intrcpt                        -0.9418  0.2945  -3.1978  68  0.0021  -1.5296
## df1Comparator$comparatorreg     0.2516  0.3115   0.8076  68  0.4222  -0.3701
##                                  ci.ub
## intrcpt                        -0.3541  **
## df1Comparator$comparatorreg     0.8733
##
## ---
## Signif. codes:  0 '***' 0.001 '**' 0.01 '*' 0.05 '.' 0.1 ' ' 1
```

For peer review only

Discipline, secondary outcomes

```
##
## Mixed-Effects Model (k = 22; tau^2 estimator: ML)
##
## tau^2 (estimated amount of residual heterogeneity):     1.1358
## tau (square root of estimated tau^2 value):             1.0658
## I^2 (residual heterogeneity / unaccounted variability): 92.81%
## H^2 (unaccounted variability / sampling variability):   13.91
##
## Tests for Residual Heterogeneity:
## Wld(df = 19) = 152.4015, p-val < .0001
## LRT(df = 19) = 232.6890, p-val < .0001
##
## Test of Moderators (coefficients 2:3):
## F(df1 = 2, df2 = 19) = 1.3121, p-val = 0.2926
##
## Model Results:
##
##                                          estimate      se     tval  df    pval
## intrcpt                                    0.5830  0.2642   2.2067  19  0.0398
## df2$disciplineDentistry (DENT)            -0.9288  0.8150  -1.1396  19  0.2686
## df2$disciplineHealth Professions (HEAL)    1.2320  1.1580   1.0639  19  0.3007
##                                            ci.lb   ci.ub
## intrcpt                                   0.0300  1.1359  *
## df2$disciplineDentistry (DENT)           -2.6346  0.7771
## df2$disciplineHealth Professions (HEAL)  -1.1917  3.6557
##
## ---
## Signif. codes:  0 '***' 0.001 '**' 0.01 '*' 0.05 '.' 0.1 ' ' 1
```

For peer review only

Comparator, secondary outcomes

```
##
## Mixed-Effects Model (k = 22; tau^2 estimator: ML)
##
## tau^2 (estimated amount of residual heterogeneity):     1.1341
## tau (square root of estimated tau^2 value):             1.0650
## I^2 (residual heterogeneity / unaccounted variability): 93.39%
## H^2 (unaccounted variability / sampling variability):   15.12
##
## Tests for Residual Heterogeneity:
## Wld(df = 20) = 188.4912, p-val < .0001
## LRT(df = 20) = 268.8685, p-val < .0001
##
## Test of Moderators (coefficient 2):
## F(df1 = 1, df2 = 20) = 3.4451, p-val = 0.0782
##
## Model Results:
##
##                                       estimate      se    tval  df    pval
## intrcpt                                 0.4164  0.2535  1.6425  20  0.1161
## droplevels(df2$comparator)protocol      1.7751  0.9564  1.8561  20  0.0782
##                                         ci.lb   ci.ub
## intrcpt                               -0.1124  0.9452
## droplevels(df2$comparator)protocol    -0.2198  3.7700  .
##
## ---
## Signif. codes:  0 '***' 0.001 '**' 0.01 '*' 0.05 '.' 0.1 ' ' 1
```


For peer review only - http://bmjopen.bmj.com/site/about/guidelines.xhtml

Source, secondary outcomes

```
##
## Mixed-Effects Model (k = 22; tau^2 estimator: ML)
##
## tau^2 (estimated amount of residual heterogeneity):     0.6838
## tau (square root of estimated tau^2 value):             0.8269
## I^2 (residual heterogeneity / unaccounted variability): 88.00%
## H^2 (unaccounted variability / sampling variability):   8.33
##
## Tests for Residual Heterogeneity:
## Wld(df = 16) = 135.1594, p-val < .0001
## LRT(df = 16) = 187.0805, p-val < .0001
##
## Test of Moderators (coefficients 2:6):
## F(df1 = 5, df2 = 16) = 3.3793, p-val = 0.0284
##
## Model Results:
##
##                               estimate      se     tval  df    pval    ci.lb
## intrcpt                        -0.3517  0.3203  -1.0981  16  0.2884  -1.0306
## df2$sourcejournal               1.6186  0.4626   3.4989  16  0.0030   0.6379
## df2$sourceOther: funder         2.9203  1.4041   2.0798  16  0.0540  -0.0563
## df2$sourcereg,journal           0.9517  0.9029   1.0540  16  0.3075  -0.9624
## df2$sourcereg,searchEngine      2.1280  0.9400   2.2639  16  0.0378   0.1353
## df2$sourcesearchEngine          0.5912  0.6049   0.9774  16  0.3429  -0.6911
##                                ci.ub
## intrcpt                       0.3272
## df2$sourcejournal             2.5993  **
## df2$sourceOther: funder       5.8968   .
## df2$sourcereg,journal         2.8658
## df2$sourcereg,searchEngine    4.1206   *
## df2$sourcesearchEngine        1.8735
##
## ---
## Signif. codes:  0 '***' 0.001 '**' 0.01 '*' 0.05 '.' 0.1 ' ' 1
```

For peer review only

Version, secondary outcomes

```
##
## Mixed-Effects Model (k = 22; tau^2 estimator: ML)
##
## tau^2 (estimated amount of residual heterogeneity):     1.2281
## tau (square root of estimated tau^2 value):             1.1082
## I^2 (residual heterogeneity / unaccounted variability): 93.15%
## H^2 (unaccounted variability / sampling variability):   14.60
##
## Tests for Residual Heterogeneity:
## Wld(df = 18) = 164.5429, p-val < .0001
## LRT(df = 18) = 247.8955, p-val < .0001
##
## Test of Moderators (coefficients 2:4):
## F(df1 = 3, df2 = 18) = 0.3299, p-val = 0.8038
##
## Model Results:
##
##                                estimate      se     tval  df    pval    ci.lb
## intrcpt                          0.3831  0.4475   0.8560  18  0.4033  -0.5571
## df2$versionMost recent version   0.5515  0.6480   0.8512  18  0.4058  -0.8098
## df2$versionNo                   -0.0300  0.6500  -0.0462  18  0.9637  -1.3956
## df2$versionOther                 0.1605  0.9210   0.1743  18  0.8636  -1.7744
##                                 ci.ub
## intrcpt                        1.3233
## df2$versionMost recent version  1.9128
## df2$versionNo                  1.3356
## df2$versionOther               2.0954
##
## ---
## Signif. codes:  0 '***' 0.001 '**' 0.01 '*' 0.05 '.' 0.1 ' ' 1
```


For peer review only - http://bmjopen.bmj.com/site/about/guidelines.xhtml

Disclosed, secondary outcomes

```
##
## Mixed-Effects Model (k = 22; tau^2 estimator: ML)
##
## tau^2 (estimated amount of residual heterogeneity):     1.2148
## tau (square root of estimated tau^2 value):             1.1022
## I^2 (residual heterogeneity / unaccounted variability): 93.11%
## H^2 (unaccounted variability / sampling variability):   14.52
##
## Tests for Residual Heterogeneity:
## Wld(df = 18) = 173.0004, p-val < .0001
## LRT(df = 18) = 241.2204, p-val < .0001
##
## Test of Moderators (coefficients 2:4):
## F(df1 = 3, df2 = 18) = 0.5040, p-val = 0.6843
##
## Model Results:
##
##                                                           estimate
## intrcpt                                                     0.2683
## df2$disclosedexcludedDisclosedDiscrepancies                 0.7064
## df2$disclosedNo                                             0.3885
## df2$disclosedYes, and NONE of the discrepancies were disclosed   -0.5224
##                                                               se     tval
## intrcpt                                                   0.6643   0.4039
## df2$disclosedexcludedDisclosedDiscrepancies               1.0390   0.6799
## df2$disclosedNo                                           0.7324   0.5304
## df2$disclosedYes, and NONE of the discrepancies were disclosed 1.0664  -0.4899
##                                                           df    pval
## intrcpt                                                   18  0.6910
## df2$disclosedexcludedDisclosedDiscrepancies               18  0.5052
## df2$disclosedNo                                           18  0.6023
## df2$disclosedYes, and NONE of the discrepancies were disclosed 18  0.6301
##                                                             ci.lb   ci.ub
## intrcpt                                                   -1.1273  1.6639
## df2$disclosedexcludedDisclosedDiscrepancies               -1.4765  2.8894
## df2$disclosedNo                                           -1.1503  1.9272
## df2$disclosedYes, and NONE of the discrepancies were disclosed -2.7627  1.7179
##
## intrcpt
## df2$disclosedexcludedDisclosedDiscrepancies
## df2$disclosedNo
## df2$disclosedYes, and NONE of the discrepancies were disclosed
##
## ---
## Signif. codes:  0 '***' 0.001 '**' 0.01 '*' 0.05 '.' 0.1 ' ' 1
```


For peer review only - http://bmjopen.bmj.com/site/about/guidelines.xhtml

Publication year, secondary outcomes

```
##
## Mixed-Effects Model (k = 22; tau^2 estimator: ML)
##
## tau^2 (estimated amount of residual heterogeneity):     1.3181
## tau (square root of estimated tau^2 value):             1.1481
## I^2 (residual heterogeneity / unaccounted variability): 93.94%
## H^2 (unaccounted variability / sampling variability):   16.51
##
## Tests for Residual Heterogeneity:
## Wld(df = 20) = 199.2193, p-val < .0001
## LRT(df = 20) = 284.0615, p-val < .0001
##
## Test of Moderators (coefficient 2):
## F(df1 = 1, df2 = 20) = 0.1305, p-val = 0.7217
##
## Model Results:
##
##                estimate        se     tval  df    pval      ci.lb     ci.ub
## intrcpt        -55.6213  155.4756  -0.3577  20  0.7243  -379.9378  268.6951
## df2$pubYear      0.0279    0.0772   0.3613  20  0.7217    -0.1331    0.1889
##
## ---
## Signif. codes:  0 '***' 0.001 '**' 0.01 '*' 0.05 '.' 0.1 ' ' 1
```


For peer review only - http://bmjopen.bmj.com/site/about/guidelines.xhtml

F. Forest plots for all meta-analyses in Table 2 of the manuscript (outcome discrepancies)

For peer review only

For peer review only - http://bmjopen.bmj.com/site/about/guidelines.xhtml

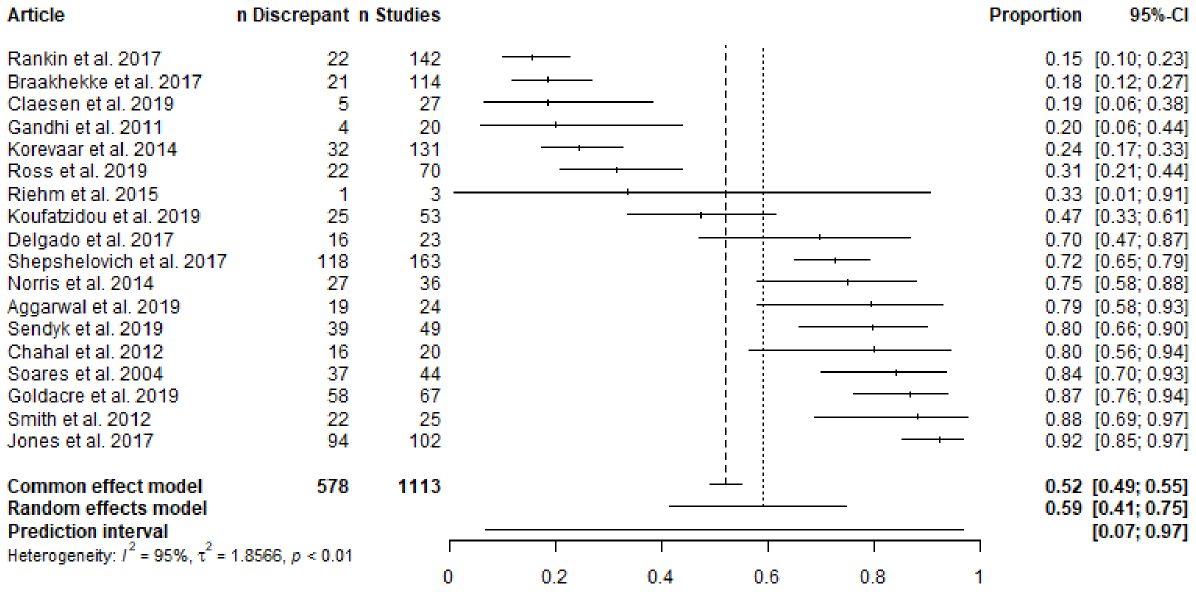

Supplementary Figure F1. Any outcome discrepancy.

Article	n Discrepant	n Studies	Proportion	95%-CI
Wildt et al. 2011	0	66	0.00	[0.00; 0.05]
Zhang et al. 2017	25	299	0.08	[0.05; 0.12]
Stockmann et al. 2014	6	68	0.09	[0.03; 0.18]
Gandhi et al. 2011	2	20	0.10	[0.01; 0.32]
Raghav et al. 2015	9	74	0.12	[0.06; 0.22]
White et al. 2019	0	3	0.00	[0.00; 0.71]
You et al. 2012	19	134	0.14	[0.09; 0.21]
Li et al. 2013	22	155	0.14	[0.09; 0.21]
Hartung et al. 2014	16	110	0.15	[0.09; 0.23]
Chan et al. 2017	9	58	0.16	[0.07; 0.27]
Khan et al. 2012	1	6	0.17	[0.00; 0.64]
Delgado et al. 2017	39	216	0.18	[0.13; 0.24]
Fleming et al. 2015	25	137	0.18	[0.12; 0.26]
Braakhekke et al. 2017	21	114	0.18	[0.12; 0.27]
Goldacre et al. 2019	13	67	0.19	[0.11; 0.31]
Koufatzidou et al. 2019	11	53	0.21	[0.11; 0.34]
Delgado et al. 2017	5	23	0.22	[0.07; 0.44]
Vera-badillo et al. 2013	7	30	0.23	[0.10; 0.42]
Thomas et al. 2016	4	16	0.25	[0.07; 0.52]
Anand et al. 2014	15	59	0.25	[0.15; 0.38]
Jones et al. 2018	27	103	0.26	[0.18; 0.36]
Shepshelovich et al. 2018	19	72	0.26	[0.17; 0.38]
Gopal et al. 2018	109	413	0.26	[0.22; 0.31]
Shinohara et al. 2015	12	43	0.28	[0.15; 0.44]
Calméjane et al. 2018	77	274	0.28	[0.23; 0.34]
Scott et al. 2015	17	60	0.28	[0.17; 0.41]
Pranić et al. 2016	6	21	0.29	[0.11; 0.52]
Aggarwal et al. 2019	7	24	0.29	[0.13; 0.51]
Killeen et al. 2014	32	108	0.30	[0.21; 0.39]
Al-Marzouki et al. 2008	11	37	0.30	[0.16; 0.47]
Smith et al. 2013	26	87	0.30	[0.21; 0.41]
Ewart et al. 2009	34	110	0.31	[0.22; 0.40]
Mathieu et al. 2009	46	147	0.31	[0.24; 0.39]
Pandis et al. 2015	49	152	0.32	[0.25; 0.40]
Tricco et al. 2016	31	96	0.32	[0.23; 0.43]
Mathieu et al. 2012	12	37	0.32	[0.18; 0.50]
Hahn et al. 2002	2	6	0.33	[0.04; 0.78]
Walker et al. 2014	25	75	0.33	[0.23; 0.45]
Chen et al. 2019	130	389	0.33	[0.29; 0.38]
Chahal et al. 2012	7	20	0.35	[0.15; 0.59]
Shepshelovich et al. 2017	62	163	0.38	[0.31; 0.46]
Ofosu & Posner 2019	36	93	0.39	[0.29; 0.49]
Huić et al. 2011	59	152	0.39	[0.31; 0.47]
Won et al. 2019	25	64	0.39	[0.27; 0.52]
Bonnot et al. 2016	34	87	0.39	[0.29; 0.50]
Dwan et al. 2013	18	46	0.39	[0.25; 0.55]
Chan et al. 2004	19	48	0.40	[0.26; 0.55]
Su et al. 2015	32	71	0.45	[0.33; 0.57]
Jones et al. 2017	46	102	0.45	[0.35; 0.55]
Rosenthal et al. 2013	23	51	0.45	[0.31; 0.60]
Jones et al. 2012	26	57	0.46	[0.32; 0.59]
Wiebe et al. 2017	34	73	0.47	[0.35; 0.59]
Krsticevic et al. 2019	77	159	0.48	[0.40; 0.56]
Wayant et al. 2017	53	109	0.49	[0.39; 0.58]
Hannink et al. 2013	75	152	0.49	[0.41; 0.58]
Howard et al. 2017	91	180	0.51	[0.43; 0.58]
Smith et al. 2012	13	25	0.52	[0.31; 0.72]
Khaleel et al. 2019	106	201	0.53	[0.46; 0.60]
Rongen et al. 2016	14	26	0.54	[0.33; 0.73]
Pinto et al. 2013	34	62	0.55	[0.42; 0.68]
Rosati et al. 2016	11	20	0.55	[0.32; 0.77]
Sendyk et al. 2019	27	49	0.55	[0.40; 0.69]
Wandalkar et al. 2017	41	74	0.55	[0.43; 0.67]
Azar et al. 2019	48	85	0.56	[0.45; 0.67]
Hildenbrand et al. 2019	16	28	0.57	[0.37; 0.76]
Bradley et al. 2017	8	13	0.62	[0.32; 0.86]
Chan et al. 2004	51	82	0.62	[0.51; 0.73]
Vedula et al. 2009	8	12	0.67	[0.35; 0.90]
Ludwig et al. 2019	20	30	0.67	[0.47; 0.83]
Nankervis et al. 2012	13	18	0.72	[0.47; 0.90]
**Common effect model**	**2048**	**6314**	**0.32**	**[0.31; 0.34]**
**Random effects model**			**0.33**	**[0.29; 0.37]**
**Prediction interval**				**[0.10; 0.68]**

Heterogeneity: $I^2 = 86\%$, $\tau^2 = 0.5407$, $p < 0.01$

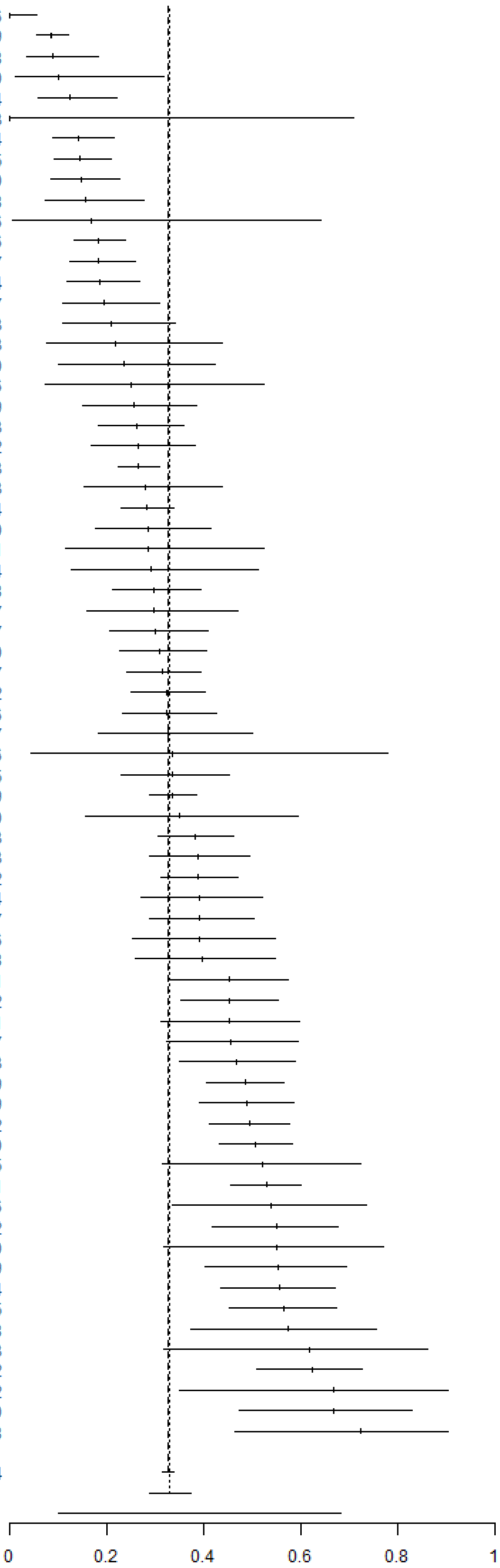

Supplementary Figure F2. Any primary outcome discrepancy.

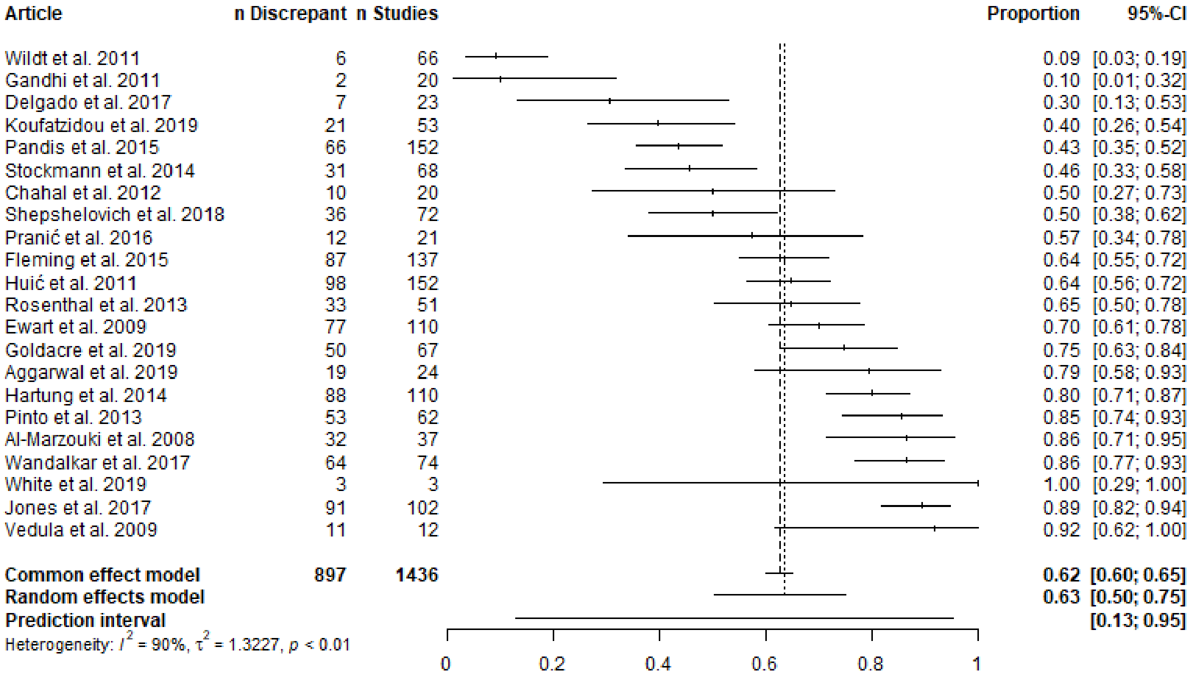

Supplementary Figure F3. Any secondary outcome discrepancy.

For peer review only - http://bmjopen.bmj.com/site/about/guidelines.xhtml

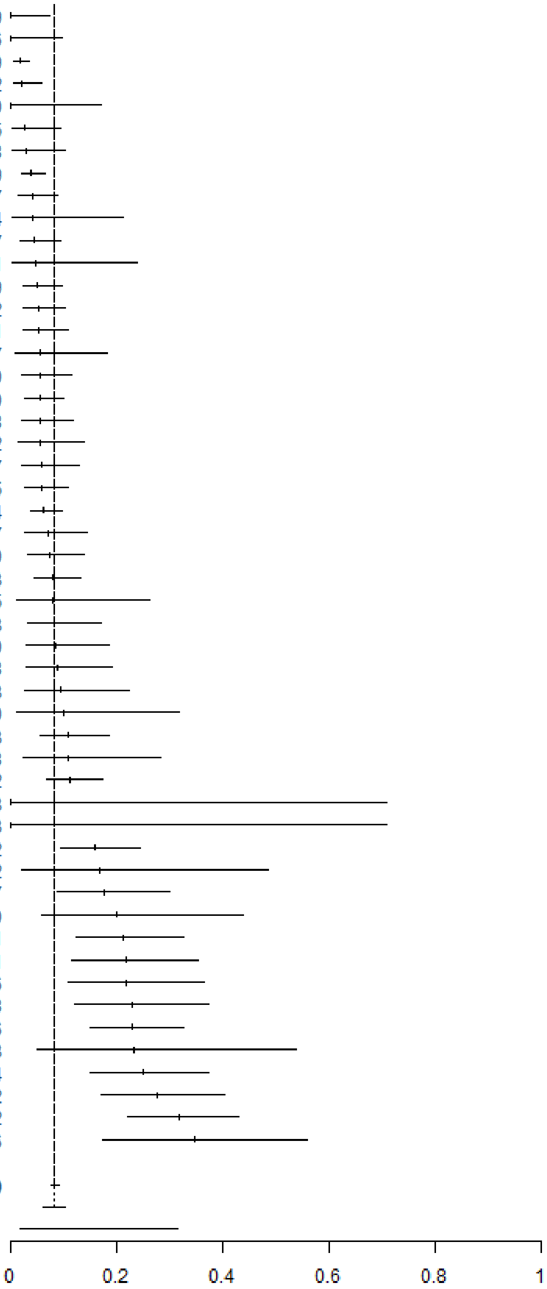

Article	n Discrepant	n Studies	Proportion	95%-CI
Sendyk et al. 2019	0	49	0.00	[0.00; 0.07]
Norris et al. 2014	0	36	0.00	[0.00; 0.10]
Chen et al. 2019	6	389	0.02	[0.01; 0.03]
Huić et al. 2011	3	152	0.02	[0.00; 0.06]
Gandhi et al. 2011	0	20	0.00	[0.00; 0.17]
Walker et al. 2014	2	75	0.03	[0.00; 0.09]
Stockmann et al. 2014	2	68	0.03	[0.00; 0.10]
Zhang et al. 2017	11	299	0.04	[0.02; 0.06]
Mathieu et al. 2009	6	147	0.04	[0.02; 0.09]
Aggarwal et al. 2019	1	24	0.04	[0.00; 0.21]
Fleming et al. 2015	6	137	0.04	[0.02; 0.09]
Pranić et al. 2016	1	21	0.05	[0.00; 0.24]
Krsticevic et al. 2019	8	159	0.05	[0.02; 0.10]
Hannink et al. 2013	8	152	0.05	[0.02; 0.10]
Korevaar et al. 2014	7	131	0.05	[0.02; 0.11]
Al-Marzouki et al. 2008	2	37	0.05	[0.01; 0.18]
Ewart et al. 2009	6	110	0.05	[0.02; 0.11]
Howard et al. 2017	10	180	0.06	[0.03; 0.10]
Killeen et al. 2014	6	108	0.06	[0.02; 0.12]
Shepshelovich et al. 2018	4	72	0.06	[0.02; 0.14]
Bonnot et al. 2016	5	87	0.06	[0.02; 0.13]
Li et al. 2013	9	155	0.06	[0.03; 0.11]
Calméjane et al. 2018	17	274	0.06	[0.04; 0.10]
Smith et al. 2013	6	87	0.07	[0.03; 0.14]
Hartung et al. 2014	8	110	0.07	[0.03; 0.14]
Shepshelovich et al. 2017	13	163	0.08	[0.04; 0.13]
Smith et al. 2012	2	25	0.08	[0.01; 0.26]
Wiebe et al. 2017	6	73	0.08	[0.03; 0.17]
Scott et al. 2015	5	60	0.08	[0.03; 0.18]
Chan et al. 2017	5	58	0.09	[0.03; 0.19]
Shinohara et al. 2015	4	43	0.09	[0.03; 0.22]
Rosati et al. 2016	2	20	0.10	[0.01; 0.32]
Jones et al. 2018	11	103	0.11	[0.05; 0.18]
Hildenbrand et al. 2019	3	28	0.11	[0.02; 0.28]
Pandis et al. 2015	17	152	0.11	[0.07; 0.17]
Riehm et al. 2015	0	3	0.00	[0.00; 0.71]
White et al. 2019	0	3	0.00	[0.00; 0.71]
Jones et al. 2017	16	102	0.16	[0.09; 0.24]
Vedula et al. 2009	2	12	0.17	[0.02; 0.48]
Jones et al. 2012	10	57	0.18	[0.09; 0.30]
Chahal et al. 2012	4	20	0.20	[0.06; 0.44]
Su et al. 2015	15	71	0.21	[0.12; 0.32]
Rosenthal et al. 2013	11	51	0.22	[0.11; 0.35]
Dwan et al. 2013	10	46	0.22	[0.11; 0.36]
Chan et al. 2004	11	48	0.23	[0.12; 0.37]
Tricco et al. 2016	22	96	0.23	[0.15; 0.33]
Bradley et al. 2017	3	13	0.23	[0.05; 0.54]
Won et al. 2019	16	64	0.25	[0.15; 0.37]
Pinto et al. 2013	17	62	0.27	[0.17; 0.40]
Chan et al. 2004	26	82	0.32	[0.22; 0.43]
Rongen et al. 2016	9	26	0.35	[0.17; 0.56]
**Common effect model**	**374**	**4560**	**0.08**	**[0.07; 0.09]**
**Random effects model**			**0.08**	**[0.06; 0.10]**
**Prediction interval**				**[0.02; 0.31]**

Heterogeneity: $I^2 = 79\%$, $\tau^2 = 0.6630$, $p < 0.01$

Supplementary Figure F4. Primary outcome demoted to secondary outcome.

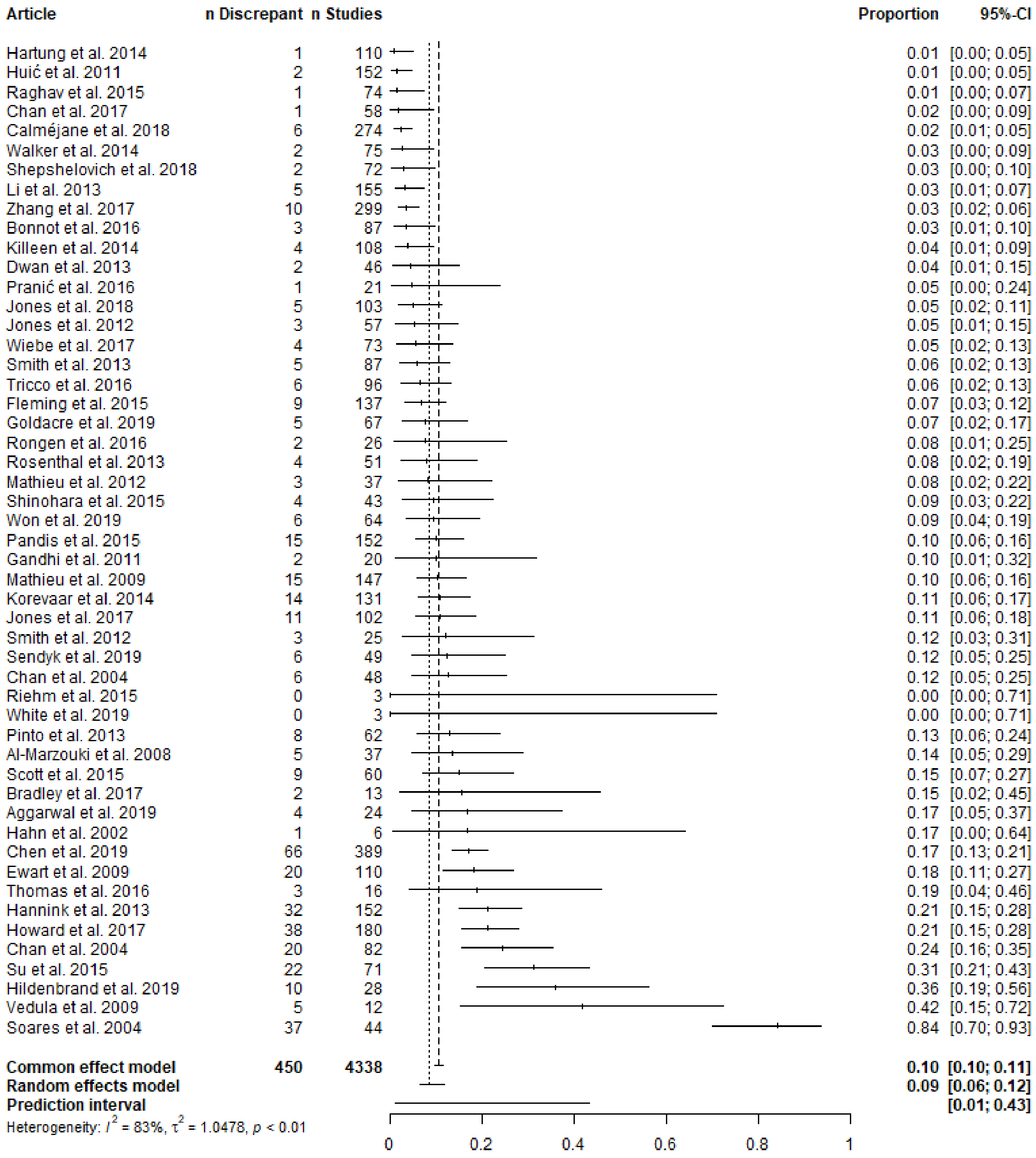

Supplementary Figure F5. Primary outcome omitted.

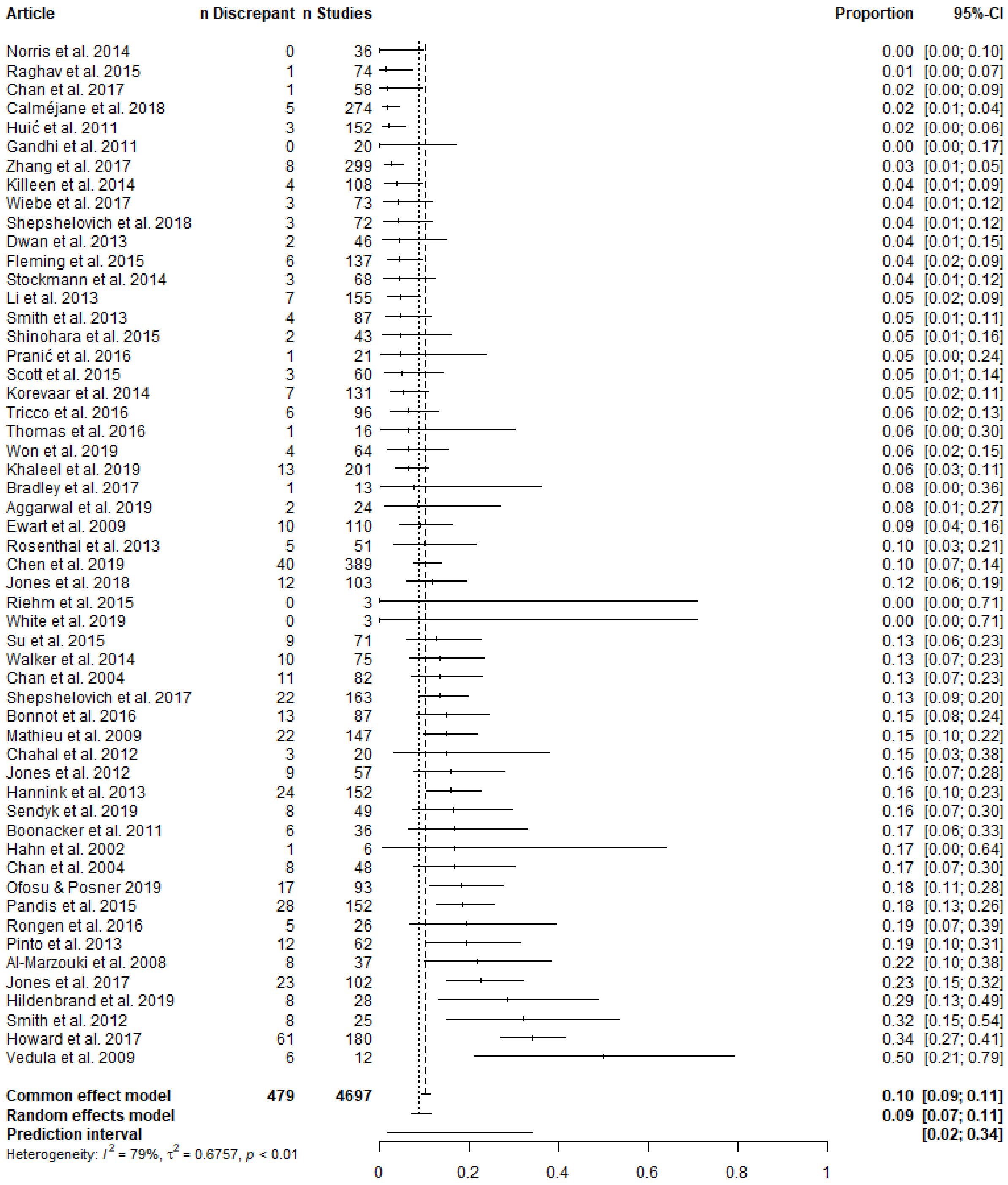

Supplementary Figure F6. Primary outcome added.

For peer review only - http://bmjopen.bmj.com/site/about/guidelines.xhtml

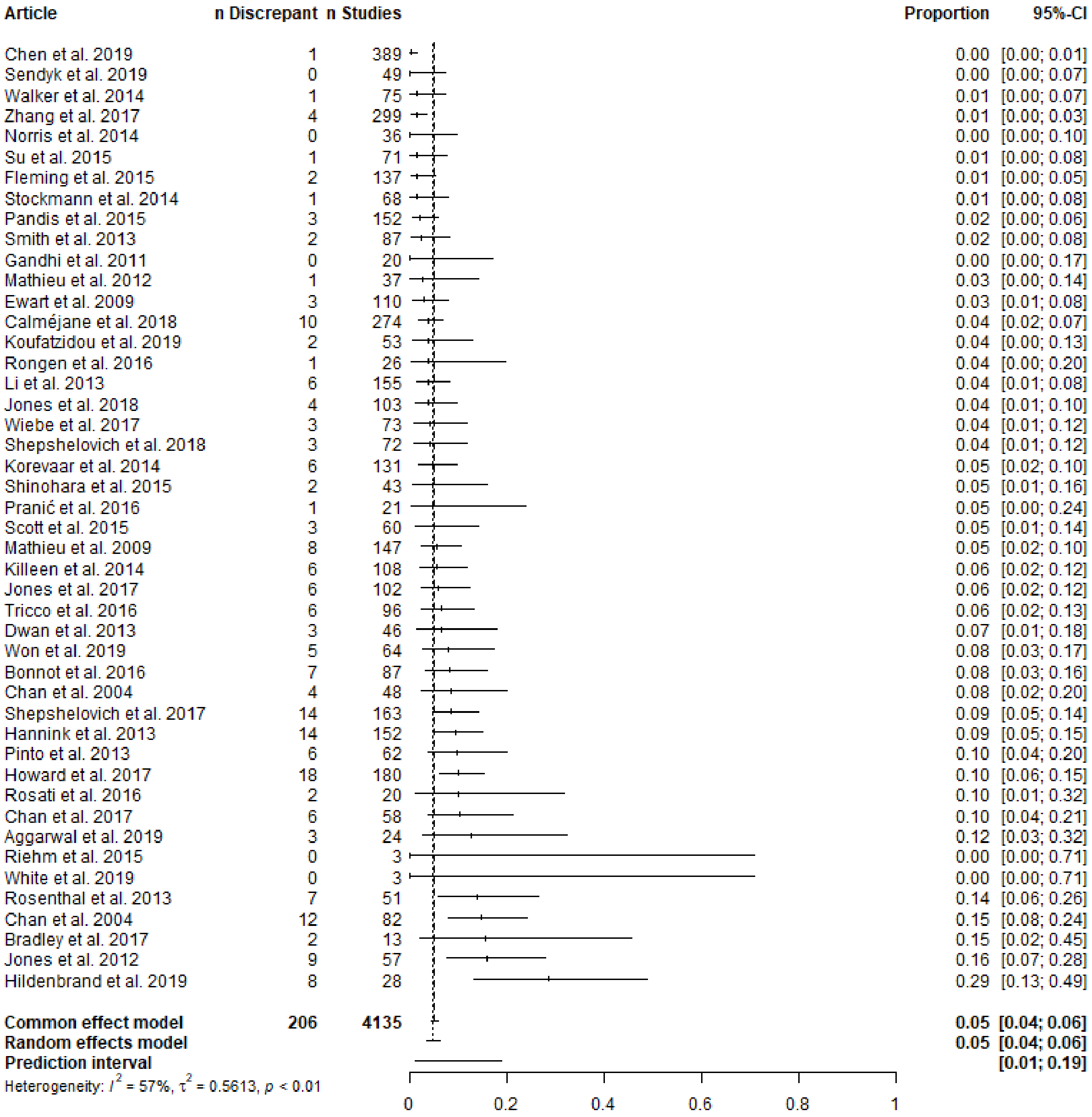

Supplementary Figure F7. Secondary outcome promoted to primary outcome.

For peer review only - http://bmjopen.bmj.com/site/about/guidelines.xhtml

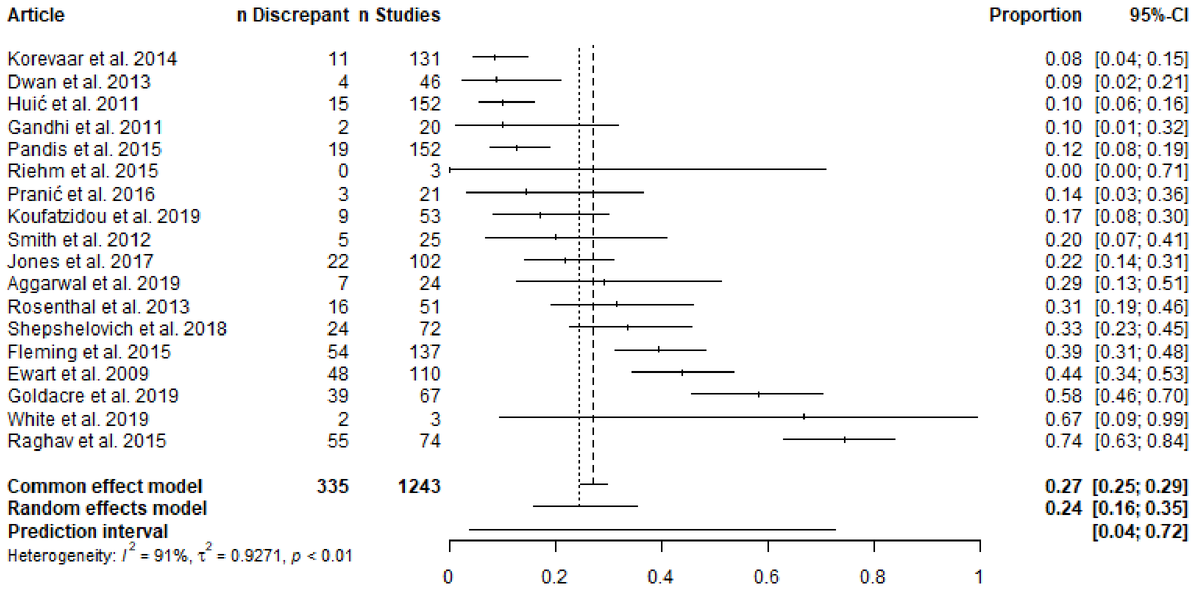

Supplementary Figure F8. Secondary outcome omitted.

For peer review only - http://bmjopen.bmj.com/site/about/guidelines.xhtml

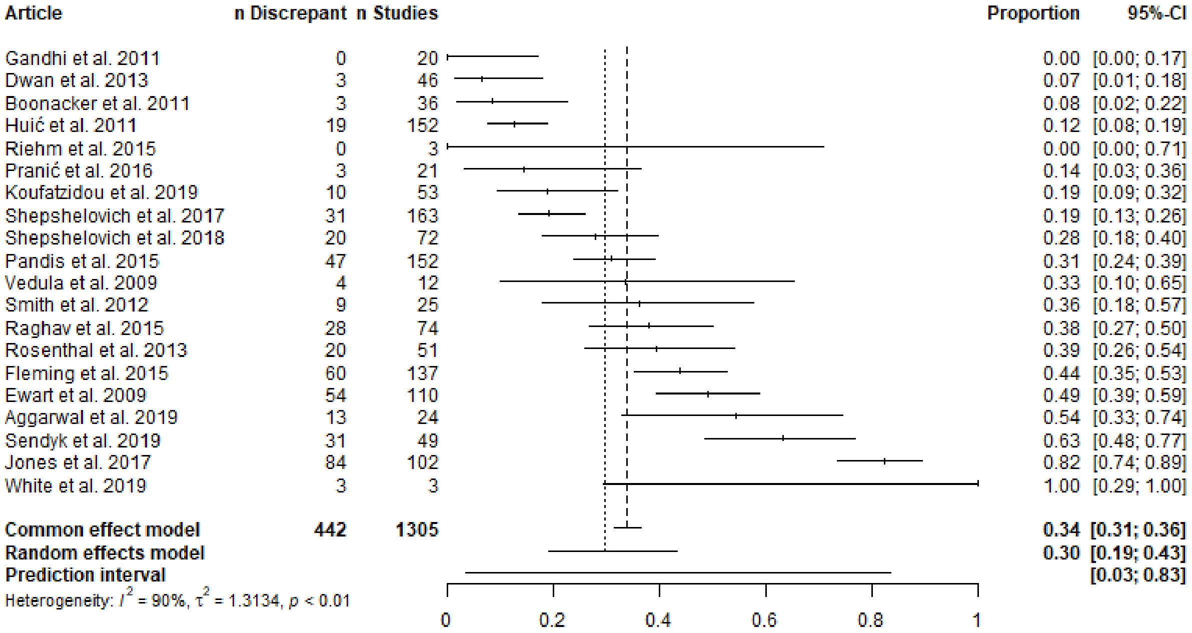

Supplementary Figure F9. Secondary outcome added.

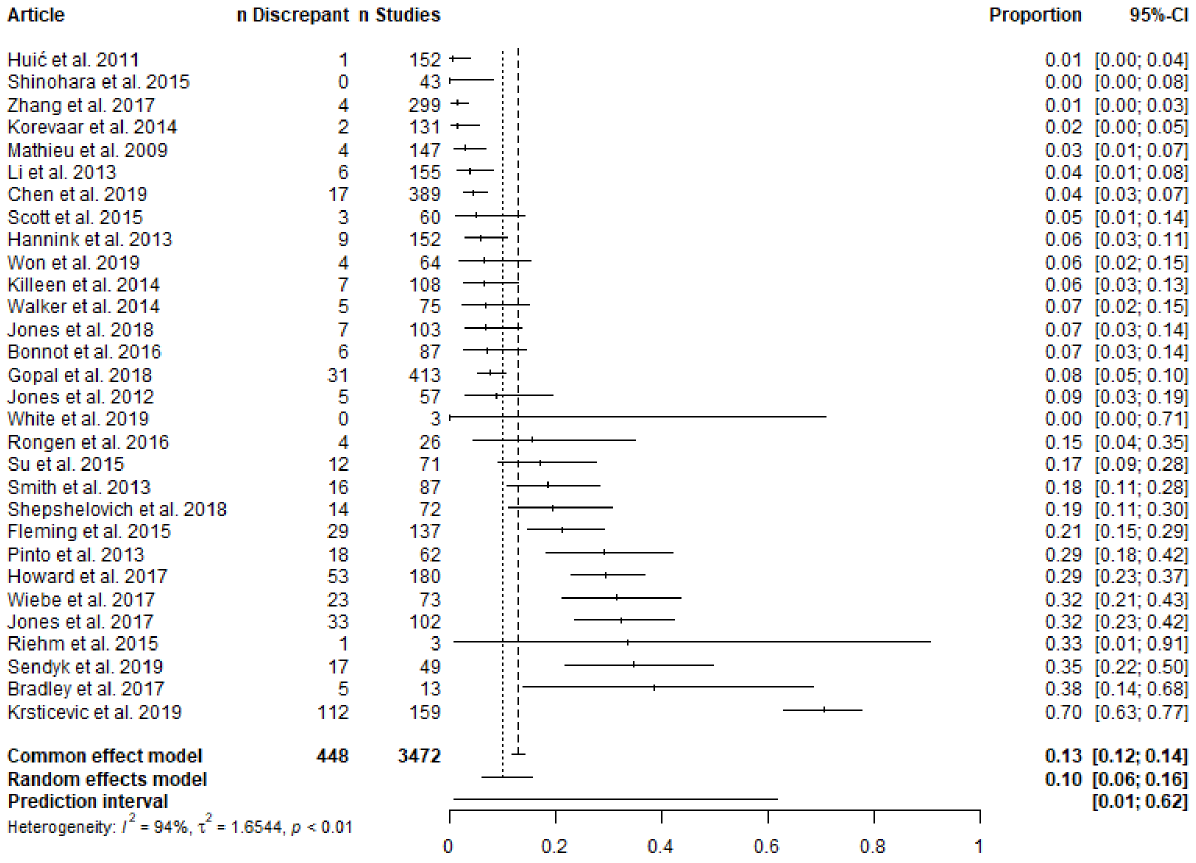

Supplementary Figure F10. Timing of outcome measurement changed.

G. Parameters potentially related to discrepancies

Disclosure of discrepancies

Some publications disclose the discrepancies between their registration or study protocol and publication. Of the articles we reviewed, k = 8 state that none of the publications disclose their primary outcome discrepancies, k = 13 state that one or more publication disclosed one or more primary outcome discrepancy, k = 68 do not report how many publications disclosed their primary outcome discrepancies, and k = 4 included only publications with undisclosed discrepancies in their sample. A random effects meta-analysis of the k = 21 articles that report the number of disclosed primary outcome discrepancies estimates that 4-19% (95% CI) (95% prediction interval: 0.3-74%) of publications with a discrepancy at least mention that a discrepancy is present (see Supplementary Figure G1 for a forest plot). Notably, a mention does not imply that the publication justified that discrepancy (e.g., Pandis et al., 2015). A few articles identified a much higher proportion of publications that disclosed discrepancies compared to the other articles. These articles focused on acupuncture (Su et al., 2015), meta-analyses (Delgado & Delgado, 2017b), and systematic reviews (Dwan, Kirkham, et al., 2013).

In our initial coding form, we identified 19 articles reporting that the studies they assessed disclosed one or more discrepancy and 9 articles reporting that the studies they assessed disclosed no discrepancies (28 articles total). Only 21 of these 28 articles reported the exact number of studies assessed and the number of discrepancies disclosed (or percentages from which we could calculate these values). Supplementary Figure G1 only includes data from those 21 articles.

Statistical significance

k = 24 articles checked whether discrepancies favored statistical significance. Of the studies for which favoring statistical significance could be determined, 49-66% (95% CI) (95% prediction interval: 23-86%) of discrepancies favored statistical significance (see Supplementary Figure G2 for a forest plot).

k = 7 articles provided information on the prevalence of discrepancies in relation to statistical significance. Studies with discrepancies may be *less* likely than studies without discrepancies to report a statistically significant result—likelihood ratio: 0.56-1.06 (95% CI) (95% prediction interval: 0.42-1.43) (see Supplementary Figure G3 for a forest plot). Likewise, rearranging the analysis on this same data shows that studies that report a statistically significant finding are *less* likely to contain a discrepancy—likelihood ratio: 0.64-0.99 (95% CI) (95% prediction interval: 0.59-1.06) (see Supplementary Figure G4 for a forest plot). Whereas these findings may appear counterintuitive, a sampling bias may influence these results—in the sense that the incentive to switch outcomes is less apparent when the registered outcome reaches statistical significance.

Industry funding

k = 22 articles provided information on the prevalence of discrepancies in relation to the type of funding a study received. A meta-analysis of these articles shows that industry funded studies are perhaps *less* likely to contain discrepancies compared to non-industry funded studies—likelihood ratio: 0.61-0.91 (95% CI) (95% prediction interval: 0.44-1.27) (see Supplementary Figure G5 for a forest plot).

Timing of registration

k = 4 articles compared whether prospectively registered studies were more likely to have primary outcome discrepancies compared to retrospectively registered studies. A meta-analysis of these studies did not find a difference—likelihood ratio: 0.46-2.57 (95% CI) (95% prediction interval: 0.13-8.85) (see Supplementary Figure G6 for a forest plot).

For peer review only - http://bmjopen.bmj.com/site/about/guidelines.xhtml

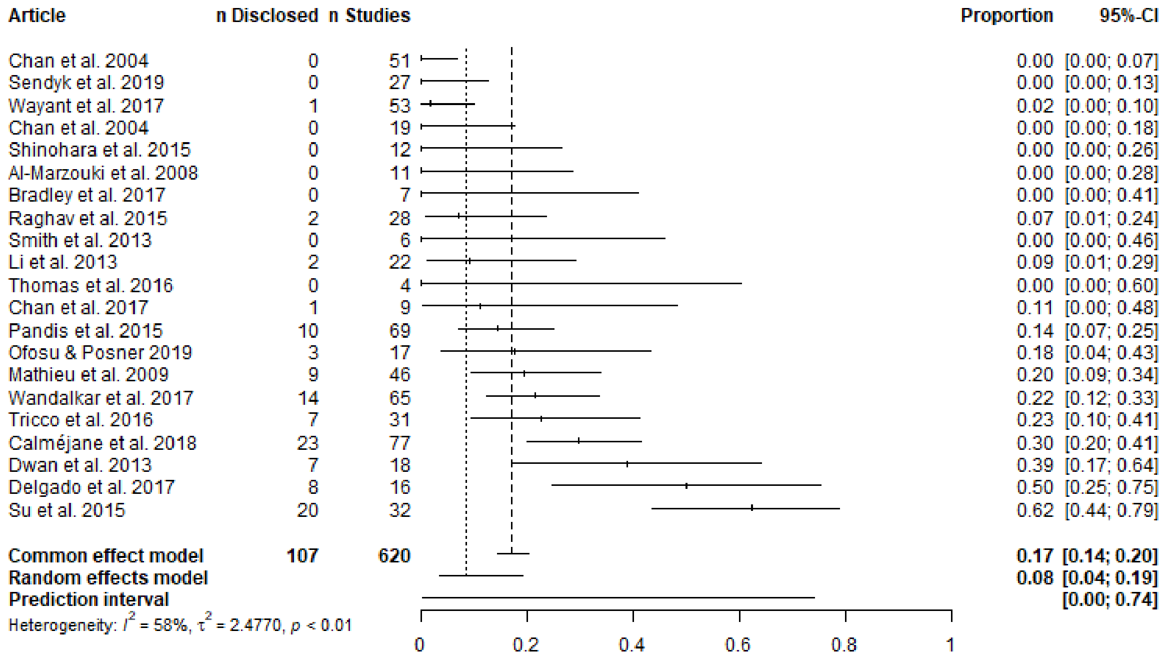

Supplementary Figure G1. Proportion of studies with at least one outcome discrepancy that also disclose an outcome discrepancy.

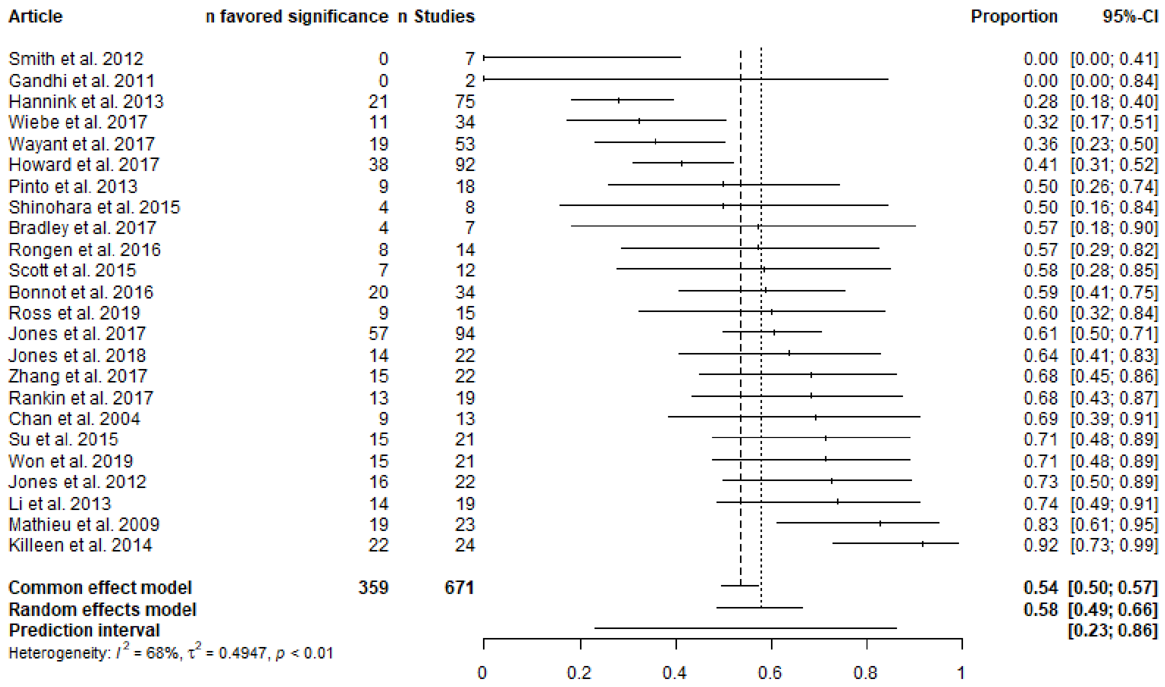

Supplementary Figure G2. Proportion of outcome discrepancies that favor statistically significant results.

For peer review only - http://bmjopen.bmj.com/site/about/guidelines.xhtml

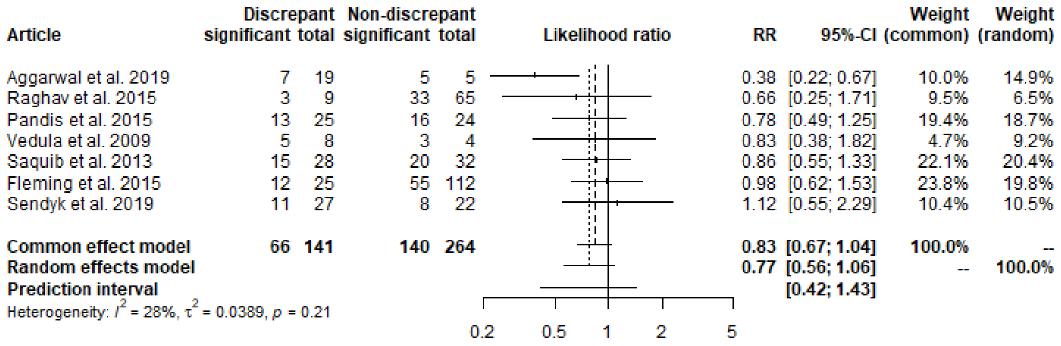

Supplementary Figure G3. Likelihood that a publication with at least one outcome discrepancy, compared to a publication with no outcome discrepancy, reports statistically significant results.

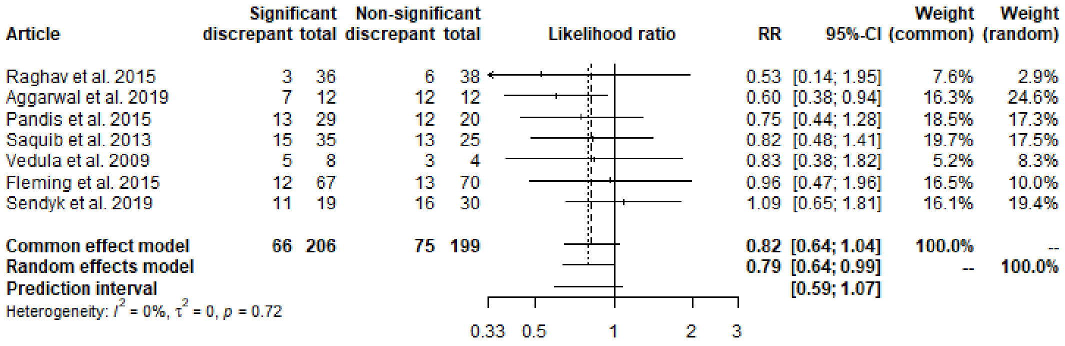

Supplementary Figure G4. Likelihood that a publication reporting a statistically significant result, compared to a publication not reporting a statistically significant results, contains at least one outcome discrepancy.

For peer review only - http://bmjopen.bmj.com/site/about/guidelines.xhtml

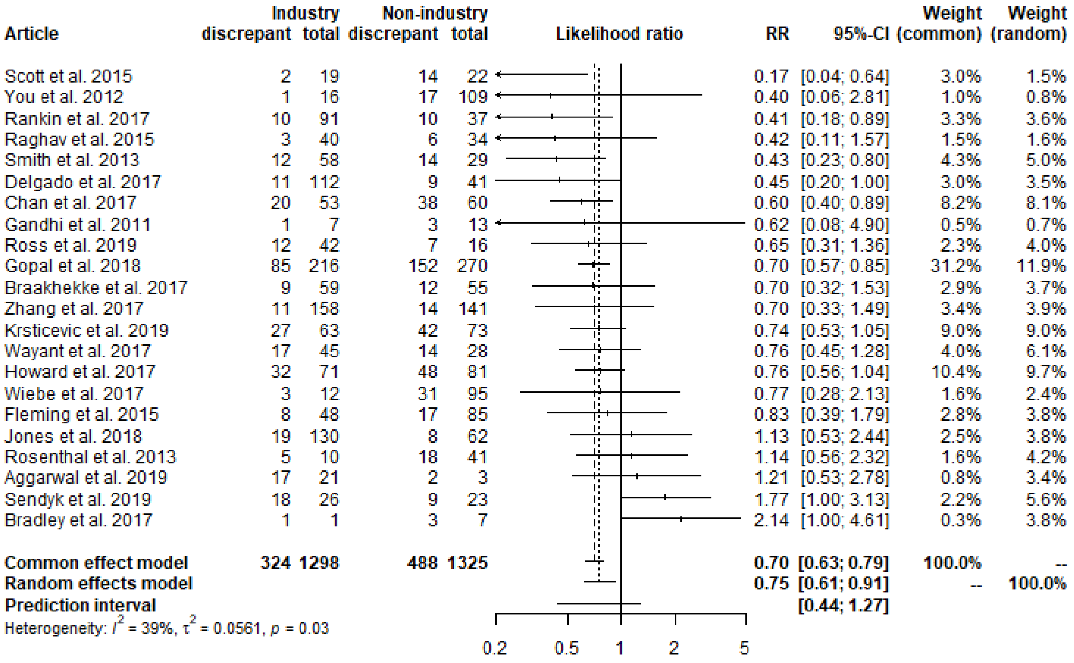

Supplementary Figure G5. Likelihood that a study funded by industry, compared to a study not funded by industry, includes at least one outcome discrepancy.

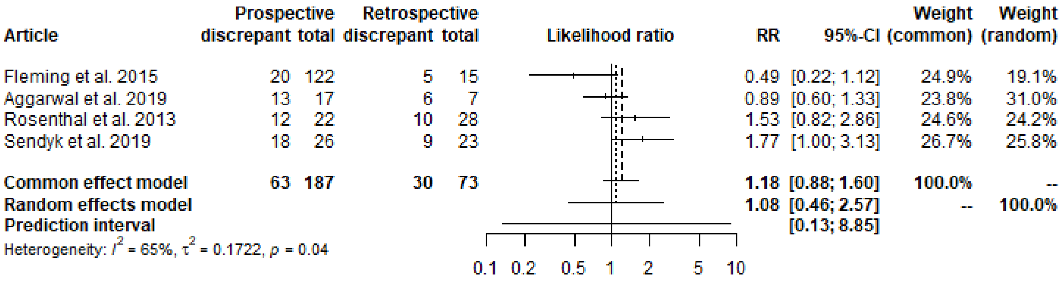

Supplementary Figure G6. Likelihood that a prospectively registered study, compared to a retrospectively registered study, includes at least one primary outcome discrepancy.

For peer review only - http://bmjopen.bmj.com/site/about/guidelines.xhtml

H. Non-outcome discrepancies

Eligibility

Discrepancies in eligibility were sometimes reported separately for inclusion and exclusion criteria. In this case, we took the larger of the two numbers because at least that many studies had a discrepancy in eligibility. Other definitions such as a "change in study population" were also used. While our meta-analysis (k = 15) estimates that 25-57% (95% CI) (95% prediction interval: 5-90%) of publications have at least one discrepancy in eligibility criteria, a broader definition of eligibility would yield an estimate in the higher end of this interval.

Sample size

Of the k = 25 articles that report on discrepancies in sample size in our meta-analysis, two required a sample size difference of at least 20%, 7 required a sample size difference of at least 10%, one required a sample size difference of at least 6%, and 15 had no mention of a minimum magnitude of change in sample size. A few articles reported both any discrepancy in sample size and discrepancies surpassing a specified magnitude. In these cases, we counted the discrepancies that reached the largest magnitude cut-off identified in the article. An estimated 26-44% (95% CI) (95% prediction interval: 8-78%) of studies had discrepancies in their sample size.

Randomization and blinding

Articles varied greatly in the way they counted discrepancies in randomization. One article surveyed for "a general statement about randomization" (Rosenthal & Dwan, 2013); one counted discrepancies in randomization if there was a change in the number of arms of a study (Sendyk et al., 2019); and one assessed only three studies with generated allocation sequences (Su et al., 2015). These articles found few discrepancies. Other articles counted studies that did not report randomization (Soares et al., 2004) or checked for discrepancies in the randomization procedure (Fleming et al., 2015). These articles found many discrepancies. Few studies include details about randomization in both their registry and publication (Fleming et al., 2015).

Only three of the k = 89 articles we reviewed reported discrepancies in blinding by checking who was blinded (Fleming et al., 2015; Rosenthal & Dwan, 2013; Su et al., 2015). Methods and results were less heterogeneous as compared to discrepancies in randomization.

Intervention

Seven articles reported on discrepancies in interventions. Three of these articles only counted high-level discrepancies (e.g., the name of the intervention) and found few discrepancies (Korevaar et al., 2014; Rosenthal & Dwan, 2013; Su et al., 2015). Three other articles counted more fine-grained discrepancies in interventions (e.g., dosage, duration of administration) and found that discrepancies were common (Hui'c et al., 2011; Juri'c et al., 2020; Prani'c & Maruši'c, 2016).

Study duration

Four articles reported on discrepancies in study duration. Although the definition of a discrepancy was similar among three of the studies, their results varied substantially—between 3% to 65% discrepant (Rosati et al., 2016; Rosenthal & Dwan, 2013; Shepshelovich et al., 2018).

For peer review only - http://bmjopen.bmj.com/site/about/guidelines.xhtml

Analyses and subgroup analyses

Twelve articles checked for discrepancies in analysis plans in general, and k = 9 checked specifically for discrepancies in subgroup analyses. Very few studies included a general analysis plan, or a subgroup analysis plan, in both their registration and publication (e.g., Fleming et al., 2015; Rosenthal & Dwan, 2013). Some articles we reviewed only evaluated discrepancies for studies that included an analysis in both their registration and publication. Three of the articles with low estimates for analysis discrepancies only checked whether plans to conduct intention-to-treat analyses were followed (Fleming et al., 2015; Rosati et al., 2016; Soares et al., 2004). Other articles checked for discrepancies in the statistical test for the primary outcome (Chan et al., 2008) or in model adjustments (Saquib et al., 2013). If articles evaluated a broader spectrum of discrepancies in the analyses they checked, and if they considered the addition of an analyses as a discrepancy, the resulting point estimate would be in the higher end of this interval: 19-52% (95% CI) (95% prediction interval: 4-86%). Discrepancies in subgroup analyses were more common: 35-93% (95% CI) (95% prediction interval: 2-99.7%).

Funding and results

Discrepancies in funding source and reported results are not directly relevant to the main theme of this paper because they do not imply a deviation from a prospectively planned study protocol. We coded for these discrepancies nonetheless, but the intervals are too wide to draw conclusions about their prevalence (Table 4).

For peer review only

For peer review only - http://bmjopen.bmj.com/site/about/guidelines.xhtml

I. Forest plots for all meta-analyses in Table 4 of the manuscript (non-outcome discrepancies)

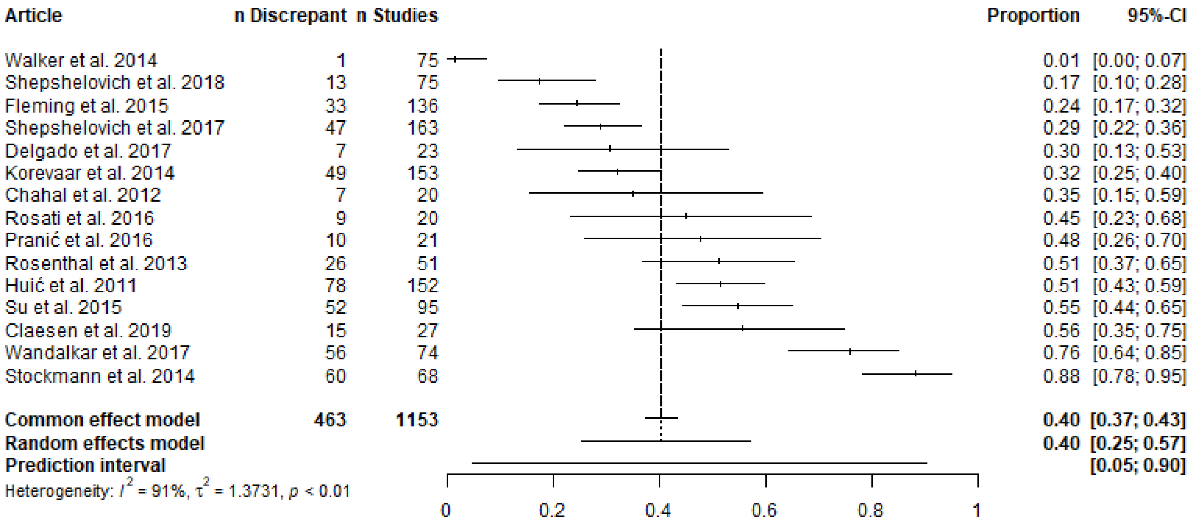

Supplementary Figure I1. Eligibility criteria.

For peer review only - http://bmjopen.bmj.com/site/about/guidelines.xhtml

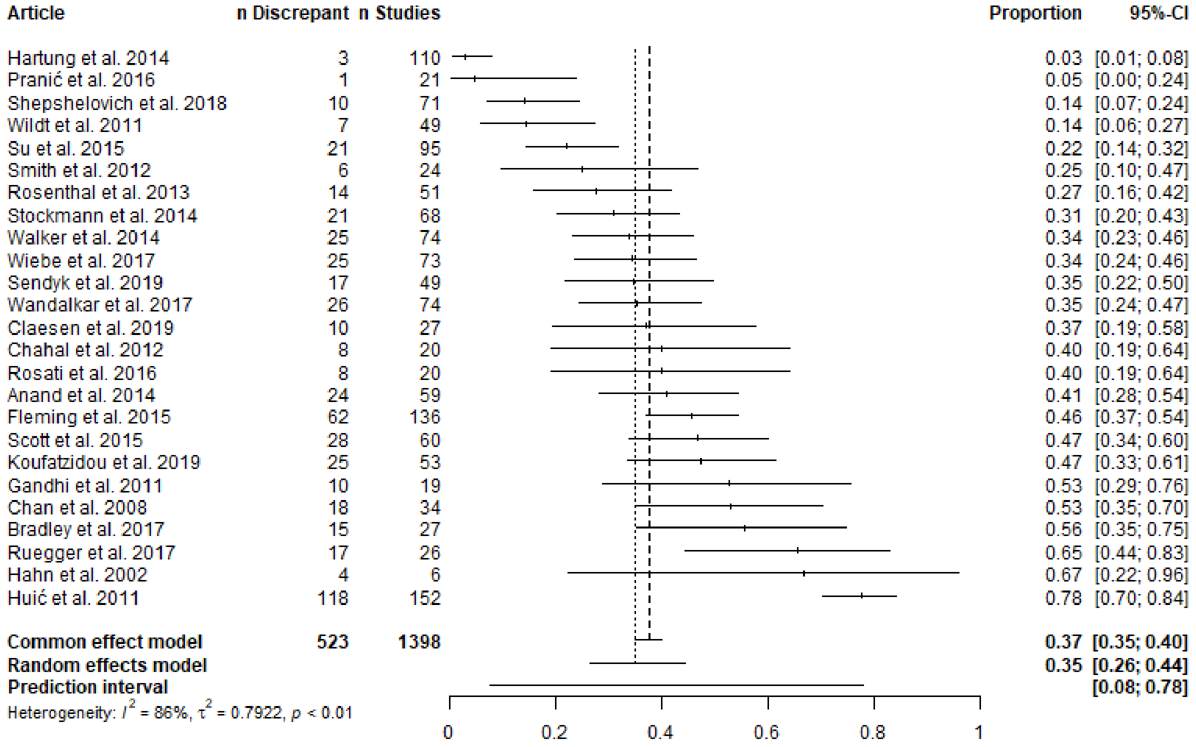

Supplementary Figure I2. Sample size.

For peer review only - http://bmjopen.bmj.com/site/about/guidelines.xhtml

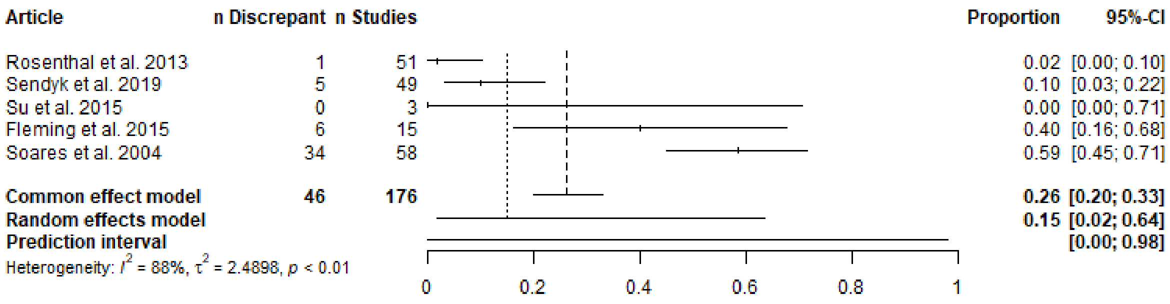

Supplementary Figure I3. Randomization.

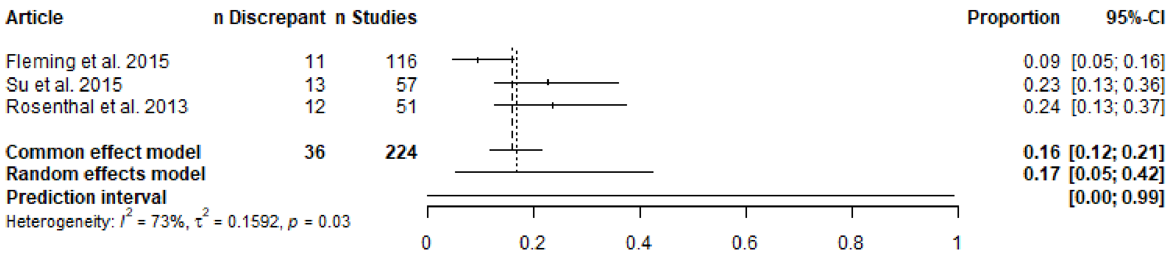

Supplementary Figure I4. Blinding.

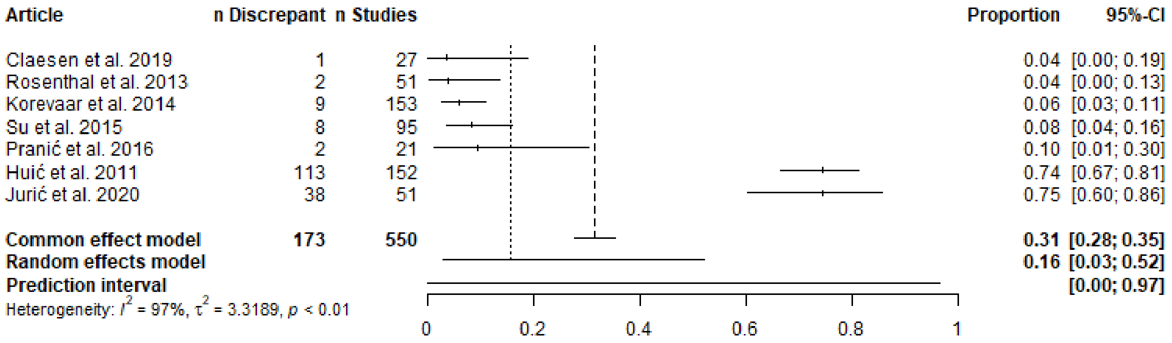

Supplementary Figure I5. Intervention.

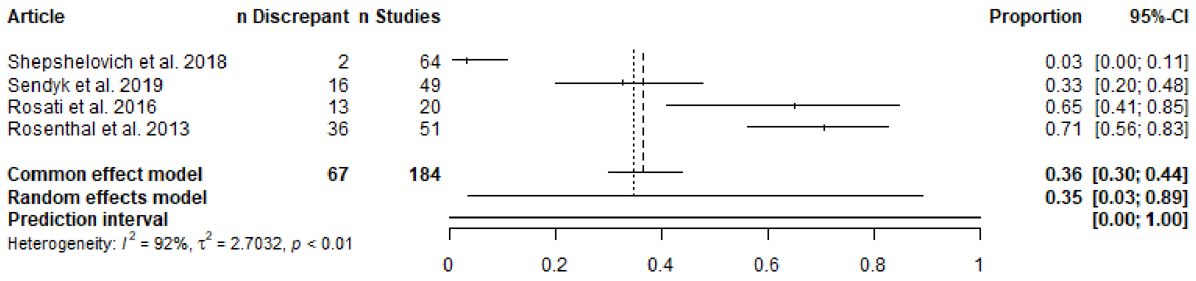

Supplementary Figure I6. Study Duration.

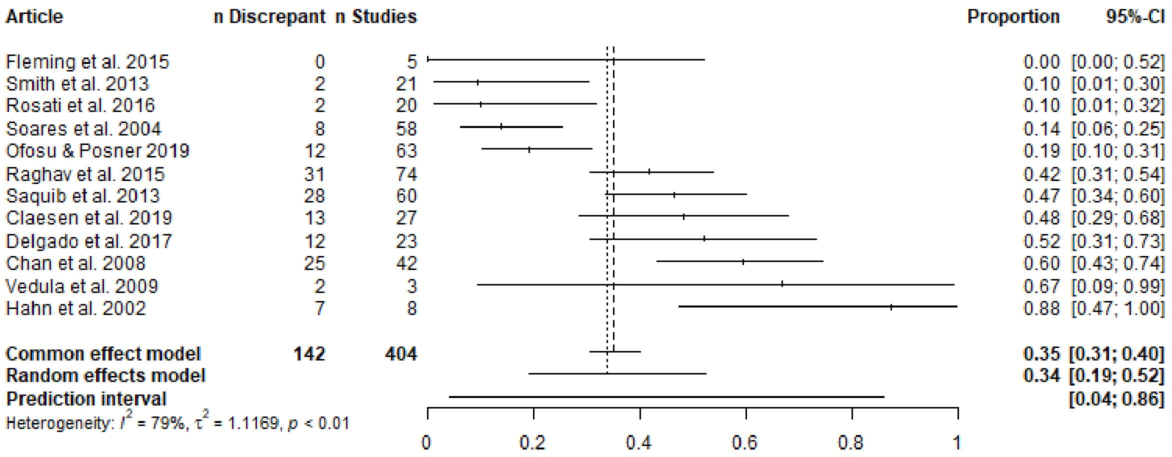

Supplementary Figure I7. Analyses.

For peer review only - http://bmjopen.bmj.com/site/about/guidelines.xhtml

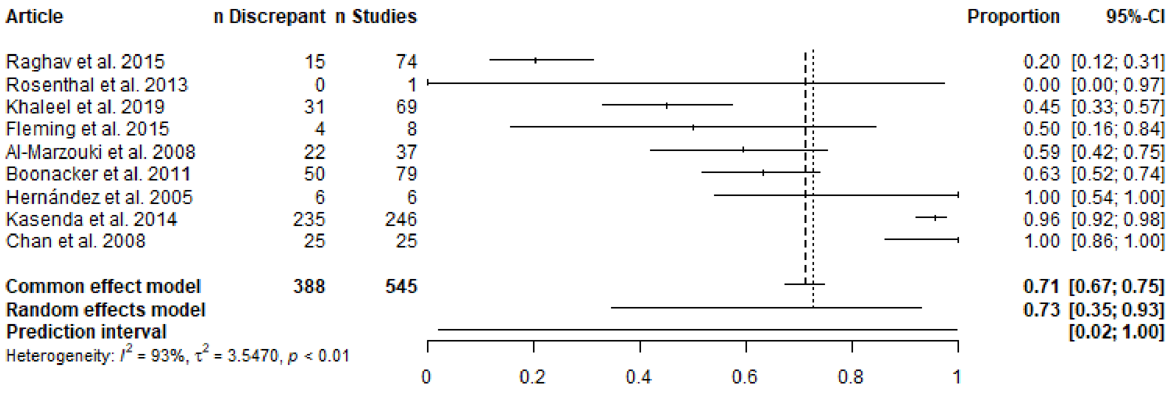

Supplementary Figure I8. Subgroup analyses.

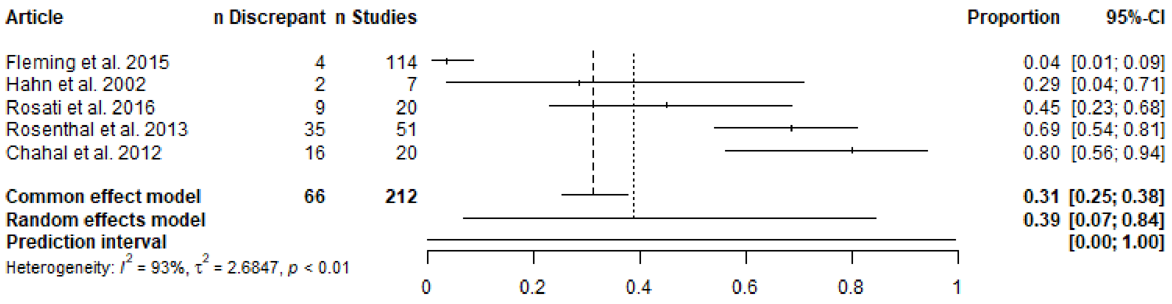

Supplementary Figure I9. Funding.

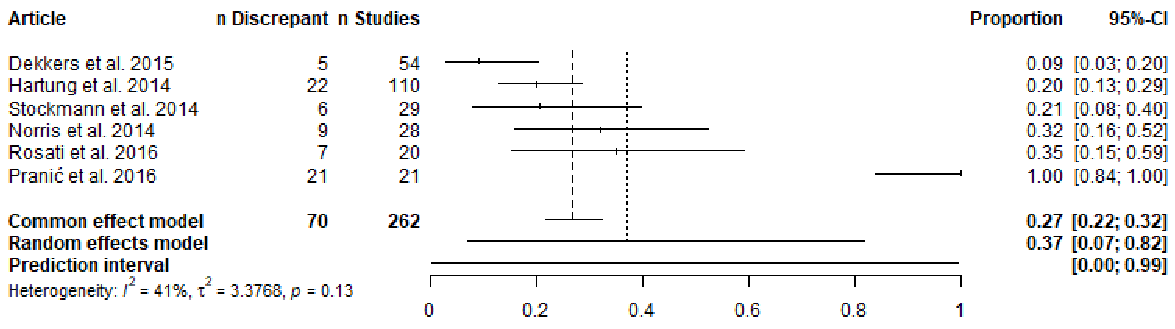

Supplementary Figure I10. Results.

For peer review only - http://bmjopen.bmj.com/site/about/guidelines.xhtml

J. Gaps in the literature

In addition to the quantitative data presented thus far, our reviewers took qualitative notes. These included a conclusion from each article, details regarding the source used to identify studies and the sub-disciplines they surveyed, and other *ad hoc* observations. One reviewer (RTT) extracted themes related to discrepancies and identified gaps in the literature using narrative synthesis. This process revealed several issues parallel to discrepancies: many studies are never registered, registrations are often imprecise, they rarely include information on analysis plans, and additional initiatives or enforcement may be necessary to improve registration in practice (see Supplementary Table J1 for a list of themes and article quotes).

We further identified at least four gaps in the literature: (1) discrepancy prevalence in fields other than clinical research, (2) discrepancy prevalence in a representative sample across clinical disciplines, (3) the level of detail in registrations, and (4) interventions that attempt to reduce undisclosed discrepancies.

Only one preprint surveyed each of psychology and economics, and no other article assessed preclinical or animal research, or sourced registrations from the Open Science Framework, AsPredicted.org, the American Economic Association (AEA) and Evidence in Governance and Politics (EGAP) registration platform, or the Registry for International Development Impact Evaluations (RIDIE). Notably, registration standards differ across disciplines and this necessitates different methods for identifying discrepancies. For example, pre-analysis plans on EGAP often contain over 10 hypotheses (Ofosu & Posner, 2019) and clinicaltrials.gov does not contain a question regarding an analysis plan.

Many articles we reviewed restricted their search to high-impact factor journals or a specific clinical discipline. Randomly sampling registrations and publications from the broader clinical literature could better estimate the prevalence of discrepancies at the level where some interventions could be applied (e.g., an ICMJE policy). With a sufficient sample size, random sampling could allow comparisons among clinical disciplines and the potential to identify best practices.

Most articles did not identify the level of specificity with which an outcome was registered. For example, outcomes can be specified at the level of domain (e.g., anxiety), specific measurement (e.g., Hamilton Anxiety Rating Scale), specific metric (e.g. change from baseline), and method of aggregation (e.g., proportion of participants with decrease >50%) (example taken from Deborah A. Zarin et al. (2011)). Outcomes registered with low specificity allow for a greater range of researcher degrees of freedom. Future research efforts can also benefit from identifying whether the registrations were prospective, which version of the registration was being assessed, and if discrepancies were disclosed. This information would help reveal how problematic the discrepancies are.

Finally, nearly all the articles focused on documenting discrepancies. Future efforts may be more fruitful if they attempt to understand the causes of discrepancies and seek to develop and evaluate solutions to the issue (e.g., following the framework for meta-research presented by Hardwicke, Serghiou, et al. (2020)).

For peer review only

For peer review only - http://bmjopen.bmj.com/site/about/guidelines.xhtml

Supplementary Table J1. Additional themes related to registration and discrepancies

Theme	Quotes
Many studies are never registered	"The more concerning issue, however, is the lack of registration or provision of registration number for randomized controlled trials within these journals." (Wiebe et al., 2017)<br>"Only 16 per cent of the published orthodontic RCTs had been registered." (Koufatzidou et al., 2019)
Registrations are imprecise	"Our results suggest that [selective outcome reporting] might well be substantial; however, the bias can only be broadly identified as protocols are not sufficiently precise." (Hahn et al., 2002)<br>"Only 5 out of the 109 trials (5%) provided enough information for us to be confident that the outcomes reported in the published trial were consistent with the original registration" (Nankervis et al., 2012)
Consumers of research should be aware of outcome switching	"If clinicians base their decisions on evidence distorted by primary outcome switching, patient care could be negatively affected" (C. W. Jones et al., 2017)<br>"Readers of medical literature should not assume that selective outcome reporting is not present, even if the report has been published in a high IF journal." (Shinohara et al., 2015)
Registration should be mandated or enforced	"there is insufficient quality control of data by data providers, ClinicalTrials.gov administrators, and journal editors, emphasizing much-needed enforcement of complete descriptions of drug interventions." (Jurić et al., 2020)<br>"Journal editors, regulators, research ethics committees, funders, and sponsors should implement policies mandating prospective registration for all clinical trials" (Chan et al., 2017)<br>"Reporting completeness and consistency were significantly better after July 2005. The ICMJE requirement for mandatory registration was associated with significant improvement in reporting quality in infectious diseases trials." (Shepshelovich et al., 2017)
Additional initiatives are needed to reduce reporting discrepancies	"Efforts to prevent nonregistration of protocols and selective reporting are needed." (Krsticevic et al., 2019)<br>"To foster credible evidence-based medicine, additional initiatives are needed to minimize selective reporting." (Raghav et al., 2015)<br>"Novel approaches are required to address this problem." (Fleming et al., 2015)
Analysis plans should be registered	"More immediate and specific remedies for the deficiencies of diet trial registries include (1) posting a detailed final statistical analysis plan before unmasking random group assignments or beginning data analyses as a minimum quality criterion..." (Ludwig et al., 2019)<br>"Definitive judgments regarding credibility of claimed subgroup effects are not possible without access to protocols and analysis plans of randomised controlled trials." (Kasenda et al., 2014)

For peer review only - http://bmjopen.bmj.com/site/about/guidelines.xhtml

PRISMA 2020 Checklist

Section and Topic	Item #	Checklist item	Location where item is reported
**TITLE**			
Title	1	Identify the report as a systematic review.	Title
**ABSTRACT**			
Abstract	2	See the PRISMA 2020 for Abstracts checklist.	Abstract
**INTRODUCTION**			
Rationale	3	Describe the rationale for the review in the context of existing knowledge.	"In 2000, ClinicalTrials.gov and the ISRCTN Registry were launched with several aims, including aiding participant recruitment, facilitating knowledge synthesis, and reducing duplication, publication bias and selective reporting (Zarin et al., 2017). In 2005, the International Committee of Medical Journal Editors (ICMJE) made prospective registration a condition of consideration for publication (De Angelis et al., 2004). Thousands of journals now claim to follow this policy (ICMJE, 2021). In parallel, the World Health Organization International Clinical Trials Registry Platform established a minimum set of required information for a trial to be considered fully registered, including experimental design elements such as the conditions being studied, intervention, key inclusion and exclusion criteria, sample size, primary outcomes, and key secondary outcomes (Sim et al., 2006). While the relatively widespread uptake of clinical trial registration has substantially improved transparency, many trials remain unregistered, are registered after enrollment of participants begins or analyses are complete (i.e., retrospective registration), are never published, or publish outcomes discrepant with those in the registration without disclosing the discrepancy (e.g., Chan et al., 2017; Scott et al., 2015). Nevertheless, the existence of registries allows researchers to identify and quantify these issues."
Objectives	4	Provide an explicit statement of the objective(s) or question(s) the review addresses.	"Here we systematically review articles that quantify the prevalence of discrepancies between registrations or study protocols and their associated publications (e.g., in primary outcome measures)."
**METHODS**			
Eligibility criteria	5	Specify the inclusion and exclusion criteria for the review and how studies were grouped for the syntheses.	"We excluded conference proceedings and articles written in a language other than English (for full inclusion and exclusion criteria, see our protocol osf.io/ktmdg)."
Information sources	6	Specify all databases, registers, websites, organisations, reference lists and other sources searched or consulted to identify studies. Specify the date when each source was last searched or consulted.	"We searched Scopus and Web of Science on 15th December 2019 using the queries in Appendix A and Appendix B of our preregistered protocol osf.io/ktmdg."
Search strategy	7	Present the full search strategies for all databases, registers and websites, including any filters and limits used.	"We searched Scopus and Web of Science on 15th December 2019 using the queries in Appendix A and Appendix B of our preregistered protocol osf.io/ktmdg."
Selection process	8	Specify the methods used to decide whether a study met the inclusion criteria of the review, including how many reviewers screened each record and each report retrieved, whether they worked independently, and if applicable, details of automation tools used in the process.	See manuscript sections *Eligibility criteria* and *Study selection*, as well as the included PRISMA flowchart.
Data collection	9	Specify the methods used to collect data from reports, including how many reviewers	See manuscript section *Study Selection*

For peer review only - http://bmjopen.bmj.com/site/about/guidelines.xhtml

PRISMA 2020 Checklist

Section and Topic	Item #	Checklist item	Location where item is reported
process		collected data from each report, whether they worked independently, any processes for obtaining or confirming data from study investigators, and if applicable, details of automation tools used in the process.	
Data items	10a	List and define all outcomes for which data were sought. Specify whether all results that were compatible with each outcome domain in each study were sought (e.g. for all measures, time points, analyses), and if not, the methods used to decide which results to collect.	"Each included article was independently coded by two of four reviewers (RTT, RC, OvdA, SW) using a coding form designed for this review. The form consisted of five sections that assessed (1) article characteristics, (2) study registration details, (3) 10 types of outcome discrepancies (listed in Table 2), (4) 10 types of non-outcome discrepancies (listed in Table 4), and (5) any additional descriptive or inferential statistics on discrepancies. The form details the operationalization of each variable we coded, and is available at osf.io/728ys. We chose items to code based on a pilot test of our protocol, as well as the categories used in a seminal paper [10] and a recent systematic review on discrepancies [7]."
	10b	List and define all other variables for which data were sought (e.g. participant and intervention characteristics, funding sources). Describe any assumptions made about any missing or unclear information.	"To be included in our meta-analyses this data had to include at least two of (1) the number of studies assessed (denominator), (2) the number of studies with a given discrepancy (numerator), and (3) the percentage of studies with a given discrepancy—form which we could calculate an unreported numerator or denominator. We did not access the raw data. If an article did not report data for a certain measure (e.g., secondary outcome discrepancies), then we did not include that article in the meta-analysis for that measure (this is why k varies among the meta-analyses we present —see Tables 2 and 4)."
Study risk of bias assessment	11	Specify the methods used to assess risk of bias in the included studies, including details of the tool(s) used, how many reviewers assessed each study and whether they worked independently, and if applicable, details of automation tools used in the process.	"Whereas we did not perform a formal risk of bias assessment—because our review differed substantially from the purpose these tools were built for—we shed light on a few potential sources of bias with additional analyses that consider the funding source, statistical significance, and the timing of registration of included studies. " … "Each included article was independently coded by two of four reviewers (RTT, RC, OvdA, SW) using a coding form designed for this review. The form is available at (osf.io/sc8p6/) and details the operationalization of each variable we coded."
Effect measures	12	Specify for each outcome the effect measure(s) (e.g. risk ratio, mean difference) used in the synthesis or presentation of results.	Included in all figures and tables.
Synthesis methods	13a	Describe the processes used to decide which studies were eligible for each synthesis (e.g. tabulating the study intervention characteristics and comparing against the planned groups for each synthesis (item #5)).	"We included articles that reported quantitative data about discrepancies between registrations or study protocols and their associated publication."
	13b	Describe any methods required to prepare the data for presentation or synthesis, such as handling of missing summary statistics, or data conversions.	"To be included in our meta-analyses this data had to include at least two of (1) the number of studies assessed (denominator), (2) the number of studies with a given discrepancy (numerator), and (3) the percentage of studies with a given discrepancy—form which we could calculate an unreported numerator or denominator. We did not access the raw data. If an article did not report data for a certain measure (e.g., secondary outcome discrepancies), then we did not include that article in the meta-analysis for that measure (this is why k varies among the meta-analyses we present —see Tables 2 and 4)."
	13c	Describe any methods used to tabulate or visually display results of individual studies and syntheses.	Data on individual studies is available in our dataset which is available as open data. We do not tabulate data from individual studies within the manuscript.

For peer review only

For peer review only - http://bmjopen.bmj.com/site/about/guidelines.xhtml

PRISMA 2020 Checklist

Section and Topic	Item #	Checklist item	Location where item is reported
	13d	Describe any methods used to synthesize results and provide a rationale for the choice(s). If meta-analysis was performed, describe the model(s), method(s) to identify the presence and extent of statistical heterogeneity, and software package(s) used.	See section *Statistical analyses*.
	13e	Describe any methods used to explore possible causes of heterogeneity among study results (e.g. subgroup analysis, meta-regression).	See section *Statistical analyses*.
	13f	Describe any sensitivity analyses conducted to assess robustness of the synthesized results.	See section *Statistical analyses*.
Reporting bias assessment	14	Describe any methods used to assess risk of bias due to missing results in a synthesis (arising from reporting biases).	"Whereas we did not perform a formal risk of bias assessment—because our review differed substantially from the purpose these tools were built for—we shed light on a few potential sources of bias with additional analyses that consider the funding source, statistical significance, and the timing of registration of included studies."
Certainty assessment	15	Describe any methods used to assess certainty (or confidence) in the body of evidence for an outcome.	We present prediction intervals, confidence intervals, and heterogeneity.
**RESULTS**			
Study selection	16a	Describe the results of the search and selection process, from the number of records identified in the search to the number of studies included in the review, ideally using a flow diagram.	See included PRISMA flowchart.
	16b	Cite studies that might appear to meet the inclusion criteria, but which were excluded, and explain why they were excluded.	We did not identify studies that meet this criteria. Also: "(the list of articles and coding is available at osf.io/wa62f)."
Study characteristics	17	Cite each included study and present its characteristics.	See section *Article characteristics*. All included articles are in the reference list and listed in the open data.
Risk of bias in studies	18	Present assessments of risk of bias for each included study.	"Whereas we did not perform a formal risk of bias assessment—because our review differed substantially from the purpose these tools were built for—we shed light on a few potential sources of bias with additional analyses that consider the funding source, statistical significance, and the timing of registration of included studies."
Results of individual studies	19	For all outcomes, present, for each study: (a) summary statistics for each group (where appropriate) and (b) an effect estimate and its precision (e.g. confidence/credible interval), ideally using structured tables or plots.	See forest plots which include confidence intervals and a prediction interval.
Results of	20a	For each synthesis, briefly summarise the characteristics and risk of bias among	See sections *Primary outcome discrepancies* and *Secondary outcome discrepancies.*

For peer review only

PRISMA 2020 Checklist

Section and Topic	Item #	Checklist item	Location where item is reported
syntheses		contributing studies.	
	20b	Present results of all statistical syntheses conducted. If meta-analysis was done, present for each the summary estimate and its precision (e.g. confidence/credible interval) and measures of statistical heterogeneity. If comparing groups, describe the direction of the effect.	See forest plots in main manuscript as well as Supplementary Materials.
	20c	Present results of all investigations of possible causes of heterogeneity among study results.	Meta-regression results are included in the sections *Primary outcome discrepancies* and *Secondary outcome discrepancies.*
	20d	Present results of all sensitivity analyses conducted to assess the robustness of the synthesized results.	“We also conducted a sensitivity analysis that included all six regressors in a single meta-regression and found that publications compared to the most recent version of a registration (k = 15) may have a smaller proportion of studies with at least one primary outcome discrepancy as compared to publications compared to the original version of a registration (k = 29) (p = 0.08; OR 95% CI: 0.32 to 1.07). All meta-regression model summaries are presented in Supplementary Material E.”
Reporting biases	21	Present assessments of risk of bias due to missing results (arising from reporting biases) for each synthesis assessed.	We feel this item is not relevant to the subject matter of our systematic review.
Certainty of evidence	22	Present assessments of certainty (or confidence) in the body of evidence for each outcome assessed.	We present prediction intervals and confidence intervals throughout.
**DISCUSSION**			
Discussion	23a	Provide a general interpretation of the results in the context of other evidence.	“Our main findings are in line with those from previous systematic reviews. These reviews included 27 articles each and found that 31% of studies had a primary outcome discrepancy in the median article they reviewed (C. W. Jones et al., 2015) and 54% of studies had any outcome discrepancy in the median article they reviewed (G. Li et al., 2018). The latter review did not distinguish between primary and secondary outcomes, and many articles they reviewed only assessed primary outcomes. Our review included all the articles contained in these systematic reviews, except for a few that did not meet our inclusion criteria (e.g., a PhD thesis, an abstract).”
	23b	Discuss any limitations of the evidence included in the review.	“We find that outcome measures in registrations and study protocols often differ from published outcome measures, that the prevalence of discrepancies varies substantially across the articles we reviewed, and that this heterogeneity is not easily assigned to specific article characteristics. Given the wide range of discrepancy prevalence across individual articles, point estimates and confidence intervals may provide false precision when extrapolating our findings to the registered literature at large. Moreover, because heterogeneity could not be explained by meta-regression of article characteristics, more precise estimates cannot be derived for subsets of the literature. The prediction intervals can reasonably be used to extrapolate to the registered medical literature at large, although the included studies do not necessarily form a representative sample.”<br>Also see *Limitations* section of the Discussion.
	23c	Discuss any limitations of the review processes used.	See response to previous box.
	23d	Discuss implications of the results for practice, policy, and future research.	See *Implications* section of the Discussion.

For peer review only - http://bmjopen.bmj.com/site/about/guidelines.xhtml

PRISMA 2020 Checklist

Section and Topic	Item #	Checklist item	Location where item is reported
**OTHER INFORMATION**			
Registration and protocol	24a	Provide registration information for the review, including register name and registration number, or state that the review was not registered.	"Registration. osf.io/ktmdg. Protocol amendments are listed in Supplementary Material A."
	24b	Indicate where the review protocol can be accessed, or state that a protocol was not prepared.	"Registration. osf.io/ktmdg. Protocol amendments are listed in Supplementary Material A."…"Each included article was independently coded by two of four reviewers (RTT, RC, OvdA, SW) using a coding form designed for this review. The form details the operationalization of each variable we coded, and is available at osf.io/728ys. The complete dataset, including the coding of each reviewer and the resolved coding, is available at osf.io/ue2c6. A cleaned dataset with only the resolved coding is available at osf.io/6cn9m."
	24c	Describe and explain any amendments to information provided at registration or in the protocol.	**"Registration**. osf.io/ktmdg. Protocol amendments are listed in Supplementary Material A."
Support	25	Describe sources of financial or non-financial support for the review, and the role of the funders or sponsors in the review.	See *Funding* section.
Competing interests	26	Declare any competing interests of review authors.	"None declared"
Availability of data, code and other materials	27	Report which of the following are publicly available and where they can be found: template data collection forms; data extracted from included studies; data used for all analyses; analytic code; any other materials used in the review.	All data and materials are openly shared on the Open Science Framework at www.osf.io/5gfty

From: Page MJ, McKenzie JE, Bossuyt PM, Boutron I, Hoffmann TC, Mulrow CD, et al. The PRISMA 2020 statement: an updated guideline for reporting systematic reviews. BMJ 2021;372:n71. doi: 10.1136/bmj.n71

For more information, visit: http://www.prisma-statement.org/

For peer review only

PRISMA 2020 for Abstracts Checklist

Section and Topic	Item #	Checklist item	Reported (Yes/No)
**TITLE**			
Title	1	Identify the report as a systematic review.	Yes
**BACKGROUND**			
Objectives	2	Provide an explicit statement of the main objective(s) or question(s) the review addresses.	Yes
**METHODS**			
Eligibility criteria	3	Specify the inclusion and exclusion criteria for the review.	Yes
Information sources	4	Specify the information sources (e.g. databases, registers) used to identify studies and the date when each was last searched.	Yes
Risk of bias	5	Specify the methods used to assess risk of bias in the included studies.	No. We did not perform a formal risk of bias assessment because our review differed substantially from the purpose these tools were built for. We address this point in the main text of the manuscript.
Synthesis of results	6	Specify the methods used to present and synthesise results.	Yes
**RESULTS**			
Included studies	7	Give the total number of included studies and participants and summarise relevant characteristics of studies.	Yes
Synthesis of results	8	Present results for main outcomes, preferably indicating the number of included studies and participants for each. If meta-analysis was done, report the summary estimate and confidence/credible interval. If comparing groups, indicate the direction of the effect (i.e. which group is favoured).	Yes
**DISCUSSION**			
Limitations of evidence	9	Provide a brief summary of the limitations of the evidence included in the review (e.g. study risk of bias, inconsistency and imprecision).	Yes
Interpretation	10	Provide a general interpretation of the results and important implications.	Yes
**OTHER**			
Funding	11	Specify the primary source of funding for the review.	No. There was no funding provided specifically for this review. This information is included after the main text.
Registration	12	Provide the register name and registration number.	Yes

For peer review only

PRISMA 2020 for Abstracts Checklist

From: Page MJ, McKenzie JE, Bossuyt PM, Boutron I, Hoffmann TC, Mulrow CD, et al. The PRISMA 2020 statement: an updated guideline for reporting systematic reviews. BMJ 2021;372:n71. doi: 10.1136/bmj.n71

For more information, visit: http://www.prisma-statement.org/

For peer review only

MOOSE (Meta-analyses Of Observational Studies in Epidemiology) Checklist

A reporting checklist for Authors, Editors, and Reviewers of Meta-analyses of Observational Studies. You must report the page number in your manuscript where you consider each of the items listed in this checklist. If you have not included this information, either revise your manuscript accordingly before submitting or note N/A.

Reporting Criteria	Reported (Yes/No)	Reported on Page No.
**Reporting of Background**		
Problem definition		
Hypothesis statement		
Description of Study Outcome(s)		
Type of exposure or intervention used		
Type of study design used		
Study population		
**Reporting of Search Strategy**		
Qualifications of searchers (eg, librarians and investigators)		
Search strategy, including time period included in the synthesis and keywords		
Effort to include all available studies, including contact with authors		
Databases and registries searched		
Search software used, name and version, including special features used (eg, explosion)		
Use of hand searching (eg, reference lists of obtained articles)		
List of citations located and those excluded, including justification		
Method for addressing articles published in languages other than English		
Method of handling abstracts and unpublished studies		
Description of any contact with authors		
**Reporting of Methods**		
Description of relevance or appropriateness of studies assembled for assessing the hypothesis to be tested		
Rationale for the selection and coding of data (eg, sound clinical principles or convenience)		
Documentation of how data were classified and coded (eg, multiple raters, blinding, and interrater reliability)		
Assessment of confounding (eg, comparability of cases and controls in studies where appropriate		

For peer review only

Reporting Criteria	Reported (Yes/No)	Reported on Page No.
Assessment of study quality, including blinding of quality assessors; stratification or regression on possible predictors of study results		
Assessment of heterogeneity		
Description of statistical methods (eg, complete description of fixed or random effects models, justification of whether the chosen models account for predictors of study results, dose-response models, or cumulative meta-analysis) in sufficient detail to be replicated		
Provision of appropriate tables and graphics		
**Reporting of Results**		
Table giving descriptive information for each study included		
Results of sensitivity testing (eg, subgroup analysis)		
Indication of statistical uncertainty of findings		
**Reporting of Discussion**		
Quantitative assessment of bias (eg, publication bias)		
Justification for exclusion (eg, exclusion of non–English-language citations)		
Assessment of quality of included studies		
**Reporting of Conclusions**		
Consideration of alternative explanations for observed results		
Generalization of the conclusions (ie, appropriate for the data presented and within the domain of the literature review)		
Guidelines for future research		
Disclosure of funding source		

Once you have completed this checklist, please save a copy and upload it as part of your submission. DO NOT include this checklist as part of the main manuscript document. It must be uploaded as a separate file.

For peer review only
